# Design, synthesis, and biological evaluation of new pyrimidine-5-carbonitrile derivatives as novel anti-cancer, dual EGFR^WT^/COX-2 inhibitors with docking studies†

**DOI:** 10.1039/d3ra06088h

**Published:** 2023-11-02

**Authors:** Nada Reda, Ahmed Elshewy, Hesham I. EL-Askary, Khaled O. Mohamed, Amira A. Helwa

**Affiliations:** a Pharmaceutical Organic Chemistry Department, College of Pharmaceutical Sciences and Drug Manufacturing, Misr University for Science and Technology (MUST) 6th of October City Egypt amira.helwa@must.edu.eg Nada.reda@must.edu.eg; b Pharmaceutical Organic Chemistry Department, Faculty of Pharmacy, Cairo University Kasr El-Aini Street Cairo 11562 Egypt khaled.mohamed@pharma.cu.edu.eg ahmed.elshewy@pharma.cu.edu.eg; c Department of Medicinal Chemistry, Faculty of Pharmacy, Galala University New Galala 43713 Egypt; d Pharmaceutical Chemistry Department, Faculty of Pharmacy. Sinai University (Arish Branch) El Arish Egypt; e Department of Pharmacognosy, Faculty of Pharmacy, Cairo University Kasr El-Aini Street Cairo 11562 Egypt hesham.elaskary@pharma.edu.eg

## Abstract

A novel series of pyrimidine-5-carbonitrile derivatives was designed, synthesized, then evaluated for their cytotoxic activity as novel anti-cancer with dual EGFR^WT^/COX-2 inhibitors. Two compounds 4e and 4f disclosed the highest activity against all NCI60 panel cell lines. They were most potent against Colo 205 (IC_50_ = 1.66, and 1.83 μM), Sequentially. The most potent two compounds disturbed cell cycle of Colo-205 cells by blocking the G1 phase, coupled with increased annexin-Vstained cells which indicated the increasing in percentage of apoptosis. In addition, 4e and 4f increase the concentration of caspase-3 by 10, and 8-fold compared to control, respectively. Moreover, the two candidate compounds were screened for cytotoxicity on normal epithelial colon cells; fortunately, they were found to be safe. Molecular docking study displayed that these compounds bound to the active site as EGFR^WT^/COX-2 inhibitors. Furthermore, 3D pharmacophore mapping disclosed many shared features between the most potent candidates 4e and 4f and the standard EGFR^WT^/COX-2 inhibitors; erlotinib, and celecoxib, respectively. Finally, the physicochemical parameter was calculated for the most potent novel anticancer candidates and the SwissAdme parameter showed that the newly synthesized compounds have good drug-likeness properties.

## Abstract 2

A series of pyrimidine-5-carbonitriles was designed and synthesized as potential anti-cancer agents with dual EGFR^WT^/COX-2 inhibition. Compounds 4e and 4f disclosed the highest activity on all NCI60 cell lines.

## Graphical abstract

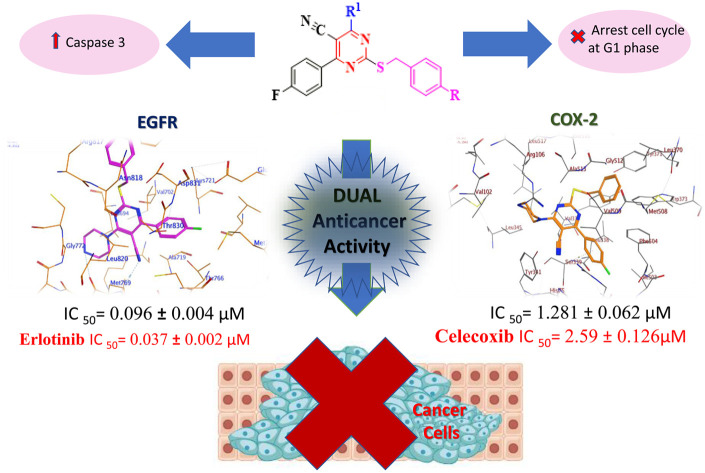

## Introduction

One of the most common causes of death in the world is cancer, a complex disease defined by uncontrolled cell development.^1^ Practitioners are currently choosing a variety of chemotherapeutic drugs, either alone or in combination, to treat a variety of malignancies with a human origin.^2^ As a target for the development of anticancer therapies, the tyrosine kinase EGFR has received substantial research.^3–5^ EGFR has been identified as a crucial regulator of numerous biological processes, including cell cycle progression, apoptosis inhibition, and tumor cell motility.^6^ Thus, suppression of the EGFR protein kinase is regarded as a promising anti-cancer treatment strategy for many types of cancer.^7^ There are two major ways to stop the oncogenic EGFR tyrosine kinase activity. The first tactic is the use of monoclonal antibodies, or “mabs,” which are intended to block the extracellular receptor domain. The second strategy involves the use of small-molecule substances that block the intracellular EGFR tyrosine kinase activity.^8,9^

**Fig. 1:**
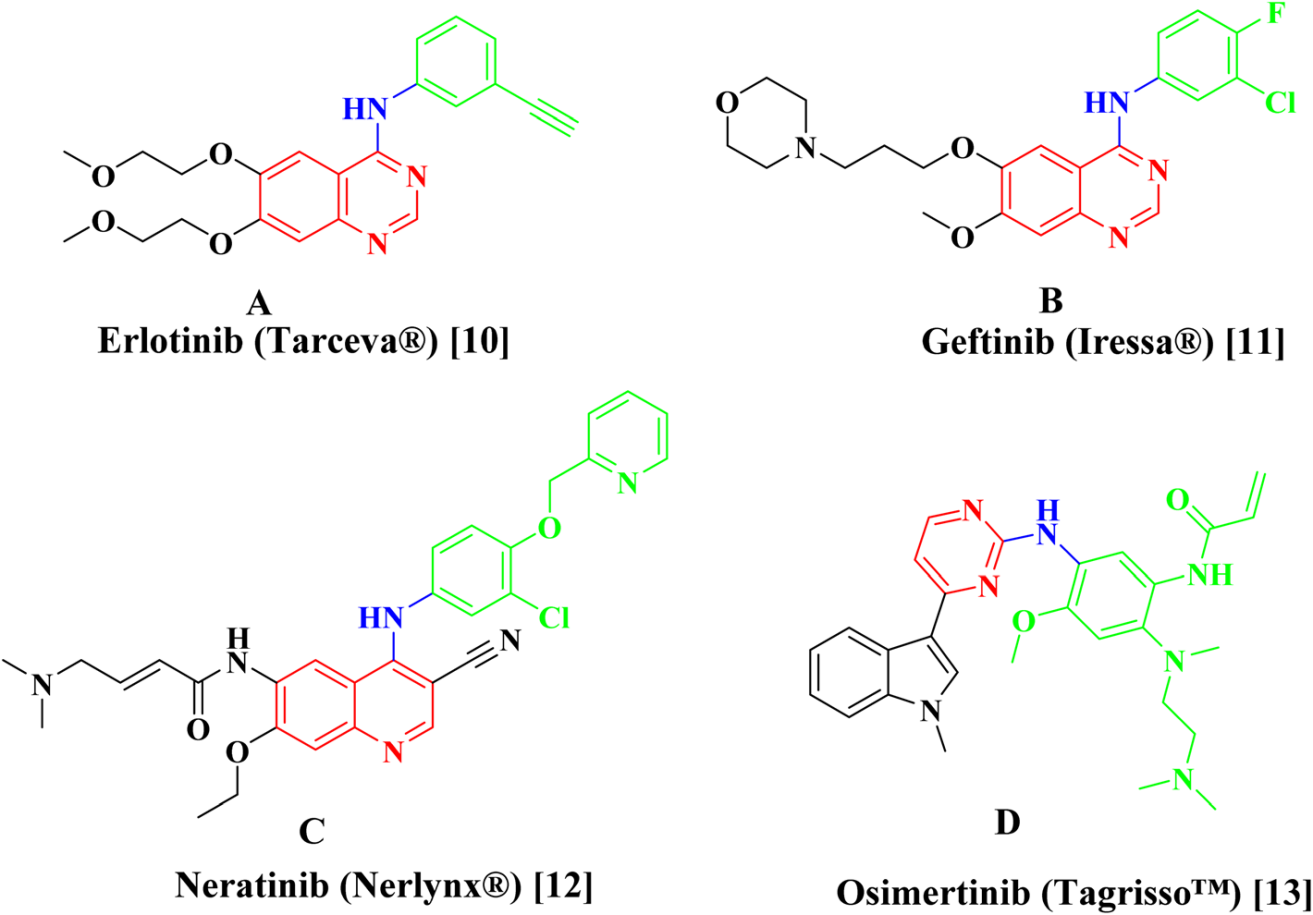
Examples of approved EGFR inhibitors.

Erlotinib A,^10^ Geftinib B,^11^ Neratinib C,^12^ and Osimertinib D (ref. 13) (Fig. 1), are EGFR-TK inhibitors approved from FDA and clinically used have been found to be extremely successful both alone and in combination therapy for treating a range of metastatic cancers. The drawbacks of the present strategy, such as its adverse effect profile, resistance to already available treatments, and low bioavailability profile highlight the pressing need for the creation of novel anticancer drugs.

When the host is defending itself against an injury or illness, inflammation is a physiologic response.^14^ Even while it has a preventive function, prolonged and excessive inflammation can occasionally cause tissue damage and a chronic state of inflammation.^15,16^ Literature offers verifiable proof in favor of the link between cancer and underlying inflammation.^17,18^

Interleukin 6 and tumor necrosis factor, two inflammatory cytokines, are known to greatly enhance tumor survival, proliferation, and angiogenesis.^19^ There are three different COX isoenzymes: COX-1, COX-2, and COX-3 that are in charge of producing the prostaglandins (PGs) PGD2, PGE2 Thromboxane A2, PGF2, and PGI2 through the arachidonic acid pathway way leading to the inflammatory response.^20^ PGs have an impact on a number of crucial normal biological processes, including angiogenesis, cell proliferation, and apoptosis.

In normal cells, the inducible enzyme COX-2 has almost very little cellular expression. But it has been measured in various cancer subtypes that COX-2 is overexpressed. In order to create a microenvironment that promotes cancer, a number of cancer-associated cells, including fibroblasts and macrophage type 2 cells, secrete COX-2. In turn COX-2 encourages apoptotic resistance, inflammation, and proliferative behaviour of cancer cells, angiogenesis, metastasis, and invasion. Hence, inhibition of COX-2 is regarded as a strategy in cancer treatment.^21^

**Fig. 2:**
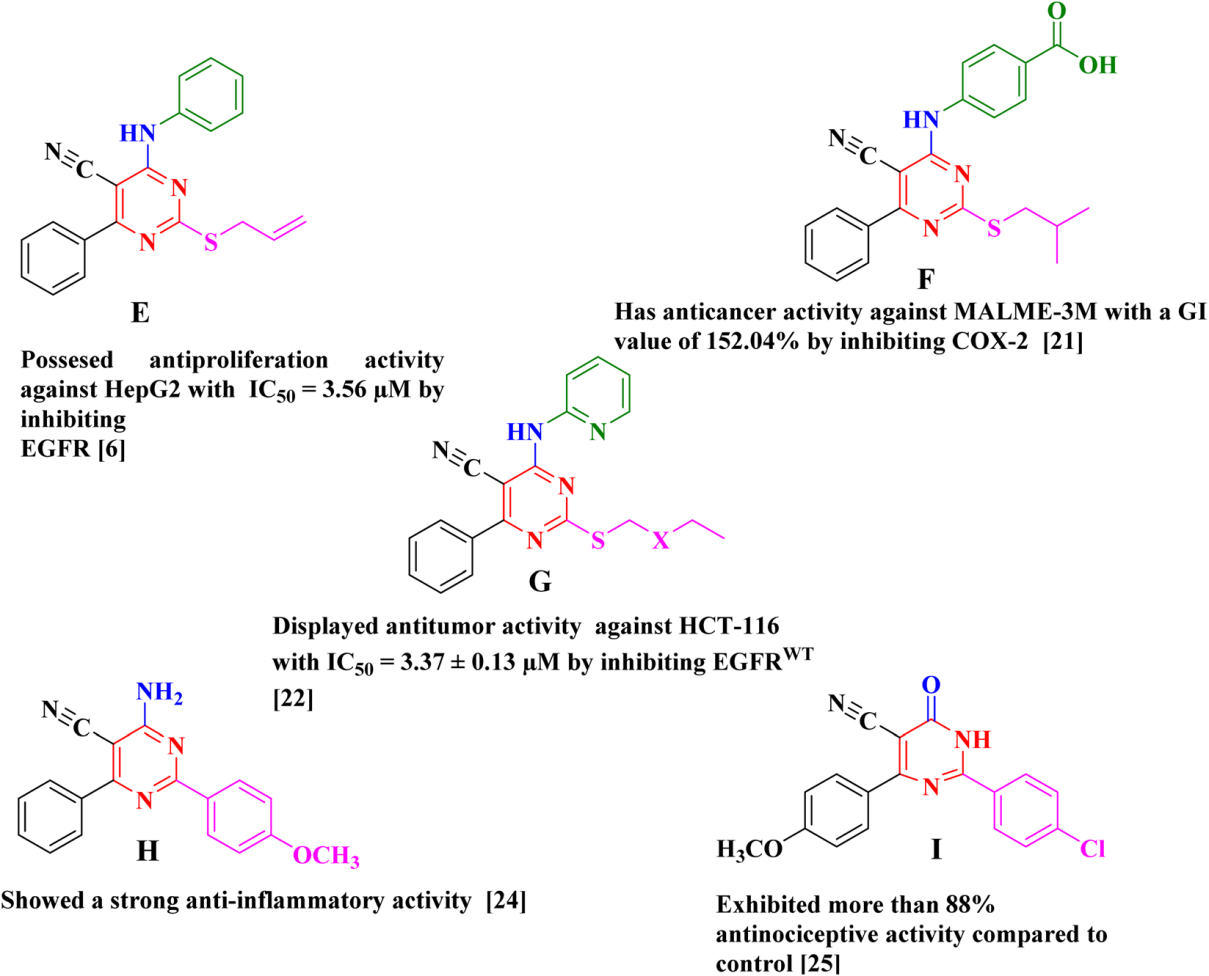
Example of antitumor and anti-inflammatory agents with pyrimidine-5-carbonitril Scaffold.

There have been several reports of pyrimidine-5-carbonitrile derivatives having cytotoxic action against various tumor cell lines by inhibiting the tyrosine kinase EGFR or COX-2 (Fig. 2).^6,21,22^ Also, pyrimidine-5-carbonitriles have also gotten recognition for their anti-inflammatory and analgesic activities through COX isoenzymes inhibition (Fig. 2).^23–26^ From the above we decided to synthesise a novel series of pyrimidine-5-carbonitrile as potential anticancer agent by targeting EGFR^WT^/COX-2.

## Results and discussions

### Chemistry

**Scheme 1:**
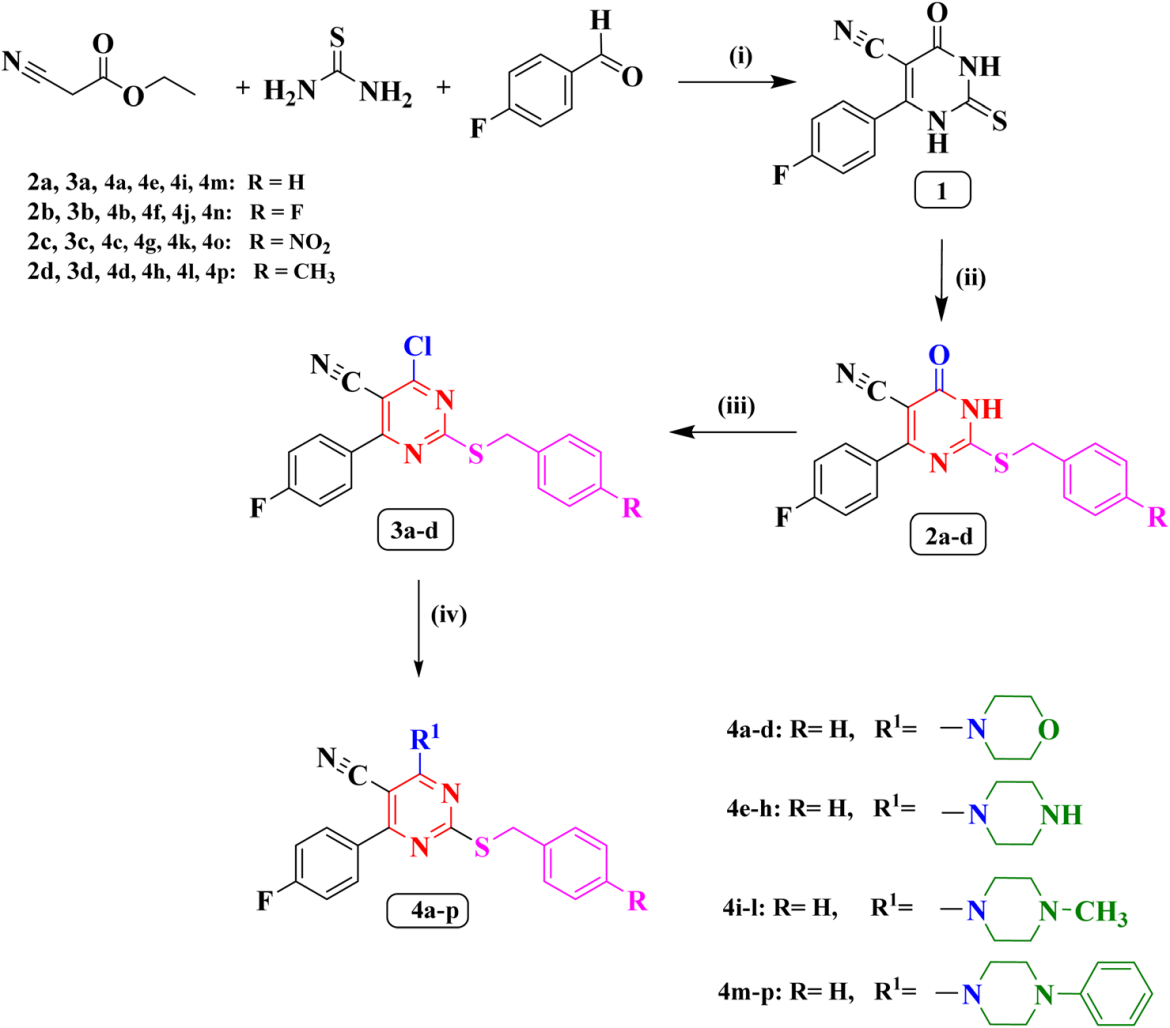
(i) anhydrous K_2_CO_3_/reflux/7 h/absolute ethanol. (ii) Appropriate benzyl chloride, anhydrous K_2_CO_3_/DMF/0–5 °C. (iii) POCl_3_/reflux/5 h. (iv) Appropriate secondary amine/dry benzene/reflux/4 h.

The synthetic methodology for the preparation of the target compounds 2a–d, 3a–d and 4a–p is depicted in Scheme 1. The structure of the newly synthesized compounds was confirmed by elemental analysis and spectral data (IR, ^1^H NMR, ^13^C NMR, ^19^F NMR and MS). The 6-(4-fluorophenyl)-4-oxo-2-thioxo-1,2,3,4-tetrahydropyrimidine-5-carbonitrile (1) is the precursor of the newly synthesized compound in this study which was synthesized by a single pot reaction of 4- fluorobenzaldehyde, ethyl cyanoacetate and thiourea in the presence of anhydrous potassium carbonate (K_2_CO_3_) as previously reported Biginelli reaction.^27,28^ The corresponding *S*-alkylated derivatives 2a–d were obtained through the reaction of compound 1 with the appropriate benzyl chloride derivatives in dimethylformamide (DMF) at −5 °C.^27,29^ The ^1^H NMR spectra of new compounds 2b–d indicated the presence of a new singlet peak around *δ* 4.28 and 4.54 ppm belonging to methylene group S-CH_2._ Additionally, the ^1^H NMR spectrum of compound 2d showed a singlet signal of three protons at *δ* 2.26 ppm corresponding to the methyl group. The ^19^F NMR spectra of compounds 2c, and 2d displayed single peak corresponding to the fluorine atom at *δ* −113.37 ppm, and *δ* −109.93 ppm, respectively.

The chloropyrimidine derivatives 3a–d were prepared *via* nucleophilic substitution reaction in presence of phosphorus oxychloride using adopting previous reported procedure.^29,30^ The IR spectra of the newly prepared compounds 3a–d revealed the lack of NH band, which confirmed the success of chlorination. However, in the course of this study a good yield of the pure target compounds 4a–p was obtained when 4-chloro-6-(4-fluorophenyl)-2-((4-substitutedbenzyl)thio)pyrimidine-5-carbonitriles (3a–d) was allowed to react with the double molar concentration of various secondary amines in dry benzene.^31^ The ^19^F NMR spectrum of compound 3b showed two peaks at *δ* −106.96 ppm, and *δ* −114.86 ppm corresponding to the two fluorine atoms. On the other hand, the ^19^F NMR spectrum of compound 3c showed single peak at *δ* −106.90 ppm corresponding to one fluorine atom.

The ^1^H NMR spectra of compounds 4a–d revealed the presence of two triplet signals around *δ* 3.68–3.70 ppm and *δ* 3.89–3.92 ppm attributed to four CH_2_ of the morpholine ring. Furthermore, The IR spectra of (piperazin-1-yl) pyrimidine derivatives 4e–h showed the appearance of one absorption band at 3446 cm^−1^ for N–H of piperazine. Also, their ^1^H NMR spectra indicated two characteristic signals around *δ* 2.79–3.39 ppm and *δ* 3.81–4.15 ppm disclosing the presence of piperazine protons. The ^19^F NMR spectrum of compounds 4a, 4d, 4e, 4g, and 4h displayed single peak from *δ* −108.71 ppm to *δ* −109.04 ppm. On the contrary, ^19^F NMR spectrum of compound 4f showed two peaks at *δ* −109.03 ppm, and *δ* −115.47 ppm corresponding to two the fluorine atoms.

On the other hand, the ^1^H NMR spectra of compounds 4i–l revealed the appearance of new a singlet signal of three protons around *δ* 2.21–2.25 ppm pointing to methyl group attached to piperazine ring. Finally, the ^1^H NMR spectra of compounds (4-phenylpiperazin-1-yl) pyrimidine 4m–p disclosed the appearance of five aromatic protons in the aromatic range corresponding to phenyl group attached to piperazine ring. The ^19^F NMR spectra of compounds 4i, 4k, 4m, 4o, and 4p displayed single peak from *δ* −108.84 ppm to *δ* −108.96 ppm corresponding to the single fluorine atom in these compounds. On the other hand, the ^19^F NMR spectra of compounds 4j, and 4n showed two peaks at *δ* (−108.93, and −115.46 ppm), and *δ* (−108.8, and −115.41 ppm), respectively, corresponding to the two fluorine atoms.

### Biological evaluation

#### *In vitro* cytotoxicity

The National Cancer Institute (NCI), Bethesda, Maryland, United States, selected all of the novel synthesized compounds (2b–d, 3a–d and 4a–p) for *in vitro* antitumor screening at a single concentration of 10 μM toward the NCI60 cell lines panel covering leukaemia, non-small cell lungs, colon, central nervous system (CNS), melanoma, ovarian, renal, prostate, and breast cancer cells.^32–35^ The data were reported as a mean graph of the percent growth of the treated cell lines and expressed as percentage growth inhibition (GI%) in Table 1 and ESI 1.

**Table 1:** *In vitro* anticancer screening activity of the most potent compounds (2b, 4b, 4c, 4d, 4e, 4f, 4i, and 4j) against some of NCI cell line at 10 μM concentration (one-dose study)

Compound	GI%^a^									
	K-652	SR	HOP-62	NCI-H226	SF-539	SNB-75	OVCAR-4	786–0	RXF393	HS578T
2b	—b	—	—	—	—	—	73.14	84.82	—	—
4b	—	—	75.65	—	—	82.51	—	75.34	—	74.34
4c	—	—	—	76.22	73.77	92.38	—	—	90.21	84.42
4d	—	—	—	—	—	88.04	—	—	—	—
4e	Lethal to all NCI60 cell lines									
4f	Lethal to almost all NCI60 cell lines									
4i	73.15	70.98	—	—	—	—	—	—	—	—
4j	—	—	—	—	—	74.91	—	—	80.61	—

a GI value is the growth inhibitory%.

b GI value > 70%.

From the obtained results, the newly synthesized compounds were shown to have cytotoxic activities ranging from moderate to high. In terms of the focus on individual cell lines, compound 2b (*P*-fluorobenzyl derivative) displayed a remarkable activity toward ovarian cancer OVCAR-4 and renal cancer 786–0 With GI values 73.14% and 84.82% respectively. Compounds 2c and 2d exhibited selective activity against the leukemia HL-60(TB) with GI values of 49.95% and 43.38%, respectively. The Chlorine atom boosts the compound lipophilicity, which improves its uptake by cancer cells and hence its activity.^27,36^ As a result, compounds 3a–3d exhibited better activity profile as compared to compounds 2b–d on the most cell lines. For example, the growth inhibition% on breast cancer T-47D jumped from 13.50 (2d) to 68.67 (3d).

Addition of morpholine to compounds 3a–d results in compounds 4a–d with moderate to high cytotoxic activities across a variety of cell lines. Compound 4b displayed significant anti-tumour activity against non-small cell lung cancer (HOP-62), CNS cancer (SNB-75), renal cancer (RXF 393) and breast cancer (HS578T) with GI value of (75.65%, 82.51%, 75.34% and 74.34%, respectively). Broad cytotoxic activity was also demonstrated by compound 4c (*p*-nitrobenzyl derivative). In non-small cell lung cancer (NCI-H226) and CNS cancers (SF-539), it exerted remarkable activity with GI value of 76.22% and 73.77%, respectively. Additionally, it demonstrated superior cytotoxic activity on CNS cancers (SNB-75), renal cancer (RXF 393) and breast cancer (HS 578T) with GI values of and 92.38%, 90.21% and 84.42%, respectively than other derivatives in its series. Replacing nitro group in 4c by methyl group in 4d reduced the activity against all the cell lines. Knowing that, this compound displayed a great cytotoxic effect on CNS cancer (SNB-75) cell line (GI% = 88.04).

Shifting the morpholine ring in compounds 4a & 4b by piperazine ring at position 6 yielded the two most potent molecules in this study: 4e and 4f which were selected for further perspective screening at five-dose assay [0.01, 0.1, 1, 10 and 100 μM]^32–35^ against NCI60 different cancer cell lines, the results are displayed in Table 2 and ESI 1.†

**Table 2:** *In vitro* growth inhibition percent (GI%) for the NCI60 cancer cell lines upon treatment with 10 μM of compounds (2b–d, 3a–d and 4a–d)^a^

	2b	2c	2d	3a	3b	3c	3d	4a	4b	4c	4d
**Leukaemia**											
CCRF-CEM	—	15.45	—	—	13.30	32.30	29.71	—	12.89	22.80	—
HL-60(TB)	20.88	49.95	43.38	44.03	49.83	57.98	51.77	31.31	15.01	17.28	—
K-652	—	31.45	—	11.78	30.20	44.30	42.83	16.46	16.20	23.12	10.67
MOLT-4	—	24.34	11.64	—	19.35	38.07	29.08	—	—	26.34	—
RPMI-8226	—	14.16	—	—	—	41.51	25.03	—	—	19.73	—
SR	—	—	—	—	—	—	—	—	—	—	—

**Non-small cell lung cancer**											
A549/ATCC	15.77	—	10.16	16.44	—	33.50	16.81	—	31.03	36.61	24.90
EKVX	—	12.04	—	—	10.91	20.38	20.92	—	39.92	60.74	13.10
HOP-62	16.84	—	—	12.77	—	—	—	—	75.65	65.34	36.77
HOP-92	19.25	33.01	—	14.74	25.21	45.40	34.25	15.08	65.67	63.28	34.62
NCI-H226	24.21	22.53	—	18.61	28.68	46.54	37.93	—	51.64	76.22	21.35
NCI-H23	12.73	—	—	—	—	12.20	—	—	28.19	25.17	—
NCI-H322M	7.12	—	—	—	—	13.28	—	—	21.55	17.30	—
NCI-H460	13.27	—	—	—	—	13.48	—	—	26.13	22.56	—
NCI-H522	25.94	30.52	12.91	16.01	25.38	37.48	29.73	16.81	45.02	58.72	44.57

**Colon cancer**											
COLO 205	—	—	—	—	—	22.66	—	—	—	—	—
HCC-2998	—	—	—	—	—	12.17	—	—	12.64	—	—
HCT-116	33.39	22.23	—	22.79	18.51	40.70	25.73	10.37	33.96	35.64	19.81
HCT-15	—	13.42	15.77	—	13.00	45.64	24.97	—	20.05	27.19	—
HT29	—	37.56	10.72	—	13.44	61.63	51.51	—	—	13.41	—
KM12	—	—	—	—	—	27.77	19.05	—	12.78	15.07	—
SW-620	—	10.78	—	—	—	37.21	11.21	—	14.48	19.46	—

**CNS cancer**											
SF-268	24.67	—	—	21.56	—	18.20	12.95	—	56.58	50.66	22.32
SF-295	25.73	10.37	—	27.87	10.01	29.81	11.51	—	63.10	58.52	32.25
SF-539	45.33	—	—	24.06	—	28.21	15.58	16.91	50.95	73.77	32.27
SNB-19	18.35	—	—	16.70	—	10.67	—	—	56.32	53.96	44.48
SNB-75	23.60	—	—	—	—	—	—	—	82.51	92.38	88.04
U251	33.40	13.78	—	39.15	—	31.17	15.75	—	52.85	44.68	13.13

**Melanoma**											
LOX IMVI	10.55	14.24	—	—	—	14.22	—	—	23.57	15.29	11.10
MALME-3M	—	—	—	—	—	—	—	—	41.38	57.24	26.17
M14	—	—	—	—	—	12.41	—	—	—	12.25	—
MDA-MB-435	—	—	—	—	—	—	—	—	—	16.03	—
SK-MEL-2	—	—	—	—	—	—	—	—	21.29	12.63	—
SK-MEL-28	—	—	—	—	—	—	—	—	14.16	17.90	—
SK-MEL-5	—	11.85	—	—	13.82	22.36	15.98	—	25.68	38.14	10.99
UACC-257	—	—	—	—	—	24.66	—	—	22.89	45.85	17.42
UACC-62	11.33	11.42	—	—	13.95	22.80	17.58	13.24	30.66	37.29	22.83

**Ovarian cancer**											
IGROV1	—	—	—	—	—	—	—	—	—	19.45	—
OVCAR-3	—	—	—	—	—	13.71	—	—	24.80	30.28	—
OVCAR-4	73.14	—	—	49.74	—	18.42	14.97	—	39.53	42.76	20.15
OVCAR-5	—	—	—	—	—	—	—	—	16.89	—	—
OVCAR-8	48.04	—	—	27.14	10.81	16.16	—	21.20	45.16	46.89	23.86
NCI/ADR-RES	37.17	12.03	—	20.64	13.45	27.59	14.93	10.32	36.47	58.37	—
SK-OV-3	26.25	—	—	—	—	11.73	—	—	60.37	55.46	11.69

**Renal cancer**											
786–0	84.82	—	—	38.26	—	21.29	12.30	—	75.34	69.78	55.92
A498	—	—	—	—	—	—	—	—	28.08	35.57	—
ACHN	26.83	—	—	45.51	—	14.60	—	—	53.35	48.98	37.30
CAKI-1	24.10	10.99	—	21.66	—	32.89	16.99	—	69.32	64.15	44.00
RXF 393	28.91	—	—	19.19	10.29	29.76	12.49	—	58.43	90.21	48.96
SN12C	17.08	14.07	—	—	19.06	29.81	27.59	—	27.12	25.44	—
TK-10	17.90	—	—	—	—	—	—	—	22.42	15.83	—
UO-31	26.71	11.53	—	14.25	12.89	32.10	17.01	—	13.97	49.11	10.06

**Prostate cancer**											
PC-3	—	18.97	—	—	—	57.37	35.47	—	28.37	40.85	—
DU-145	—	—	—	—	—	24.93	18.41	—	22.16	26.65	—

**Breast cancer**											
MCF7	—	—	10.82	—	17.40	27.06	12.55	—	36.26	41.49	11.76
MDA-MB-231/ATCC	39.26	—	—	21.72	—	—	—	10.65	49.52	52.93	31.92
HS 578T	24.34	—	—	16.76	—	13.31	—	19.87	74.34	84.42	48.85
BT-549	—	—	15.75	11.73	—	43.62	18.08	19.15	35.72	41.46	11.94
T-47D	25.99	23.03	13.50	10.38	33.18	68.67	46.84	15.89	26.75	44.55	12.46
MDA-MB-468	14.26	—	—	—	—	19.68	—	—	37.89	49.44	—
Mean	16.01	—	—	—	—	23.91	12.62	—	34.42	39.54	14.01

a GI% < 10.

Compound 4e was the most potent one, it showed lethal effect to all cancer subpanels. Also, compound 4f was lethal to most of cell lines and displayed excellent antiproliferative activity against leukaemia (CCRF-CEM, K-652, MOLT-4), non-small cell lung cancer (EKVX) and ovarian cancer (OVCAR-5) (GI% = 95.51, 99.28, 92.21, 98.71 and 99.16, respectively). Moreover, it showed highest cytotoxicity on CNS cancer (SNB-75), renal cancer (UO-31) and prostate cancer (PC-3) with GI values 80.66%, 91.74% and 88.74% respectively. On the other hand, changing the morpholine ring in compounds 4c & 4d by piperazine ring afforded inactive compounds 4g & 4h.

The incorporation of 4-methylpiperazine ring in place of piperazine ring in compounds 4e–h reduced the activity in compounds 4i & 4j in comparison with 4e & 4f, in contrast it improved the activity in compounds 4k & 4l compared to 4g & 4h. Compound 4i exerted remarkable activity against various of cell lines. For instance, it demonstrated activity on leukaemia (K-652, SR), (GI% = 73.15 and 70.98, respectively). Also, compound 4j exhibited significant activity against CNS cancer (SNB-75) and renal Cancer (RXF 393) with GI values of (74.91% and 80.61%, respectively). Compounds 4k & 4l showed moderate activity against numerous cell lines. Replacing methyl Piperazine ring by phenyl piperazine ring resulted in 4m–p which were inactive compounds compared to other compounds in this study.

Upon closer inspection of the data reported in Table 3 & ESI 1,† it became clear that the most potent members in our series were compounds 4e & 4f. the preliminary one-dose NCI anticancer screening was passed by compounds 4e & 4f. these two potent cytotoxic candidates were involved into further investigation against NCI60 cancer cell lines in five-dose screening (0.01–100 μM). Results of five-dose antitumour screening were reported as three descriptors (1) the median concentration of a tested compound that would result in a 50% reduction in cell growth is known as median growth inhibition (GI_50_). (2) The molar concentration of the compound responsible for total growth inhibition, also known as total growth inhibition (TGI). (3) The molar concentration that causes 50% of all cells to die is referred to as the LC_50_ (median lethal concentration) Tables 4 and 5. Thus, mean graph mid-points (MG-MID) for compounds 4e and 4f against each of the different subpanels and full panel cell lines were calculated to determine their GI_50_, TGI, and LC_50_ values Table 4 and 5.

**Table 3:** *In vitro* growth inhibition percent (GI%) for the NCI60 cancer cell lines upon treatment with 10 μM of compounds (4a–p)^a^^,^^b^

	4e	4f	4g	4h	4i	4j	4k	4l	4m	4n	4o	4p
**Leukaemia**												
CCRF-CEM	L	95.51	—	—	37.86	—	17.51	15.17	11.06	16.65	—	—
HL-60(TB)	L	L	—	—	36.50	—	—	12.21	—	—	—	—
K-652	L	99.28	—	—	73.15	18.51	23.28	28.64	10.59	12.49	—	17.97
MOLT-4	L	92.21	—	—	49.82	—	17.62	10.54	—	12.40	—	—
RPMI-8226	L	L	—	—	56.41	14.97	17.84	15.46	14.20	22.34	15.35	18.39
SR	L	L	—	—	70.98	—	—	—	—			

**Non-small cell lung cancer**												
A549/ATCC	L	L	—	—	38.33	27.72	18.72	13.07	—	—	—	11.63
EKVX	L	98.71	—	—	20.21	18.41	22.37	12.54	—	18.68	13.93	20.31
HOP-62	L	L	—	—	10.31	64.39	26.33	12.81	—	—	—	—
HOP-92	L	L	—	—	22.73	66.16	47.05	39.32	—	—	—	—
NCI-H226	L	23.08	—	—	—	37.79	—	—	—	—	—	—
NCI-H23	L	L	—	—	—	22.20	19.67	—	—	—	—	—
NCI-H322M	L	63.95	—	—	16	24.65	26.32	18.01	—	—	—	—
NCI-H460	L	L	—	—	35.06	—	—	—	—	—	—	—
NCI-H522	L	L	—	—	31.71	23.62	21.32	33.39	—	10.27	—	13.84

**Colon cancer**												
COLO 205	L	L	—	—	13.56	—	—	—	—	—	—	12.23
HCC-2998	L	L	—	—	—	—	—	—	—	—	—	—
HCT-116	L	L	—	—	45.78	25.31	18.38	13.10	—	11.81	—	15.11
HCT-15	L	L	—	—	46.82	—	15.69	10.32	10.81	—	—	13.95
HT29	L	L	—	—	67.00	—	—	10.57	—	—	—	—
KM12	L	L	—	—	19.06	—	—	—	—	—	—	—
SW-620	L	L	—	—	10.04	—	—	—	—	—	—	—

**CNS cancer**												
SF-268	L	58.46	—	—	26.82	15.77	—	—	—	14.13	—	15.26
SF-295	L	L	—	—	12.26	56.76	29.29	17.58	—	—	—	—
SF-539	L	L	—	—	14.45	54.78	40.98	31.78	—	—	—	—
SNB-19	L	50.36	—	—	10.01	40.28	29.58	14.28	—	—	—	—
SNB-75	L	80.66	15.86	19.47	41.03	74.91	25.71	35.62	—	15.56	12.45	14.84
U251	L	L	—	—	—	26.16	—	—	—	—	—	—

**Melanoma**												
LOX IMVI	L	L	—	—	25.45	22.86	20.90	12.85	—	—	—	—
MALME-3M	L	L	—	—	—	39.52	27.18	11.70	—	—	—	—
M14	L	L	—	—	18.15	10.37	—	—	—	10.14	—	11.03
MDA-MB-435	L	L	—	—	12.84	—	—	—	—	—	—	10.29
SK-MEL-2	L	L	—	—	—	—	—	—	—	10.64	—	15.10
SK-MEL-28	L	L	—	—	—	16.21	14.43	—	—	—	—	—
SK-MEL-5	L	L	—	—	33.65	—	16.39	—	—	12.43	—	13.68
UACC-257	L	L	—	—	27.77	—	—	—	—	12.89	—	14.05
UACC-62	L	L	—	—	19.60	22.49	19.78	12.96	16.18	25.83	18.11	29.69

**Ovarian cancer**												
IGROV1	L	L	—	—	—	16.88	—	—	—	—	—	—
OVCAR-3	L	L	—	—	14.43	16.15	—	—	—	—	—	—
OVCAR-4	L	L	—	—	22.50	24.09	—	—	—	10.40	12.28	10.63
OVCAR-5	L	99.16	—	—	—	17.45	16.79	—	—	—	—	—
OVCAR-8	L	L	—	—	14.07	32.05	11.69	11.92	—	—	—	—
NCI/ADR-RES	L	L	—	—	—	42.93	25.06	12.67	—	—	—	—
SK-OV-3	L	43.30	—	—	—	55.50	20.93	—	—	—	—	—

**Renal cancer**												
786–0	L	L	—	—	29.30	67.06	26.44	44.23	—	—	—	—
A498	L	—	—	—	12.50	—	—	—	—	—	—	—
ACHN	L	L	—	—	14.77	39.09	31.49	29.95	—	—	—	—
CAKI-1	L	L	10.98	11.67	37.93	57.49	34.76	36.94	10.65	16.34	13.69	16.64
RXF 393	L	L	15.23	—	49.72	80.61	68.50	42.40	—	—	—	—
SN12C	L	L	—	—	12.61	20.47	15.82	11.94	—	—	—	—
TK-10	L	L	—	—	—	14.97	—	—	—	—	—	—
UO-31	L	91.74	11.87	12.10	36.00	30.15	40.05	17.62	—	—	—	—

**Prostate cancer**												
PC-3	L	88.74	—	—	31.85	32.32	25.95	24.21	—	25.21	—	25.11
DU-145	L	L	—	—	—	—	—	—	—	—	—	—

**Breast cancer**												
MCF7	L	L	—	—	30.78	14.77	17.63	12.79	16.08	24.84	14.77	21.28
MDA-MB-231/ATCC	L	L	—	—	15.90	45.82	35.12	28.90	—			
HS 578T	L	L	—	10.10	32.04	60.43	42.57	31.10	—	—	—	—
BT-549	L	17.34	—	—	—	32.05	46.49	10.77	—	—	—	—
T-47D	L	L	—	—	42.49	18.27	28.64	18.05	15.11	24.33	—	12.29
MDA-MB-468	L	L	—	—	29.17	23.57	12.43	—	—	—	10.88	13.55
Mean	L	L	—	—	24.31	25.08	17.52	11.17	—	—	—	—

a GI% > 100.

b GI% < 10.

The data confirmed that compounds 4e & 4f evoked potent anticancer activity against the all-tested cell lines with effective growth inhibition full panel GI_50_ (MG-MID) values of 10.66, and 13.46 μM, respectively and cytostatic activity full panel TGI (MG-MID) values of 23.60, and 26.45 μM, separately. Furthermore, the LC_50_ values (cytotoxicity) ranged (from 5 to 69 μM) and (from 6.34 to >100 μM), respectively, to examined cell lines.

In respect to activity against each single cell line in Table 4. Compound 4e demonstrated a distinctive activity pattern against leukemia cell lines and colon cancer cell lines with GI_50_ range of 1.77–17.10 μM. Particularly, it exhibited a remarkable high activity toward leukaemia K-562 (GI_50_ = 2.21, TGI = 5.42 and LC_50_ = 18.70 μM), leukaemia SR (GI_50_ = 1.92, TGI = 4.61 and LC_50_ = 13.80 μM), colon cancer colo 205 (GI_50_ = 1.66, TGI = 3.17 and LC_50_ = 6.04 μM), colon cancer HCC-2998 (GI_50_ = 1.76, TGI = 3.22 and LC_50_ = 5.89 μM) and melanoma LOX IMVI (GI_50_ = 1.71, TGI = 3.33 and LC_50_ = 6.48 μM). Also, compound 4f displayed a remarkable high activity against leukaemia SR with (GI_50_ = 2.18, TGI = 5.07 and LC_50_ = 17.50 μM), and colon cancer × 205 (GI_50_ = 1.83, TGI = 3.40 and LC_50_ = 6.34 μM), colon cancer HCC-2998 (GI_50_ = 2.53, TGI = 3.22 and LC_50_ = 6.27 μM), melanoma LOX IMVI (GI_50_ = 2.28, TGI = 5.69 and LC_50_ = 22.70 μM), melanoma SK-MEL-28 (GI_50_ = 1.92, TGI = 4.04 and LC_50_ = 8.47 μM) (Table 5).

**Table 4:** Median growth inhibitory (GI_50,_ μM), total growth inhibitory (TGI, μM) and median lethal (LC_50_) concentrations of compound 4e

Subpanel tumor cell lines	Activity			Subpanel tumor cell lines	Activity		
	GI_50_	TGI	LC_50_		GI_50_	TGI	LC_50_
**Leukemia**				**Melanoma**			
CCRF-CEM	13.90	31.00	69.00	MALME-3M	14.10	28.20	56.30
HL-60(TB)	4.50	17.30	46.50	M14	12.00	24.80	51.40
K-562	2.21	5.42	18.70	MDA-MB-435	16.90	31.50	58.80
MOLT-4	7.07	21.20	52.70	SK-MEL-28	16.30	30.30	56.30
RPMI-8226	3.99	17.50	65.40	SK-MEL-5	15.70	29.20	54.50
SR	1.92	4.61	13.80	UACC-257	13.10	26.20	52.60
**Non-small cell lung cancer**				UACC-62	14.40	28.20	55.00
A549/ATCC	8.10	21.50	50.20	**Ovarian cancer**			
EKVX	12.00	24.80	51.00	IGROV1	14.70	28.90	56.70
HOP-62	13.20	28.10	59.90	OVCAR-3	14.30	29.10	59.10
HOP-92	4.20	18.10	48.40	OVCAR-4	15.70	34.20	74.70
NCI-H226	13.80	33.40	81.30	OVCAR-5	14.10	27.60	54.20
NCI-H23	15.10	29.30	56.50	OVCAR-8	6.21	21.80	59.10
NCI-H322M	14.30	27.90	54.20	NCI/ADR-RES	11.70	27.40	64.40
NCI-H460	9.20	22.90	53.90	SK-OV-3	16.00	29.50	54.60
NCI-H522	11.30	25.10	55.80	**Renal cancer**			
**Colon cancer**				786–0	11.00	25.40	58.90
COLO 205	1.66	3.17	6.04	A498	13.10	27.30	56.90
HCC-2998	1.76	3.22	5.89	ACHN	14.00	27.50	54.00
HCT-116	3.51	11.60	38.10	CAKI-1	16.00	29.70	55.10
HCT-15	5.05	17.50	43.80	RXF 393	3.14	14.80	44.20
HT29	3.30	10.50	36.60	SN12C	13.80	27.20	53.80
KM12	17.10	32.80	63.10	TK-10	14.50	28.40	55.50
SW-620	3.38	13.10	40.90	**Prostate cancer**			
**CNS cancer**				PC-3	11.80	25.80	56.40
SF-268	12.90	29.10	65.40	DU-145	15.00	29.00	56.00
SF-295	15.80	30.10	57.50	**Breast cancer**			
SF-539	15.70	29.40	54.90	MCF7	3.94	15.30	44.80
SNB-19	14.20	29.30	60.60	HS 578T	14.10	32.00	72.70
U251	4.36	16.10	44.60	BT-549	13.00	26.40	53.60
**Melanoma**				T-47D	10.10	24.80	61.00
LOX IMVI	1.71	3.33	6.48	MDA-MB-468	6.71	21.30	51.60

**Table 5:** Median growth inhibitory (GI_50,_ μM), total growth inhibitory (TGI, μM) and median lethal (LC_50_) concentrations of compound 4f

Subpanel tumor cell lines	Activity			Subpanel tumor cell lines	Activity		
	GI_50_	TGI	LC_50_		GI_50_	TGI	LC_50_
**Leukaemia**				**Melanoma**			
CCRF-CEM	14.90	33.60	76.00	MALME-3M	14.70	28.40	54.80
HL-60(TB)	7.66	24.00	63.80	M14	13.60	26.80	52.90
K-562	3.76	17.80	>100	MDA-MB-435	16.80	31.30	58.40
MOLT-4	10.50	26.00	64.40	SK-MEL-28	16.40	30.20	55.90
RPMI-8226	6.75	24.60	74.40	SK-MEL-5	16.80	30.50	55.40
SR	2.18	5.07	17.50	UACC-257	14.80	28.70	55.80
**Non-small cell lung cancer**				UACC-62	15.90	29.90	56.20
A549/ATCC	11.50	25.00	54.20	**Ovarian cancer**			
EKVX	13.10	26.40	52.90	IGROV1	15.60	29.80	56.80
HOP-62	17.00	33.40	65.80	OVCAR-3	15.50	30.80	61.20
HOP-92	7.64	22.10	53.40	OVCAR-4	16.50	33.60	68.70
NCI-H226	15.90	35.70	80.30	OVCAR-5	14.50	28.00	53.90
NCI-H23	16.00	30.40	57.50	OVCAR-8	13.20	30.50	70.60
NCI-H322M	14.00	27.20	52.80	NCI/ADR-RES	13.70	30.40	67.50
NCI-H460	14.30	29.40	60.40	SK-OV-3	16.00	29.80	55.40
NCI-H522	13.90	29.90	64.30	**Renal cancer**			
**Colon cancer**				786–0	13.80	29.30	62.60
COLO 205	1.83	3.40	6.34	A498	15.30	29.80	58.20
HCC-2998	2.53	6.27	6.27	ACHN	15.00	28.60	54.50
HCT-116	4.15	15.60	42.80	CAKI-1	15.30	29.00	54.60
HCT-15	11.70	24.60	51.80	RXF 393	73.00	21.40	51.50
HT29	4.87	17.30	51.70	SN12C	15.30	29.00	55.20
KM12	18.00	33.60	63.00	TK-10	15.00	29.70	58.80
SW-620	5.97	19.30	48.70	**Prostate cancer**			
**CNS cancer**				PC-3	12.50	27.70	61.30
SF-268	14.10	30.90	67.50	DU-145	16.80	31.30	58.10
SF-295	16.60	30.90	57.50	**Breast cancer**			
SF-539	15.60	29.00	54.10	MCF7	6.43	20.20	50.10
SNB-19	14.90	29.40	58.10	HS 578T	15.60	34.60	77.00
U251	10.30	24.70	59.10	BT-549	10.70	23.00	49.40
**Melanoma**				T-47D	13.80	30.40	67.20
LOX IMVI	2.28	5.69	22.70	MDA-MB-468	12.40	26.20	55.30

Regarding subpanel selectivity toward certain cancer type, the method of determining a compound's selectivity index is to divide the full panel MG-MID (μM) value by each single subpanel. Values above six indicate high selectivity against the subpanel; moderate selectivity is denoted by values between three and six. Conversely, compounds are termed nonselective if their selectivity is less than three.^30,37^ In this study, considering GI_50_ and TGI values compound 4e was regarded as broad-spectrum, non-selective anticancer, with selectivity index ranges from (0.79 to 2.09) and from (0.83 to 2.01) respectively (Table 6). Similar results were reported with Compound 4f, which demonstrated non-selective anticancer efficacy across the nine tumor panels tested at both GI_50_ and TGI levels, with selectivity index ranges from (0.57 to 1.92) and from (0.86 to 1.54), respectively (Table 7).

**Table 6:** Median growth inhibitory concentrations (GI_50_, μM) and median total growth inhibitory concentrations (TGI, μM) of *in vitro* subpanel tumor cell lines for compound 4e

Subpanel tumor cell line^c^	Median growth inhibitory concentrations (GI_50_, μM)		Median total inhibitory concentrations (TGI, μM)	
	MG-MID^a^ (G_I50_)	Selectivity index	MG-MID^b^ (TGI)	Selectivity index
I	5.59	1.90	16.17	2.01
II	11.24	0.95	25.67	0.92
III	5.10	2.09	13.12	1.79
IV	12.59	0.85	26.80	0.88
V	13.02	0.82	25.21	0.93
VI	13.24	0.81	28.35	0.83
VII	12.22	0.87	25.75	0.92
VIII	13.40	0.79	27.40	0.86
IX	9.57	1.11	23.96	0.98
Full panel MG-MID	10.66^d^	—	23.60^e^	—

a Median values calculated according to the data obtained from NCI^'^s *in vitro* disease-oriented human tumor cell screen.

b Median values calculated according to the data obtained from NCI^'^s *in vitro* disease-oriented human tumor cell screen.

c I, Leukemia; II, non-small cell lung cancer; III, colon cancer; IV, CNS cancer; V, melanoma; VI, ovarian cancer; VII, renal cancer; VIII, prostate cancer; IX, breast cancer.

d GI_50_ (μM) full panel mean-graph mid-point (MG-MID) = the average sensitivity of all cell lines towards the test agent.

e TGI (μM) full panel mean-graph mid-point (MG-MID) = the average sensitivity of all cell lines towards the test agent.

**Table 7:** Median growth inhibitory concentrations (GI_50_, μM) and Median total growth inhibitory concentrations (TGI, μM) of *in vitro* subpanel tumor cell lines for compound 4f

Subpanel tumor cell line^c^	Median growth inhibitory concentrations (GI_50_, μM)		Median total inhibitory concentrations (TGI, μM)	
	MG-MID^a^ (GI_50_)	Selectivity index	MG-MID^b^ (TGI, μM)	Selectivity index
I	7.63	1.76	21.84	1.21
II	13.70	0.98	28.83	0.91
III	7.00	1.92	17.15	1.54
IV	14.30	0.94	28.98	0.91
V	13.91	0.96	26.43	1.00
VI	15.00	0.89	30.41	0.86
VII	23.24	0.57	28.11	0.94
VIII	14.65	0.91	29.50	0.89
IX	11.78	1.14	26.88	0.98
Full panel MG-MID	13.46^d^	—	26.45^e^	—

a Median values calculated according to the data obtained from NCI^'^s *in vitro* disease-oriented human tumor cell screen.

b Median values calculated according to the data obtained from NCI^'^s *in vitro* disease-oriented human tumor cell screen.

c I, Leukemia; II, non-small cell lung cancer; III, colon cancer; IV, CNS cancer; V, melanoma; VI, ovarian cancer; VII, renal cancer; VIII, prostate cancer; IX, breast cancer.

d GI_50_ (μM) full panel mean-graph mid-point (MG-MID) = the average sensitivity of all cell lines towards the test agent.

e TGI (μM) full panel mean-graph mid-point (MG-MID) = the average sensitivity of all cell lines towards the test agent.

Additionally, these compounds' cytotoxicity was tested on non-tumorigenic cell line FHC (epithelial colon cell line), (ATCC® CRL-1831™) to examine their safety on normal cells, taking Staurosporine as a reference. The IC_50_ values and selectivity indexes were calculated in Table 8.^38,39^ Compound 4e displayed moderate safety pattern with SI = 17.01, while compounds 4f exhibited high safety threshold on normal epithelial colon cells with SI = 25.32. Further perspectives of their mechanisms of action were carried.

**Table 8:** IC_50_ values of compounds 4e, and 4f against Colo 205 cell line and the normal epithelial colon cell line and their selectivity index

Compounds	IC_50_ (μM) Colo 205	IC_50_ (μM) FHC	Selectivity index (S.I.) FHC/Colo 205
4e	1.66	28.25	17.01
4f	1.83	46.34	25.32
Staurosporine	—	23.00	—

#### *In vitro* EGFR ^WT^ inhibition

The potential inhibitory action of compounds 4e and 4f was assessed against EGFR^WT^ to take a closer look at the mechanism underlying the potent anticancer effect of 4e and 4f. Comparing the two tested compounds (4e), displayed more distinct EGFR^WT^ inhibitory activity (IC_50=_0.096 μM) than (4f) (IC_50=_0.235 μM) compared to the reference drug erlotinib against EGFR^WT^ (IC_50_ = 0.037 μM), as shown in Table 9.

**Table 9:** *In vitro* inhibitory activity of compounds 4e and 4f against EGFR^WT^

Compound	EGFR IC_50_ (μM)^a^
4e	0.096 ± 0.004
4f	0.235 ± 0.01
Erlotinib	0.037 ± 0.002

a The results given are means ± SD of three experiments.

#### *In vitro* COX-2 inhibition

The *in vitro* inhibitory activity of compounds 4e, and 4f was evaluated against COX-2, using indomethacin and celecoxib as standards in order to determine the involvement of COX-2 in cancer. The results are represented in Table 10. The target compounds exerted inhibitory activity against COX-2 with (IC_50_ = 1.281 and 5.264 μM, respectively) compared to indomethacin and celecoxib with (IC_50_ = 0.63 and 2.59 μM, respectively). Besides to their inhibitory activity compounds 4e and 4f exhibited good selectivity toward COX-2 over COX-1, with a selectivity index (SI) of 8.55 and 6.01, respectively. indomethacin and celecoxib, the standard drugs, were found to have SI of 0.23 and 8.203. The outcome shown that substances with greater selectivity for COX-2 than COX-1 also have a wider range of effectiveness against different cancer cell lines.

**Table 10:** Cyclooxygenase (COX)-II inhibitory activity of compounds 4e & 4f

Compound	COX1/2 IC_50_ (μM)^a^		SI
	COX1	COX2	
4e	10.96 ± 0.31	1.281 ± 0.062	8.55
4f	31.62 ± 0.89	5.264 ± 0.255	6.01
Indomethacin	0.145 ± 0.004	0.63 ± 0.031	0.23
Celecoxib	21.27 ± 0.604	2.59 ± 0.126	8.203

a The values are the means ± SD of three experiments.

### Cell cycle analysis

Inhibition of the EGFR^WT^/COX-2 pathway has been linked to alterations in cell survival and proliferation, as reported.^22,40^ Therefore, cell cycle analysis was carried out using flowcytometric analysis to determine the impact of the dual inhibition of EGFR^WT^/COX-2 by compounds 4e and 4f on cell cycle progression, as reported.^41,42^ The Colo 205 cells were treated with compounds 4e & 4f on their IC_50_ and their impacts on the different phases of cell growth were displayed in Table 11 and graphically depicted in Fig. 2. Compounds 4e and 4f showed the disturbance in cell cycle was done with significant increase in the percentage of cells at G0–G1 phase, about (1.2–1.1) fold more than in the control. These results indicated that compounds 4e & 4f had an antiproliferative impact by inducing apoptosis and increasing cell cycle arrest in the G0–G1 phase.

**Table 11:** Effect of compounds 4e, and 4f on cell cycle analysis of colo 205 cell lines

Compound	DNA content			
	% G0–G1	% S	% G2/M	Comment
4e	61.31	35.82	2.87	Cell growth arrest@ G1
4f	55.01	39.6	5.39	Cell growth arrest@ G1
Control	49.02	41.88	9.1	—

### Annexin V-FITC apoptosis assay

**Fig. 3:**
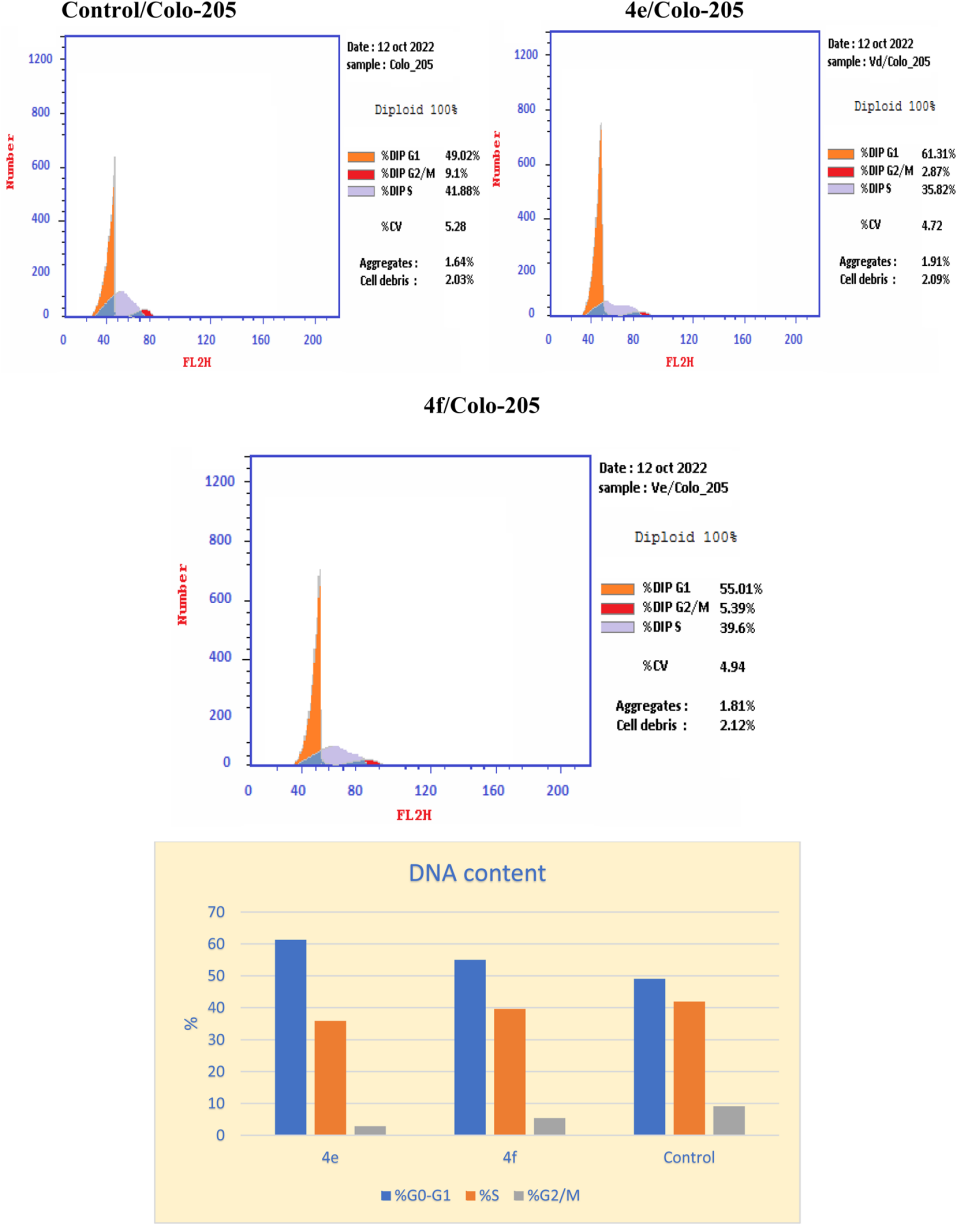
The effect of the new cytotoxic compound (4e and 4f) on the cell cycle of Colo 205 cell line.

The Annexin V-FITC/propidium iodide dual staining assay was used to assess the effect of compound (4e and 4f) on inducing apoptosis in Colo-205 cells. This assay allows for the identification of early stages of apoptosis before the loss of cell membrane integrity, allowing measurements of apoptotic death kinetics in relation to the cell cycle. The present findings showed that after treatment with compound (4e and 4f) on their IC_50_, the proportion of total apoptotic cells in the Colo-205 cell line jumped to 51.07 (4e) and to 43.12 (4f) in comparison to control cells (1.73), indicating a high pro-apoptotic activity (Table 12 and Fig. 3). This reveals that planned apoptosis, not nonspecific necrosis, is the cause of the cytotoxic activity of the target compounds.

**Table 12:** Distribution of apoptotic cells in the Colo-205 cell line after treatment with (4e) and (4f), as seen in the annexin V-FITC assay

Code	Apoptosis			Necrosis
	Total	Early	Late	
4e	51.07	19.91	25.22	5.94
4f	43.12	26.15	12.71	4.26
Control	1.73	0.44	0.15	1.14

### Detection of caspase 3 concentration in colo 205 cells

**Fig. 4:**
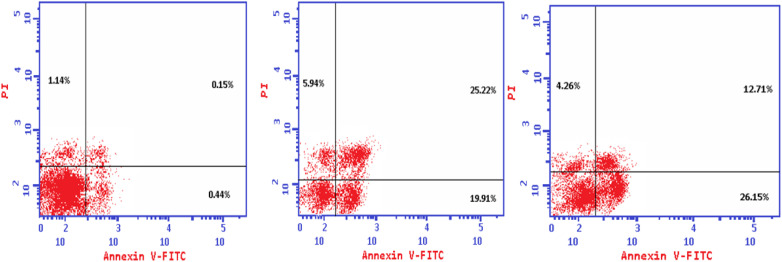
Apoptosis in Colo 205 cell line induced by compounds 4e, and 4f comparable to control.

Among the death proteases, the caspases family of cysteine-dependent aspartate-directed proteases is significant. There are multiple levels where caspase activation and activity are regulated.^43^ Caspases are activated during apoptosis, which eventually results in cell death by deactivating a number of signals.^44^ One of the crucial survival signals, EGFR, may be the target of caspase activation to ensure the completion of apoptosis.^45^ Additionally, it has been reported that activating caspase-3 through COX-2 inhibition enhances apoptosis.^40^ Therefore, the impact of the novel compounds on the level of caspase 3 was examined. Colo 205 cells were treated with 4e and 4f at their previously reported IC_50_ values, and as compared to the control, they significantly increased the level of active caspase 3 by about 10 and 8-fold, respectively. As indicated in (Table 13 and Fig. 4) the results of the 4e and 4f compounds were remarkably like those of the standard drug staurosporine (a protein kinase inhibitor).

**Table 13:** Concentration of caspase 3 in Colo 205 after treated with 4e & 4f

Compound	Caspase 3 pg mL	Fold
	Colo_205	
4e	808.9 ± 11.9	9.71
4f	656.4 ± 13.1	7.88
Staurosporine	766.2 ± 10.4	9.2
Control	83.27 ± 3.53	1

### Molecular docking study

#### Docking at the active site of EGFR^WT^

**Fig. 5:**
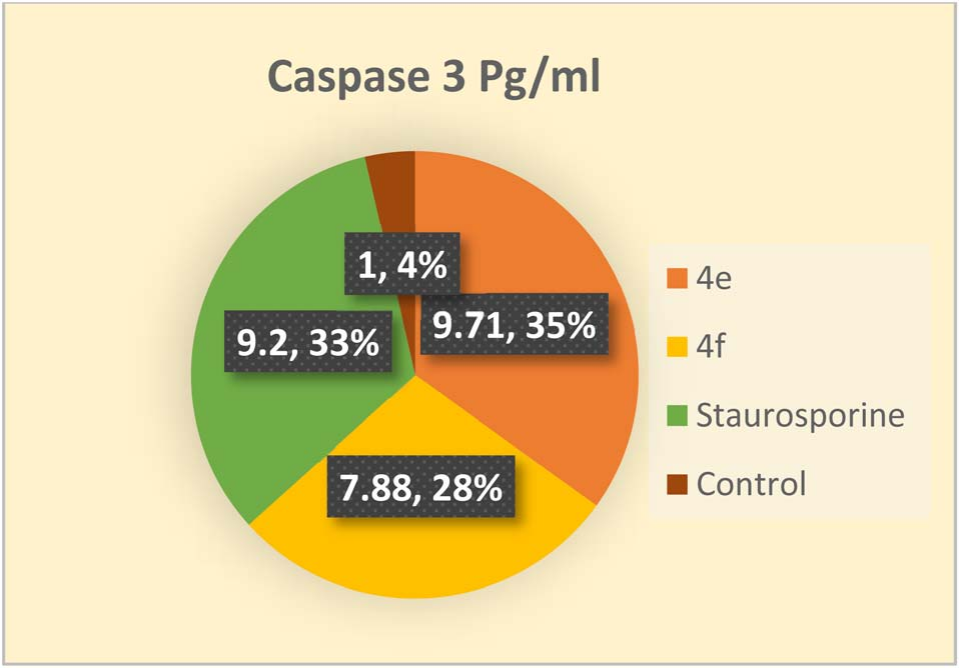
Level of active caspase-3 in Colo-205 cells treated with compounds 4e and 4f.

**Fig. 6:**
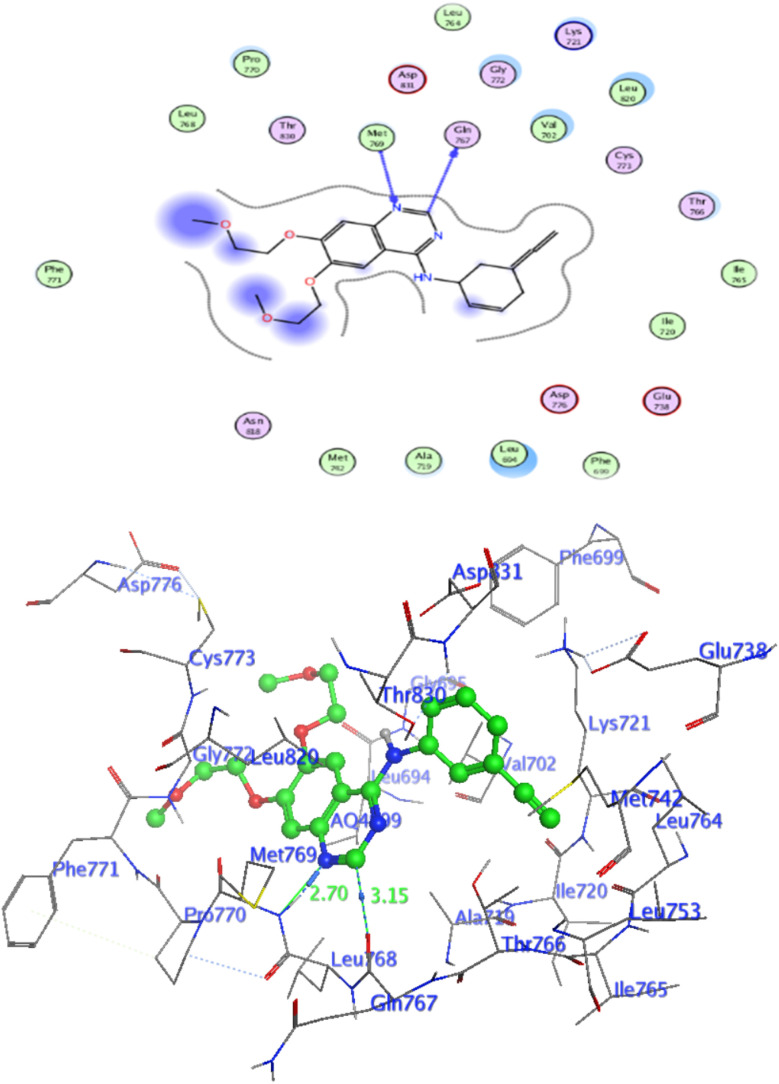
Interaction of Erlotinib with EGFR.

**Fig. 7:**
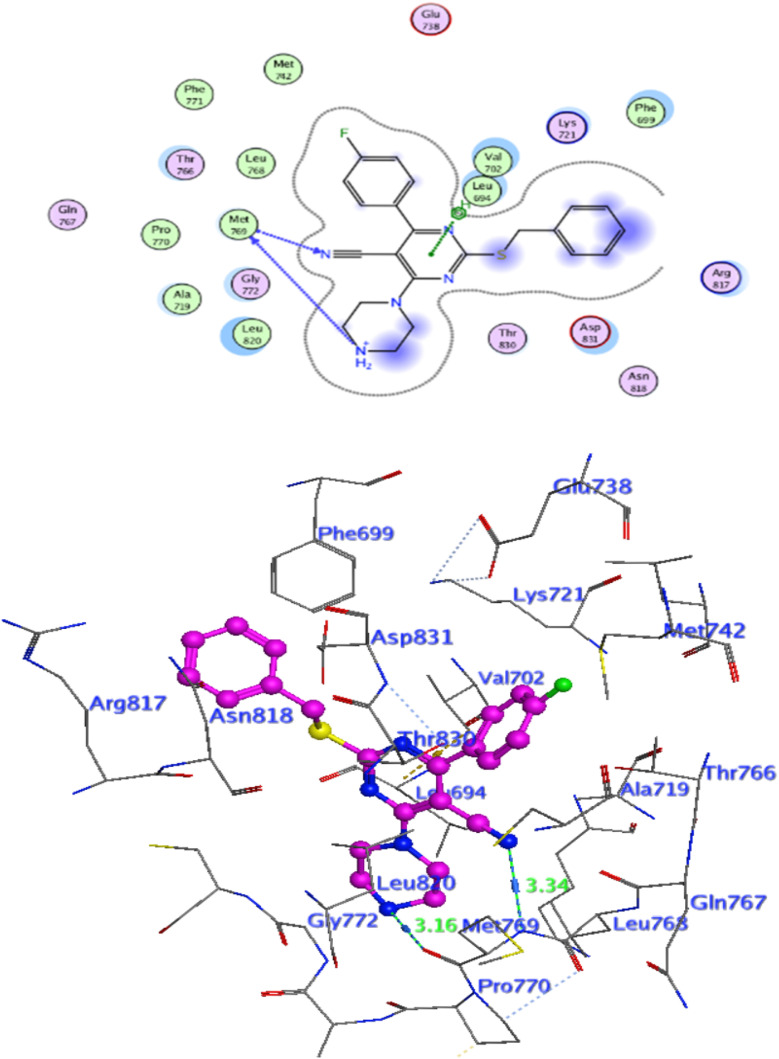
Interaction of 4e with EGFR.

For a better understanding of the potential binding mode of compounds 5a, and 5b the molecular docking study was performed. Docking study was done at the binding site of the crystal structure of EGFR^WT^ co-crystallized with erlotinib [PDB ID code 1m17]^46^ by MOE v 2014.0901 software. Using the best-scored conformation predicted by the MOE scoring function, the most stable docking model was chosen. The outcomes of the docking were displayed in (Fig. 5–7) and in Table 14. The docking process was validated by redocking the co-crystallized conformer of erlotinib to the active EGFR^WT^ site. After that, the root mean square deviation (RMSD) was used to compare the redocking pose to the original pose. The redocking pose of erlotinib was almost near the initial pose (RMSD = 1.83 Å) and interact with the main amino acids lining the active pocket as reported.^46^ Erlotinib interacts with the active binding site of EGFR^WT^ by two bonding interactions; the quinazoline N1 in erlotinib interacted with Met769 *via* H-bonding with bond distance 2.70 Å, and quinazoline C2 interacted with Gln767 *via* H-bonding with bond distance 3.15 Å (Fig. 5). The molecular docking analysis results of the newly synthesized compounds into the binding site of EGFR^WT^ demonstrated a strong to moderate affinity of the novel compounds to the binding site with the crucial amino acids which erlotinib bound to them. 4 N of piperazine ring in 4e and 4f interacted with Met769 *via* H-bond as H-donor with bond distance 3.16, and 3.11 Å, respectively. Also, N of cyano group in 4e interacted with Met769 as H-acceptor with bond distance 3.34 Å, furthermore, the pyrimidine ring in 4e could bind with Val 702 *via* pi-H bond with bond distance 3.86 Å. The aromatic ring of benzyl moiety in 4f could bind to Lys721 *via* a pi-cation bond with bond distance 4.47 Å (Fig. 6 and Fig. 7). the docking study results showed that the new compounds 4e and 4f interact with the crucial amino acids of the EGFR^wt^ active site (Met769, Val 702, Lys721).^22^

**Table 14:** The docking binding scores and the interacting residues of the newly synthesized compound against EGFR^WT^ and COX-2

Comp	EGRF^WT^ (PDB: 1M17)		COX-2 (PDB: 3LN1)	
	S score kcal mol^−1^	Interacting residue (H-bond & Pi-bond)	S score kcal mol^−1^	Interacting residue (H-bond & Pi-bond)
2b	−6.14	Val702, Lys721, Thr766	−7.20	Arg106
2c	−6.27	Val702, Lys721, Thr766	−6.39	Phe504
2d	−6.26	Val702, Lys721, Thr766	−5.64	Arg106
3a	−6.29	Val702, Lys721, Thr766	−7.38	Arg106
3b	−6.48	Val702, Lys721, Leu694	−7.35	Arg106
3c	−6.66	Met769, Lys721	−5.62	Arg499
3d	−6.37	Val702, Cys773	−4.86	Ser399
4a	−6.80	Val702, Lys721, Asp831	−7.46	Ser399
4b	−6.64	Val702, Lys721	−7.47	Arg106
4c	−6.61	Lys721, The766	−4.01	Arg106, Met508
4d	−6.80	Val702, Thr766	−4.64	Ser399, Val509
4e	−6.86	Met769, Val702	−6.01	Ser399
4f	−6.84	Met769, Lys721	−7.24	Ser339, Arg106
4g	−6.59	Met769, Lys721	−5.29	Ser339, Val509
4h	−6.43	Val702, Met742, Glu738	−4.99	Ser339, Leu517
4i	−6.70	Val702, thr766, Leu694	−5.06	Arg106
4j	−6.63	Val702, Lys721	−6.45	Ser339, Arg106
4k	−6.52	Met769, leu694	−3.04	Ser339, Val509
4l	−6.76	Met769, Val702, Lys721	−3.08	Ser339, Val509
4m	−6.15	Lys721	−1.02	Arg499
4n	−6.65	Lys721	−0.83	Ser339
4o	−6.74	Lys721	−0.92	Arg499
4p	−6.26	Lys721	−0.85	Val509
Erlotinib	−7.14	Met769, Gln767	—	—
Celecoxib	—	—	−8.23	Ser339, Arg499, Leu338

### Docking against COX-2 active site

**Fig. 8:**
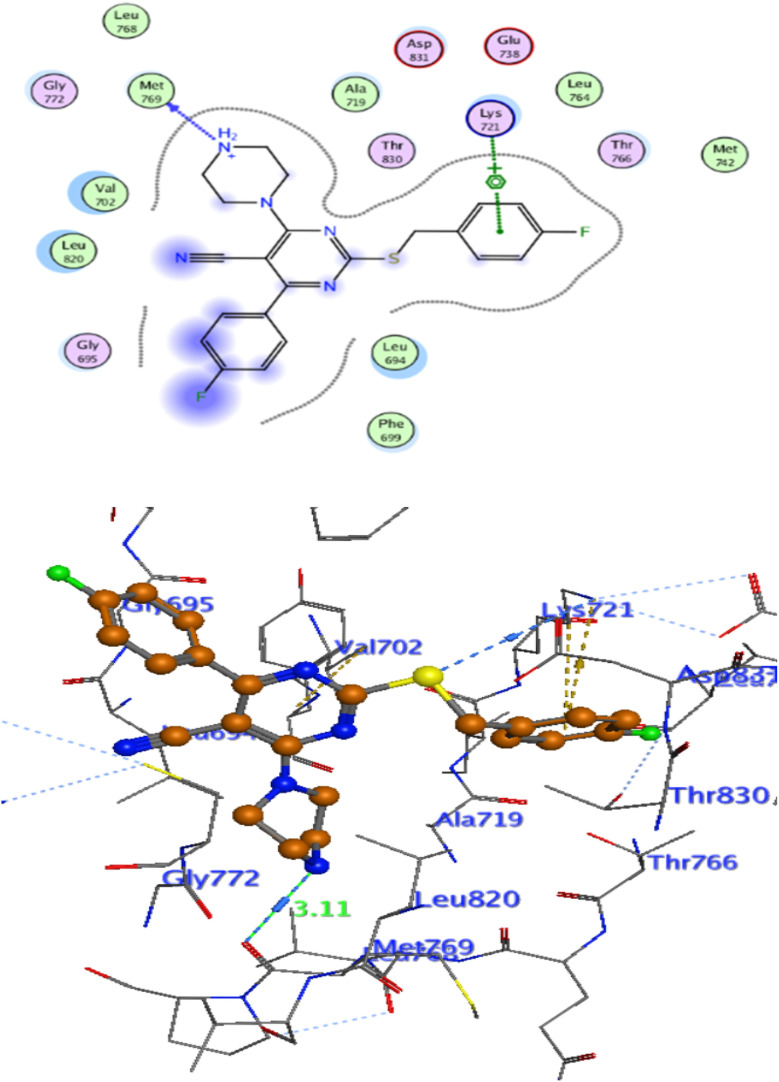
Interaction of 4f with EGFR.

**Fig. 9:**
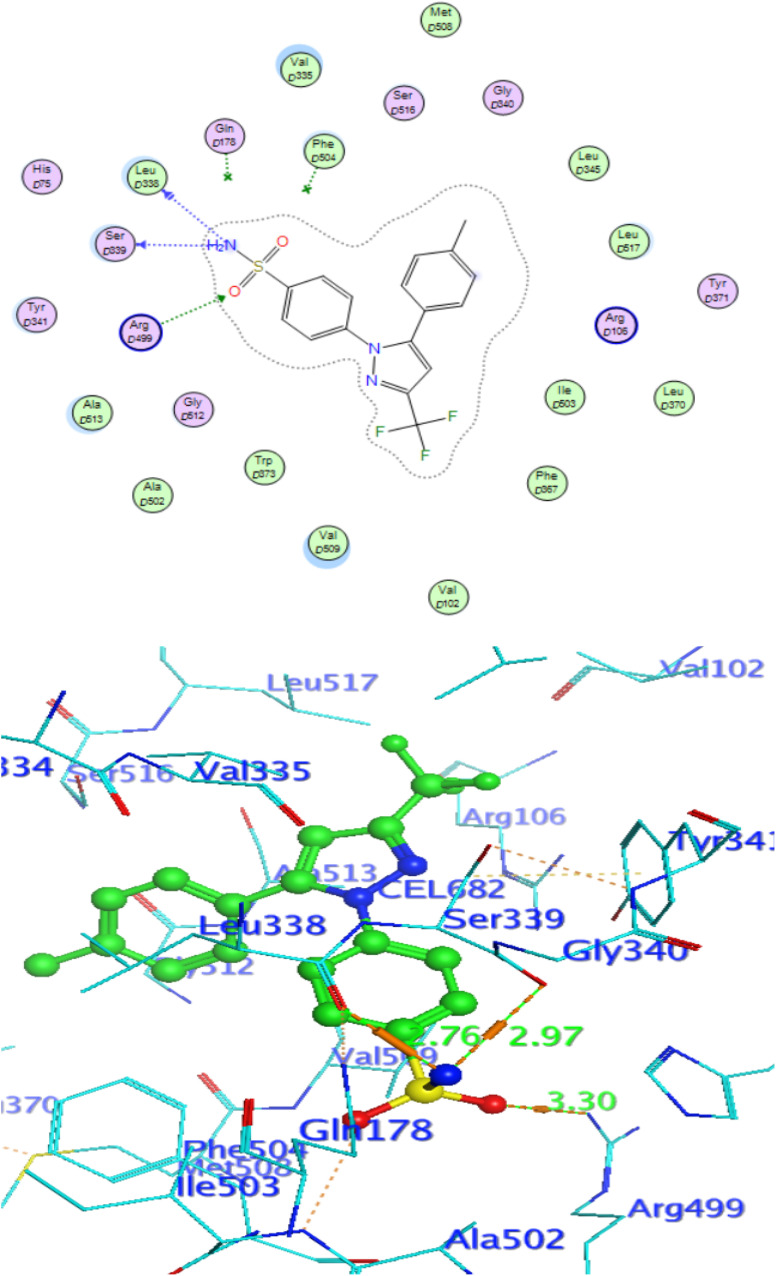
Celecoxib interaction with 3LN1 active site.

**Fig. 10:**
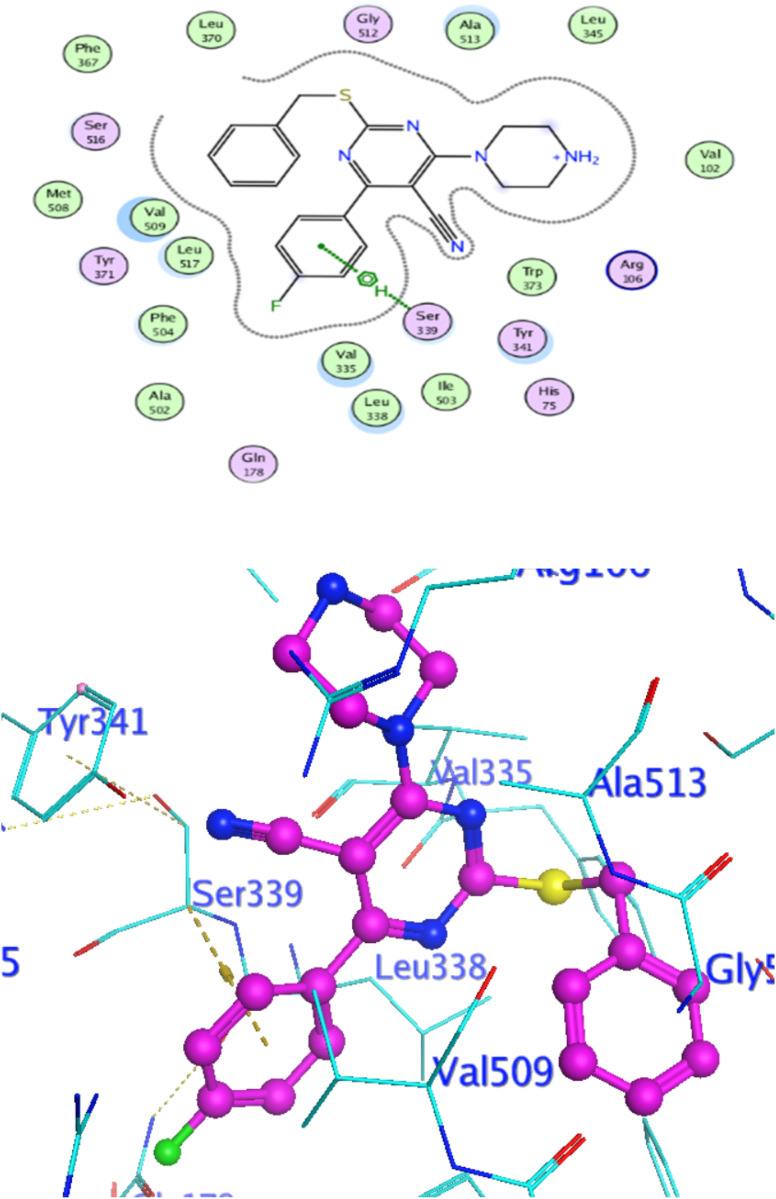
Interaction of 4e with 3NL1 active site.

In attempt to understand the binding mode of compounds 4e and 4f into the COX-2 active site, molecular docking study was carried out. Docking of the most potent cytotoxic compounds 4e, and 4f and celecoxib into the crystal structure of the COX-2 enzyme catalytic domain in complex with celecoxib [PDB ID code 3LN1]^47^ use the Molecular Operating Environment MOE software, 2014.0901. The results of the docking process were presented in (Fig. 8–10), and Table 14. Redocking the co-crystallized conformer of celecoxib against COX-2 binding site was done to confirm the docking procedure, next, the redocking pose was compared to the initial pose by (RMSD). Celecoxib was docked approximately in the same region (RMSD = 0.66). Interaction of celecoxib with COX-2 binding site (Fig. 6) displayed H-bonding interactions between N of sulphonamide group and Leu338 with bond distance 2.76 Å, and H-bonding interactions between N of sulphonamide group and Ser339 with bond distance 2.97 Å, furthermore it showed H-bonding interaction between O of sulphonamide group and Arg499 with bond distance 3.30 Å. The docking study of the newly synthesized compounds into COX-2 binding site displayed interaction of the investigated compounds with the active site through the main amino acid (Ser339) bound to celecoxib. The aromatic ring of *P*-fluorophenyl in 4e could binds to Ser339 *via* Pi-bond with bond distances 3.82 Å. On the other hand, *P*-fluorophenyl in 4f could binds to Ser339 *via* Pi-bond. While N of cyano group in 4f could bind to Arg106 *via* H-bonding with bond distance 3.05 Å. from the obtained results the value of the *P*-fluorophenyl moiety in the interaction with the main amino acid Ser339 is noticed.

### Pharmacophore analysis study

**Fig. 11:**
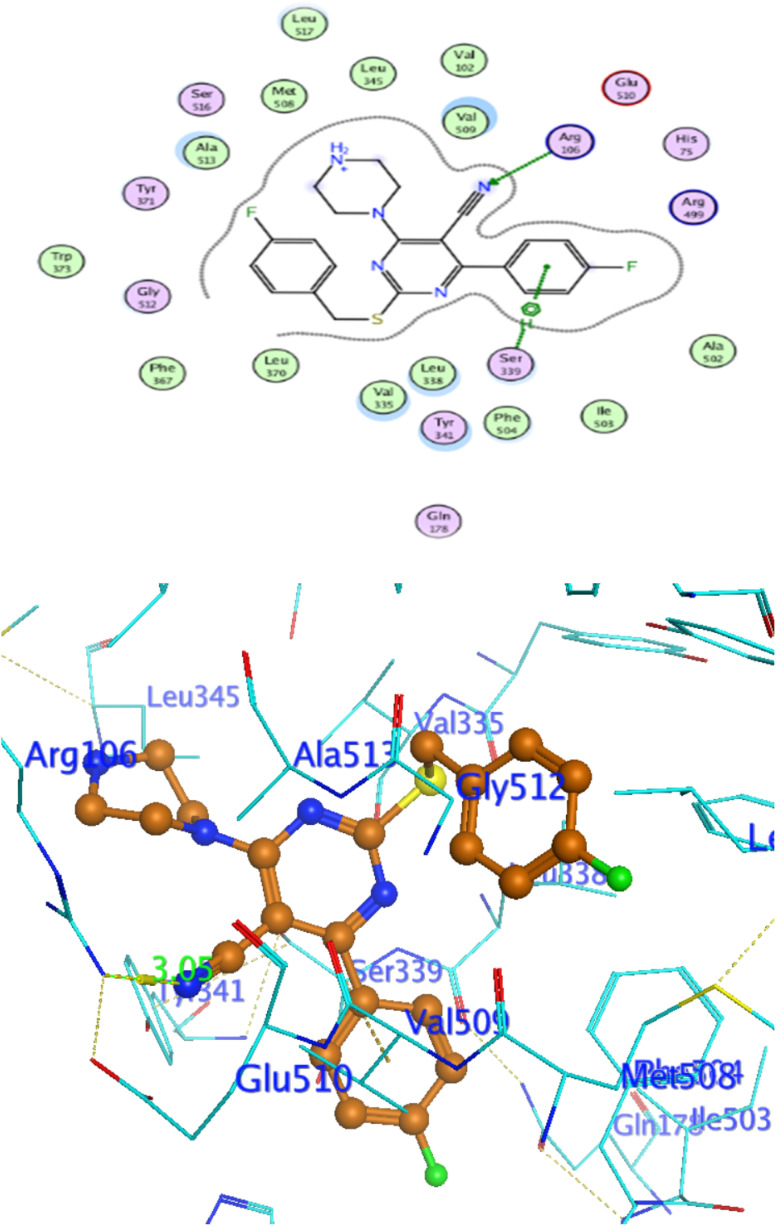
Interaction of 4f with 3NL1 active site.

The most potent candidate in our study which show inhibitory activity against EGFR^WT^/COX-2 was undergoing flexible alignment with erlotinib, and celecoxib individually to determine its pharmacophoric features. Pharmacophore mapping was carried out by MOE. At First, 4e and erlotinib were underwent flexible alignment using flexible alignment tool in MOE and the best alignment structure was selected. then generating a 3D pharmacophore query to mapping the pharmacophore of these aligned structures by using MOE v 2014.0901 (Fig. 11), six features are shared between compound 4e and erlotinib; F1: aromatic moiety, F2: Pi-ring center, F3, F4, and F5: hydrogen bond acceptor, F6: hydrogen bond acceptor/donor. The two compounds' good alignment and the pharmacophore mapping may indicate that compound 5a possesses the necessary pharmacophoric characteristics arranged appropriately as erlotinib, which may assist to explain why it is such an effective EGFR inhibitor and why it is so promiscuous.

**Fig. 12:**
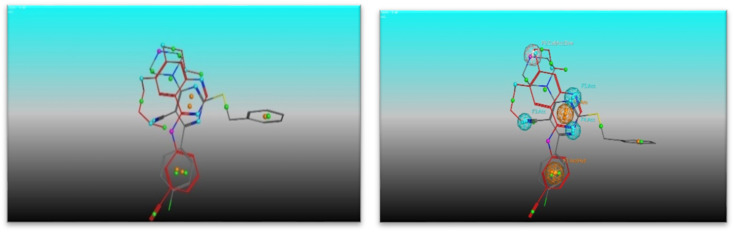
(Left) alignment of 4e (grey) with erlotinib (red) and (right) the shared pharmacophore features (green for hydrophobic, purple for H. B. donor, blue for H. B. acceptor and orange for aromatic moiety).

**Fig. 13:**
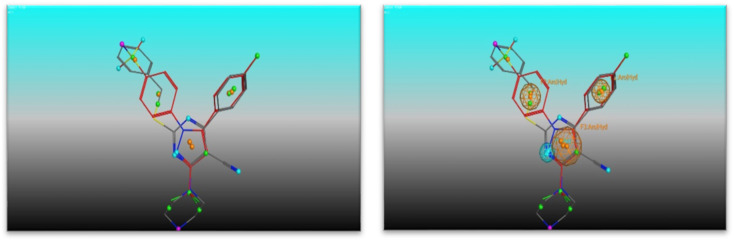
(Left) alignment of 4e (grey) with celecoxib (red) and (right) the shared pharmacophore features (green for hydrophobic, purple for H. B. donor, blue for H. B. acceptor and orange for aromatic moiety).

Furthermore, alignment of compound 4e with celecoxib was carried out by the flexible alignment function in the MOE software (Fig. 12, then pharmacophore query was builded. the result of the flexible alignment was four features are shared between 5a and celecoxib, F1: aromatic moiety, F2: hydrophobic center, F3: Pi-ring center, F4: hydrogen bond acceptor Fig. 13). These shared pharmacophoric features between them may illustrate the activity of 4e against COX-2. So that may give better understanding of the anti COX-2 activity of the new compound 4e.

### *In silico* studies

For the most cytotoxic members (2a, 3c, 3d, 4b, 4c, 4d, 4e, 4f, and 4i, 4j, 4k, 4l) physicochemical parameters were computed. Physicochemical parameters including log *P* (partition coefficient), molecular weight (MW), hydrogen bond acceptors and donors, and the number of free rotatable bonds have a major effect on the drug. Physicochemical parameters of the newly synthesized compounds were examined using the online tool Swissadme (Table 15). Topological polar surface area, or TPSA, measures a molecule's capability for intestinal absorption; a TPSA value less than 140 is required.^48^ The TPSA range for all newly synthesized compounds in this study is between 74.87 to 133.16. The GI absorption is high in all tested compounds except 3c and 4c. All *in silico* tested compounds cannot permeate BBB. Considering binding with plasma protein (P-gp parameter) compounds (2b, 3c, 3d, 4c, 4i, 4k) showed no binding with plasma protein. But compounds (4b, 4d, 4e, 4f, 4j, 4l) disclosed binding with plasma protein. Further physiochemical descriptors like hydrogen bond acceptors, hydrogen bond donors and the number of rotatable bonds went aline with Lipniski rule of five.^49^ All tested compound displayed acceptable bioavailability score >0.25. The parameters showed the suitability of the new compounds as potential therapeutic candidates.

**Table 15:** Physicochemical properties values of the most active candidates (2b, 3c, 3d, 4b, 4c, 4d, 4e, 4f, 4i, 4j, 4k, 4l)^a^

Compound	Mol. Wt^b^	Log *P*^c^	TPSA^d^ (A2)	*n*-ROTB^e^	*n*-HB donor^f^	*n*-HB acceptor^g^	GI absorption	BBB permeant	P-gp substrate	Lipinski's violation	Bioavailability score
2b	355.36	3.15	94.84	4	1	5	High	No	No	0	0.55
3c	400.81	3.15	120.69	5	0	6	Low	No	No	0	0.55
3d	369.84	3.60	74.87	4	0	4	High	No	No	0	0.55
4b	424.47	3.60	87.34	5	0	6	High	No	Yes	0	0.55
4c	451.47	1.85	133.16	6	0	7	Low	No	No	0	0.55
4d	420.50	2.98	87.34	5	0	5	High	No	Yes	0	0.55
4e	405.49	2.77	90.14	5	1	5	High	No	Yes	0	0.55
4f	423.48	3.14	90.14	5	1	6	High	No	Yes	0	0.55
4i	419.52	2.98	81.35	5	0	5	High	No	No	0	0.55
4j	437.51	3.35	81.35	5	0	6	High	No	Yes	0	0.55
4k	464.52	2.06	127.17	6	0	7	High	No	No	0	0.55
4l	433.54	2.06	81.35	5	0	5	High	No	Yes	0	0.55

a Physicochemical parameters were getted from online tool http://www.swissadme.ch.

b Molecular weight.

c Theoretical log *P*.

d Topological polar surface area.

e Total number of free rotated bounds.

f Number of hydrogen bond donors.

g Number of hydrogen bond acceptors.

## Conclusion

A variety of novel anti-cancer compounds were designed, synthesized and assessed for antiproliferative activities against NCI 60 cell line panel. Compounds 4e, and 4f (with Piperazine moiety) have the most potent cytotoxic activity. these compounds have been evaluated for their inhibitory activity against EGFR^WT^ (IC_50_ = 0.09–0.23 μM) relative to erlotinib (IC_50_ = 0.03 μM). Moreover, Compounds 4e, and 4f showed selective inhibitory activity against COX-2 *in vitro* (IC_50_ = 1.28–5.26 μM, SI = 8.55–6.01) relative to celecoxib (IC_50_ = 2.59 μM, SI = 8.20). Additionally, compounds 4e, and 4f had the ability to arrest cell cycle at G1 phase and lead to increasing the proportion of the cells in G0–G1 phase in Colo-205. The considerable increase in the percentage of annexin V-FITC binding cells indicated that 4e, and 4f were actively undergoing apoptosis. Also, these two substances elevated the amount of active caspase-3, which further supports that a caspase-3-dependent pathway was adopted to trigger cancer cell apoptosis. Fortunately, compounds 4e, and 4f had low cytotoxicity on normal epithelial colon cell (IC_50_ = 46.34, 28.25 μM), respectively and was safer than the clinically prescribed drug Staurosporine (IC_50_ = 23.00 μM). Molecular docking study demonstrated that, they interacted with EGFR^WT^ crucial amino acids lining the active site. Additionally, these substances demonstrated comparable binding interactions and orientation as selective COX-2 inhibitors. As a whole, anti-proliferation and EGFR^WT^ and COX-2 inhibition have been found to be significantly correlated. In practical terms, our novel compounds 4e, and 4f appear to be exciting hits for further optimization and development of new potent, and selective anti-cancer agents.

## Experimental part

### General

Without any additional purification, we used all commercially accessible reagents. The Stuart apparatus was used to measure melting points, and the results were reported as it is. IR spectra were carried out as KBr discs on a Shimadzu IR 435 spectrophotometer (Misr University for Science and Technology, Faculty of Pharmacy, Egypt), readings were reported in cm^−1^. ^1^H NMR spectra were recorded on BRUKER 400 MHz spectrophotometers using TMS as a standard signal, and chemical shift values were recorded in ppm on a *δ* scale. ^13^C NMR spectra were recorded on BRUKER 100 MHz spectrophotometer using TMS as an standard signal. On *δ* scale, chemical shift readings were recorded in ppm. The ^19^F NMR spectra were recorded on BRUKER 376.25 MHz. ^1^H NMR, ^13^C NMR, and ^19^F NMR were performed at (center for drug discovery research and development, Ain Shams University, Egypt). Mass spectra and elemental analyses were recorded on the Regional Centre for Mycology and Biotechnology, Al-Azhar University, Egypt. Using TLC aluminum sheets that had been coated with UV fluorescent silica gel (Merck 60F 254), the reactions' progression was observed and visualized by short and long UV lamp. The purity of compounds (2c, 3c, 4d, 4f) was detected by HPLC at the lambda max of each of them.

## Chemistry

The reported procedure was followed to prepare compounds 1,^28^ and 2a.^50^

### The general procedure of synthesis of compounds (2a–d)

A solution of appropriate benzyl chloride (1.28 g, 8 mmol) in dimethylformamide (5 mL) was added dropwise with stirring to a solution of compound (1) (2 gm, 8 mmol) and anhydrous potassium carbonate (1.1 g, 8 mmol) in dimethylformamide (20 mL), maintained at 0–5 °C. After 3 h, the reaction mixture was poured into water (20 mL), filtered and neutralized with acetic acid (2 mL). The resulting precipitate was collected by filtration, dried and crystallized from acetone.

#### 2-((4-Fluorobenzyl)thio)-4-(4-fluorophenyl)-6-oxo-1,6-dihydropyrimidine-5-carbonitrile (2b)

White powder; yield: 93%; m.p. 210–212 °C; IR (KBr, cm^−1^): 3446 (NH), 3001 (C–H aromatic), 2852 (C–H aliphatic), 2220 (CN), 1654 (C=O); ^1^H NMR (DMSO-*d*_6_-400 MHz, *δ* ppm): 4.54 (s, 2H, S-CH_2_), 7.13–7.18 (m, 2H, ArH), 7.41–7.48 (m, 4H, ArH), 8.03 (dd, 2H, *J* = 8.8 Hz, *J* = 5.6 Hz, ArH); ^13^C NMR (DMSO-*d*_6_-100 MHz, *δ* ppm): 33.88, 93.49, 115.79 (2C, d, *J*_CF_^2^ = 22 Hz), 116.21 (2C, d, *J*_CF_^2^ = 22 Hz), 131.51 (2C, d, *J*_CF_^3^ = 8 Hz), 131.91 (2C, d, *J*_CF_^3^ = 9 Hz), 133.27, 144.53, 149.57, 161.77, 161.94 (1C, d, *J*_CF_^1^ = 243 Hz), 164.48 (1C, d, *J*_CF_^1^ = 249 Hz), 166.31, 166.56; MS (*m*/*z*): 134.59 (100%), 355.36 (M^+^, 19.02%), 356.73 (M + 1, 15.59%); Anal. Calcd. For C_18_H_11_F_2_N_3_OS (355.36): C: 60.84; H: 3.12; N: 11.82. Found: C: 61.08; H: 3.20; N: 12.09.

#### 4-(4-Fluorophenyl)-2-((4-nitrobenzyl) thio)-6-oxo-1,6-dihydropyrimidine-5-carbonitrile (2c)

White powder; yield: 76%; m.p. 295–297 °C; IR (KBr, cm^−1^): 3444 (NH), 3066 (C–H aromatic), 2843 (C–H aliphatic), 2206 (CN), 1600 (C=O); ^1^H NMR (DMSO-*d*_6_-400 MHz, *δ* ppm): 4.41 (s, 2H, S-CH_2_), 7.27–7.31 (m, 2H, ArH), 7.68 (d, 2H, *J* = 8.00 Hz, ArH), 7.81 (dd, 2H, *J* = 8.80 Hz, *J* = 4.40 Hz, ArH), 8.16 (d, 2H, *J* = 8.00 Hz, ArH); ^13^C NMR (DMSO-*d*_6_-100 MHz, *δ* ppm): 33.52, 89.56, 115.51 (2C, d, *J*_CF_^2^ = 22 Hz), 120.46, 123.88 (2C), 130.47, 130.92 (2C, d, *J*_CF_^3^ = 10 Hz), 134.62 (1C, d, *J*_CF_^4^ = 3 Hz), 146.74, 148.43, 163.38 (1C, d, *J*_CF_^1^ = 246 Hz), 166.43, 170.64, 171.40; ^19^F NMR (DMSO-*d*_6_-376.25 MHz, *δ* ppm): −111.37; MS (*m*/*z*): 225.81 (100%), 382.48 (M^+^, 59.31%); Anal. Calcd. For C_18_H_11_F_2_N_4_O_3_S (382.37): C: 56.54; H: 2.90; N: 14.65. Found: C: 56.79; H: 3.15; N: 14.74. HPLC purity = 98.9%.

#### 4-(4-Fluorophenyl)-2-((4-methylbenzyl)thio)-6-oxo-1,6-dihydropyrimidine-5-carbonitrile (2d)

Yellowish white powder; yield: 87%; m.p. 230–232 °C; IR (KBr, cm^−1^): 3419 (NH), 3078 (C–H aromatic), 2922 (C–H aliphatic), 2204 (CN), 1664 (C=O); ^1^H NMR (DMSO-*d*_6_-400 MHz, *δ* ppm): 2.26 (s, 3H, CH_3_), 4.28 (s, 2H, S-CH_2_), 7.09 (d, 2H, *J* = 8.00 Hz, ArH), 7.27–7.34 (m, 4H, ArH), 7.88 (t, 2H, ArH); ^13^C NMR (DMSO-*d*_6_-100 MHz, *δ* ppm): 21.12, 34.19, 89.83, 115.61 (2C, d, *J*_CF_^2^ = 22 Hz), 119.92, 129.27 (2C), 129.37 (2C), 131.07 (2C, d, *J*_CF_^3^ = 9 Hz), 134.81 (1C, d, *J*_CF_^4^ = 3 Hz), 135.78, 136.45, 163.55 (1C, d, *J*_CF_^1^ = 246 Hz), 166.38, 169.61, 171.49; ^19^F NMR (DMSO-*d*_6_-376.25 MHz, *δ* ppm): −109.93; MS (*m*/*z*): 218.45 (100%), 351.72 (M^+^, 21.44%); Anal. Calcd. For C_19_H_14_FN_3_OS (351.40): C: 64.94; H: 4.02; N: 11.96. Found: C: 64.75; H: 4.16; N: 12.18.

### General procedure of synthesis of compounds (3a–d)

A mixture of each compounds 2a–d (1gm, 3 mmol) and phosphorus oxychloride (10 mL) was refluxed for 5 h. The reaction mixture was gradually poured onto crushed ice with vigorous stirring. The precipitate was collected by filtration, dried, and crystallized from the appropriate solvent to give compounds (3a–d).

#### 2-(Benzylthio)-4-chloro-6-(4-fluorophenyl) pyrimidine-5-carbonitrile (3a)

White powder; yield: 71%; m.p. 125–127 °C; IR (KBr, cm^−1^): 3066 (C–H aromatic), 2920 (C–H aliphatic), 2233 (CN); ^1^H NMR (DMSO-*d*_6_-400 Hz, *δ* ppm): 4.53 (s, 2H, S-CH_2_), 7.26 (d, 1H, *J* = 8.00 Hz, ArH), 7.32 (t, 2H, ArH), 7.44 (d, 4H, *J* = 8.00 Hz, ArH), 8.04 (t, 2H, ArH); ^13^C NMR (DMSO-*d*_6_-100 Hz, *δ* ppm): 35.52, 102.16, 115.25, 116.51 (2C, d, *J*_CF_^2^ = 22 Hz), 127.93, 128.98, 129.57 (2C, d, *J*_CF_^3^ = 9 Hz), 131.20 (1C, d, *J*_CF_^4^ = 3 Hz), 132.38, 132.47, 136.96, 161.74 (1C, d, *J*_CF_^1^ = 242 Hz), 163.67, 166.17, 167.76, 174.57; MS (*m*/*z*): 76.11 (100%), 355.75 (M^+^, 16.52%); Anal. Calcd. For C_18_H_11_ClFN_3_S (355.82): C: 60.76; H: 3.12; N: 11.81. Found: C: 60.92; H: 3.35; N: 12.05.

#### 4-Chloro-2-((4-fluorobenzyl) thio)-6-(4-fluorophenyl) pyrimidine-5-carbonitrile (3b)

White powder; yield: 63%; m.p. 135–137 °C; IR (KBr, cm^−1^): 3082 (C–H aromatic), 2918 (C–H aliphatic), 2222 (CN); ^1^H NMR (DMSO-*d*_6_-400 MHz, *δ* ppm): 4.52 (s, 2H, S-CH_2_), 7.14 (t, 2H, ArH), 7.40–7.50 (m, 4H, ArH), 8.00–8.06 (dd, 2H, *J* = 8.00 Hz, *J* = 4.00 Hz, ArH); ^13^C NMR (DMSO-*d*_6_-100 MHz, *δ* ppm): 34.68, 102.20, 115.74 (2C, d, *J*_CF_^2^ = 25 Hz), 115.88, 116.50 (2C, d, *J*_CF_^2^ = 22 Hz), 131.52 (2C, d, *J*_CF_^3^ = 8 Hz), 131.91 (2C, d, *J*_CF_^3^ = 9 Hz), 133.14 (1C, d, *J*_CF_^4^ = 3 Hz), 133.27 (1C, d, *J*_CF_^4^ = 3 Hz), 161.29 (1C, d, *J*_CF_^1^ = 241 Hz), 162.99, 164.51 (1C, d, *J*_CF_^1^ = 250 Hz), 167.78, 174.45; ^19^F NMR (DMSO-*d*_6_-376.25 MHz, *δ* ppm): −106.96, −114.86; MS (*m*/*z*): 224.87 (100%), 373.30 (M^+^, 15.45%); Anal. Calcd. For C_18_H_10_ClF_2_N_3_S (373.81): C: 57.84; H: 2.70; N: 11.24. Found: C: 58.07; H: 2.83; N: 11.51.

#### 4-Chloro-6-(4-fluorophenyl)-2-((4-nitrobenzyl) thio) pyrimidine-5-carbonitrile (3c)

White powder; yield:59%; m.p. 155–157 °C; IR (KBr, cm^−1^): 3078 (C–H aromatic), 2989 (C–H aliphatic), 2235 (CN); ^1^H NMR (DMSO-*d*_6_-400 MHz, *δ* ppm): 4.65 (s, 2H, S-CH_2_), 7.47 (t, 2H, ArH), 7.74 (d, 2H, ArH), 8.02 (t, 2H, ArH), 8.17 (d, 2H, ArH); ^13^C NMR (DMSO-*d*_6_-100 MHz, *δ* ppm): 34.68, 102.51, 115.18, 116.22 (2C, d, *J*_CF_^2^ = 22 Hz), 123.98, 124.01, 130.72 (2C, d, *J*_CF_^3^ = 10 Hz), 131.10, 131.87, 131.97, 145.59, 147.17, 163.06, 164.50 (1C, d, *J*_CF_^1^ = 249 Hz), 167.91, 173.95; ^19^F NMR (DMSO-*d*_6_-376.25 MHz, *δ* ppm): −106.90; MS (*m*/*z*): 45.24 (100%), 400.35 (M^+^, 14.12%); Anal. Calcd. For C_18_H_10_ClFN_4_O_2_S (400.81): C: 53.94; H: 2.51; N: 13.98. Found: C: 53.82; H: 2.79; N: 14.07. HPLC purity = 100%.

#### 4-Chloro-6-(4-fluorophenyl)-2-((4-methylbenzyl)thio)pyrimidine-5-carbonitrile (3d)

White powder; yield: 59%; m.p. 110–112 °C; IR (KBr, cm^−1^): 3010 (C–H aromatic), 2880 (C–H aliphatic), 2240 (CN); ^1^H NMR (DMSO-*d*_6_-400 MHz, *δ* ppm): 2.25 (s, 3H, CH_3_), 4.47 (s, 2H, S-CH_2_), 7.12 (d, 2H, *J* = 8.00 Hz, ArH), 7.32 (d, 2H, *J* = 8.00 Hz, ArH), 7.46 (t, 2H, ArH), 8.05 (t, 2H, ArH); ^13^C NMR (DMSO-*d*_6_-100 MHz, *δ* ppm): 21.11, 34.91, 89.38, 115.61 (2C, d, *J*_CF_^2^ = 22 Hz), 119.92 (2C), 129.32 (2C, d, *J*_CF_^3^ = 10 Hz), 131.03 (2C), 131.12, 134,21, 136.45, 163.54 (1C, d, *J*_CF_^1^ = 246 Hz), 166.38, 169.61, 171.49; MS (*m*/*z*): 96.12 (100%), 369.83 (M^+^, 35.64%); Anal. Calcd. For C_19_H_13_ClFN_3_S (369.84): C: 61.70; H: 3.54; N: 11.36. Found: C: 61.83; H: 3.66; N: 11.59.

### General procedure of synthesis of compounds (4a–p)

To a solution of compounds 3a–d (1 mmol) in dry benzene (10 mL), the appropriate secondary amine (2 mmol) was added then refluxed for 4 h. The reaction mixture was filtered on hot. The solvent was allowed to evaporate from the resulting filtrate, the obtained precipitate was dried and recrystallized from benzene.

#### 2-(Benzylthio)-4-(4-fluorophenyl)-6-morpholinopyrimidine-5-carbonitrile (4a)

White crystals; yield: 50%; m.p. 140–142 °C; IR (KBr, cm^−1^): 3059 (C–H aromatic), 2962, 3860 (C–H aliphatic), 2210 (CN); ^1^H NMR (DMSO-*d*_6_-400 MHz, *δ* ppm): 3.69 (s, 4H, CH_2_–N–CH_2_), 3.91 (s, 4H, CH_2_–O–CH_2_), 4.42 (s, 2H, S-CH_2_), 7.24 (t, 1H, ArH), 7.30 (m, 6H, ArH), 7.90 (t, 2H, ArH); ^13^C NMR (DMSO-*d*_6_-100 MHz, *δ* ppm): 35.00, 47.56 (2C), 66.33 (2C), 84.35, 115.97 (2C, d, *J*_CF_^2^ = 22 Hz), 118.30, 127.59, 128.93 (2C), 129.18 (2C), 132.33 (2C, d, *J*_CF_^3^ = 10 Hz), 132.93 (1C, d, *J*_CF_^4^ = 3 Hz), 137.99, 162.04, 164.32 (1C, d, *J*_CF_^1^ = 247 Hz), 169.36, 172.53; ^19^F NMR (DMSO-*d*_6_-376.25 MHz, *δ* ppm): −108.91; MS (*m*/*z*): 91.29 (100%), 406.17 (M^+^, 16.44%); Anal. Calcd. For C_22_H_19_FN_4_OS (406.48): C: 65.01; H: 4.71; N: 13.78. Found: C: 65.23; H: 4.89; N: 11.05.

#### 2-((4-Fluorobenzyl) thio)-4-(4-fluorophenyl)-6-morpholinopyrimidine-5-carbonitrile (4b)

White powder; yield: 68%; m.p. 150–152 °C; IR (KBr, cm^−1^): 3064 (C–H aromatic), 2916, 2852 (C–H aliphatic), 2210 (CN); ^1^H NMR (DMSO-*d*_6_-400 MHz, *δ* ppm): 3.70 (t, 4H, *J* = 4.40 Hz, CH_2_–N–CH_2_), 3.91 (t, 4H, *J* = 4.4 Hz, CH_2_–O–CH_2_), 4.41 (s, 2H, S-CH_2_), 7.14 (t, 2H, *J* = 8.00 Hz, ArH), 7.38 (t, 2H, *J* = 8.00 Hz, ArH), 7.45 (t, 2H, ArH), 7.91 (t, 2H, ArH); ^13^C NMR (DMSO-*d*_6_-100 MHz, *δ* ppm): 34.14, 47.56 (2C), 66.32 (2C), 84.40, 115.67 (2C, d, *J*_CF_^2^ = 21 Hz), 115.98 (2C, d, *J*_CF_^2^ = 22 Hz), 118.28, 131.11 (2C, d, *J*_CF_^3^ = 8 Hz), 132.31 (2C, d, *J*_CF_^3^ = 9 Hz), 132.91 (1C, d, *J*_CF_^4^ = 3 Hz), 134.30 (1C, d, *J*_CF_^4^ = 3 Hz), 161.74 (1C, d, *J*_CF_^1^ = 242 Hz), 163.11, 164.34 (1C, d, *J*_CF_^1^ = 248 Hz), 169.39, 172.40; Anal. Calcd. For C_22_H_18_F_2_N_4_OS (424.47): C: 62.25; H: 4.27; N: 13.20. Found: C: 62.12; H: 4.58; N: 13.41.

#### 4-(4-Fluorophenyl)-6-morpholino-2-((4-nitrobenzyl) thio) pyrimidine-5-carbonitrile (4c)

White powder; yield: 64%; m.p. 170–172 °C; IR (KBr, cm^−1^): 3074 (C–H aromatic), 2974, 2856 (C–H aliphatic), 2210 (CN); ^1^H NMR (DMSO-*d*_6_-400 MHz, *δ* ppm): 3.68 (t, 4H, CH_2_–N–CH_2_), 3.89 (t, 4H, CH_2_–O–CH_2_), 4.53 (s, 2H, S-CH_2_), 7.36 (t, 2H, *J* = 8.00 Hz, ArH), 7.68 (d, 2H, *J* = 8.00 Hz, ArH), 7.88 (dd, 2H, *J* = 8.00 Hz, *J* = 5.60 Hz, ArH), 8.16 (d, 2H, *J* = 8.00 Hz, ArH); ^13^C NMR (DMSO-*d*_6_-100 MHz, *δ* ppm): 34.23, 47.56 (2C), 66.30 (2C), 84.59, 116.00 (2C, d, *J*_CF_^2^ = 22 Hz), 118.20, 124.02 (2C), 130.32 (2C), 132.32 (2C, d, *J*_CF_^3^ = 9 Hz), 132.83 (1C, d, *J*_CF_^4^ = 3 Hz), 146.76, 146.99, 162.00, 164.35 (1C, d, *J*_CF_^1^ = 248 Hz), 169.46, 171.87; Anal. Calcd. For C_22_H_18_FN_5_O_3_S (451.48): C: 58.53; H: 4.02; N: 15.51. Found: C: 58.80; H: 4.23; N: 15.69.

#### 4-(4-Fluorophenyl)-2-((4-methylbenzyl) thio)-6-morpholinopyrimidine-5-carbonitrile (4d)

White crystals; yield:57%; m.p. 158–160 °C; IR (KBr, cm^−1^): 3018 (C–H aromatic), 2964,2858 (C–H aliphatic), 2210 (CN); ^1^H NMR (DMSO-*d*_6_-400 MHz, *δ* ppm): 2.26 (s, 3H, –CH_3_), 3.70 (t, 4H, CH_2_–N–CH_2_), 3.92 (t, 4H, CH_2_–O–CH_2_), 4.37 (s, 2H, S-CH_2_), 7.12 (d, 2H, *J* = 8.00 Hz, ArH), 7.28 (d, 2H, *J* = 8.00 Hz, ArH), 7.37 (t, 2H, ArH), 7.91 (t, 2H, ArH); ^13^C NMR (DMSO-*d*_6_-100 MHz, *δ* ppm): 21.13, 34.80, 47.55 (2C), 66.33 (2C), 84.29, 115.97 (2C, d, *J*_CF_^2^ = 21 Hz), 118.31, 129.14 (2C), 129.49 (2C), 132.31 (2C, d, *J*_CF_^3^ = 9 Hz), 132.98 (1C, d, *J*_CF_^4^ = 3 Hz), 134.75, 136.81, 162.03, 164.32 (1C, d, *J*_CF_^1^ = 248 Hz), 169.35, 172.63; ^19^F NMR (DMSO-*d*_6_-376.25 MHz, *δ* ppm): −108.91; Anal. Calcd. For C_23_H_21_FN_4_OS (420.51): C: 65.70; H: 5.03; N: 13.32. Found: C: 65.59; H: 5.20; N: 13.49. HPLC purity = 100%.

#### 2-(Benzylthio)-4-(4-fluorophenyl)-6-(piperazin-1-yl) pyrimidine-5-carbonitrile (4e)

Yellowish white crystals; yield: 66%; m.p. 120–122 °C; IR (KBr, cm^−1^): 3446 (NH), 3059 (C–H aromatic), 2962, 2848 (C–H aliphatic), 2208 (CN); ^1^H NMR (DMSO-*d*_6_-400 MHz, *δ* ppm): 1.21 (s, 1H, NH), 2.80 (t, 4H, CH_2_–NH–CH_2_), 3.83 (t, 4H, CH_2_–N–CH_2_), 4.42 (s, 2H, S-CH_2_), 7.24 (t, 1H, *J* = 8.00 Hz, ArH), 7.30–7.42 (m, 6H, ArH), 7.90 (dd, 2H, *J* = 8.00 Hz, *J* = 4.00 Hz, ArH); ^13^C NMR (DMSO-*d*_6_-100 MHz, *δ* ppm): 34.97, 45.95 (2C), 48.63 (2C), 83.91, 115.90 (2C, d, *J*_CF_^2^ = 22 Hz), 118.43, 127.57, 128.91 (2C), 129.14 (2C), 132.28 (2C, d, *J*_CF_^3^ = 9 Hz), 133.03 (1C, d, *J*_CF_^4^ = 3 Hz), 138.02, 161.77, 164.27 (1C, d, *J*_CF_^1^ = 247 Hz), 169.44, 172.41; ^19^F NMR (DMSO-*d*_6_-376.25 MHz, *δ* ppm): −108.74; Anal. Calcd. For C_22_H_20_FN_5_S (405.50): C: 65.17; H: 4.97; N: 17.27. Found: C: 64.98; H: 5.13; N: 17.40.

#### 2-((4-Fluorobenzyl)thio)-4-(4-fluorophenyl)-6-(piperazin-1-yl) pyrimidine-5-carbonitrile (4f)

Yellow powder; yield: 64%; m.p. 120–122 °C; IR (KBr, cm^−1^): 3446 (NH), 3064 (C–H aromatic), 2918, 2850 (C–H aliphatic), 2208 (CN); ^1^H NMR (DMSO-*d*_6_-400 MHz, *δ* ppm): 2.72 (t, 4H, CH_2_–NH–CH_2_), 3.83 (t, 4H, CH_2_–N–CH_2_), 4.41 (s, 2H, S-CH_2_), 7.14 (t, 2H, ArH), 7.37 (t, 2H, *J* = 8.80 Hz, ArH), 7.46 (t, 2H, *J* = 8.00 Hz, ArH), 7.93 (dd, 2H, *J* = 9.20 Hz, *J* = 5.20 Hz, ArH); ^13^C NMR (DMSO-*d*_6_-100 MHz, *δ* ppm): 34.11, 45.99 (2C), 48.68 (2C), 84.01, 115.65 (2C, d, *J*_CF_^2^ = 21 Hz), 115.93 (2C, d, *J*_CF_^2^ = 22 Hz), 118.41, 128.41, 131.08 (2C, d, *J*_CF_^3^ = 8 Hz), 132.30 (2C, d, *J*_CF_^3^ = 9 Hz), 133.02 (1C, d, *J*_CF_^4^ = 3 Hz), 134.36 (1C, d, *J*_CF_^4^ = 3 Hz), 161.77 (1C, d, *J*_CF_^1^ = 242 Hz), 162.95, 163.06 (1C, d, *J*_CF_^1^ = 248 Hz), 169.46, 172.26; ^19^F NMR (DMSO-*d*_6_-376.25 MHz, *δ* ppm): −109.03, −115.47; MS (*m*/*z*): 338.87 (100%), 423.42 (M+, 12.50%); Anal. Calcd. For C_22_H_19_F_2_N_5_S (423.49): C: 62.40; H: 4.52; N: 16.54. Found: C: 62.31; H: 4.68; N: 16.81. HPLC purity = 92.35%.

#### 4-(4-Fluorophenyl)-2-((4-nitrobenzyl) thio)-6-(piperazin-1-yl) pyrimidine-5-carbonitrile (4g)

Buff powder; yield: 71%; m.p. 235–237 °C; IR (KBr, cm^−1^): 3446 (NH), 3078 (C–H aromatic), 2920,2850 (C–H aliphatic), 2208 (CN); ^1^H NMR (DMSO-*d*_6_-400 MHz, *δ* ppm): 2.79 (s, 4H, CH_2_–NH–CH_2_), 3.81 (s, 4H, CH_2_–NH–CH_2_), 4.55 (s, 2H, S-CH_2_), 7.38 (t, 2H, *J* = 8.00 Hz, ArH), 7.71 (d, 2H, *J* = 8.00 Hz, ArH), 7.90 (t, 2H, ArH), 8.18 (d, 2H, *J* = 8.00 Hz, ArH); ^13^C NMR (DMSO-*d*_6_-100 MHz, *δ* ppm): 34.46, 45.70 (2C), 48.31 (2C), 84.38, 115.97 (2C, d, *J*_CF_^2^ = 21 Hz), 118.31, 124.00 (2C), 130.30 (2C), 132.32 (2C, d, *J*_CF_^3^ = 9 Hz), 146.81, 146.96, 162.07, 164.31 (1C, d, *J*_CF_^1^ = 248 Hz), 169.34, 171.67; ^19^F NMR (DMSO-*d*_6_-376.25 MHz, *δ* ppm): −108.71; Anal. Calcd. For C_22_H_19_FN_6_O_2_S (450.49): C: 58.66; H: 4.25; N: 18.66. Found: C: 58.92; H: 4.37; N: 18.90.

#### 4-(4-Fluorophenyl)-2-((4-methylbenzyl)thio)-6-(piperazin-1-yl)pyrimidine-5-carbonitrile (4h)

White powder; yield: 62%; m.p. 250–252 °C; IR (KBr, cm^−1^): 3446 (NH), 3022 (C–H aromatic), 2918, 2850 (C–H aliphatic), 2206 (CN); ^1^H NMR (DMSO-*d*_6_-400 MHz, *δ* ppm): 2.25 (s, 3H, N–CH_3_), 3.39 (s, 4H, CH_2_–NH–CH_2_, overlapped), 4.15 (s, 4H, CH_2_–NH–CH_2_), 4.42 (s, 2H, S-CH_2_), 7.13 (d, 2H, *J* = 8.00 Hz, ArH), 7.31 (d, 2H, *J* = 8.00 Hz, ArH), 7.36–7.42 (m, 2H, *J* = 8.00 Hz, ArH), 7.94 (dd, 2H, *J* = 8.00 Hz, *J* = 4.00 Hz, ArH); ^13^C NMR (DMSO-*d*_6_-100 MHz, *δ* ppm): 21.12, 34.81, overlapped (2C), 46.06 (2C), 84.14, 116.02 (2C, d, *J*_CF_^2^ = 22 Hz), 118.10, 129.17 (2C), 129.53 (2C), 132.32 (2C, d, *J*_CF_^3^ = 9 Hz), 132.95 (1C, d, *J*_CF_^4^ = 3 Hz), 134.81, 136.84, 161.92, 164.29 (1C, d, *J*_CF_^1^ = 241 Hz), 169.19, 172.53; ^19^F NMR (DMSO-*d*_6_-376.25 MHz, *δ* ppm): −109.04; Anal. Calcd. For C_23_H_22_FN_5_S (419.52): C: 65.85; H: 5.29; N: 16.69. Found: C: 65.71; H: 5.48; N: 16.92.

#### 2-(Benzylthio)-4-(4-fluorophenyl)-6-(4-methylpiperazin-1-yl) pyrimidine-5-carbonitrile (4i)

Yellowish white crystals; yield:70%; m.p. 138 °C; IR (KBr, cm^−1^): 3026 (C–H aromatic), 2846, 2796 (C–H aliphatic), 2208 (CN); ^1^H NMR (DMSO-*d*_6_-400 MHz, *δ* ppm): 2.21 (s, 3H, N–CH_3_), 2.42 (s, 4H, CH_2_–NCH_3_–CH_2_), 3.91 (s, 4H, CH_2_–N–CH_2_), 4.42 (s, 2H, S-CH_2_), 7.24 (t, 1H, ArH), 7.30–7.42 (m, 6H, ArH), 7.91 (t, 2H, ArH); ^13^C NMR (DMSO-*d*_6_-100 MHz, *δ* ppm): 35.00, 45.83, 47.06 (2C), 54.68 (2C), 84.21, 115.93 (2C, d, *J*_CF_^2^ = 22 Hz), 118.35, 127.59, 128.92 (2C), 129.15 (2C), 132.31 (2C, d, *J*_CF_^3^ = 9 Hz), 132.92 (1C, d, *J*_CF_^4^ = 3 Hz), 137.96, 161.94, 164.30 (1C, d, *J*_CF_^4^ = 249 Hz), 169.40, 172.52; ^19^F NMR (DMSO-*d*_6_-376.25 MHz, *δ* ppm): −108.92; Anal. Calcd. For C_23_H_22_FN_5_S (419.52): C: 65.85; H: 5.29; N: 16.69. Found: C: 65.94; H: 5.43; N: 16.87.

#### 2-((4-Fluorobenzyl)thio)-4-(4-fluorophenyl)-6-(4-methylpiperazin-1-yl)pyrimidine-5-carbonitrile (4j)

White powder; yield: 75%; m.p. 165–167 °C; IR (KBr, cm^−1^): 3066 (C–H aromatic), 2846, 2796 (C–H aliphatic), 2210 (CN); ^1^H NMR (DMSO-*d*_6_-400 MHz, *δ* ppm): 2.22 (s, 3H, N–CH_3_), 2.44 (t, 4H, CH_2_–NCH_3_–CH_2_), 3.90 (t, 4H, CH_2_–N–CH_2_), 4.42 (s, 2H, S-CH_2_), 7.14 (t, 2H, ArH), 7.38 (t, 2H, ArH), 7.46 (dd, 2H, *J* = 8.00 Hz, *J* = 4.00 Hz, ArH), 7.92 (t, 2H, ArH); ^13^C NMR (DMSO-*d*_6_-100 MHz, *δ* ppm): 34.13, 45.84, 47.09 (2C), 54.69 (2C), 84.32, 115.56 (2C, d, *J*_CF_^2^ = 21 Hz), 115.96 (2C, d, *J*_CF_^2^ = 22 Hz), 118,33, 131.09 (2C, d, *J*_CF_^3^ = 8 Hz), 132.31 (2C, d, *J*_CF_^3^ = 9 Hz), 132.95 (1C, d, *J*_CF_^4^ = 3 Hz), 134.30 (1C, d, *J*_CF_^4^ = 3 Hz), 161.31 (1C, d, *J*_CF_^1^ = 243 Hz), 162.95, 164.31 (1C, d, *J*_CF_^1^ = 247 Hz), 169.43, 172.37; ^19^F NMR (DMSO-*d*_6_-376.25 MHz, *δ* ppm): −108.93, −115.46; Anal. Calcd. For C_23_H_21_F_2_N_5_S (437.51): C: 63.14; H: 4.84; N: 4.84. Found: C: 63.25; H: 5.02; N: 12.35.

#### 4-(4-Fluorophenyl)-6-(4-methylpiperazin-1-yl)-2-((4-nitrobenzyl)thio)pyrimidine-5-carbonitrile (4k)

Buff powder; yield: 67%; m.p. 178–180 °C; IR (KBr, cm^−1^): 3072 (C–H aromatic), 2987, 2850 (C–H aliphatic), 2210 (CN); ^1^H NMR (DMSO-*d*_6_-400 MHz, *δ* ppm): 2.21 (s, 3H, N–CH_3_), 2.41 (t, 4H, CH_2_–NCH_3_–CH_2_), 3.87 (t, 4H, CH_2_–N–CH_2_), 4.55 (s, 2H, S-CH_2_), 7.38 (t, 2H, ArH), 7.70 (d, 2H, *J* = 8.00 Hz, ArH), 7.90 (dd, 2H, *J* = 8.00 Hz, *J* = 4.00 Hz, ArH), 8.18 (d, 2H, ArH); ^13^C NMR (DMSO-*d*_6_-100 MHz, *δ* ppm): 34.26, 45.78, 47.06 (2C), 54.64 (2C), 84.53, 115.96 (2C, d, *J*_CF_^2^ = 22 Hz), 118.25, 124.00 (2C), 130.29 (2C), 132.33 (2C, d, *J*_CF_^3^ = 9 Hz), 132.84, 146.73, 146.97, 161.94, 164.33 (1C, d, *J*_CF_^1^ = 248 Hz), 169.49, 171.84; ^19^F NMR (DMSO-*d*_6_-376.25 MHz, *δ* ppm): −108.86; MS (*m*/*z*): 70.09 (100%), 464.98 (M^+^, 16.15%); Anal. Calcd. For C_23_H_21_FN_6_O_2_S (464.52): C: 59.47; H: 4.56; N: 18.09. Found: C: 59.73; H: 4.68; N: 17.91.

#### 4-(4-Fluorophenyl)-2-((4-methylbenzyl) thio)-6-(4-methylpiperazin-1-yl) pyrimidine-5-carbonitrile (4l)

Buff powder; yield: 63%; m.p. 150–152 °C; IR (KBr, cm^−1^): 3018 (C–H aromatic), 2854, 2796 (C–H aliphatic), 2206 (CN); ^1^H NMR (DMSO-*d*_6_-400 MHz, *δ* ppm): 2.25 (s, 6H, N–CH_3_, Ar-CH_3_), 2.51 (4H, CH_2_–NCH_3_–CH_2_, overlapped), 3.91 (s, 4H, CH_2_–N–CH_2_), 4.35 (s, 2H, S-CH_2_),7.12 (d, 2H, *J* = 8.00 Hz, ArH), 7.29 (d, 2H, *J* = 8.00 Hz, ArH), 7.38 (t, 2H, ArH), 7.92 (t, 2H, *J* = 8.00 Hz, ArH); ^13^C NMR (DMSO-*d*_6_-100 MHz, *δ* ppm): 21.10, 34.79, 45.63, 46.89 (2C), 54.54 (2C), 84.25, 115.95 (2C, d, *J*_CF_^2^ = 22 Hz), 118.34, 129.10 (2C), 129.48 (2C), 132.32 (2C, d, *J*_CF_^3^ = 9 Hz), 132.92 (1C, d, *J*_CF_^4^ = 3 Hz), 134.72, 136.82, 161.99, 164.31 (1C, d, *J*_CF_^1^ = 248 Hz), 169.36, 172.64; Anal. Calcd. For C_24_H_24_FN_5_S (433.55): C: 66.49; H: 5.58; N: 16.15. Found: C: 66.57; H: 5.74; N: 16.32.

#### 2-(Benzylthio)-4-(4-fluorophenyl)-6-(4-phenylpiperazin-1-yl) pyrimidine-5-carbonitrile (4m)

Yellow crystals; yield: 63%; m.p. 165–167 °C; IR (KBr, cm^−1^): 3061, 3030 (C–H aromatic), 2983, 2843 (C–H aliphatic), 2206 (CN); ^1^H NMR (DMSO-*d*_6_-400 MHz, *δ* ppm): 3.33 (4H, CH_2_–NCH_3_–CH_2_, overlapped), 4.07 (s, 4H, CH_2_–N–CH_2_), 4.44 (s, 2H, S-CH_2_), 6.81 (t, 2H, *J* = 8.00 Hz, ArH), 6.97 (d, 2H, *J* = 8.00 Hz, ArH), 7.18–7.27 (m, 2H, ArH), 7.31–7.45 (m, 6H, ArH), 7.93 (t, 2H, ArH); ^13^C NMR (DMSO-*d*_6_-100 MHz, *δ* ppm): 35.02, 46.91 (2C), 48.28 (2C), 84.32, 115.88 (2C, d, *J*_CF_^2^ = 20 Hz), 118.39, 119.65 (2C), 127.61 (2C), 128.96 (2C), 129.19 (2C), 129.37 (2C), 129.50 (2C), 132.32 (2C, d, *J*_CF_^3^ = 9 Hz), 138.00, 150.83, 163.16 (1C, d, *J*_CF_^1^ = 247 Hz), 169.40, 172.54; ^19^F NMR (DMSO-*d*_6_-376.25 MHz, *δ* ppm): −108.96; Anal. Calcd. For C_28_H_24_FN_5_S (481.59): C: 69.83; H: 5.02; N: 14.54. Found: C: 69.71; H: 5.28; N: 14.73.

#### 2-((4-Fluorobenzyl)thio)-4-(4-fluorophenyl)-6-(4-phenylpiperazin-1-yl)pyrimidine-5-carbonitrile (4n)

Yellowish white crystals; yield: 77%; m.p. 180–182 °C; IR (KBr, cm^−1^): 3059, 3003 (C–H aromatic), 2897, 2839 (C–H aliphatic), 2206 (CN); ^1^H NMR (DMSO-*d*_6_-400 MHz, *δ* ppm): 3.29 (4H, CH_2_–NCH_3_–CH_2_, overlapped), 4.04 (d, 4H, CH_2_–N–CH_2_), 4.45 (s, 2H, S-CH_2_), 6.77–6.83 (m, 1H, ArH), 6.95 (t, 3H, ArH), 7.10–7.18 (m, 2H, ArH), 7.20–7.26 (m, 2H, ArH), 7.34–7.41 (m, 2H, ArH), 7.44–7.50 (m, 2H, ArH), 7.92 (t, 2H, ArH); ^13^C NMR (DMSO-*d*_6_-100 MHz, *δ* ppm): 34.19, 46.91 (2C), 48.26 (2C), 84.34, 115.67 (2C, d, *J*_CF_^2^ = 21 Hz), 115.96 (2C, d, *J*_CF_^2^ = 22 Hz), 118.37, 119.65 (2C), 129.48 (2C), 131.09 (2C, d, *J*_CF_^3^ = 8 Hz), 132.31 (2C, d, *J*_CF_^3^ = 9 Hz), 132.95 (1C, d, *J*_CF_^4^ = 3 Hz), 134.30 (1C, d, *J*_CF_^4^ = 3 Hz), 150.80, 161.74 (1C, d, *J*_CF_^1^ = 242 Hz), 161.93, 162.97, 164.31 (1C, d, *J*_CF_^1^ = 249 Hz), 169.39, 172.42; ^19^F NMR (DMSO-*d*_6_-376.25 MHz, *δ* ppm): −108.88, −115.41; Anal. Calcd. For C_28_H_23_F_2_N_5_S (499.58): C: 67.32; H: 4.64; N: 14.02. Found: C: 67.54; H: 4.73; N: 14.29.

#### 4-(4-Fluorophenyl)-2-((4-nitrobenzyl)thio)-6-(4-phenylpiperazin-1-yl)pyrimidine-5-carbonitrile (4o)

Golden yellow crystals; yield: 75%; m.p. 165–167 °C; IR (KBr, cm^−1^): 3068, 3028 (C–H aromatic), 2883, 2850 (C–H aliphatic), 2208 (CN); ^1^H NMR (DMSO-*d*_6_-400 MHz, *δ* ppm): 3.30 (4H, CH_2_–NCH_3_–CH_2_, overlapped), 4.05 (s, 4H, CH_2_–N–CH_2_), 4.57 (s, 2H, S-CH_2_), 6.81 (t, 1H, ArH), 6.94 (d, 2H, *J* = 8.00 Hz, ArH), 7.23 (t, 2H, ArH), 7.39 (t, 2H, ArH), 7.73 (d, 2H, *J* = 8.00 Hz, ArH), 7.92 (t, 2H, ArH), 8.20 (d, 2H, *J* = 8.00 Hz, ArH); ^13^C NMR (DMSO-*d*_6_-100 MHz, *δ* ppm): 34.31, 46.92 (2C), 48.22 (2C), 84.51, 115.80, 115.97 (2C, d, *J*_CF_^2^ = 21 Hz), 118.28, 119.66 (2C), 124.02 (2C), 129.47 (2C), 130.26 (2C), 132.30 (2C, d, *J*_CF_^3^ = 9 Hz), 132.82 (1C, d, *J*_CF_^4^ = 3 Hz), 146.73, 146.94, 150.77, 161.90, 164.33 (1C, d, *J*_CF_^1^ = 248 Hz), 169.48, 171.88; ^19^F NMR (DMSO-*d*_6_-376.25 MHz, *δ* ppm): −108.84; Anal. Calcd. For C_28_H_23_FN_6_O_2_S (526.59): C: 63.87; H: 4.40; N: 15.96. Found: C: 63.59; H: 4.61; N: 16.20.

#### 4-(4-Fluorophenyl)-2-((4-methylbenzyl) thio)-6-(4-phenylpiperazin-1-yl) pyrimidine-5-carbonitrile (4p)

Light brown powder; yield: 78%; m.p. 145–147 °C; IR (KBr, cm^−1^): 3062, 3024 (C–H aromatic), 2904, 2833 (C–H aliphatic), 2200 (CN); ^1^H NMR (DMSO-*d*_6_-400 MHz, *δ* ppm): 2.26 (s, 3H, CH_3_), 3.29 (s, 4H, CH_2_–NCH_3_–CH_2_, overlapped), 4.07 (s, 4H, CH_2_–N–CH_2_), 4.41 (s, 2H, S-CH_2_), 6.81 (t, 1H, ArH), 6.96 (d, 2H, *J* = 8.00 Hz, ArH), 7.13 (d, 2H, *J* = 8.00 Hz, ArH), 7.24 (t, 2H, *J* = 8.00 Hz, ArH), 7.32 (d, 2H, *J* = 8.00 Hz, ArH), 7.38 (t, 2H, *J* = 8.00 Hz, ArH), 7.94 (t, 2H, ArH); ^13^C NMR (DMSO-*d*_6_-100 MHz, *δ* ppm): 21.11, 34.83, 46.91 (2C), 48.28 (2C), 84.24, 115.96 (2C, d, *J*_CF_^2^ = 20 Hz), 118.39 (2C), 119.66 (2C), 129.10 (2C), 129.49 (2C), 129.51 (2C), 132.30 (2C, d, *J*_CF_^3^ = 9 Hz), 132.95 (1C, d, *J*_CF_^4^ = 3 Hz), 134.73, 136.82, 150.83, 161.92, 164.31 (1C, d, *J*_CF_^1^ = 248 Hz), 169.36, 172.65; ^19^F NMR (DMSO-*d*_6_-376.25 MHz, *δ* ppm): −108.93; MS (*m*/*z*): 198.63 (100%), 494.56 (M^+^, 36.20%); Anal. Calcd. For C_29_H_26_FN_5_S (495.60): C: 70.28; H: 5.29; N: 14.13. Found: C: 70.54; H: 5.64; N: 14.30.

## Biological activity

### *In vitro* cytotoxic screening

The preliminary *in vitro* antitumor investigation of our novel potential anticancer candidates was carried out by NCI, MD, USA (www.dtp.nci.nih.gov). All new compounds 2b–d, 3a–d and 4a–p were tested against NCI60 cell lines covering different types of cancer. Results of anticancer screening assay was recorded for each compound as growth inhibition percent (GI%). Two members of the new series 4e & 4f disclosed potent anticancer activity against all tested cell line, as a result 4e & 4f have been undergo further anticancer screening in five-dose assay [0.01, 0.1, 1, 10, and 100 μM] by NCI. Moreover, the safety profile of 4e & 4f at normal cells were evaluated [6, 7].

### *In vitro* EGFR^WT^ inhibitory assay

Using BPS Bioscience EGFR Kinase Assay Kit (Catalog #:40 321), the most potent anticancer compounds 4e and 4f inhibitory activity against EGFR^WT^ was evaluated by following the manufacturer instructions. (1) Prepare the mixture of N wells × (kinase assay buffer 6 μl, ATP1 μl, PTK substrate 1 μl and water 17 μl). to every well pipette the 25 μl. (2) Add 5 μl of compounds 4e & 4f, separately to wells signed as “Test”. On the contrary, add 5  μl of inhibitor buffer to control and plank wells. (3) Pipette 20 μl of EGFR^WT^ to test and control wells, on the blank wells pipette 20 μl kinase assay buffer. Then incubate at R.T for 40 min. (4) end the incubation after 40 min by adding to every well 50 μl of Kinase-Glo Max reagent, and incubate 15 min. By measuring the ATP quantity after kinase reaction, the activity of kinase could be detected. (5) use microplate reader to measure the luminescence.^51^

### *In vitro* COX-2 inhibitory assay

The inhibitory activity of novel 4e & 4f against COX-1/COX-2 was evaluated by using Biovision COX-1/COX-2 Inhibitor Screening Kit (Catalog #: K548) and (Catalog #: K547), respectively. Setting indomethacin and celecoxib as standards. Cycloxygenase interacts with its substrate (arachidonic acids) to produce prostaglandin G2, which binds with specific probe provided with the kit, that results in fluorescence which could be detected at *λ*_Ex_ = 535 nm/*λ*_Em_ = 587 nm in R.T for 5 min.^52^

### Cell cycle analysis and apoptosis

Cell Culture Unit, VACSERA, Cairo, Egypt, conducted an apoptosis investigation. These tests were conducted on the most potent compounds, 4e & 4f, to examine their cytotoxicity in the Colo 205 cell line, which is the most sensitive cell line.

### Cell cycle analysis

Flow cytometric analysis was used to examine the impact of the most potent compounds 5a and 5b on cell cycle progression. Results reported as DNA% of each cell cycle phase: (S, G1, G2, M). The following reported procedure was followed; (1) applying compounds 4e and 4f on Colo 205 cell line separately, then the Colo 205 was cultured. (2) The cells were collected in PBS diluted by nanopure water as a single cell suspension (by adding trypsine to dissociate cells). (3) Centrifuge cells by 500×*g* for 5 min, decant the supernatant. (4) Using PBS wash the cells, gently tapping the tube to resuspend cells. (5) In ice bath fix the cells in 66% cooled ethanol, stored in +4 °C 2 h at least (6) put the sample on bench at room temperature, inverted tube gently to resuspend cells. (7) Centrifuge, discard supernatant, wash and resuspend cells once more time. (8) Gently adding PI solution with RNAase (preventing RNA staining and interfering results), (9) Incubate samples in R.T, dark conditions for 20 min (10) transfer samples into ice, dark conditions. (11) Genteelly tapping to resuspend cells which settled during incubation step. (12) Put sample into flow cytometer and run it. Detect the fluorescence of protium iodide in FL2.^41,42^ Additional assessment of the pro-apoptotic effect was performed by using Annexin V-FITC and propidium iodide.

### Annexin V-FITC apoptosis assay

Detection of apoptosis by using BioVision Annexin V-FITC kits (Catalog #: K101-25) takes just 15 min. the Colo 205 cell line was treated by our most potent candidates 4e and 4f, separately. Centrifuge to collect cells, using binding buffer to resuspend cells. Pipette 5 μl of Annexine-V and 5 μl. Subsequently, incubate sample for 5 min. In R.T, dark condition. Detect Annexine V-FITC binding cells detected by FACSCaliber flow cytometer (*E*_x_ = 488 nm; *E*_m_ = 530 nm) which indicate apoptotic cells. On the other side the PI stained cells detected by FL2 flow cytometer, which detect necrotic cells.^53,54^

### Caspase-3 enzyme assay

Using an Invetrogen (Catalog #: KH 01091) ELISA kit, the level of human active caspase-3 was evaluated. (1) Into the antibody coated wells add 100 μl of standard diluent buffer excepts wells wich detected to be the chromogen blank, then add 100 μl. (2) Pipette 100 μl of Hu caspase-3 (active) standard or control or the sample of protein extracted from most sensitive cell line after treated with the most potent members 4e, and 4f (3) use plate cover to cover wells then incubate 2 h in R.T (4) using wash buffer to wash wells then aspirate liquid, (5) add 100 μl of caspase-3 (active) detection antibody solution into all wells except the chromogen plank wells, (6) using plate cover to cover wells plate then incubate 1 h in R.T, (7) using wash buffer to wash wells then discard the liquid, (8) pipette 100 μl Anti-Rabbit IgG HRP Solution to all well except the chromogen blank, (9) cover plates and incubate in R.T for 30 min. (10) Decant liquid from wells then wash, (11) pipette 100 μl of chromogen substrate to all wells, The well fluid will start to become blue. (12) 30 minutes should be spent incubating at room temperature in the dark. (13) To each well, add 100 μl of stop solution. To mix, lightly tap the plate's edge. The well solution should transform from blue to yellow. (14) Read the absorbance of each well using a microtiter plate reader at 450 nm. (15) Draw the standard curve using a curve fitting program. (16) The standard curve can be used to determine the concentrations for unknown samples and controls.^55^

### Molecular docking study

A molecular docking analysis was conducted using the reported methodology^47^ using MOE software program (MOE 2014.0901). The EGFR^WT^/COX-2 crystal structure (PDB code: 1M17, and 3LN1), respectively, was taken from the protein data bank. Using the Molecular Operating Environment (MOE 2014.0901) program, all molecular docking simulations and docking investigations into the active site of 1M17/3LN1 of compound 4e & 4f were performed. With an MMFF94X forced field, energy minimizations were carried out with an RMSD gradient of 0.05 kcal mol^−1^-1Energy minimizations were done using a 0.05 kcal mol^−1^ RMSD gradient, and calculation of partial charges was done. The following process was applied; preparation of the target protein 1M17/3LN1; water molecules were deleted, repeated chains were deleted, unnecessary ligand were deleted, then hydrogen atoms were added to protein, hydrogen atoms were hided, MOE site finder wizard was used for detection of the active pocket, the 3D structures of the ligands created using Chemdraw professional, then saved as mol file, all newly synthesized compounds were involved in one MOE database then docked into the target 1M17/3LN1.

### D pharmacophore mapping

Alignment of the most potent compound 4e and EGFR^WT^/COX-2 standard inhibitors; (erlotinib, and celecoxib), where performed by MOE v 2014.0901. Using flexible alignment function in MOE, subsequently, pharmacophore query was builded, using consensus tool in pharmacophore query, the shared features were detected.

### Physicochemical parameter

Physicochemical parameters (drug likeness) were predicted and calculated using SwissADME online tool.

## Conflicts of interest

The authors declare that they have no known competing financial interests or personal relationships that could have appeared to influence the work reported in this paper.

## Supplementary Material

RA-013-D3RA06088H-s001

RA-013-D3RA06088H-s002
