# Supplementary material for: Design, synthesis, and biological evaluation of new pyrimidine-5-carbonitrile derivatives as novel anti-cancer, dual EGFRWT/COX-2 inhibitors with docking studies
Source: RSC Adv. 2023 Nov 2;13(46):32296–320. doi: 10.1039/d3ra06088h (PMC10620772; doi:10.1039/d3ra06088h)
Supplement: RA-013-D3RA06088H-s001 [file RA-013-D3RA06088H-s001.pdf]

Electronic Supplementary Material (ESI) for RSC Advances.
This journal is © The Royal Society of Chemistry 2023

Design, Synthesis, and Biological Evaluation of New Pyrimidine-5-carbonitrile Derivatives as Novel Anti-cancer, Dual EGFR^{WT}/COX-2 Inhibitors with Docking Studies.

Nada Reda[a,*], Ahmed Elshewy[b,c], Hesham I. EL-Askarye, Khaled O. Mohamed[b], Amira A. Helwa[a]

Spectral Data

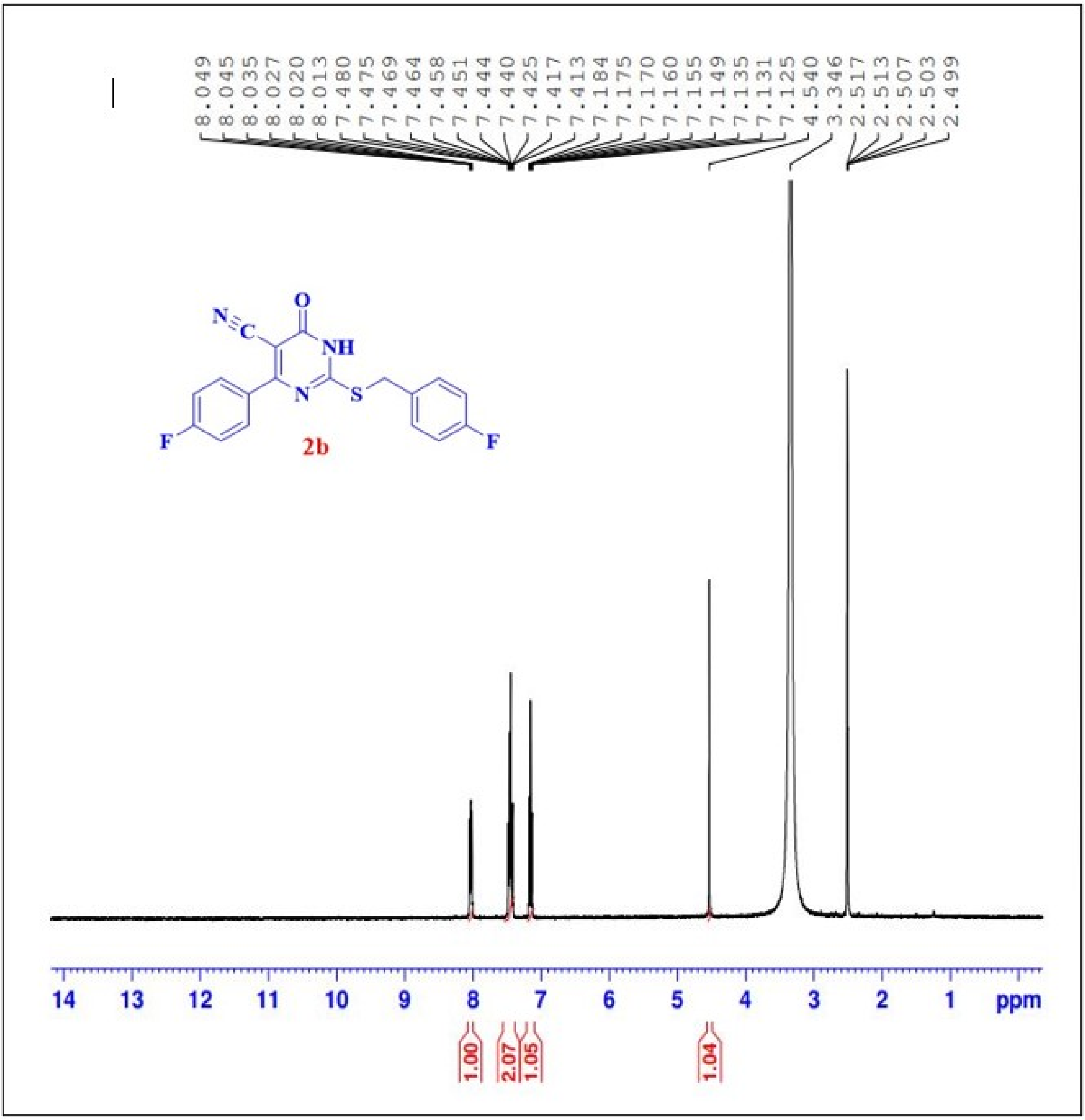

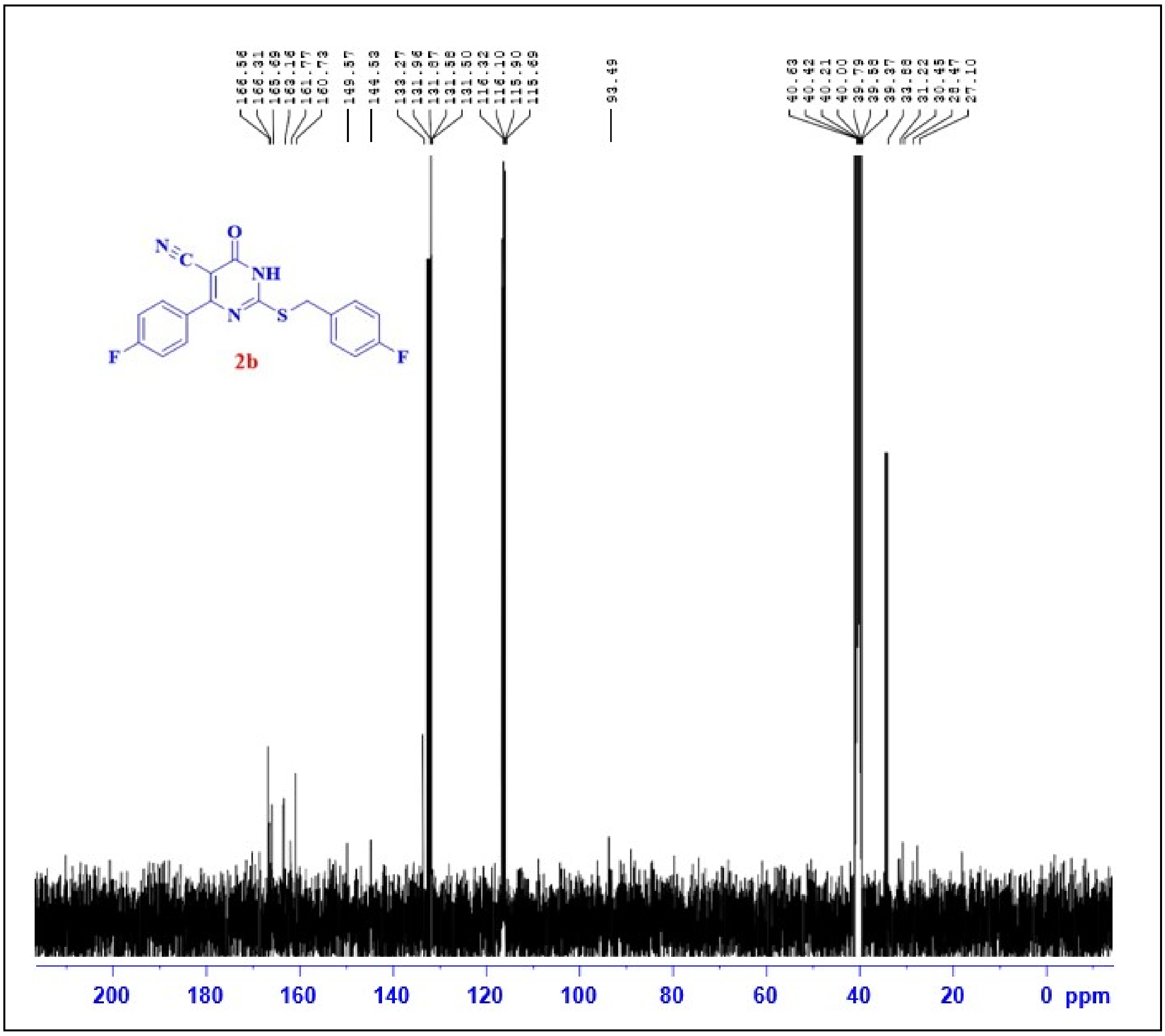
166.56
166.31
165.69
163.16
161.77
160.73
149.57
144.53
133.27
131.96
131.87
131.58
131.50
116.32
116.10
115.90
115.69
93.49
40.63
40.42
40.21
40.00
39.79
39.58
39.37
33.88
31.22
30.45
28.47
27.10
N
C
O
NH
N
S
F
F
2b
200
180
160
140
120
100
80
60
40
20
0
ppm

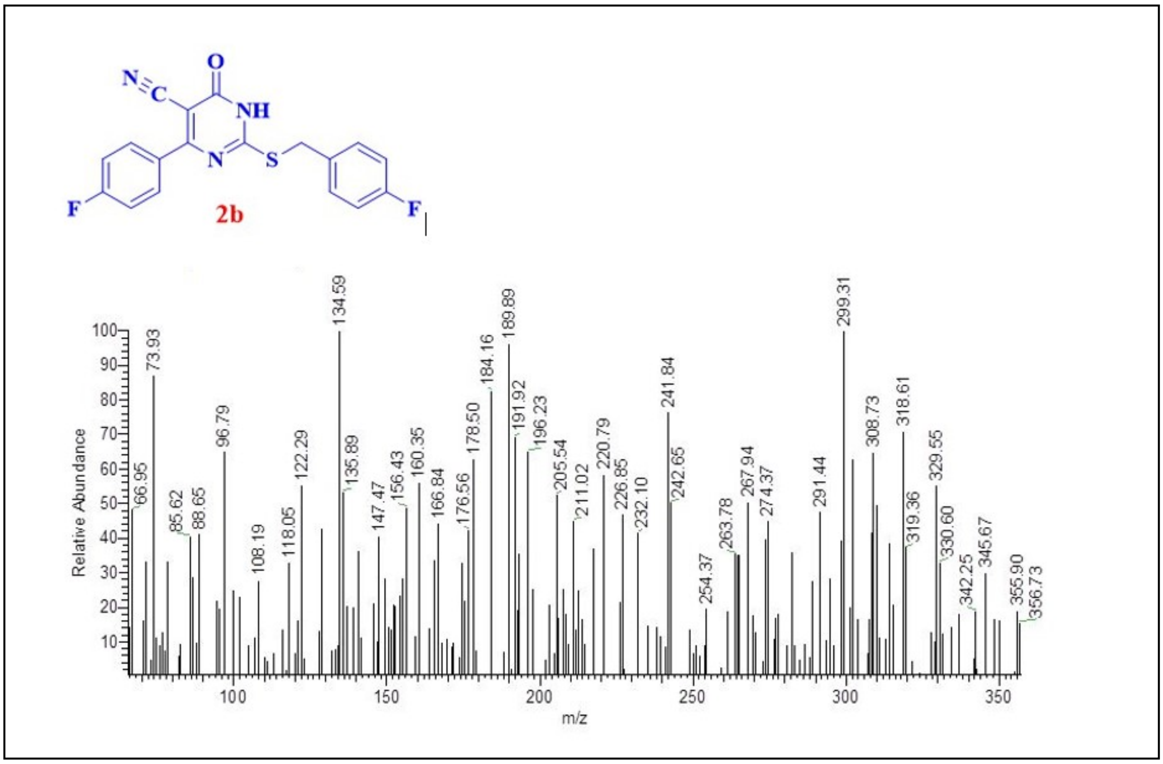

N
C
O
NH
N
S
F
F
2b
Relative Abundance
m/z
0
10
20
30
40
50
60
70
80
90
100
100
150
200
250
300
350
66.95
73.93
85.62
88.65
96.79
108.19
118.05
122.29
134.59
135.89
147.47
156.43
160.35
166.84
176.56
178.50
184.16
189.89
191.92
196.23
205.54
211.02
220.79
226.85
232.10
241.84
242.65
254.37
263.78
267.94
274.37
291.44
299.31
308.73
318.61
319.36
329.55
330.60
342.25
345.67
355.90
356.73

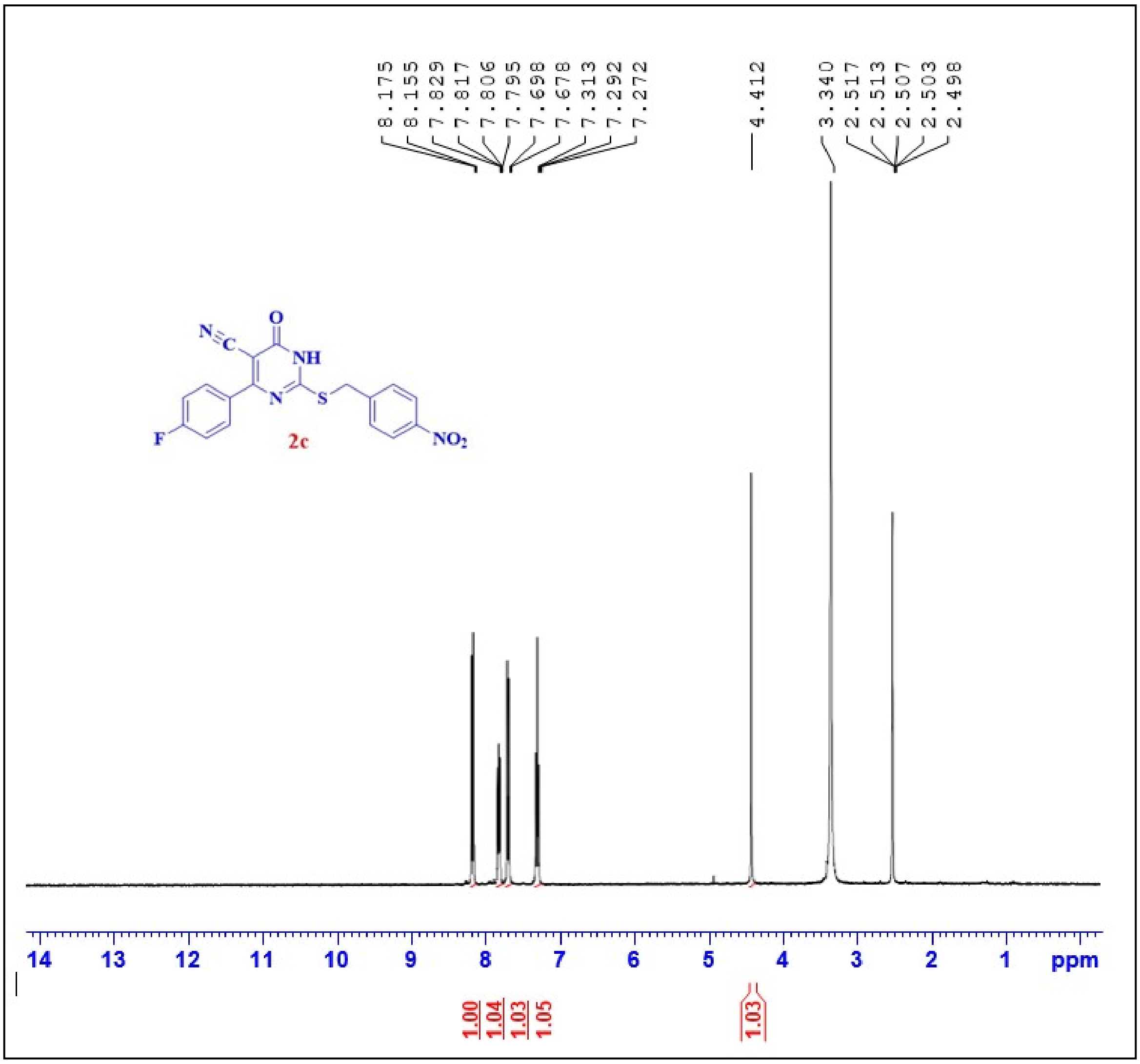

8.175
8.155
7.829
7.817
7.806
7.795
7.698
7.678
7.313
7.292
7.272
4.412
3.340
2.517
2.513
2.507
2.503
2.498
N
C
O
NH
F
N
S
NO2
2c
14
13
12
11
10
9
8
7
6
5
4
3
2
1
ppm
1.00
1.04
1.03
1.05
1.03

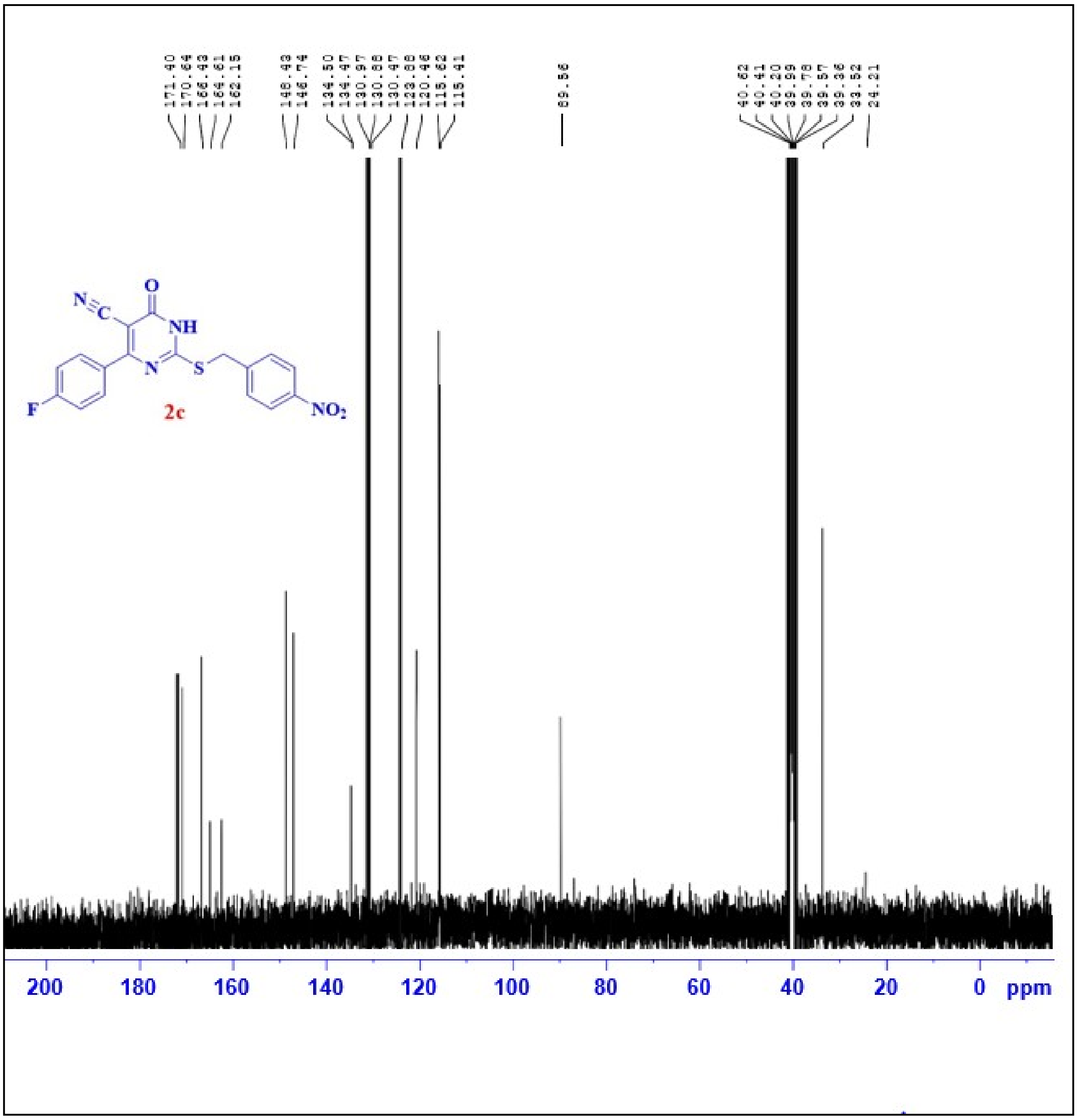
171.40
170.64
166.43
164.61
162.15
148.43
146.74
134.50
134.47
130.97
130.88
130.47
123.88
120.46
115.62
115.41
89.56
40.62
40.41
40.20
39.99
39.78
39.57
39.36
33.52
24.21
N
C
O
NH
N
S
F
NO2
2c
200
180
160
140
120
100
80
60
40
20
0
ppm

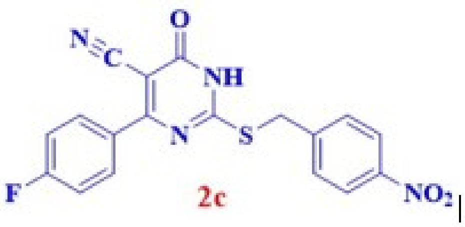
N
C
O
NH
N
S
F
NO2
2c

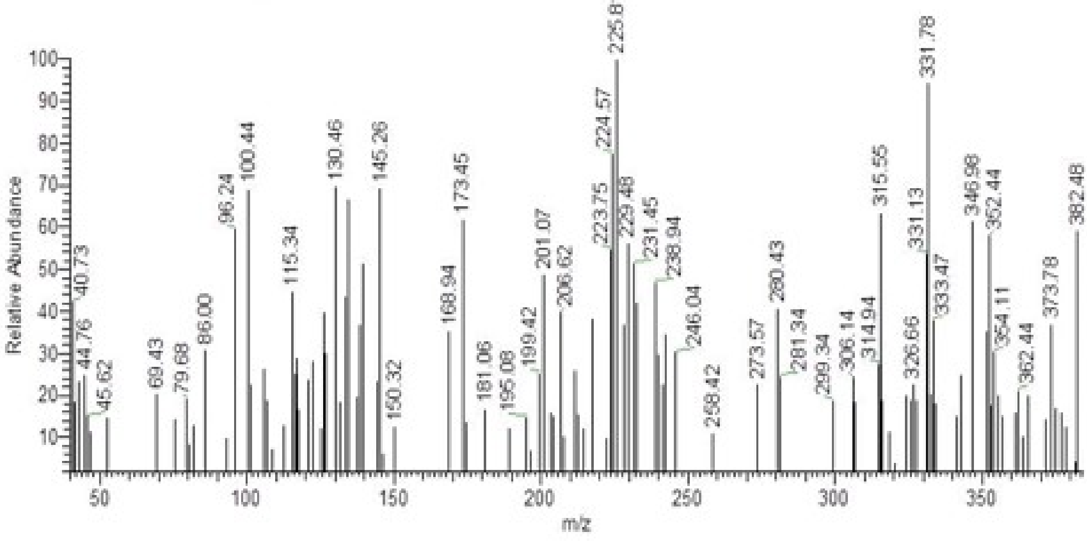
Relative Abundance
m/z
225.81
331.78
224.57
130.46
145.26
100.44
173.45
315.55
346.98
362.44
382.48
96.24
223.75
229.48
231.45
238.94
201.07
206.62
331.13
333.47
115.34
40.73
280.43
168.94
86.00
246.04
199.42
181.06
195.08
44.76
45.62
69.43
79.68
150.32
258.42
273.57
281.34
299.34
306.14
314.94
326.66
354.11
362.44
373.78

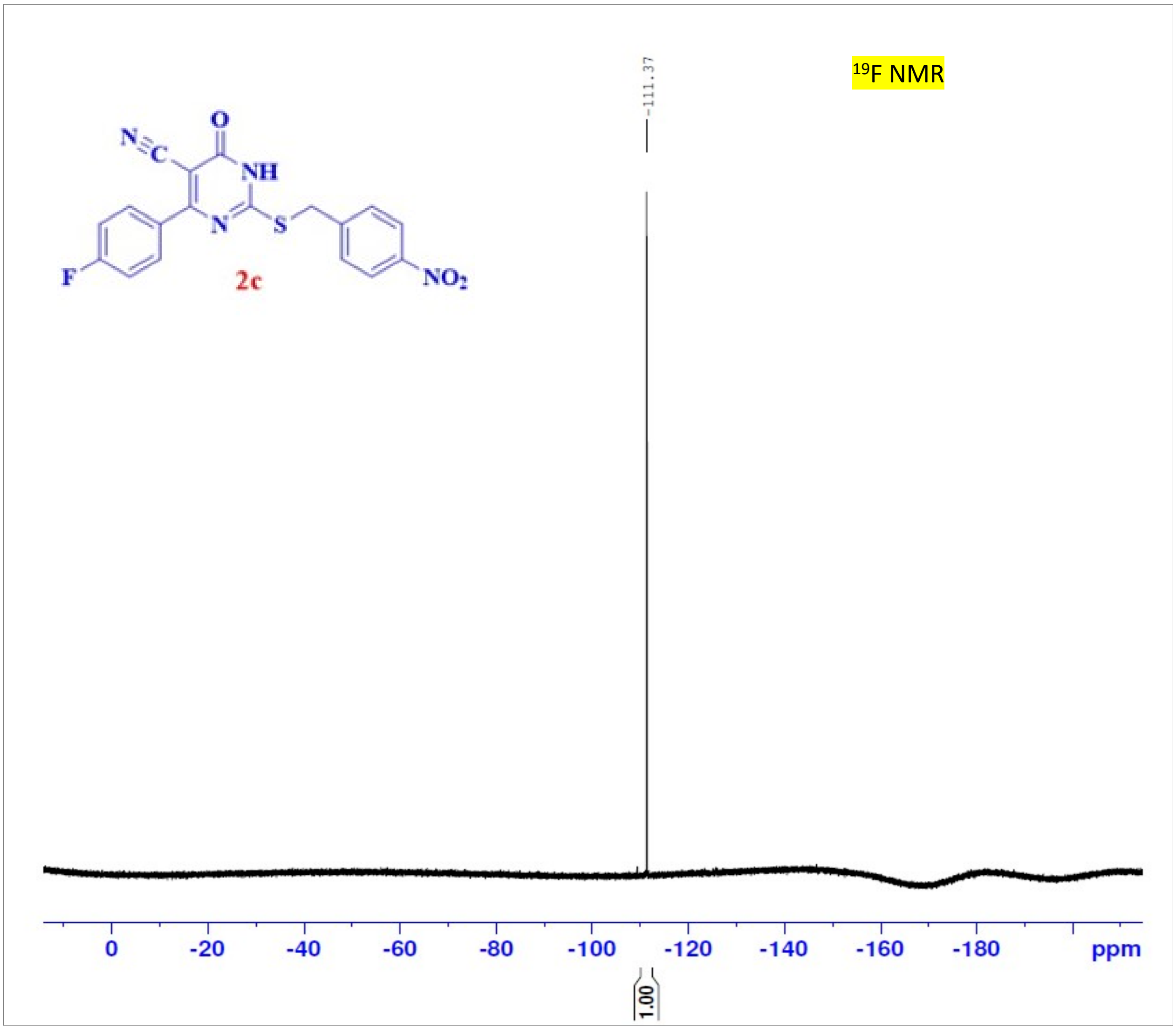
19F NMR
-111.37
N
C
O
NH
N
S
F
2c
NO2
0
-20
-40
-60
-80
-100
-120
-140
-160
-180
ppm
1.00

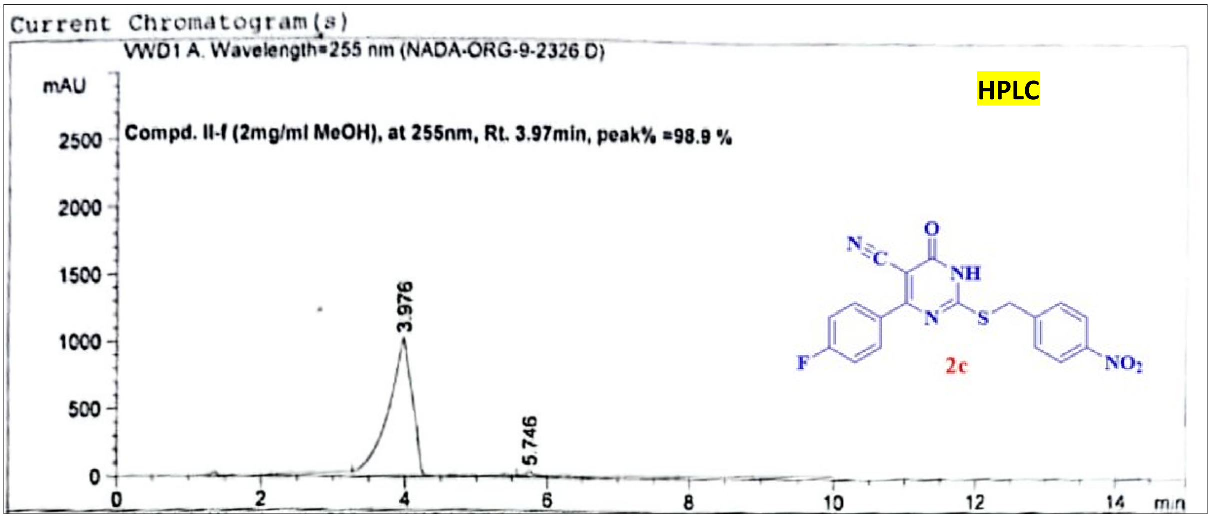
Current Chromatogram(s)
VWD1 A, Wavelength=255 nm (NADA-ORG-9-2326 D)
HPLC
mAU
Compd. II-f (2mg/ml MeOH), at 255nm, Rt. 3.97min, peak% =98.9 %
2500
2000
1500
1000
500
0
3.976
5.746
0
2
4
6
8
10
12
14
min
2c
NO2
F

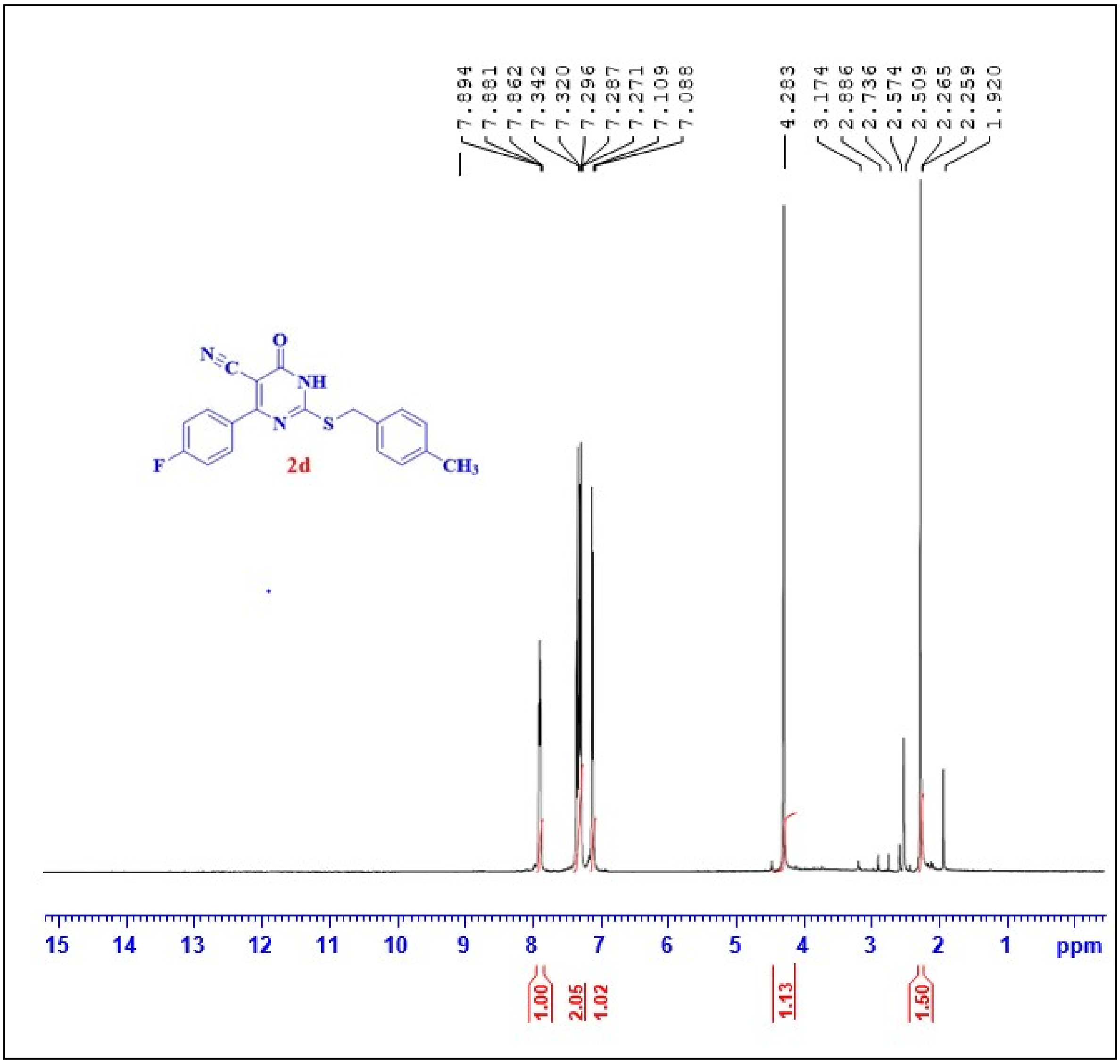
7.894
7.881
7.862
7.342
7.320
7.296
7.287
7.271
7.109
7.088
4.283
3.174
2.886
2.736
2.574
2.509
2.265
2.259
1.920
N
C
O
NH
N
S
F
CH3
2d
15
14
13
12
11
10
9
8
7
6
5
4
3
2
1
ppm
1.00
2.05
1.02
1.13
1.50

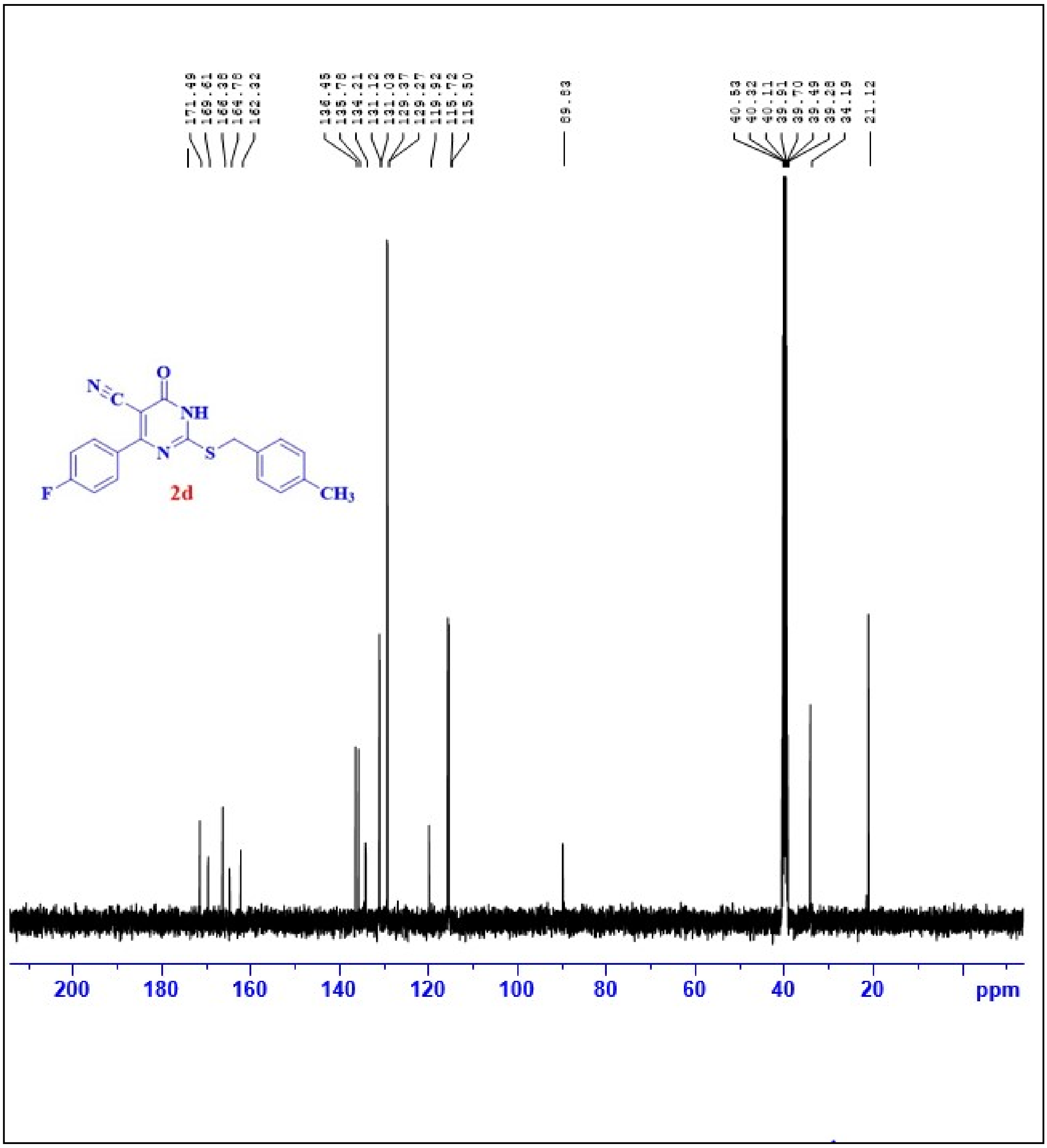

171.49
169.61
166.38
164.78
162.32
136.45
135.78
134.21
131.12
131.03
129.37
129.27
119.92
115.72
115.50
89.83
40.53
40.32
40.11
39.91
39.70
39.49
39.28
34.19
21.12
N
C
O
NH
N
S
F
CH3
2d
200
180
160
140
120
100
80
60
40
20
ppm

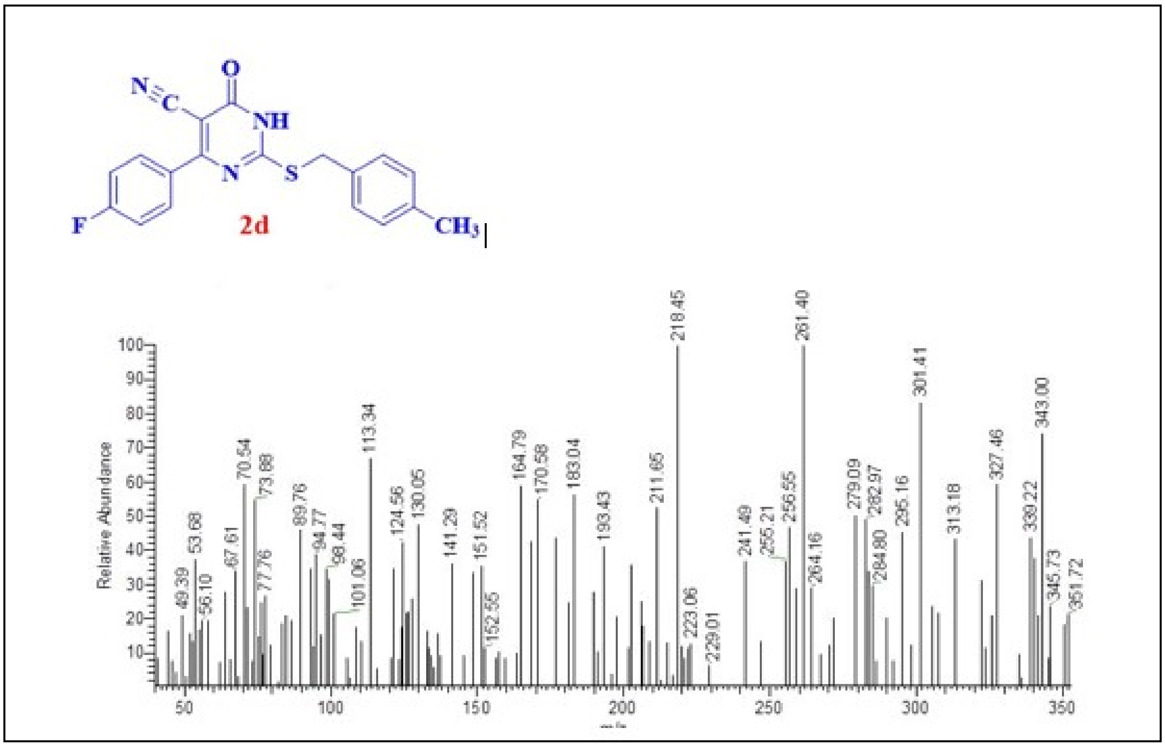
2d
Relative Abundance
218.45
261.40
301.41
343.00
113.34
327.46

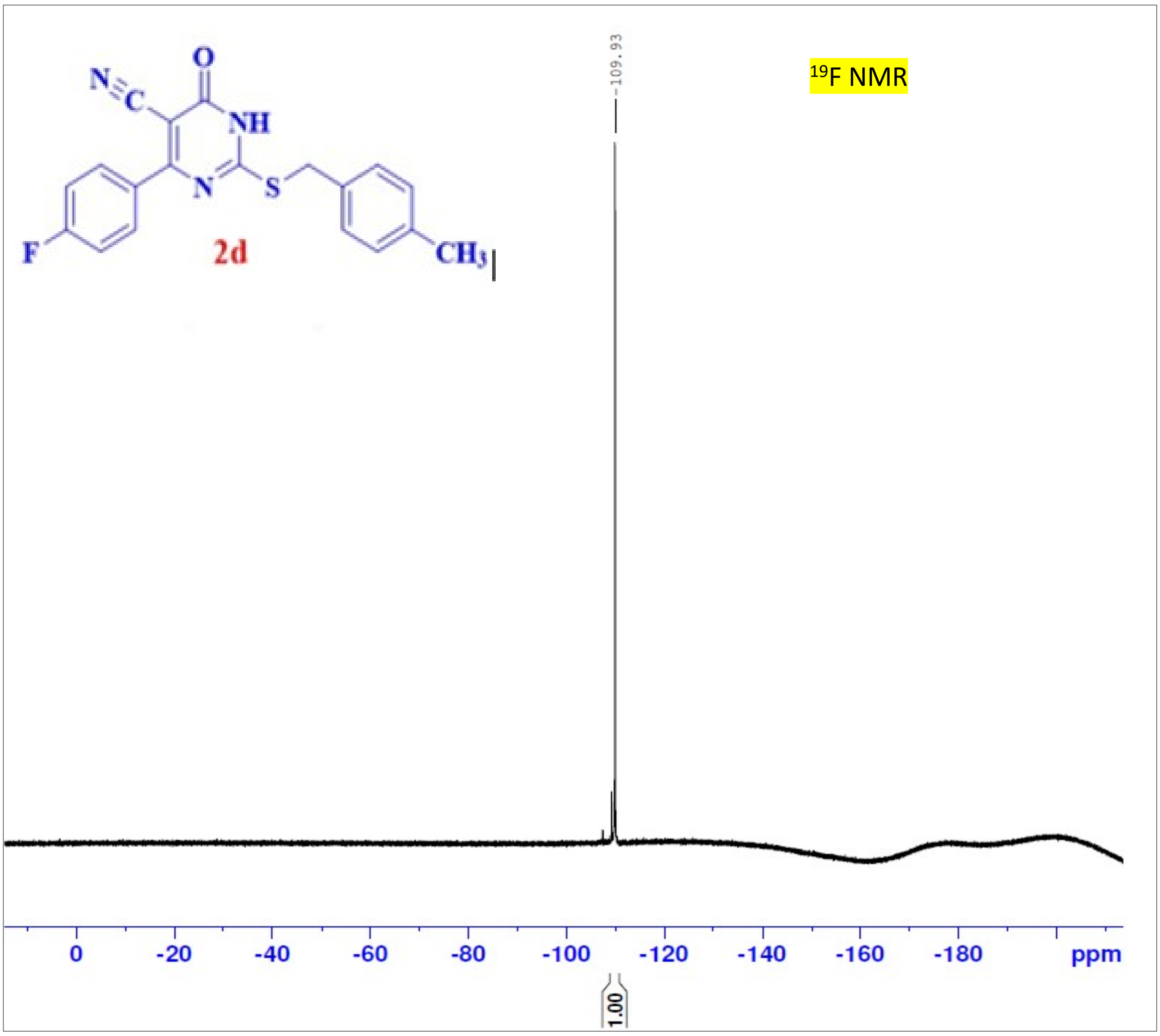
2d
19F NMR
-109.93
ppm
1.00

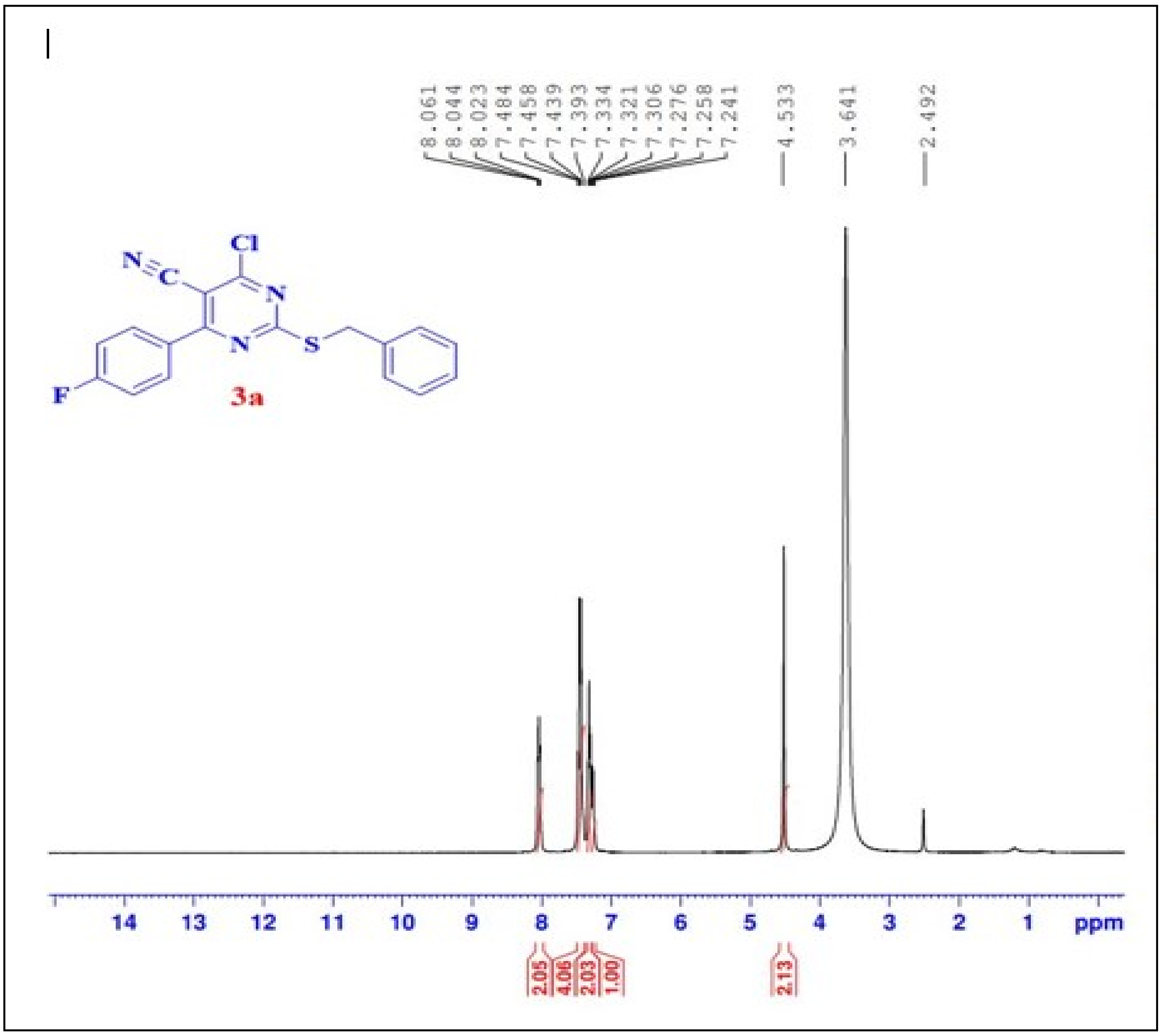
8.061
8.044
8.023
7.484
7.458
7.439
7.393
7.334
7.321
7.306
7.276
7.258
7.241
4.533
3.641
2.492
N
C
Cl
N
N
S
F
3a
14
13
12
11
10
9
8
7
6
5
4
3
2
1
ppm
2.05
4.06
2.03
1.00
2.13

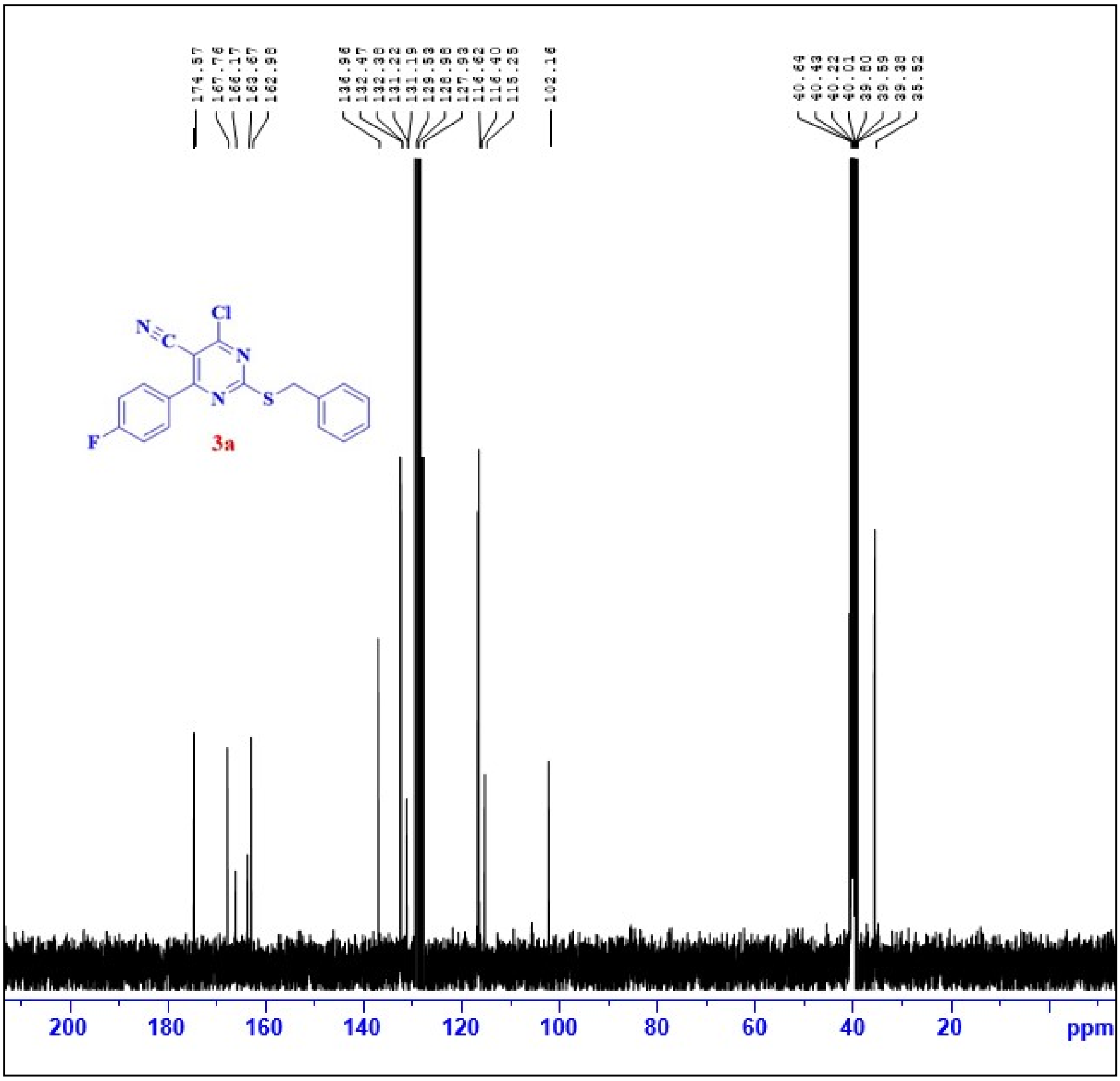

174.57
167.76
166.17
163.67
162.98
136.96
132.47
132.38
131.22
131.19
129.53
128.98
127.93
116.62
116.40
115.25
102.16
40.64
40.43
40.22
40.01
39.80
39.59
39.38
35.52
Cl
N
C
N
N
S
F
3a
200
180
160
140
120
100
80
60
40
20
ppm

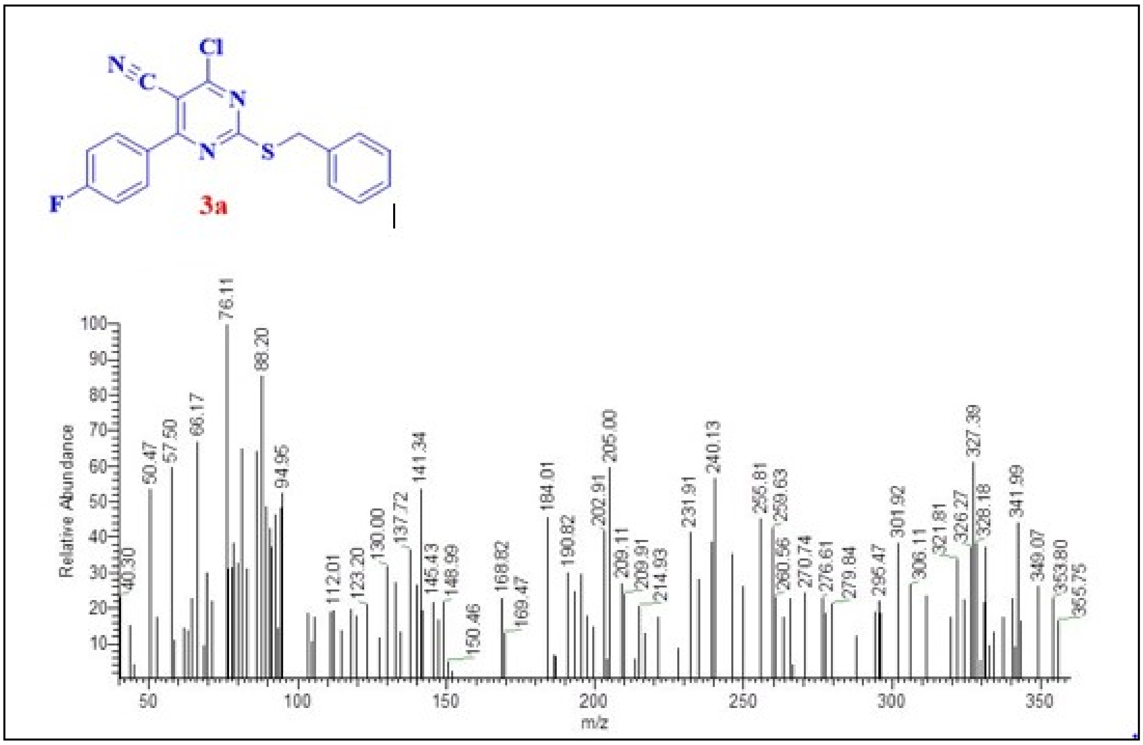

Cl
N
C
N
N
S
F
3a
Relative Abundance
m/z
76.11
88.20
66.17
57.50
50.47
94.95
40.30
112.01
123.20
130.00
137.72
141.34
145.43
148.99
150.46
168.82
169.47
184.01
190.82
202.91
205.00
209.11
209.91
214.93
231.91
240.13
255.81
259.63
260.56
270.74
276.61
279.84
295.47
301.92
306.11
321.81
326.27
327.39
328.18
341.99
349.07
353.80
355.75

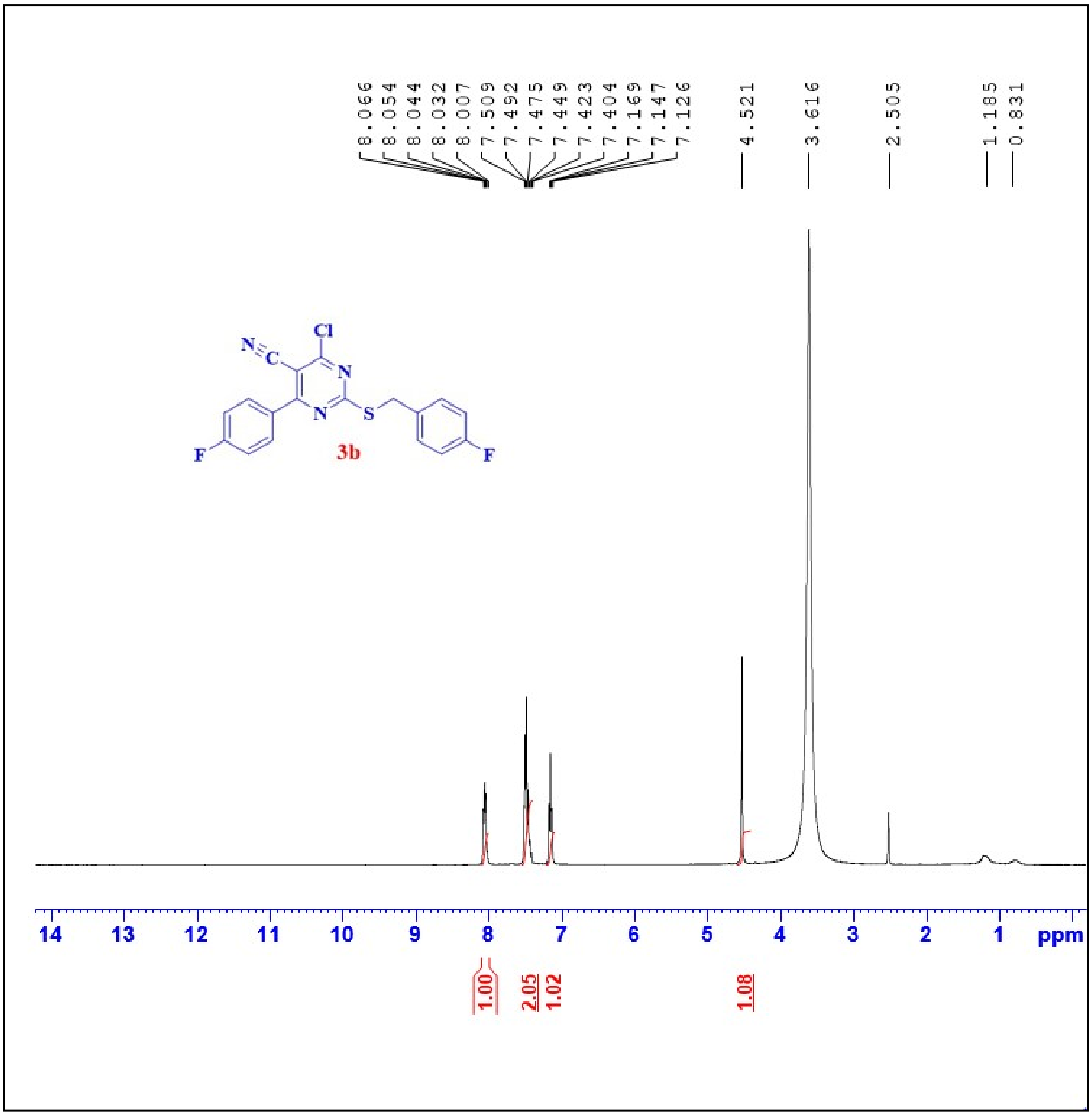
8.066
8.054
8.044
8.032
8.007
7.509
7.492
7.475
7.449
7.423
7.404
7.169
7.147
7.126
4.521
3.616
2.505
1.185
0.831
Cl
N
C
N
N
S
F
F
3b
14 13 12 11 10 9 8 7 6 5 4 3 2 1 ppm
1.00
2.05
1.02
1.08

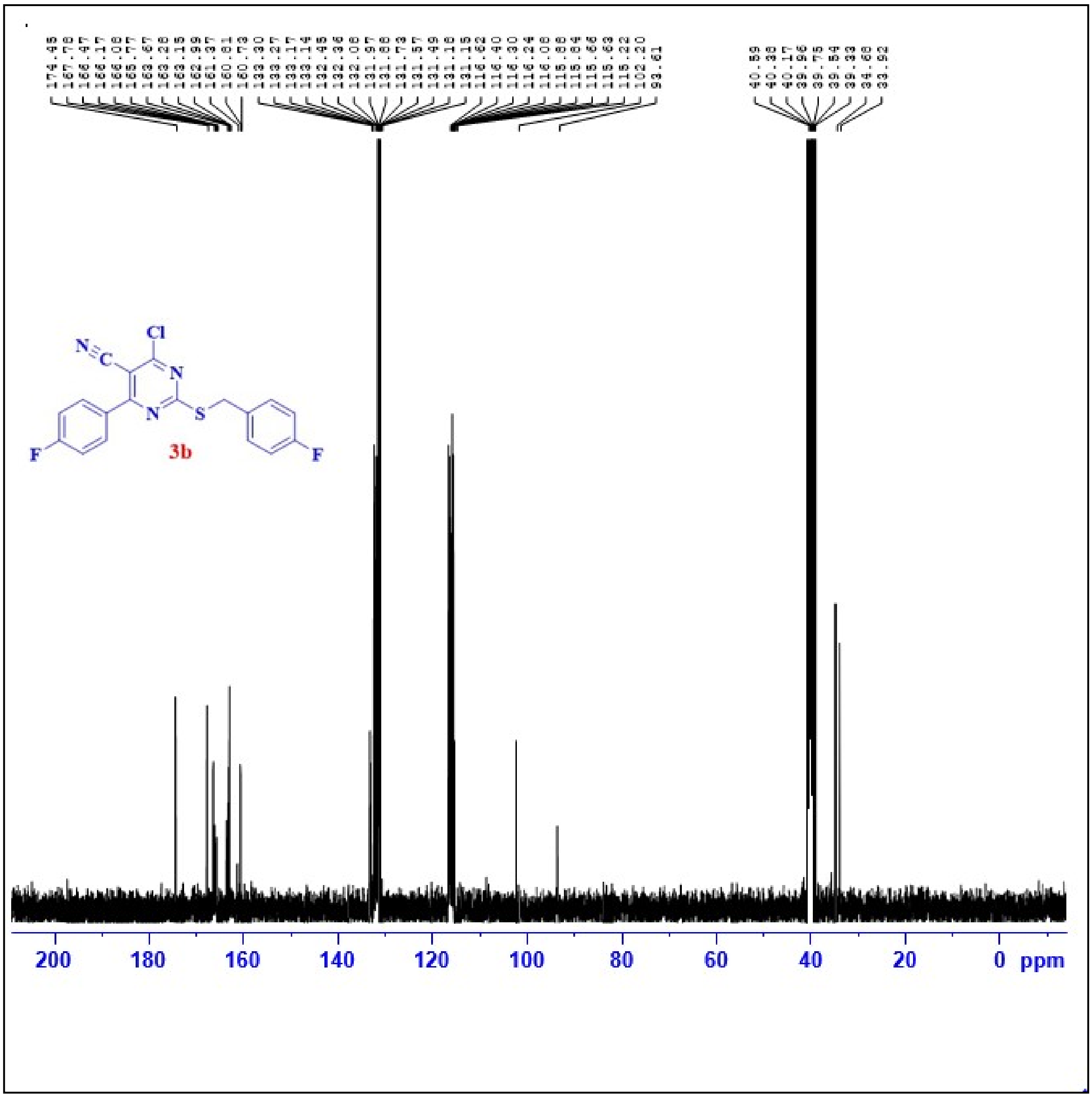
174.45
167.78
166.47
166.17
166.08
165.77
163.67
163.28
163.15
162.99
161.37
160.81
160.73
133.30
133.27
133.17
133.14
132.45
132.36
132.08
131.97
131.88
131.73
131.57
131.49
131.18
131.15
116.62
116.40
116.30
116.24
116.08
115.88
115.84
115.66
115.63
115.22
102.20
93.61
40.59
40.38
40.17
39.96
39.75
39.54
39.33
34.68
33.92
Cl
N
C
N
N
S
F
F
3b
200
180
160
140
120
100
80
60
40
20
0
ppm

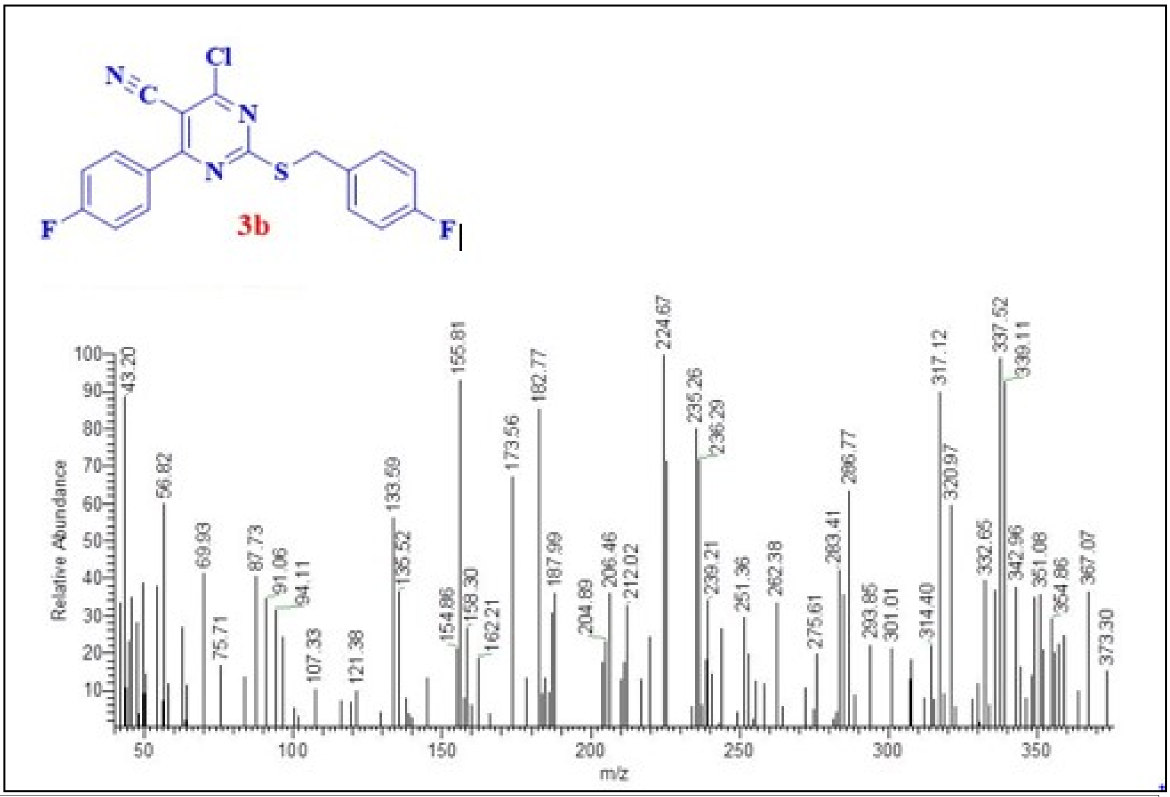

N C
Cl
N
N
S
F
F
3b
Relative Abundance
m/z
100
90
80
70
60
50
40
30
20
10
50
100
150
200
250
300
350
43.20
56.82
69.93
75.71
87.73
91.06
94.11
107.33
121.38
133.59
135.52
154.86
155.81
158.30
162.21
173.56
182.77
187.99
204.89
206.46
212.02
224.67
235.26
236.29
239.21
251.36
262.38
275.61
283.41
286.77
293.85
301.01
314.40
317.12
320.97
332.65
337.52
339.11
342.96
351.08
354.86
367.07
373.30

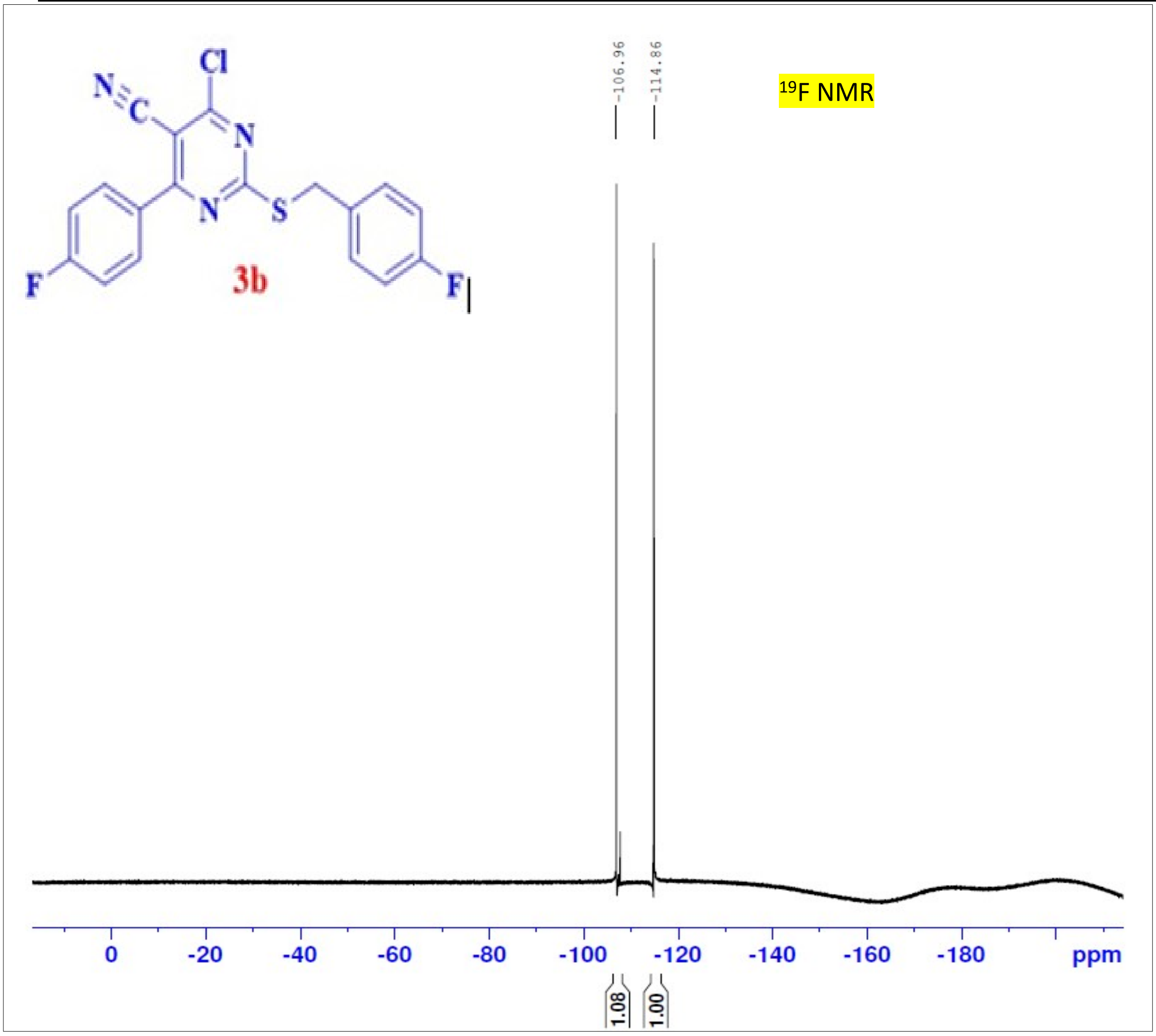

N C
Cl
N
N
S
F
F
3b
-106.96
-114.86
19F NMR
0
-20
-40
-60
-80
-100
-120
-140
-160
-180
ppm
1.08
1.00

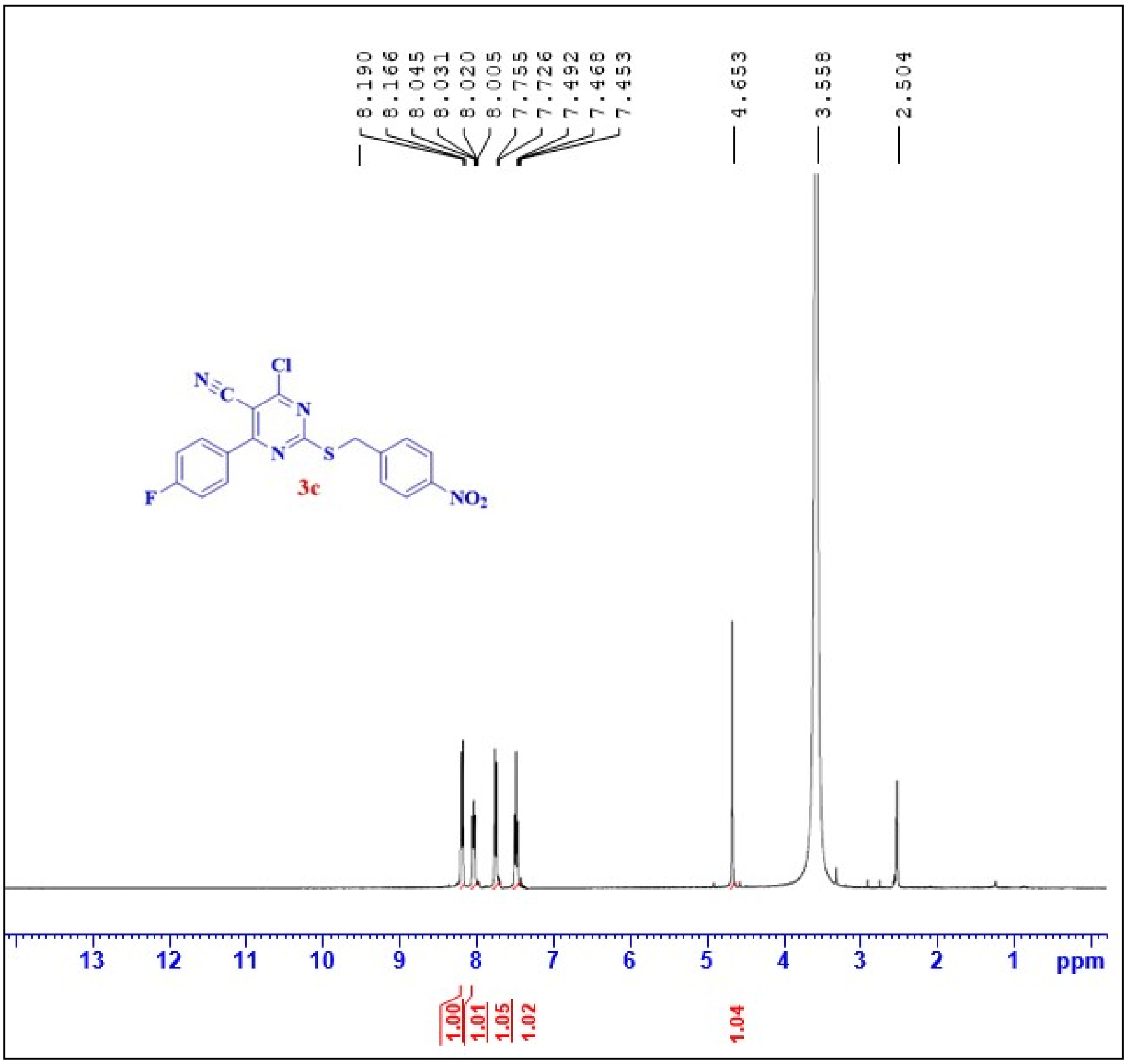
8.190
8.166
8.045
8.031
8.020
8.005
7.755
7.726
7.492
7.468
7.453
4.653
3.558
2.504
Cl
N
N
N
C
S
F
NO2
3c
13 12 11 10 9 8 7 6 5 4 3 2 1 ppm
1.00
1.01
1.05
1.02
1.04

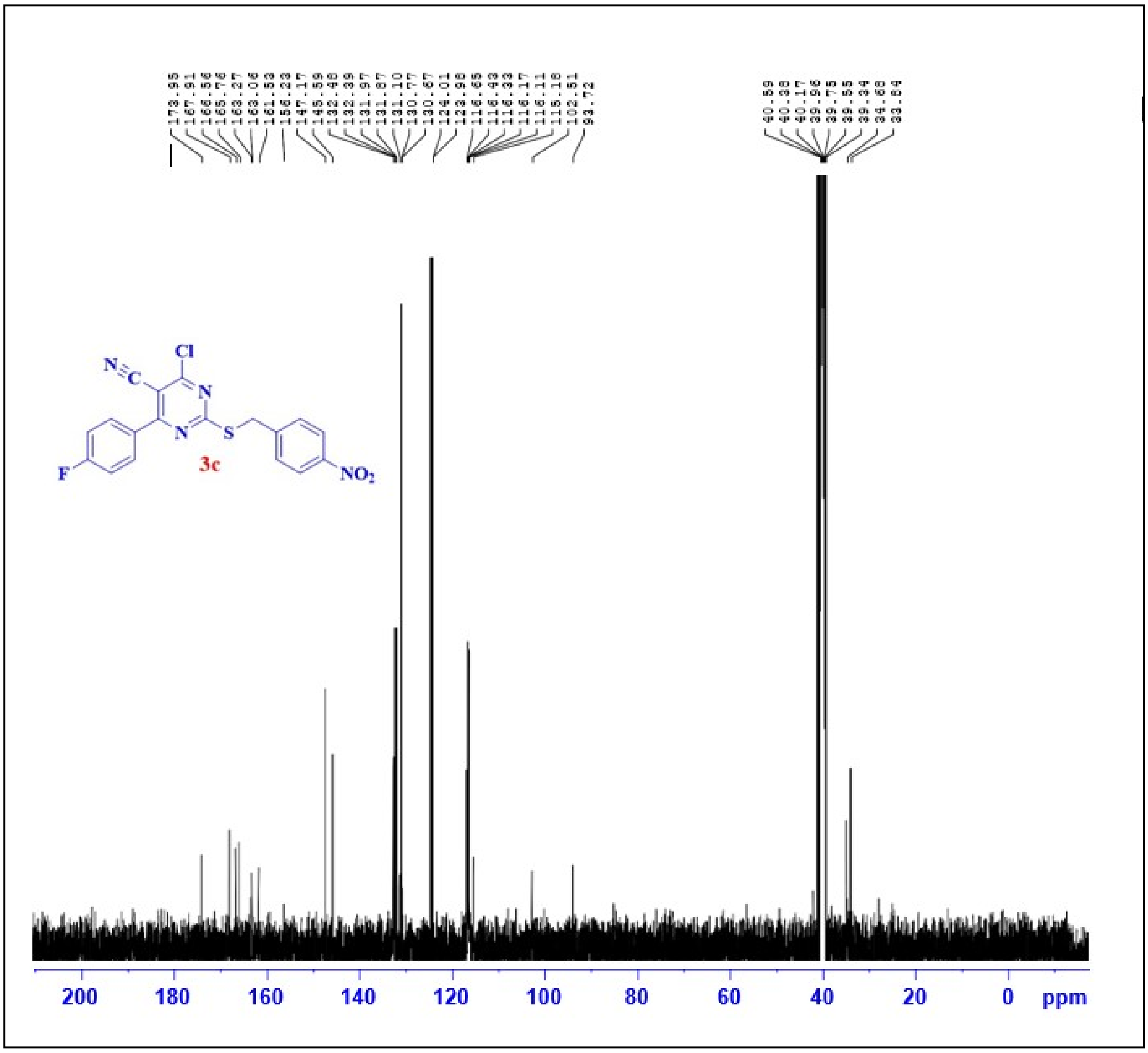
173.95
167.91
166.56
165.76
163.27
163.06
161.53
156.23
147.17
145.59
132.48
132.39
131.97
131.87
131.10
130.77
130.67
124.01
123.98
116.65
116.43
116.33
116.17
116.11
115.18
102.51
93.72
40.59
40.38
40.17
39.96
39.75
39.55
39.34
34.68
33.84
N
C
Cl
N
N
S
F
NO2
3c
200
180
160
140
120
100
80
60
40
20
0
ppm

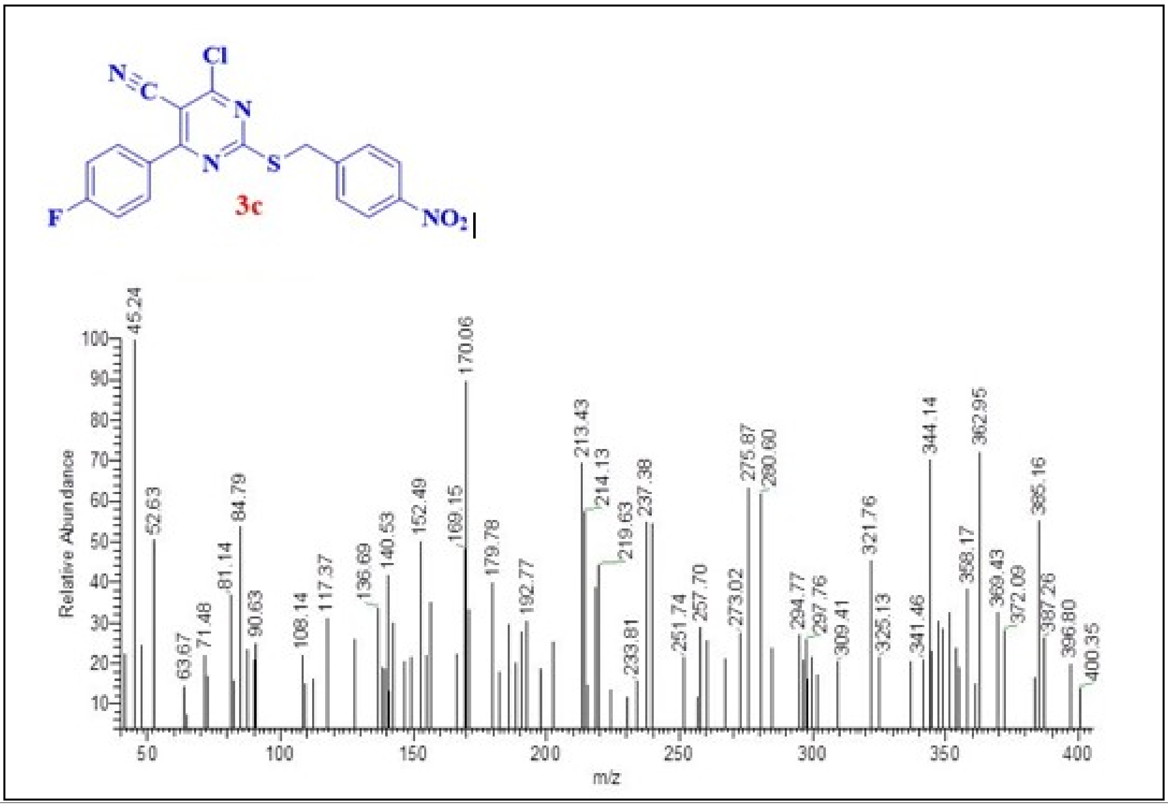

Cl
N
N
C
N
S
F
3c
NO2
Relative Abundance
m/z
45.24
52.63
63.67
71.48
81.14
84.79
90.63
108.14
117.37
136.69
140.53
152.49
169.15
170.06
179.78
192.77
213.43
214.13
219.63
233.81
237.38
251.74
257.70
273.02
275.87
280.60
294.77
297.76
309.41
321.76
325.13
341.46
344.14
358.17
362.95
369.43
372.09
385.16
387.26
396.80
400.35

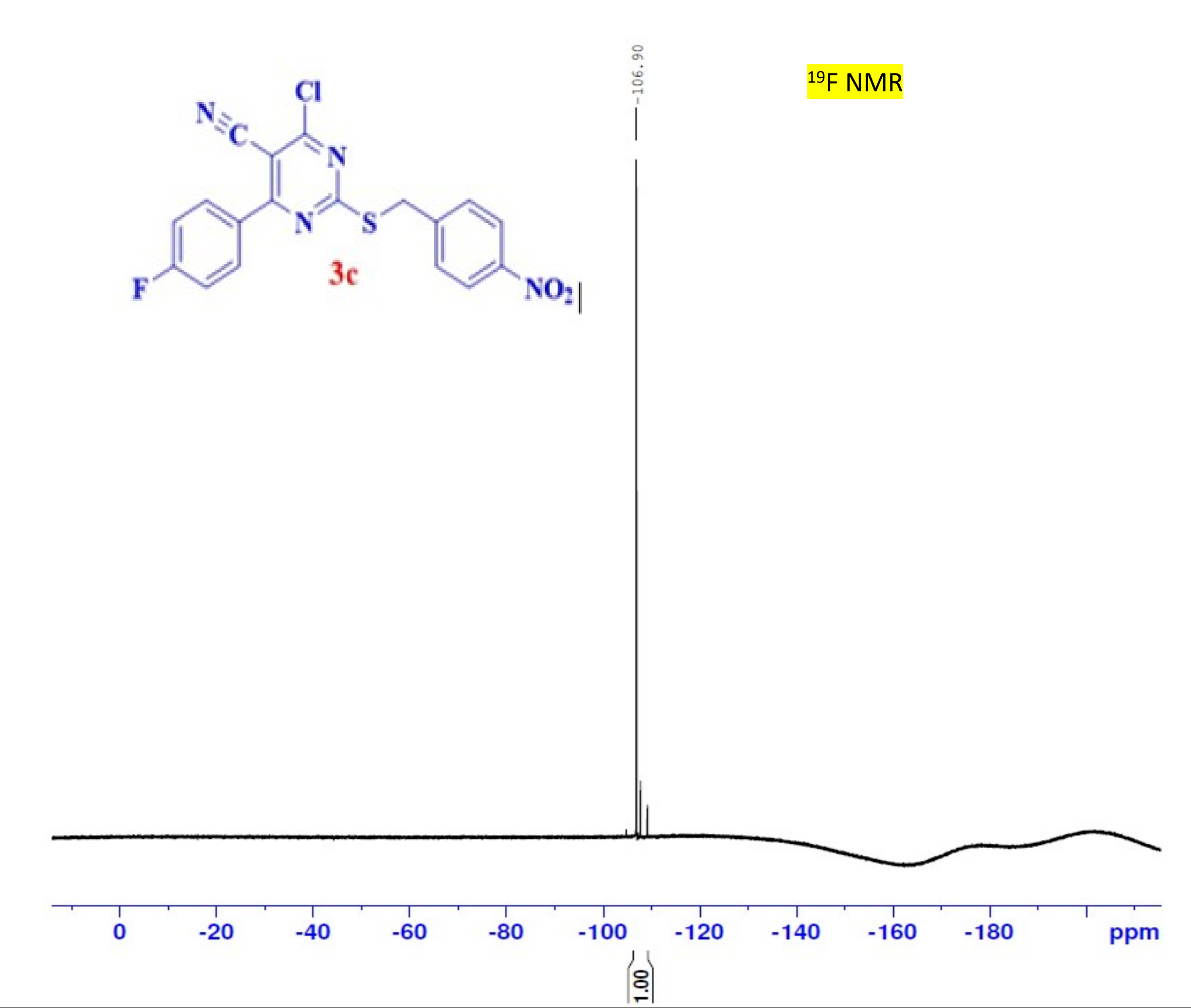

Cl
N
N
C
N
S
F
3c
NO2
-106.90
19F NMR
ppm
1.00

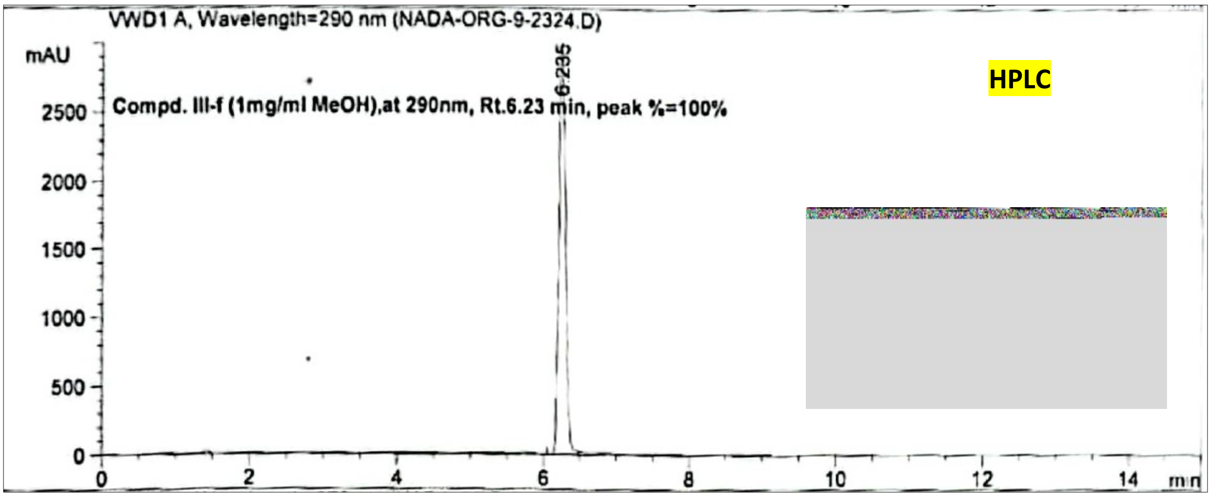
VWD1 A, Wavelength=290 nm (NADA-ORG-9-2324.D)
mAU
Compd. III-f (1mg/ml MeOH),at 290nm, Rt.6.23 min, peak %=100%
6.235
HPLC
2500
2000
1500
1000
500
0
0
2
4
6
8
10
12
14
min

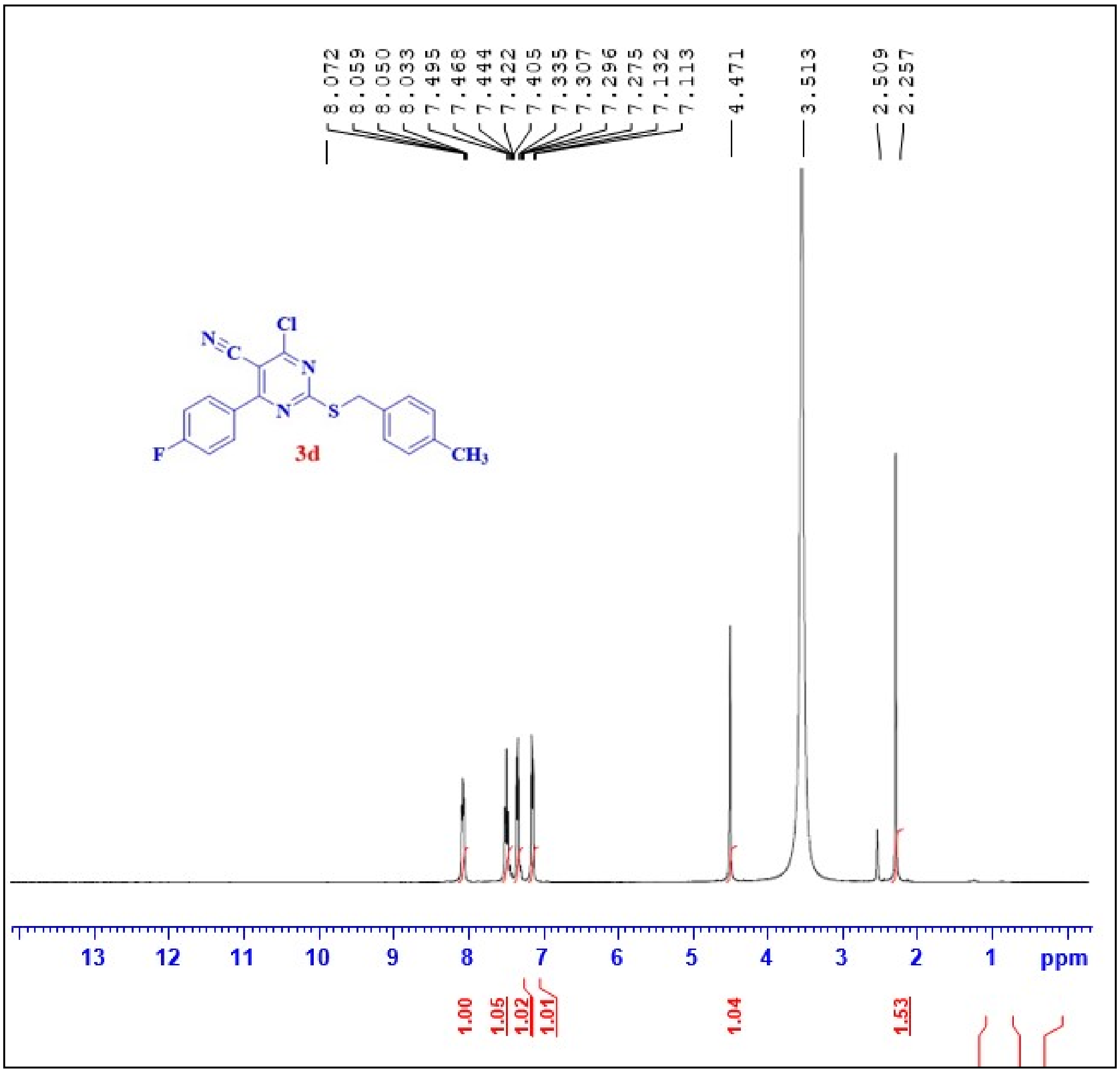
8.072
8.059
8.050
8.033
7.495
7.468
7.444
7.422
7.405
7.335
7.307
7.296
7.275
7.132
7.113
4.471
3.513
2.509
2.257
Cl
N
C
N
N
S
F
CH3
3d
13
12
11
10
9
8
7
6
5
4
3
2
1
ppm
1.00
1.05
1.02
1.01
1.04
1.53

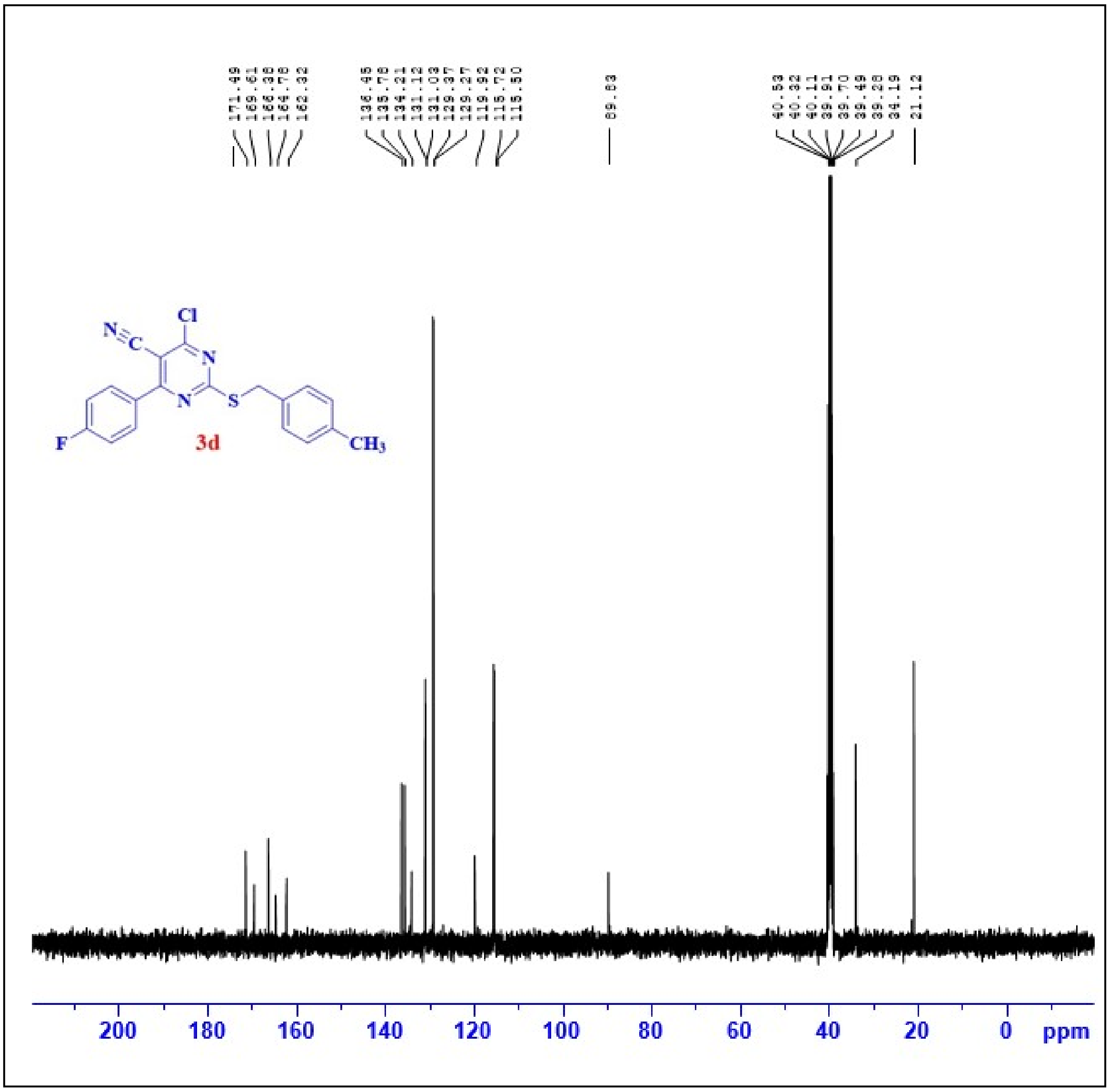
171.49
169.61
166.38
164.78
162.32
136.45
135.78
134.21
131.12
131.03
129.37
129.27
119.92
115.72
115.50
89.83
40.53
40.32
40.11
39.91
39.70
39.49
39.28
34.19
21.12
N
C
Cl
N
N
S
F
3d
CH3
200
180
160
140
120
100
80
60
40
20
0
ppm

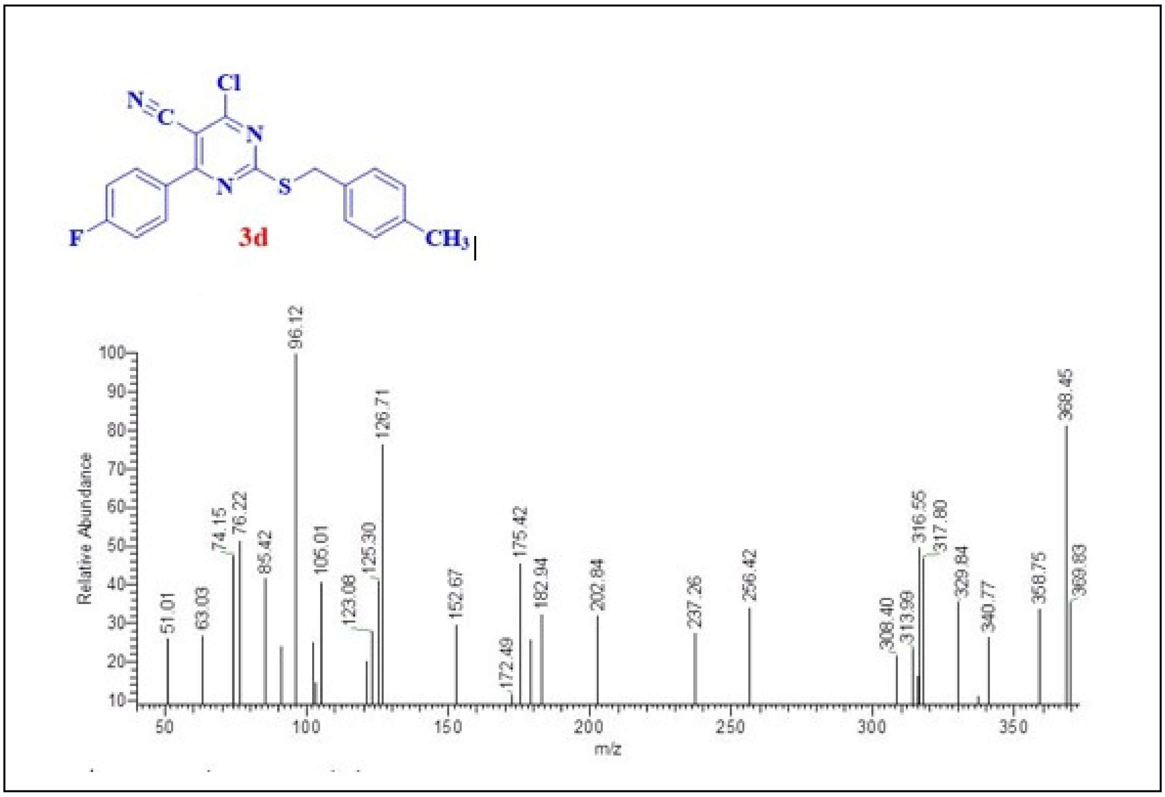

N
C
Cl
N
N
S
F
3d
CH3
Relative Abundance
m/z
96.12
126.71
368.45
74.15
76.22
85.42
105.01
125.30
123.08
51.01
63.03
152.67
172.49
175.42
182.94
202.84
237.26
256.42
308.40
313.99
316.55
317.80
329.84
340.77
358.75
369.83

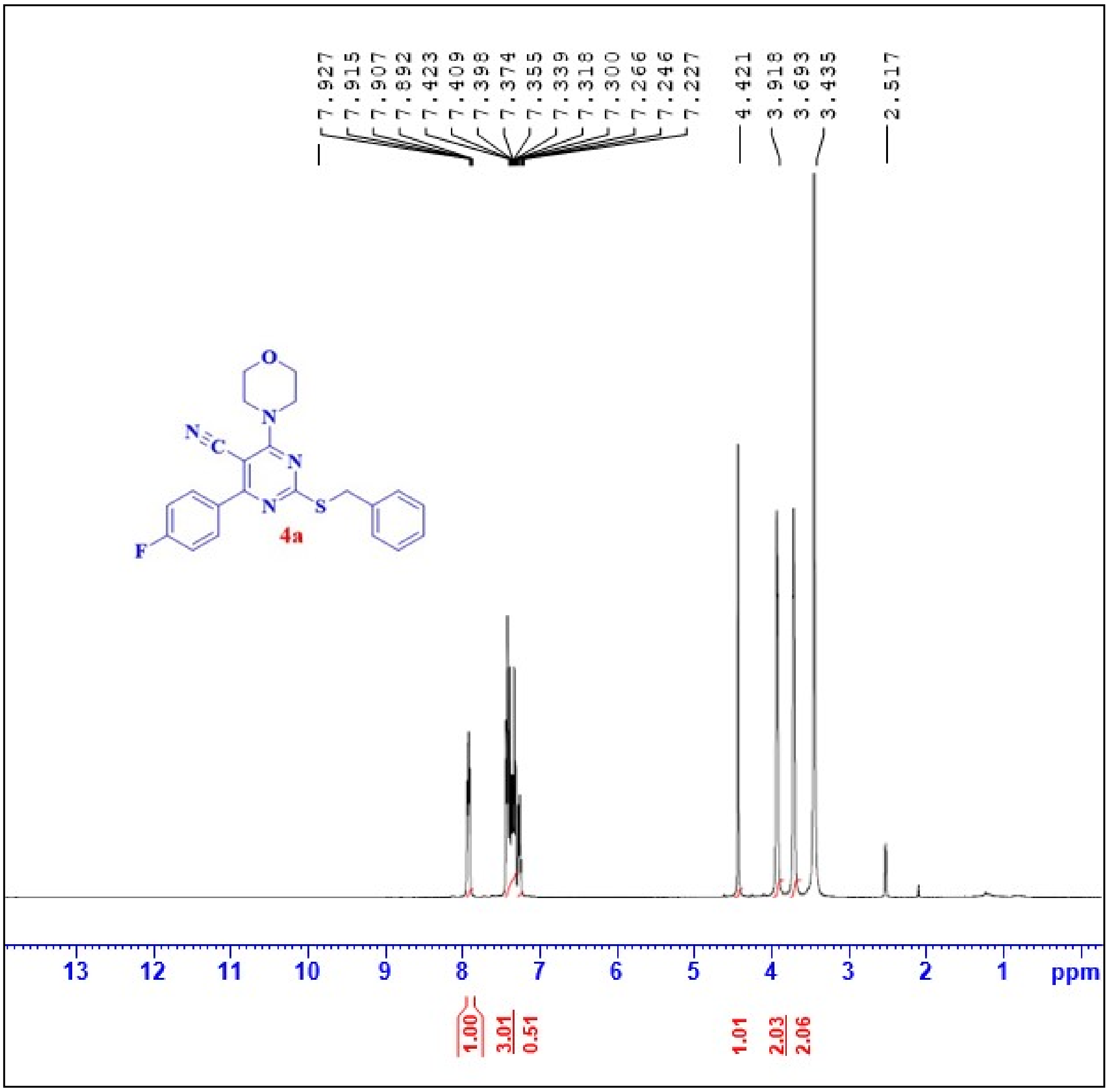
7.927
7.915
7.907
7.892
7.423
7.409
7.398
7.374
7.355
7.339
7.318
7.300
7.266
7.246
7.227
4.421
3.918
3.693
3.435
2.517
N
C
N
O
N
N
S
F
4a
13
12
11
10
9
8
7
6
5
4
3
2
1
ppm
1.00
3.01
0.51
1.01
2.03
2.06

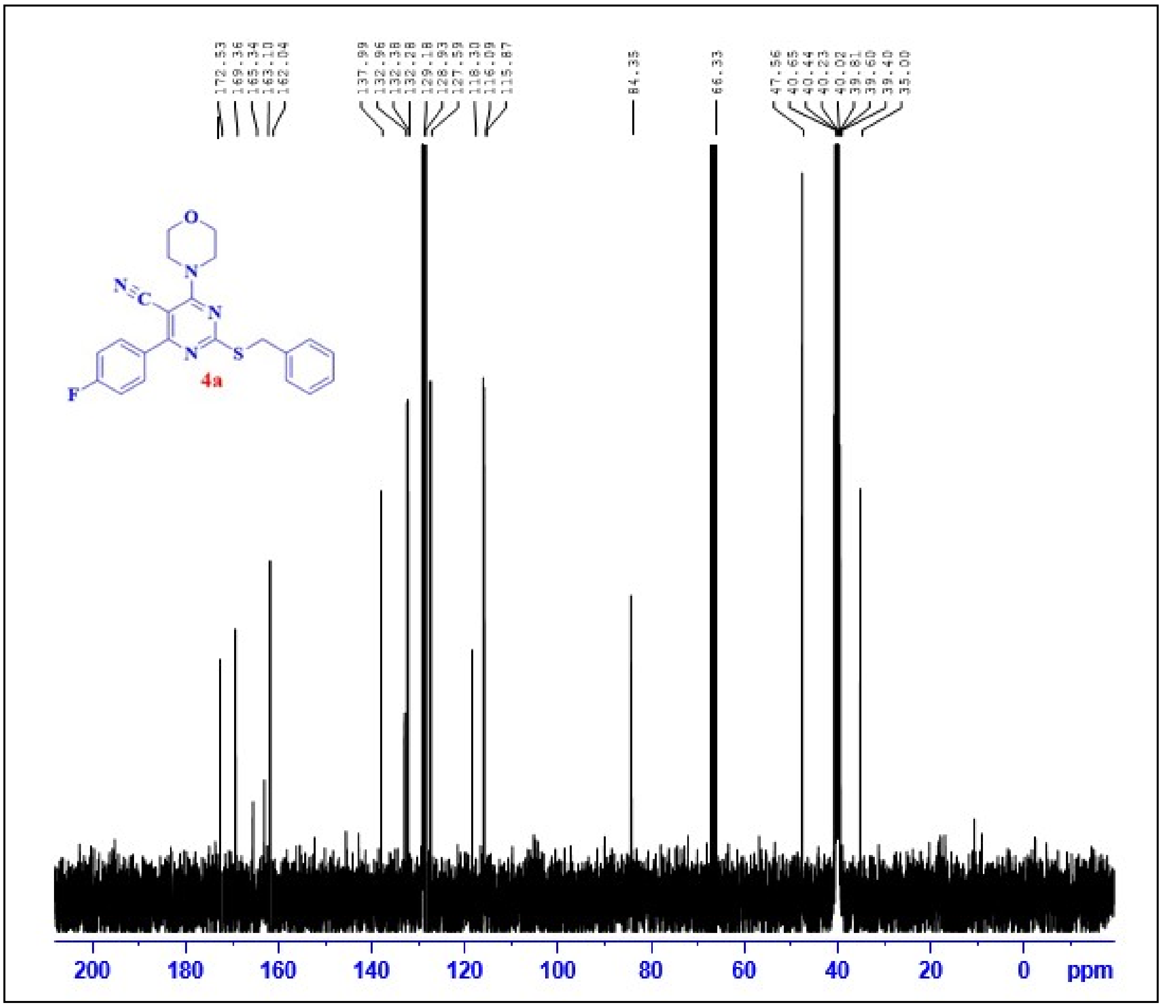
172.53
169.36
165.34
163.10
162.04
137.99
132.96
132.38
132.28
129.18
128.93
127.59
118.30
116.09
115.87
84.35
66.33
47.56
40.65
40.44
40.23
40.02
39.81
39.60
39.40
35.00
O
N
N
C
N
N
S
F
4a
200
180
160
140
120
100
80
60
40
20
0
ppm

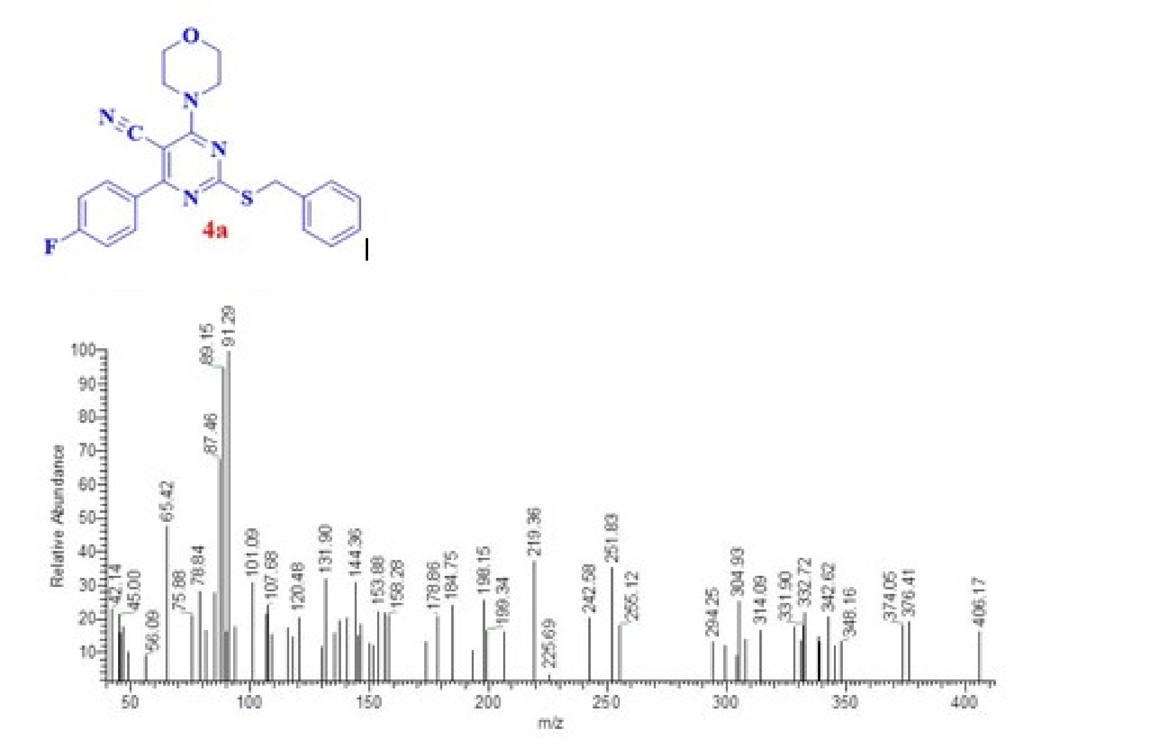
O
N
N
C
N
N
S
F
4a
Relative Abundance
m/z
42.14
45.00
56.09
65.42
75.88
78.84
87.46
89.15
91.29
101.09
107.68
120.48
131.90
144.36
153.88
158.28
178.86
184.75
198.15
199.34
219.36
225.69
242.58
251.83
255.12
294.25
304.93
314.09
331.90
332.72
342.62
348.16
374.05
376.41
406.17

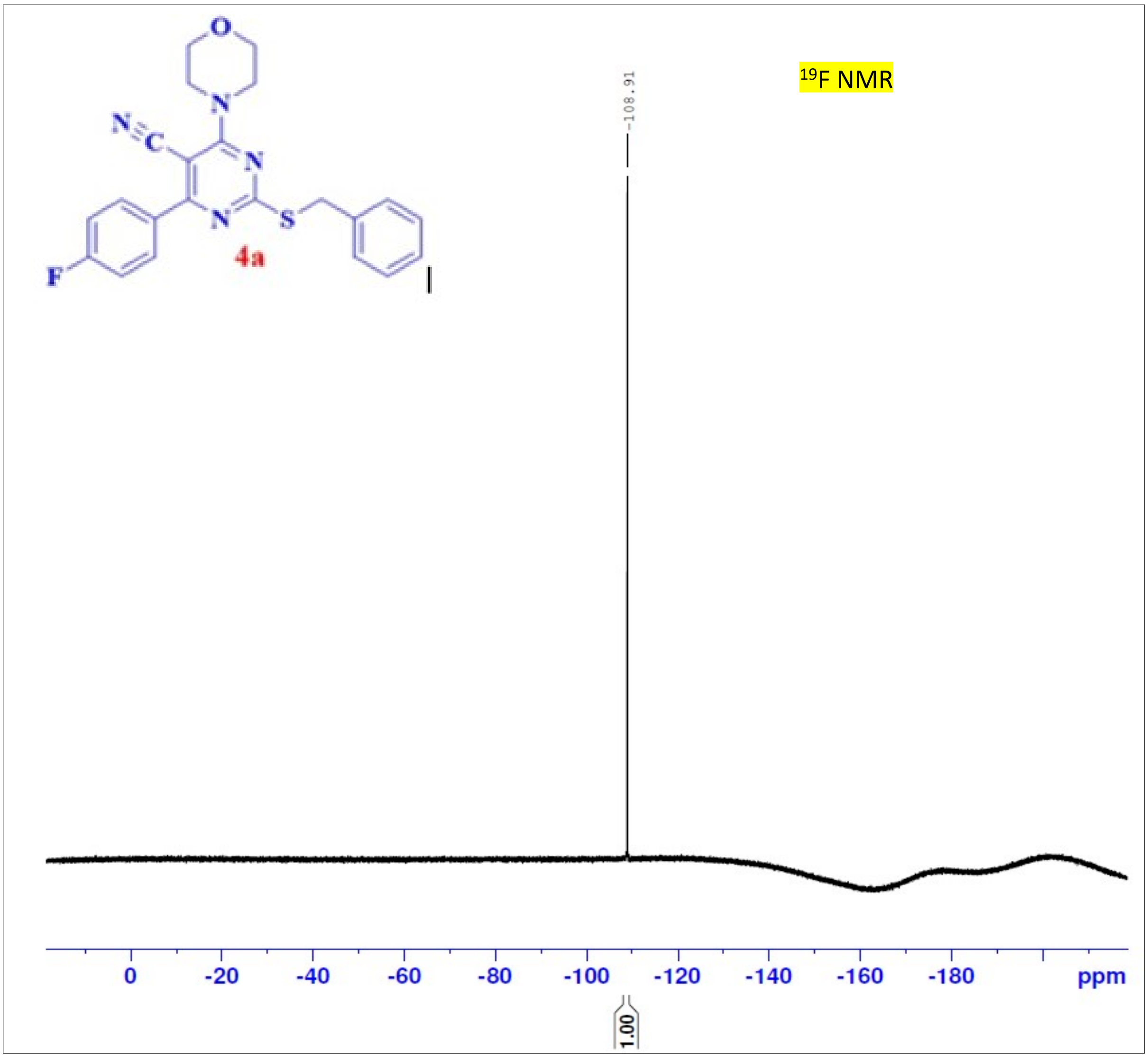
4a
19F NMR
-108.91
0
-20
-40
-60
-80
-100
-120
-140
-160
-180
ppm
1.00

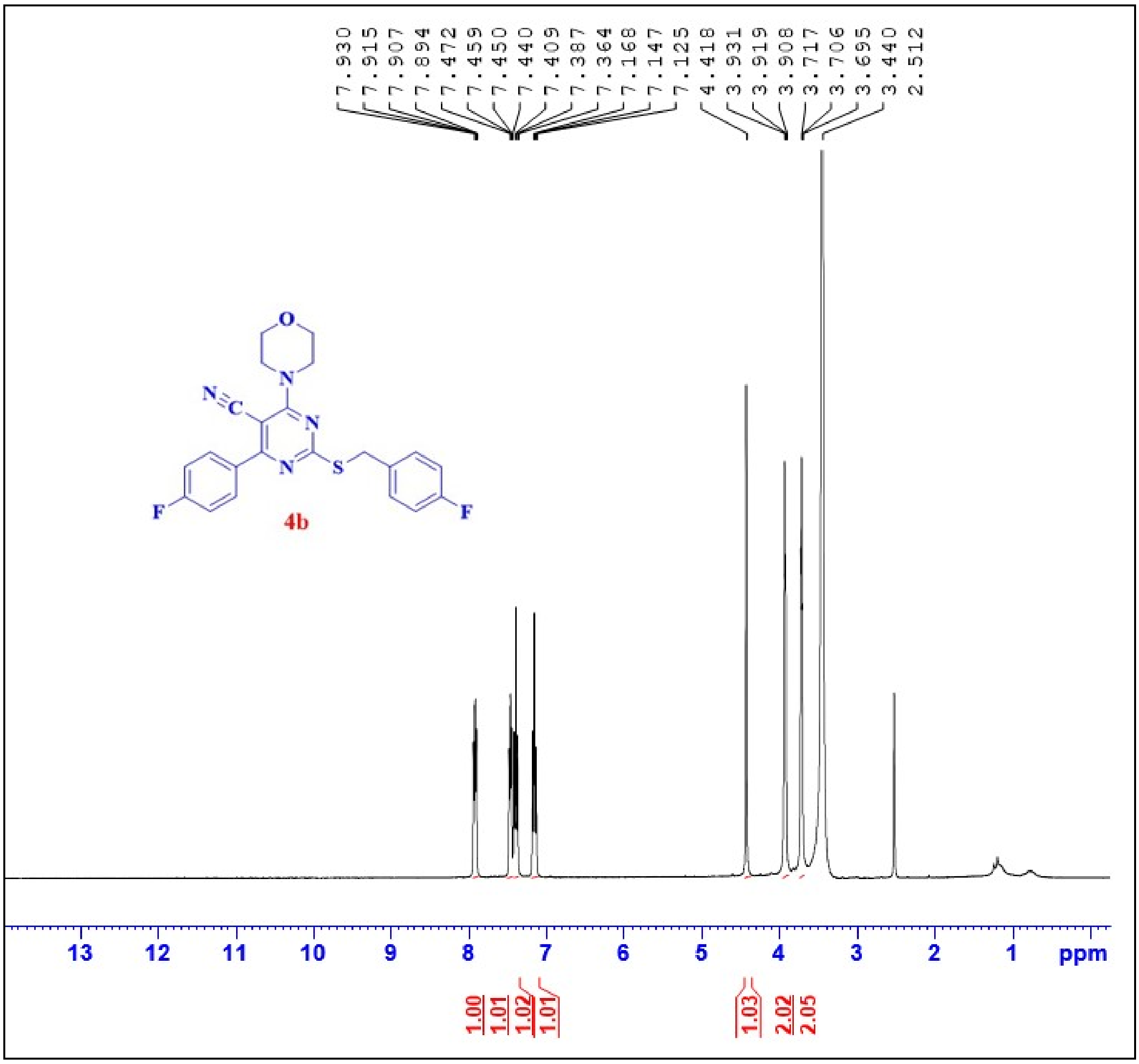
7.930
7.915
7.907
7.894
7.472
7.459
7.450
7.440
7.409
7.387
7.364
7.168
7.147
7.125
4.418
3.931
3.919
3.908
3.717
3.706
3.695
3.440
2.512
N
C
O
N
N
N
S
F
F
4b
13 12 11 10 9 8 7 6 5 4 3 2 1 ppm
1.00
1.01
1.02
1.01
1.03
2.02
2.05

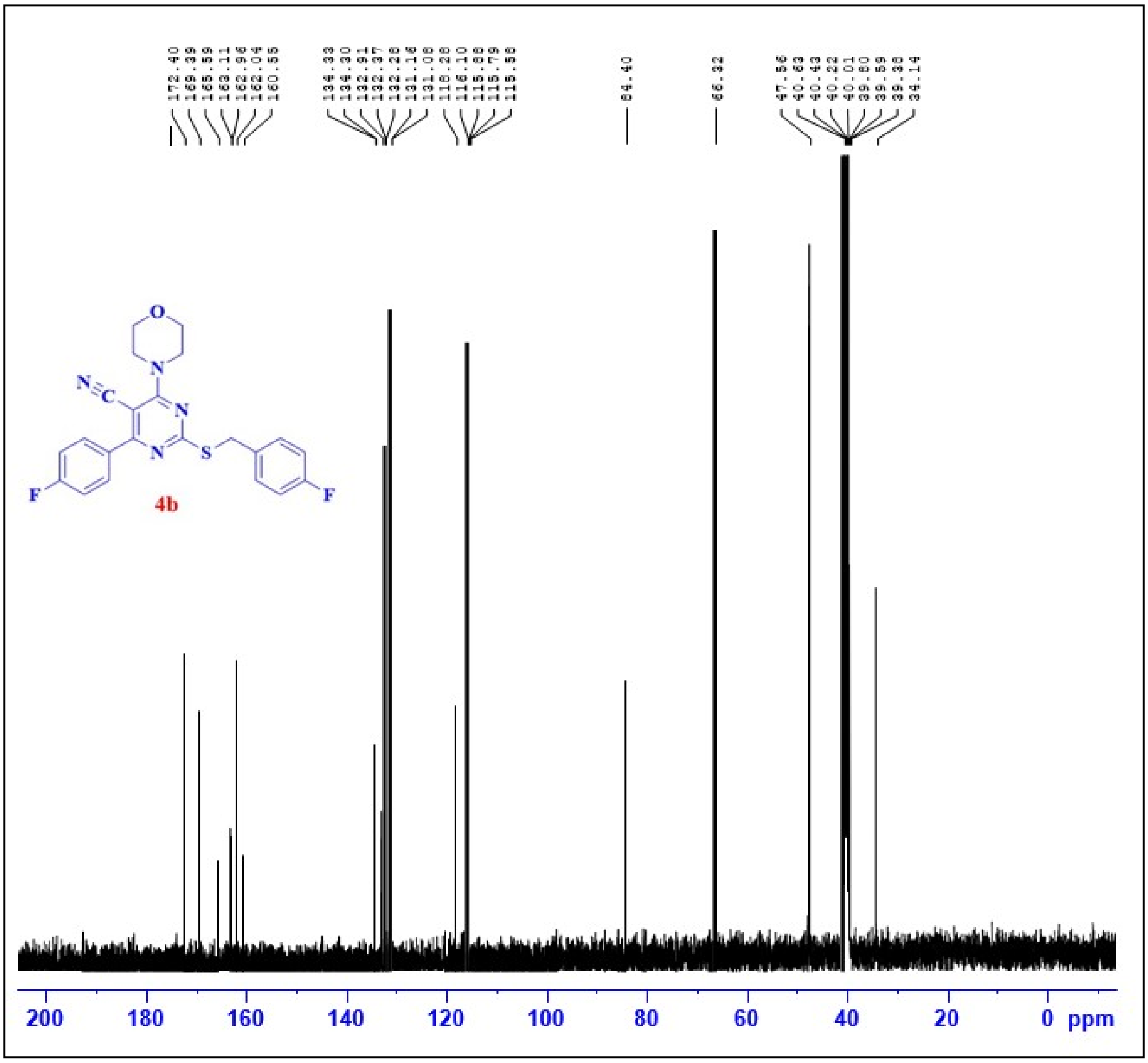
172.40
169.39
165.59
163.11
162.96
162.04
160.55
134.33
134.30
132.91
132.37
132.28
131.16
131.08
118.28
116.10
115.88
115.79
115.58
84.40
66.32
47.56
40.63
40.43
40.22
40.01
39.80
39.59
39.38
34.14
O
N
N
C
N
N
S
F
F
4b
200
180
160
140
120
100
80
60
40
20
0 ppm

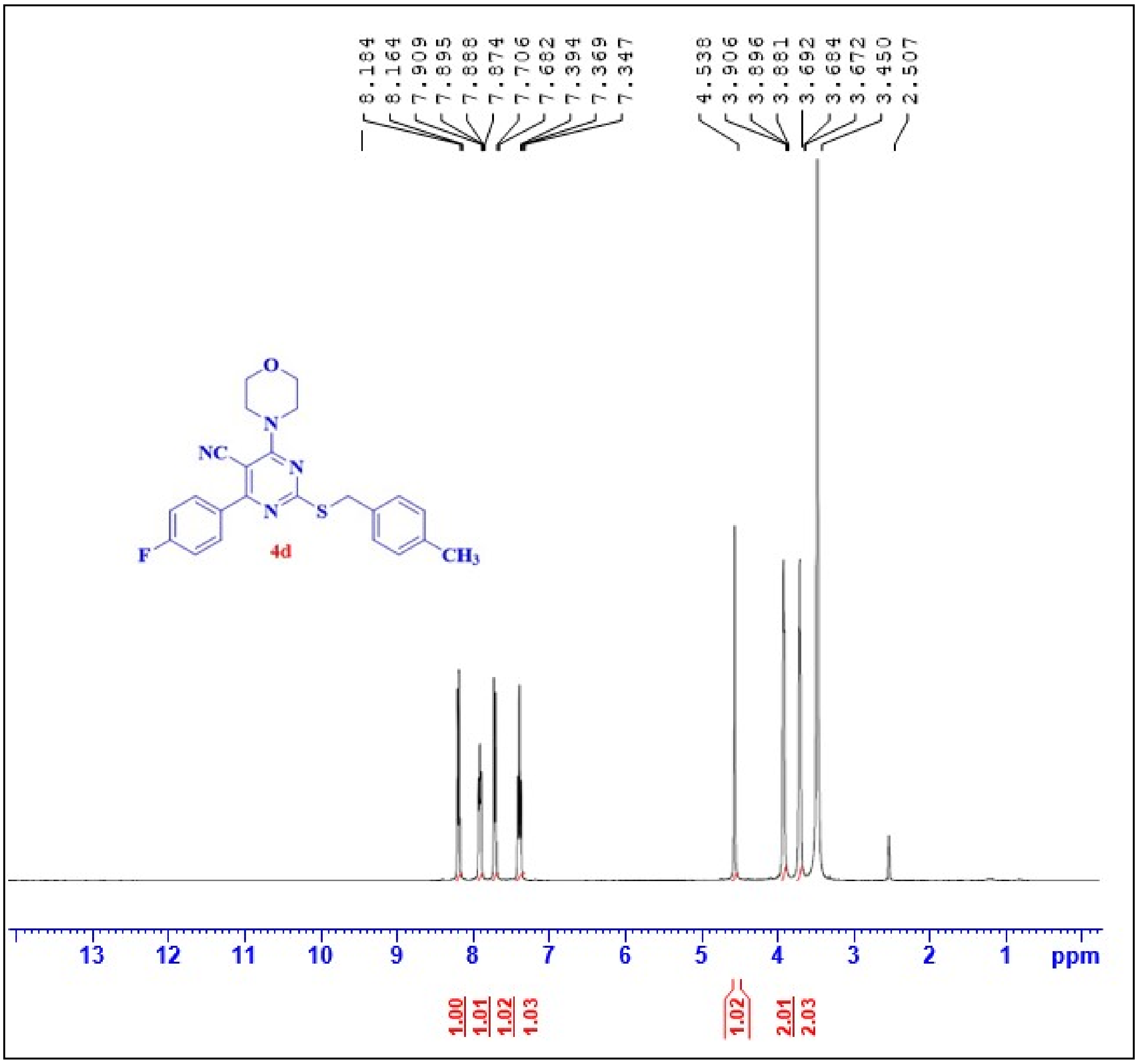
8.184
8.164
7.909
7.895
7.888
7.874
7.706
7.682
7.394
7.369
7.347
4.538
3.906
3.896
3.881
3.692
3.684
3.672
3.450
2.507
NC
N
N
O
S
F
CH3
4d
13 12 11 10 9 8 7 6 5 4 3 2 1 ppm
1.00
1.01
1.02
1.03
1.02
2.01
2.03

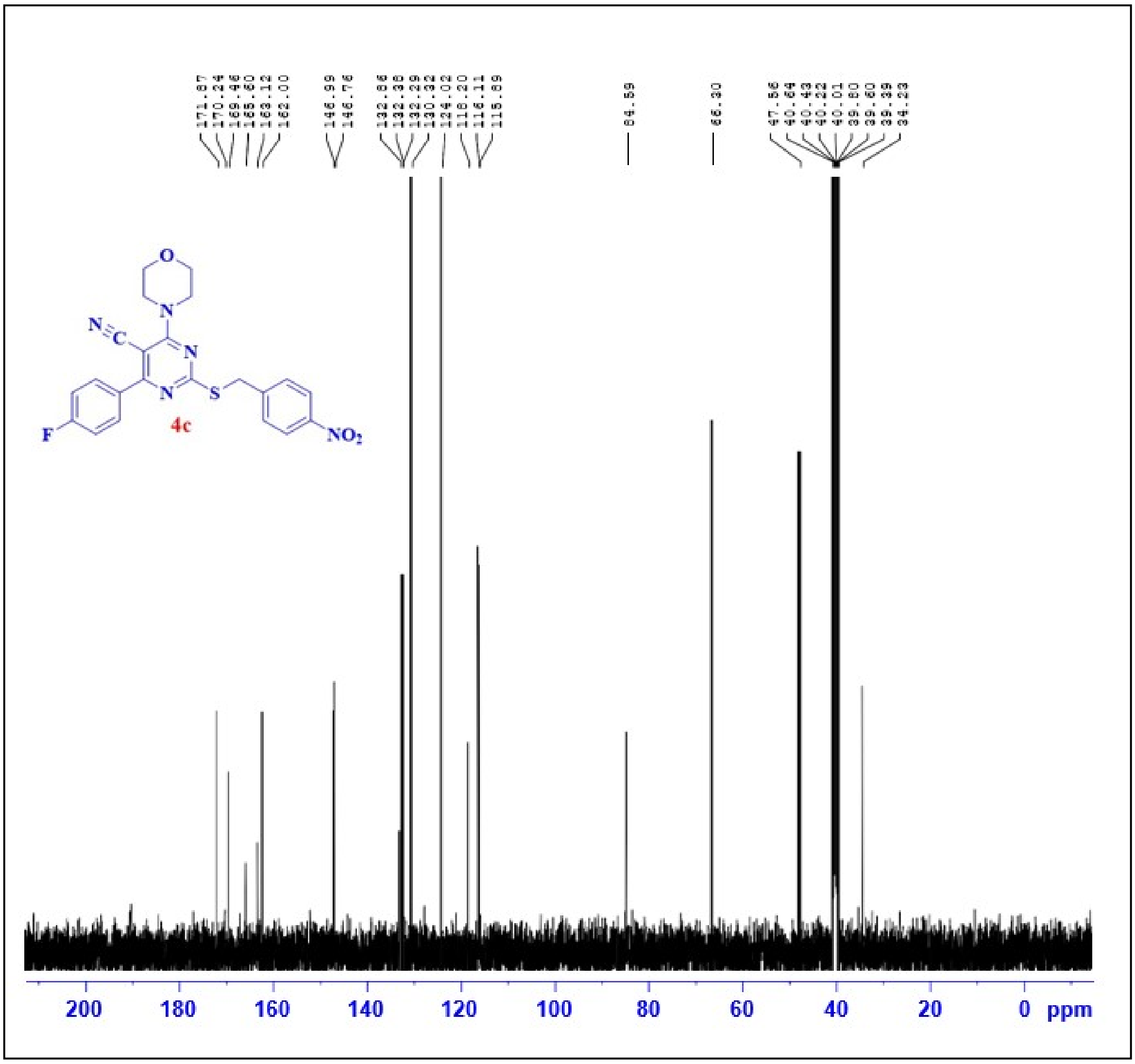
171.87
170.24
169.46
165.60
163.12
162.00
146.99
146.76
132.86
132.38
132.29
130.32
124.02
118.20
116.11
115.89
84.59
66.30
47.56
40.64
40.43
40.22
40.01
39.80
39.60
39.39
34.23
N
C
N
N
O
N
S
F
NO2
4c
200
180
160
140
120
100
80
60
40
20
0
ppm

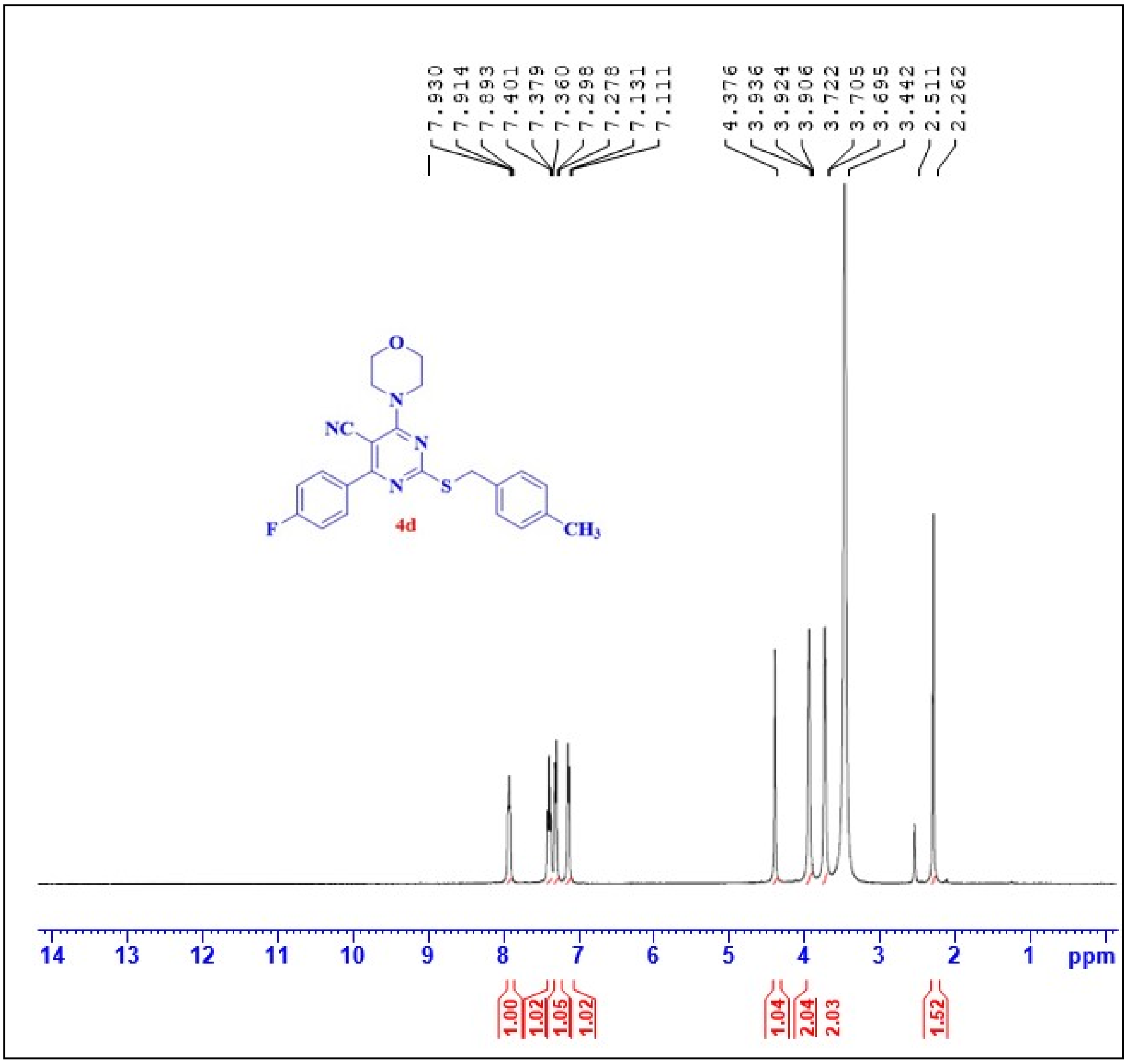

7.930
7.914
7.893
7.401
7.379
7.360
7.298
7.278
7.131
7.111
4.376
3.936
3.924
3.906
3.722
3.705
3.695
3.442
2.511
2.262
O
N
NC
N
N
S
F
4d
CH3
14 13 12 11 10 9 8 7 6 5 4 3 2 1 ppm
1.00
1.02
1.05
1.02
1.04
2.04
2.03
1.52

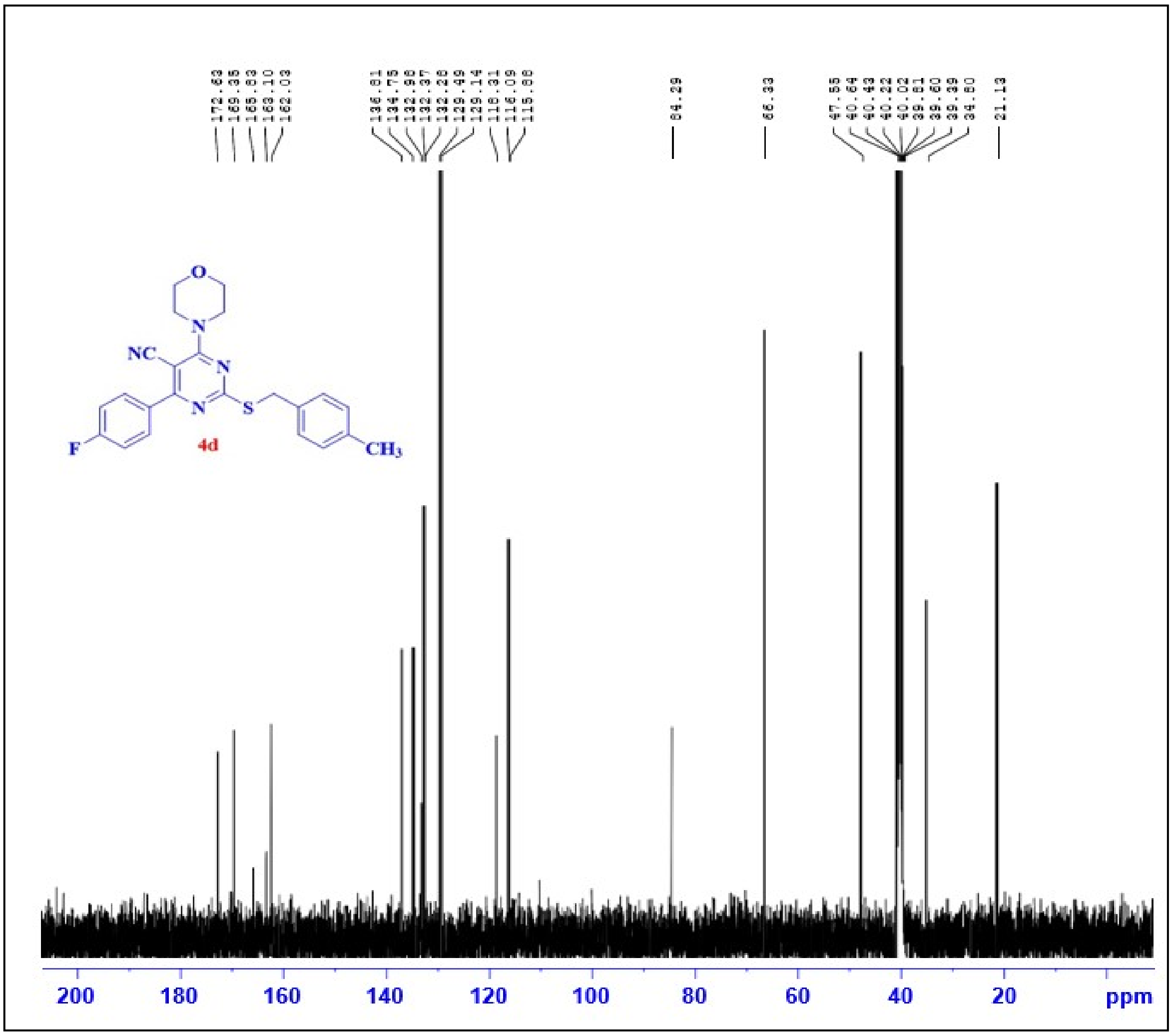

172.63
169.35
165.83
163.10
162.03
136.81
134.75
132.98
132.37
132.28
129.49
129.14
118.31
116.09
115.88
84.29
66.33
47.55
40.64
40.43
40.22
40.02
39.81
39.60
39.39
34.80
21.13
NC
N
N
N
O
S
F
CH3
4d
200
180
160
140
120
100
80
60
40
20
ppm

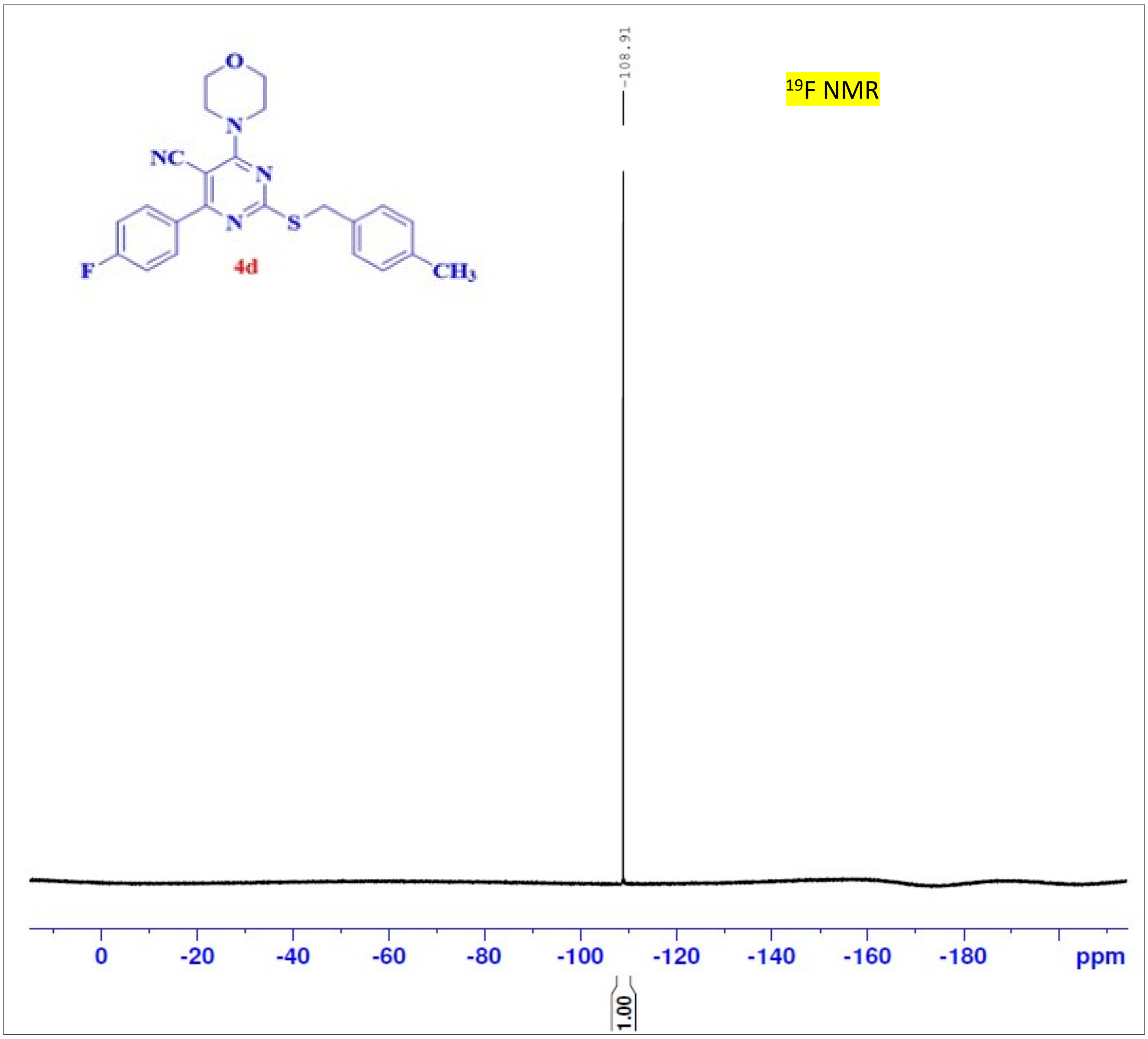
-108.91
19F NMR
NC
N
O
S
F
4d
CH3
0
-20
-40
-60
-80
-100
-120
-140
-160
-180
ppm
1.00

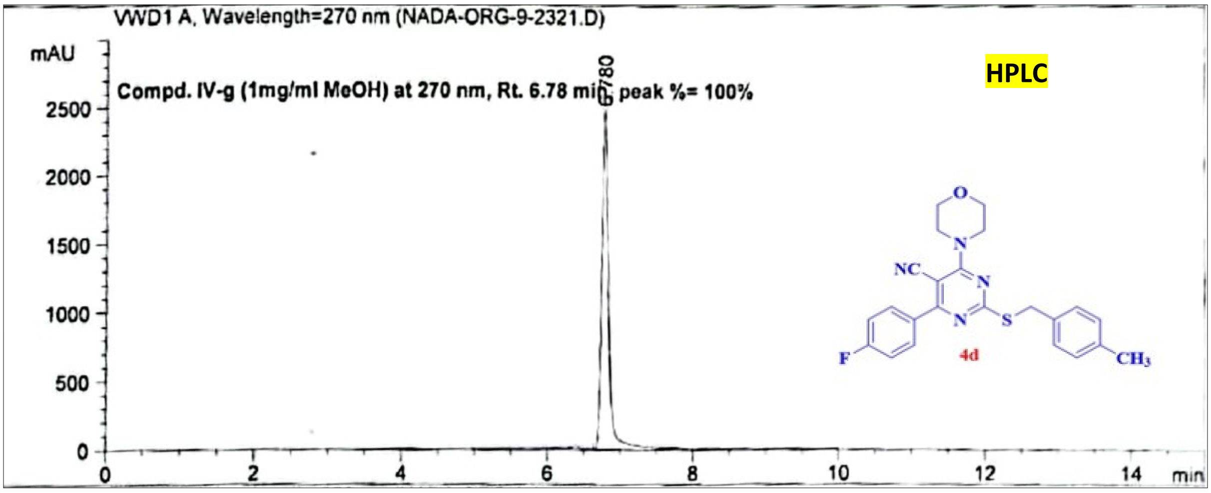
VWD1 A, Wavelength=270 nm (NADA-ORG-9-2321.D)
mAU
Compd. IV-g (1mg/ml MeOH) at 270 nm, Rt. 6.78 min, peak %= 100%
6.780
HPLC
2500
2000
1500
1000
500
0
2
4
6
8
10
12
14
min
NC
4d
CH3

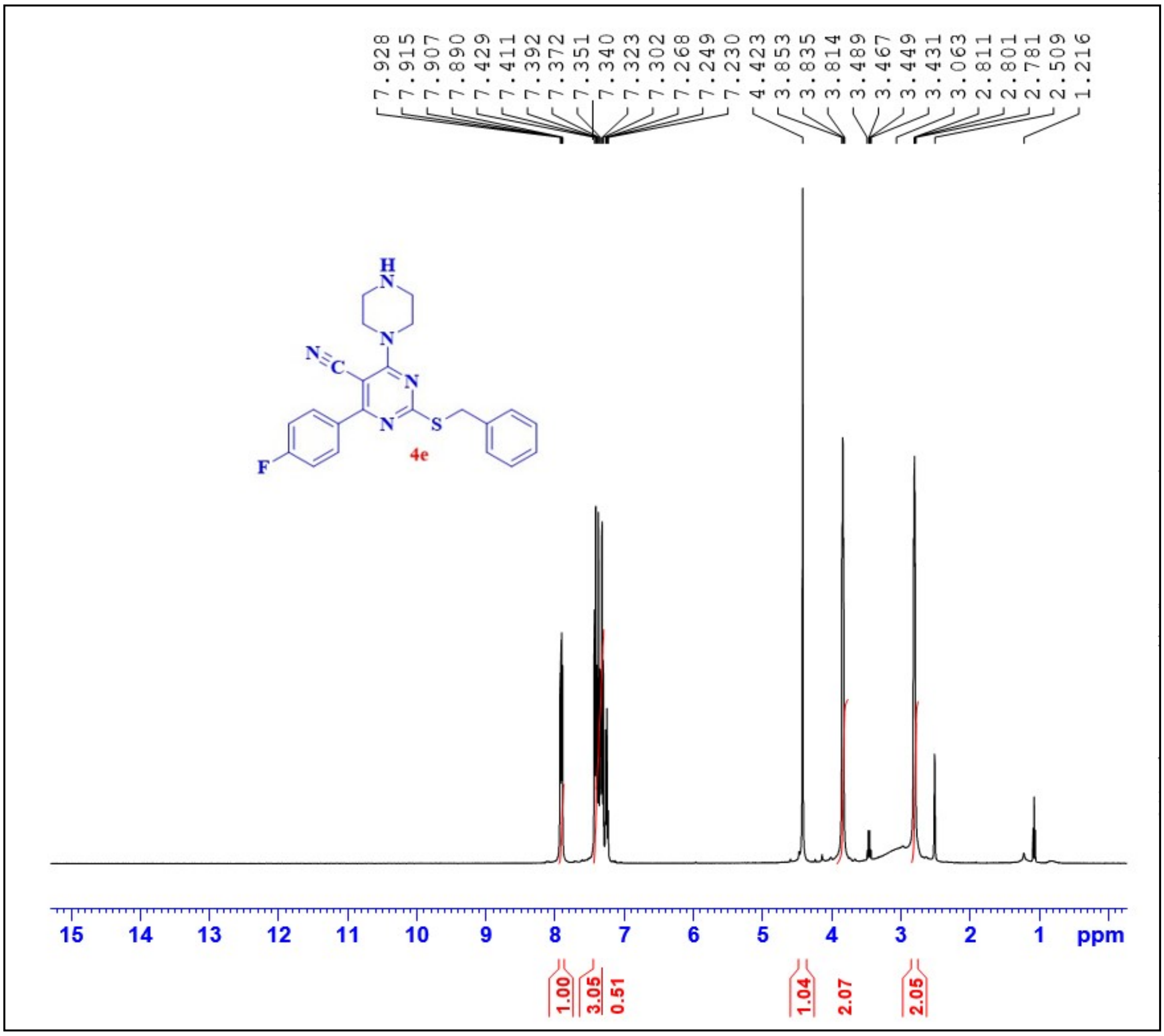
7.928
7.915
7.907
7.890
7.429
7.411
7.392
7.372
7.351
7.340
7.323
7.302
7.268
7.249
7.230
4.423
3.853
3.835
3.814
3.489
3.467
3.449
3.431
3.063
2.811
2.801
2.781
2.509
1.216
H
N
N
N
C
N
N
S
F
4e
15
14
13
12
11
10
9
8
7
6
5
4
3
2
1
ppm
1.00
3.05
0.51
1.04
2.07
2.05

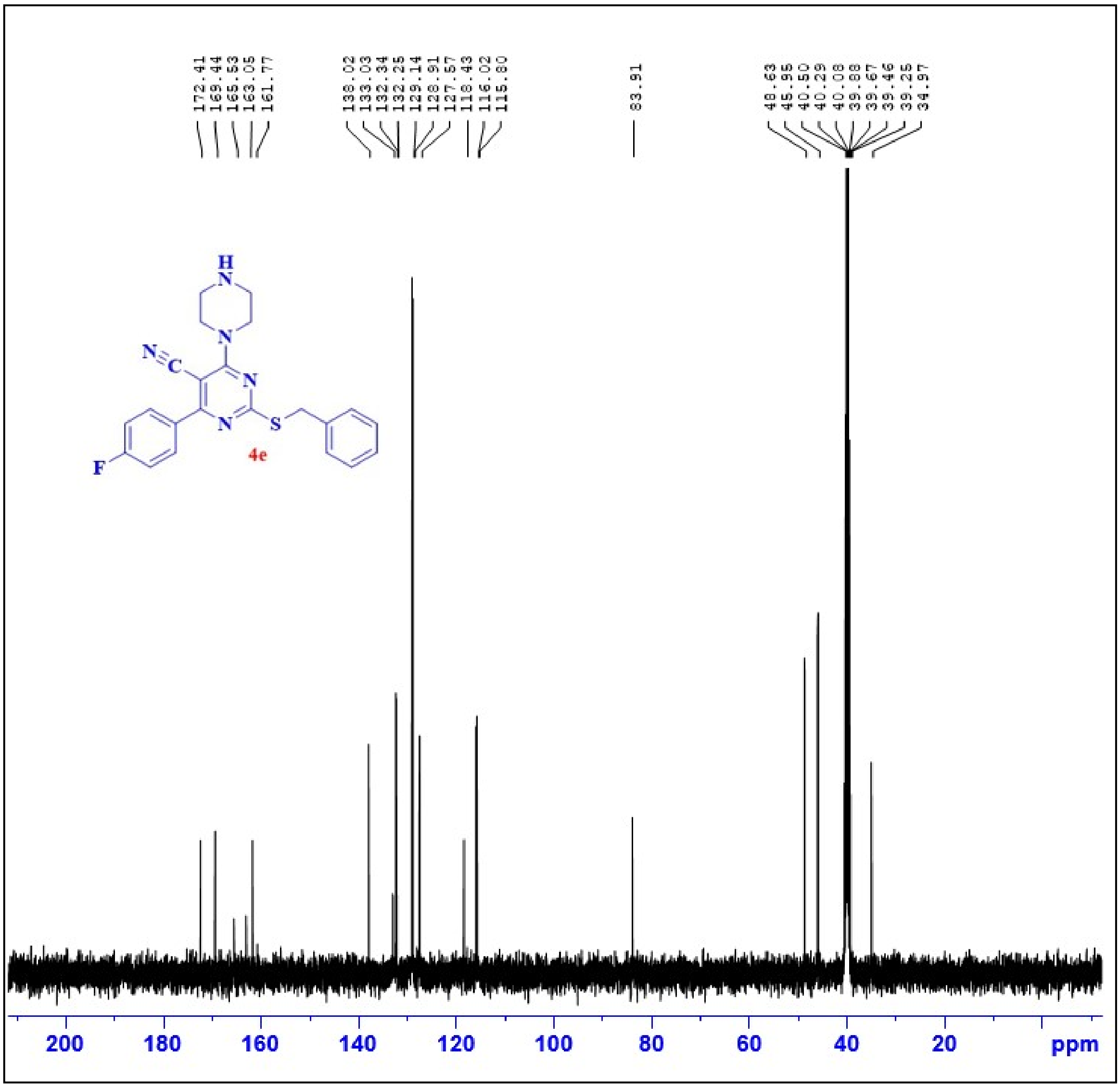

172.41
169.44
165.53
163.05
161.77
138.02
133.03
132.34
132.25
129.14
128.91
127.57
118.43
116.02
115.80
83.91
48.63
45.95
40.50
40.29
40.08
39.88
39.67
39.46
39.25
34.97
H
N
N
N
C
N
N
S
F
4e
200
180
160
140
120
100
80
60
40
20
ppm

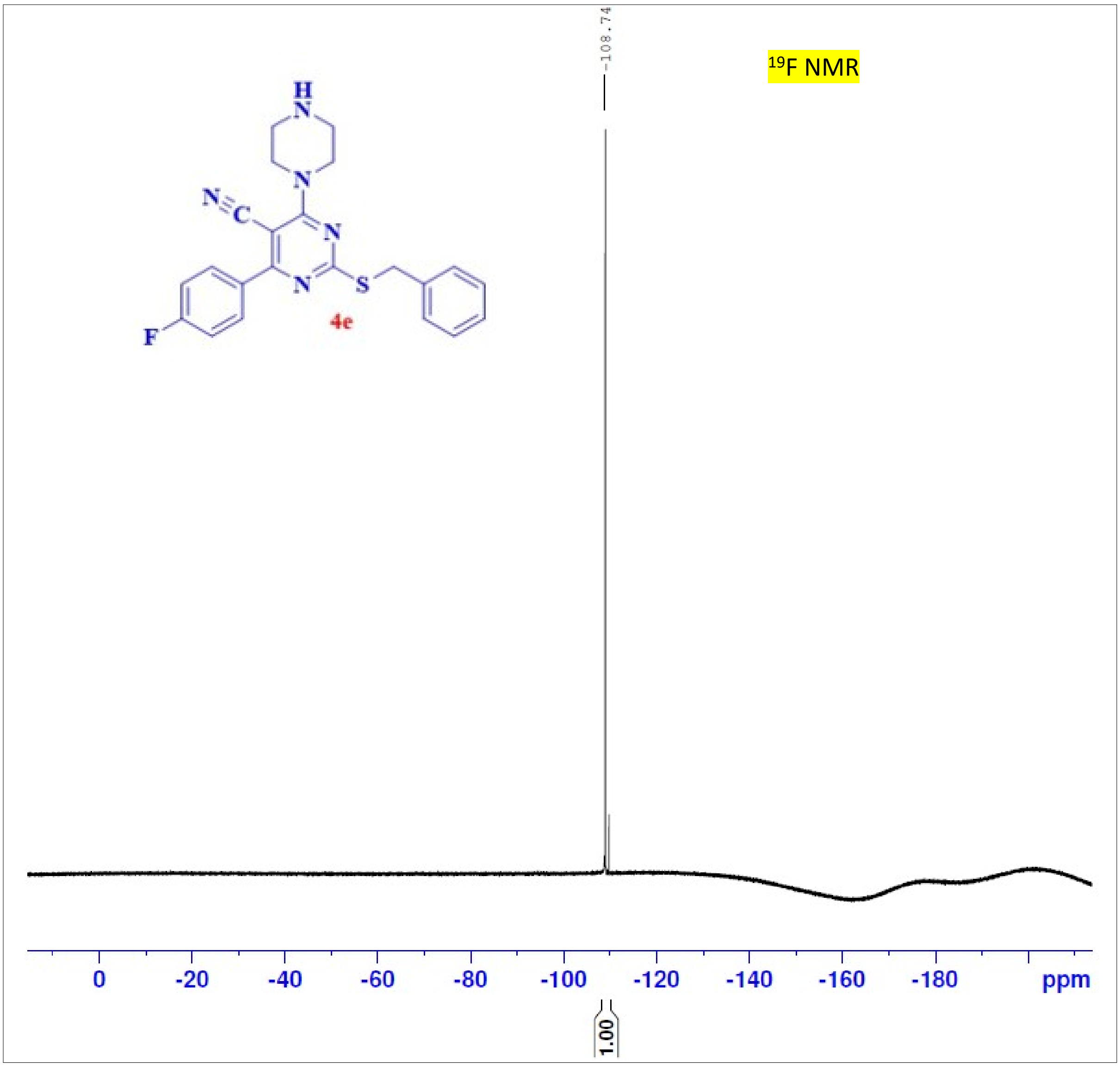

-108.74
19F NMR
H
N
N
N
C
N
N
S
F
4e
0
-20
-40
-60
-80
-100
-120
-140
-160
-180
ppm
1.00

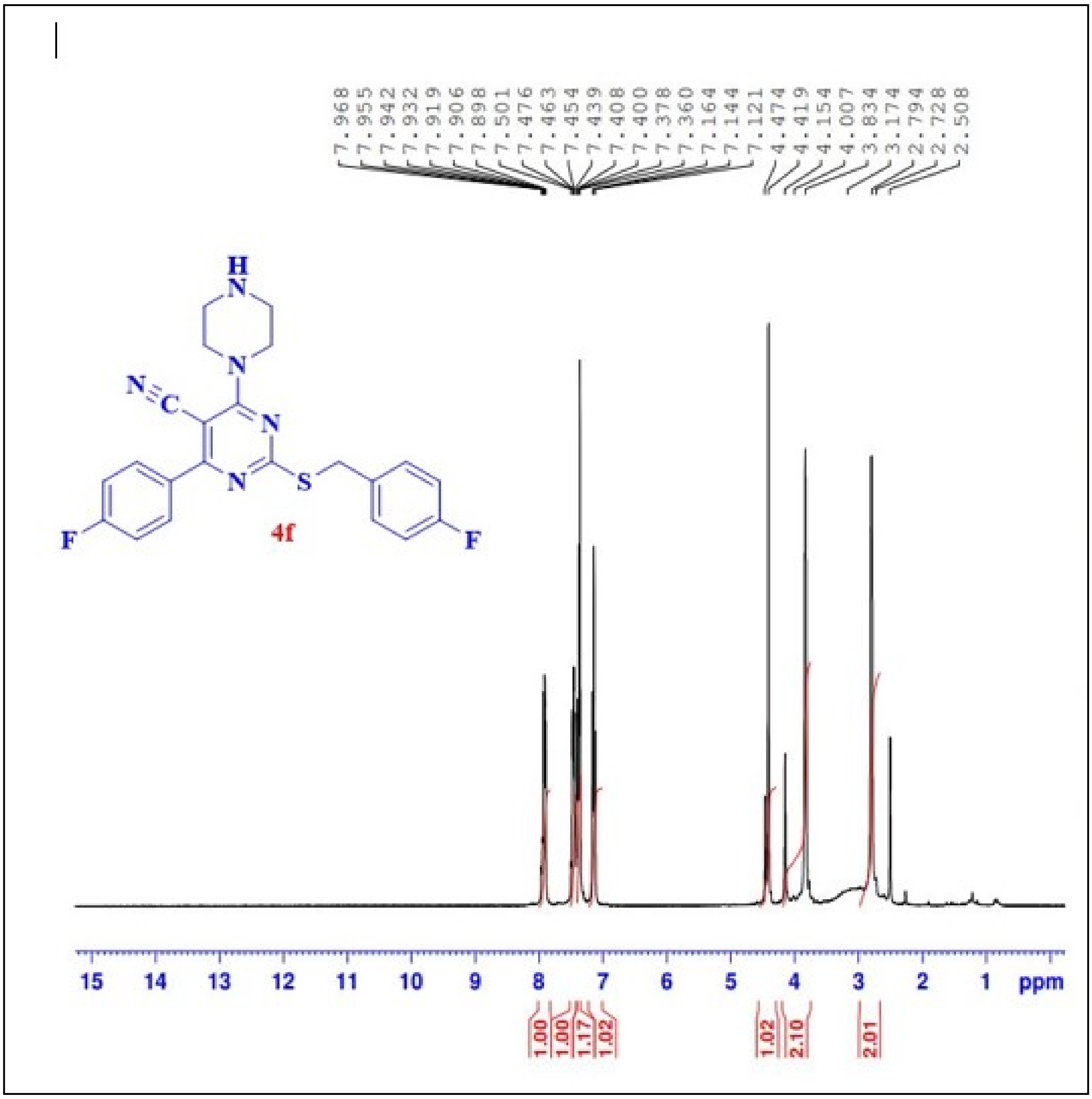
7.968
7.955
7.942
7.932
7.919
7.906
7.898
7.501
7.476
7.463
7.454
7.439
7.408
7.400
7.378
7.360
7.164
7.144
7.121
4.474
4.419
4.154
4.007
3.834
3.174
2.794
2.728
2.508
4f
15 14 13 12 11 10 9 8 7 6 5 4 3 2 1 ppm
1.00
1.00
1.17
1.02
1.02
2.10
2.01

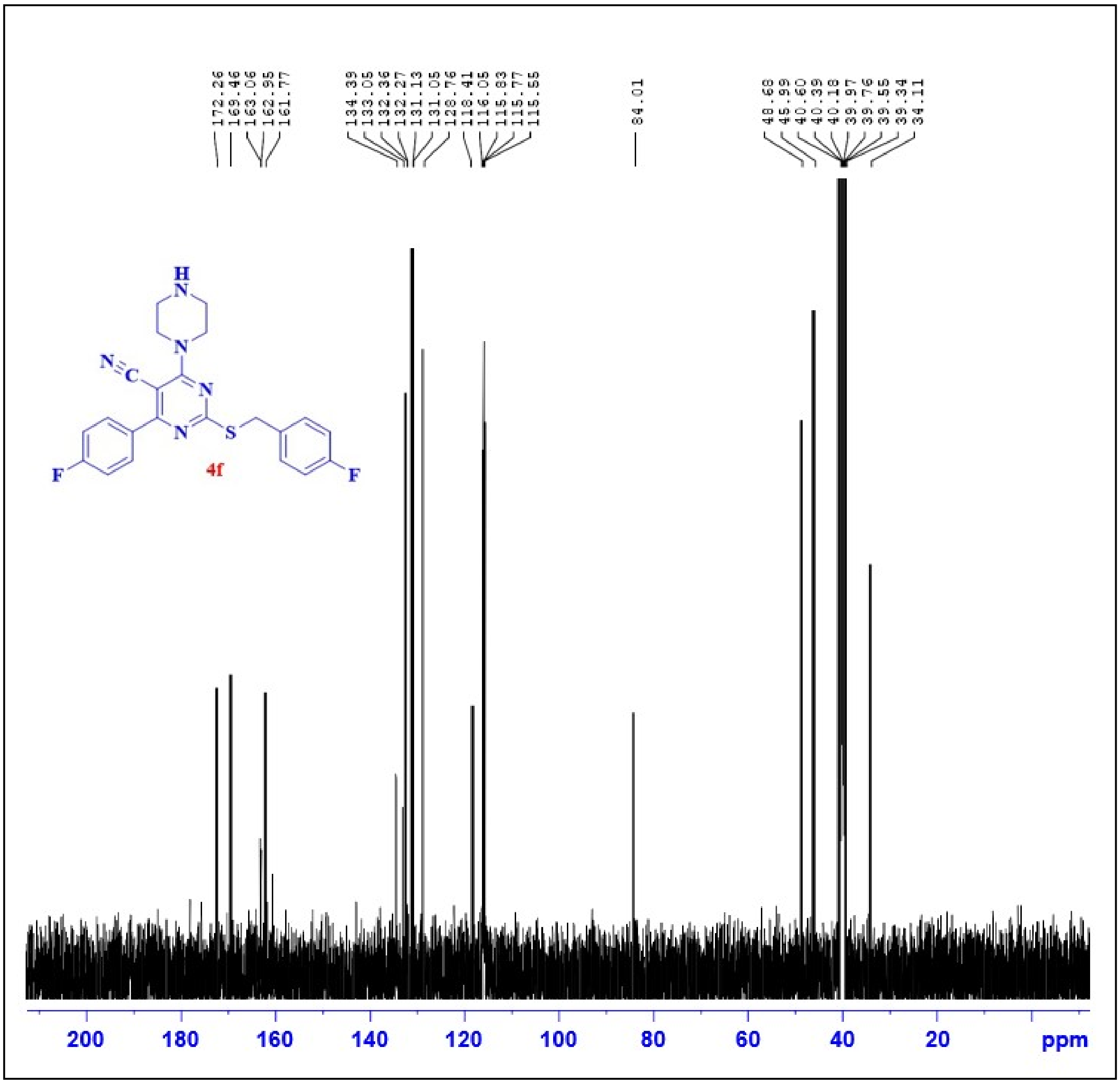
172.26
169.46
163.06
162.95
161.77
134.39
133.05
132.36
132.27
131.13
131.05
128.76
118.41
116.05
115.83
115.77
115.55
84.01
48.68
45.99
40.60
40.39
40.18
39.97
39.76
39.55
39.34
34.11
H
N
N
N
C
N
N
S
F
F
4f
200
180
160
140
120
100
80
60
40
20
ppm

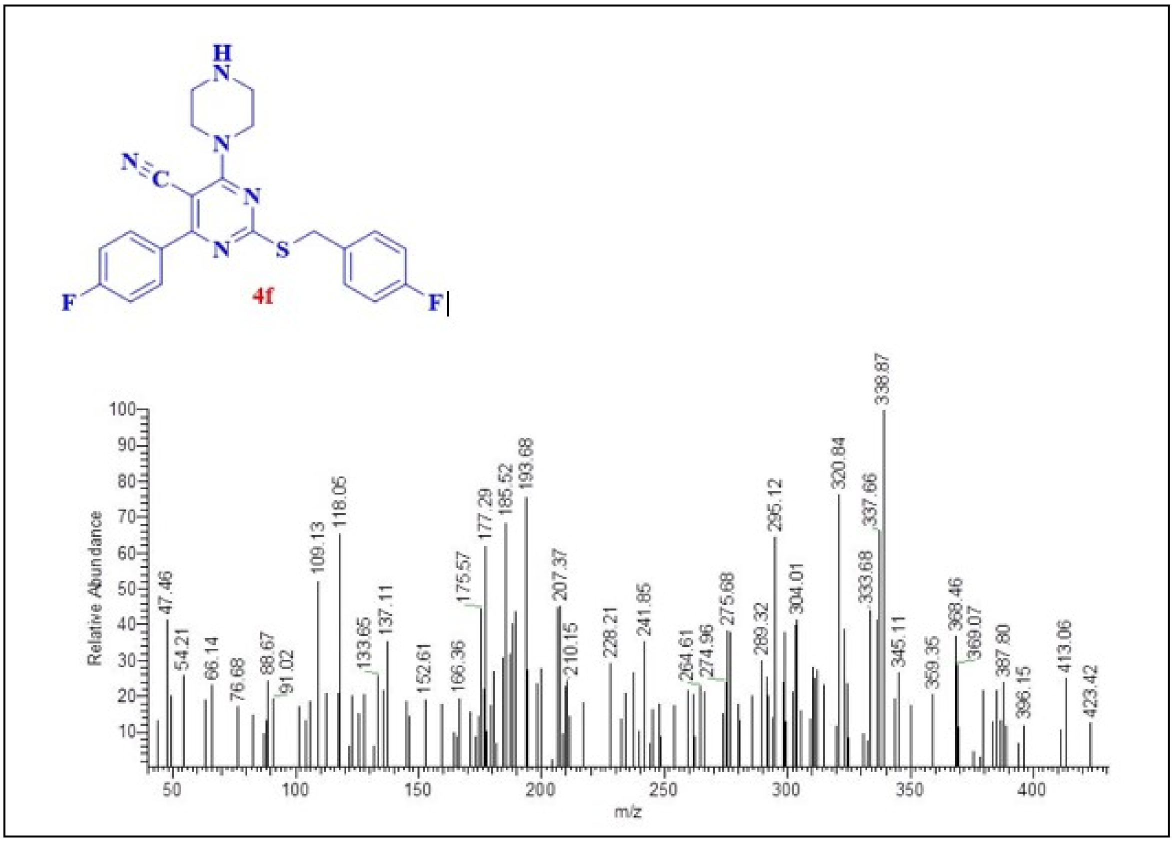

4f
Relative Abundance
m/z
338.87
320.84
337.66
193.68
185.52
177.29
118.05
295.12
109.13

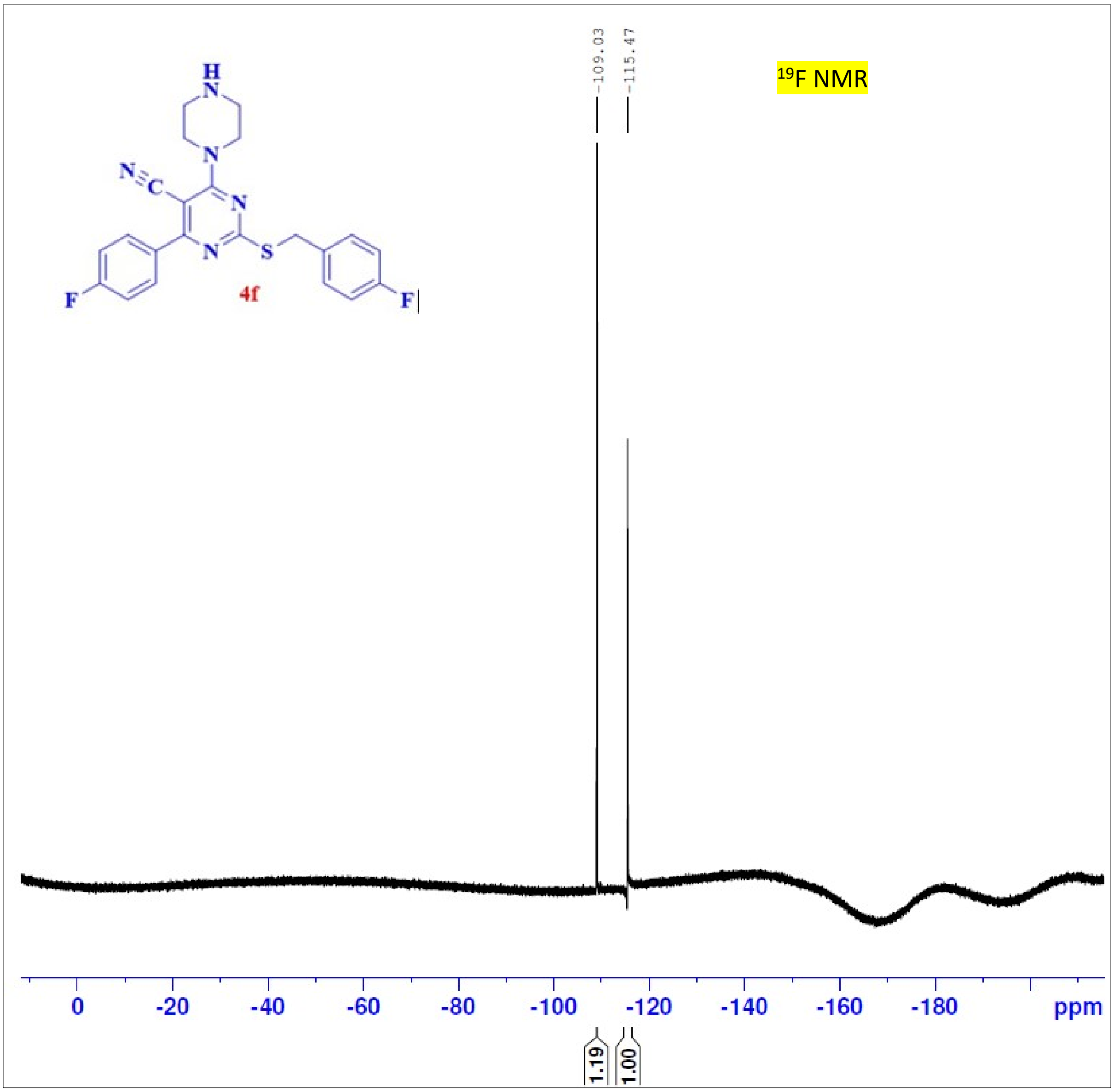

-109.03
-115.47
19F NMR
4f
0
-20
-40
-60
-80
-100
-120
-140
-160
-180
ppm
1.19
1.00

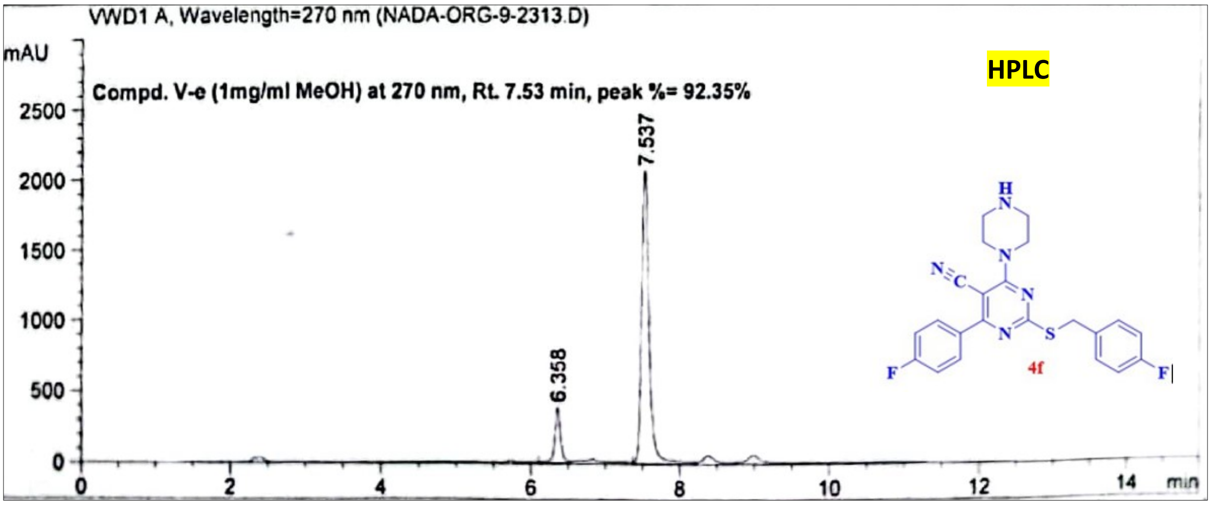

VWD1 A, Wavelength=270 nm (NADA-ORG-9-2313.D)
mAU
Compd. V-e (1mg/ml MeOH) at 270 nm, Rt. 7.53 min, peak %= 92.35%
HPLC
7.537
6.358
4f
min

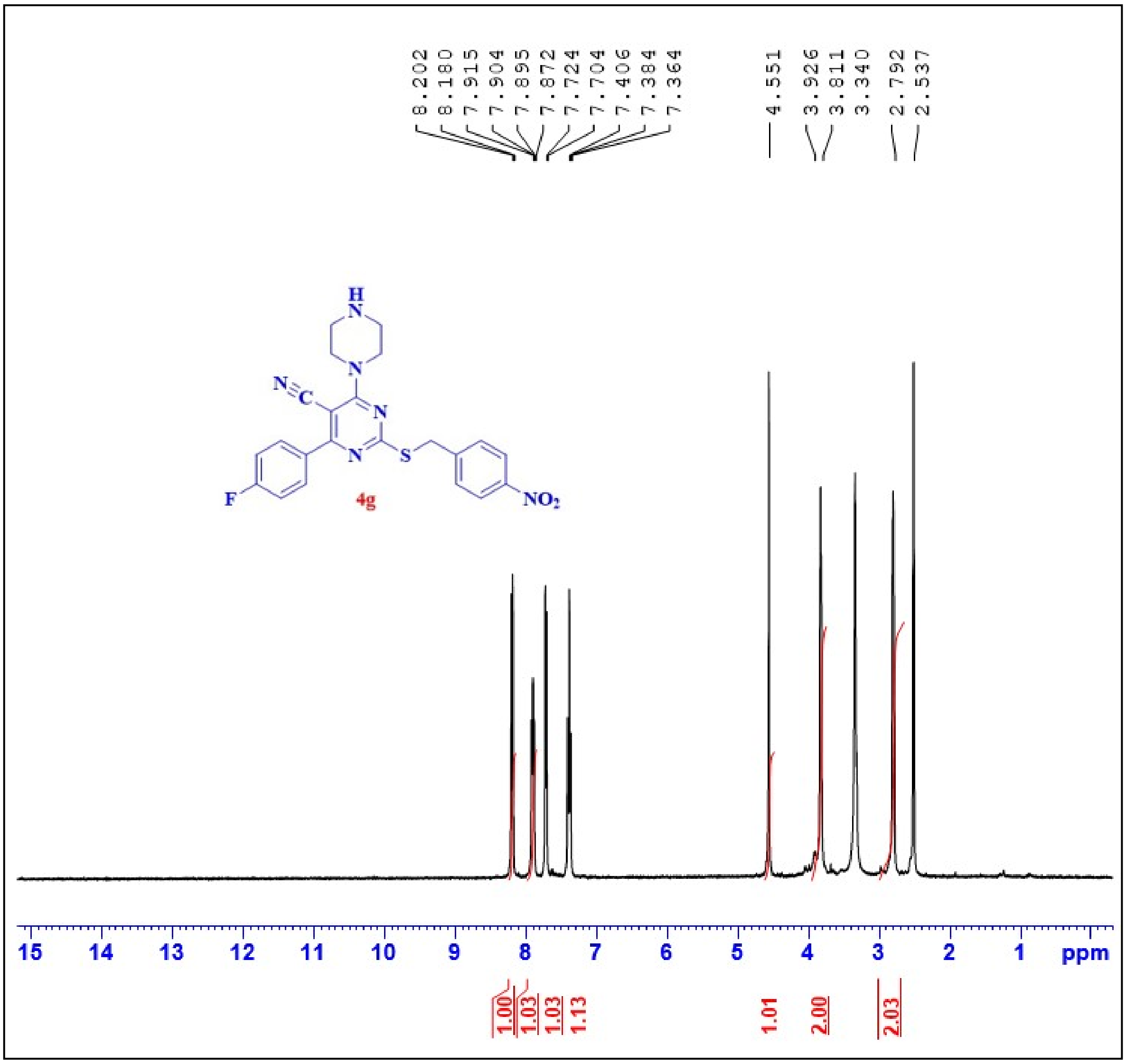
8.202
8.180
7.915
7.904
7.895
7.872
7.724
7.704
7.406
7.384
7.364
4.551
3.926
3.811
3.340
2.792
2.537
N
H
N
N
N
S
F
NO2
4g
15 14 13 12 11 10 9 8 7 6 5 4 3 2 1 ppm
1.00
1.03
1.03
1.13
1.01
2.00
2.03

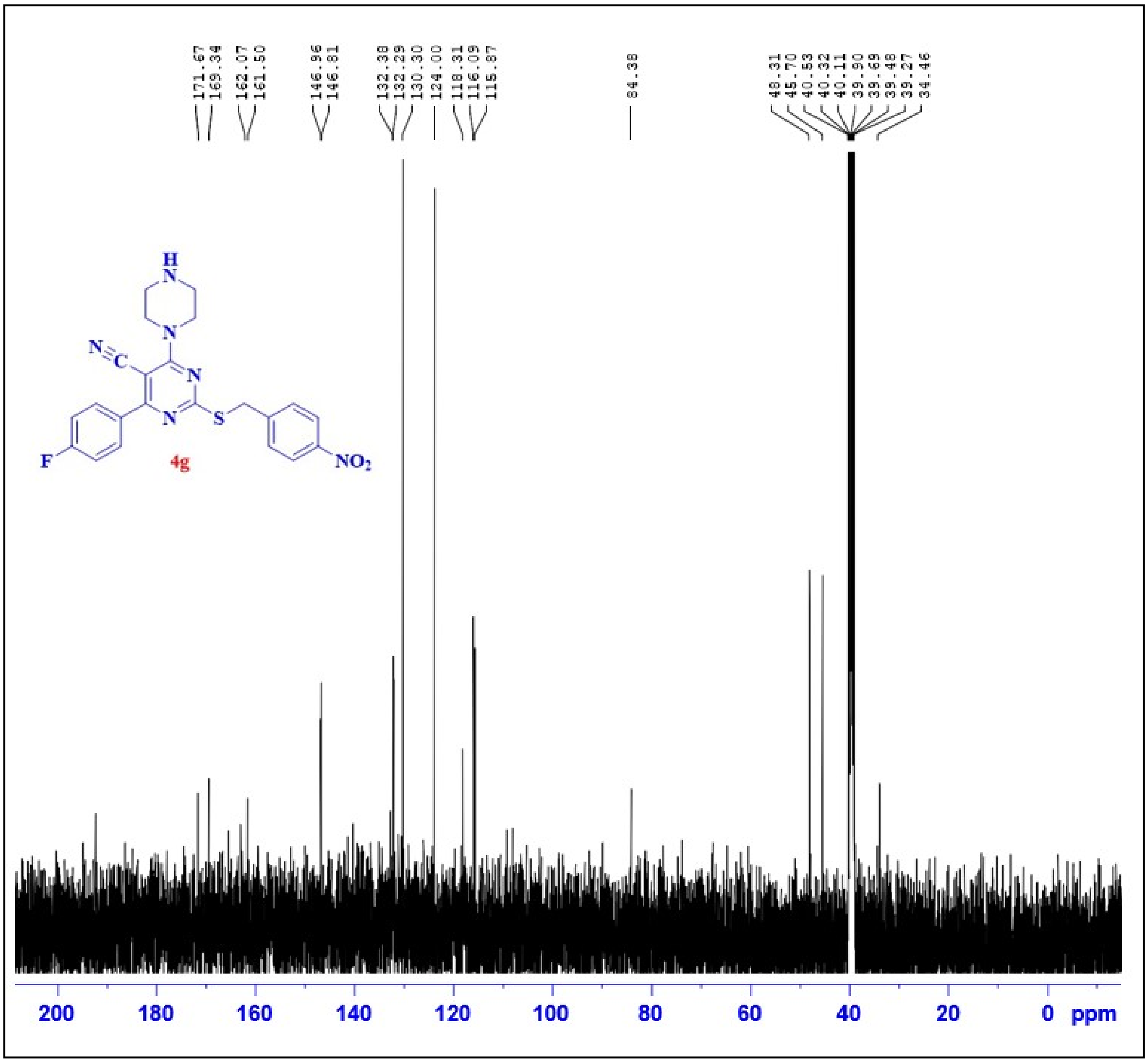
171.67
169.34
162.07
161.50
146.96
146.81
132.38
132.29
130.30
124.00
118.31
116.09
115.87
84.38
48.31
45.70
40.53
40.32
40.11
39.90
39.69
39.48
39.27
34.46
H
N
N
C
N
N
S
F
NO_2
4g
200
180
160
140
120
100
80
60
40
20
0
ppm

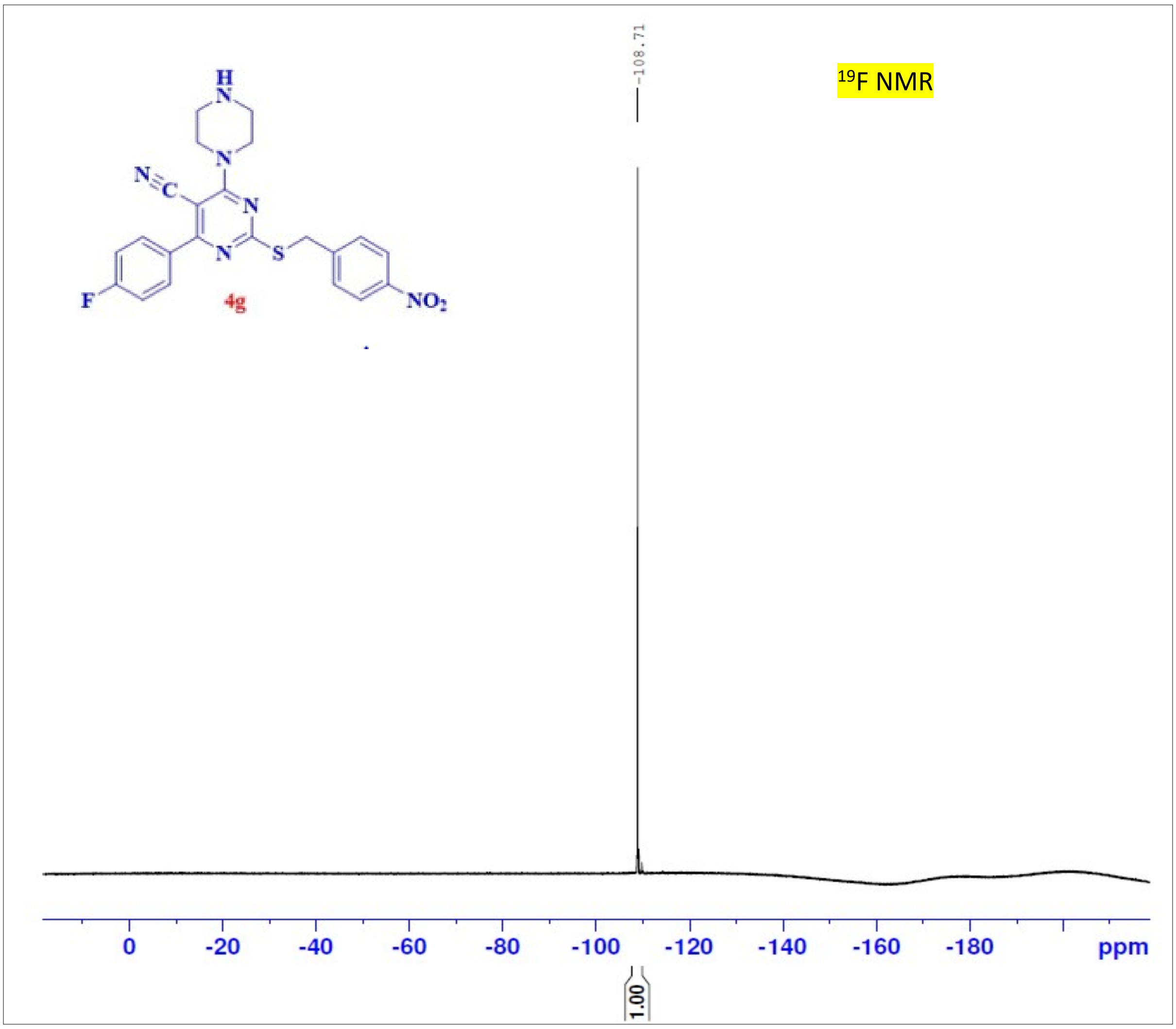
-108.71
19F NMR
4g
NO2
F
0
-20
-40
-60
-80
-100
-120
-140
-160
-180
ppm
1.00

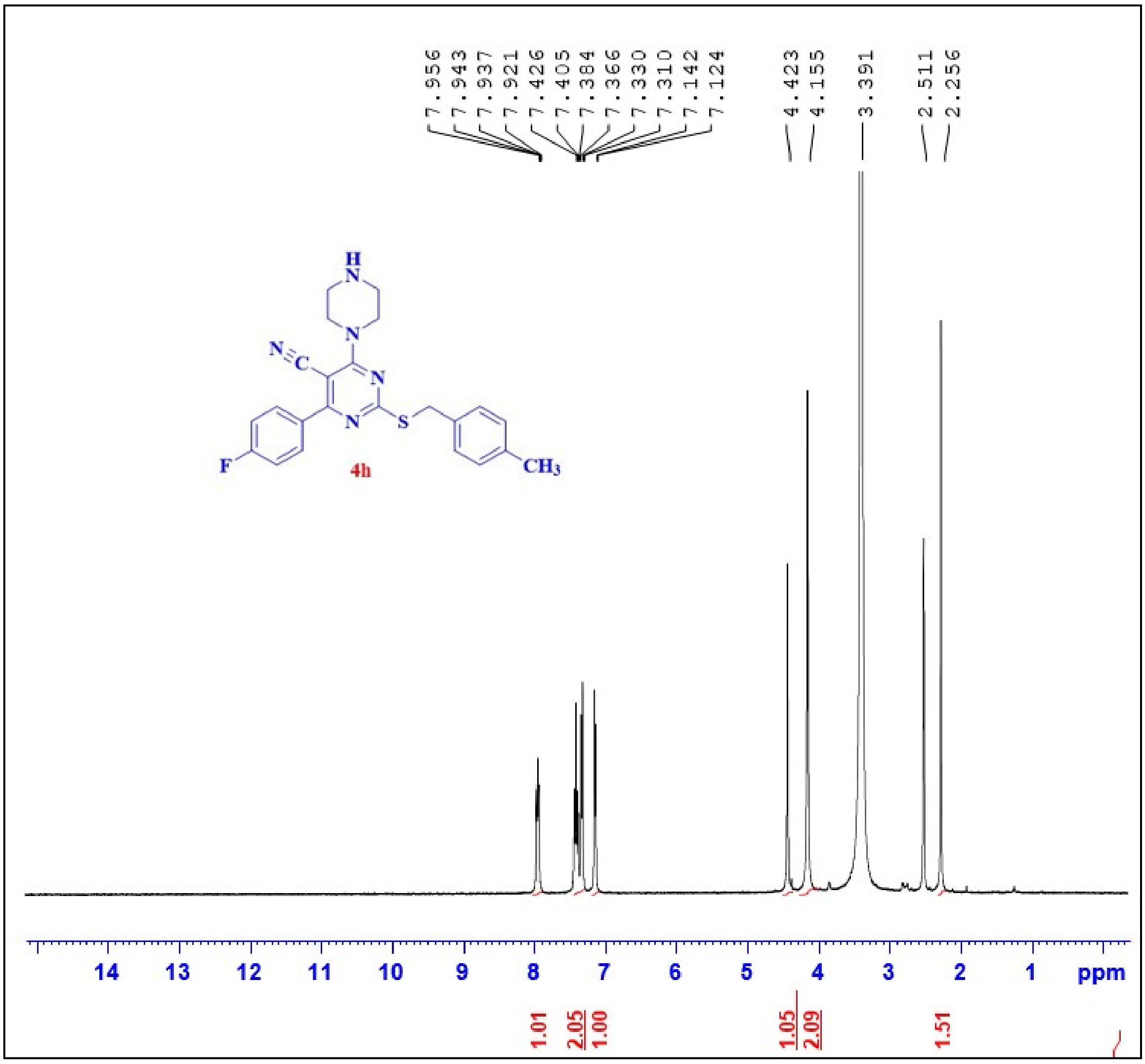
7.956
7.943
7.937
7.921
7.426
7.405
7.384
7.366
7.330
7.310
7.142
7.124
4.423
4.155
3.391
2.511
2.256
H
N
N
N
C
N
N
S
F
CH3
4h
14 13 12 11 10 9 8 7 6 5 4 3 2 1 ppm
1.01
2.05
1.00
1.05
2.09
1.51

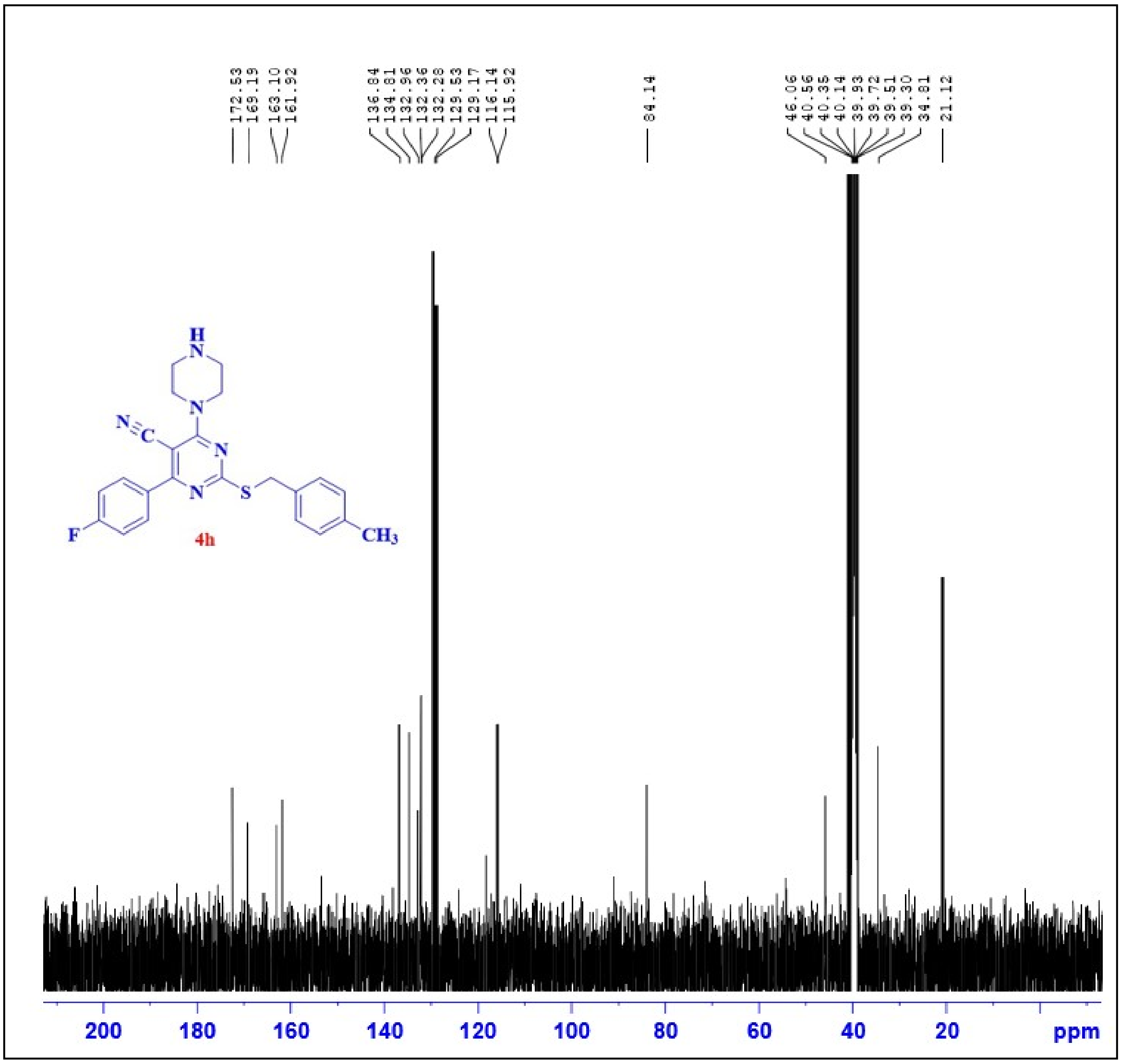
172.53
169.19
163.10
161.92
136.84
134.81
132.96
132.36
132.28
129.53
129.17
116.14
115.92
84.14
46.06
40.56
40.35
40.14
39.93
39.72
39.51
39.30
34.81
21.12
N
H
N
N
C
N
N
S
F
CH3
4h
200
180
160
140
120
100
80
60
40
20
ppm

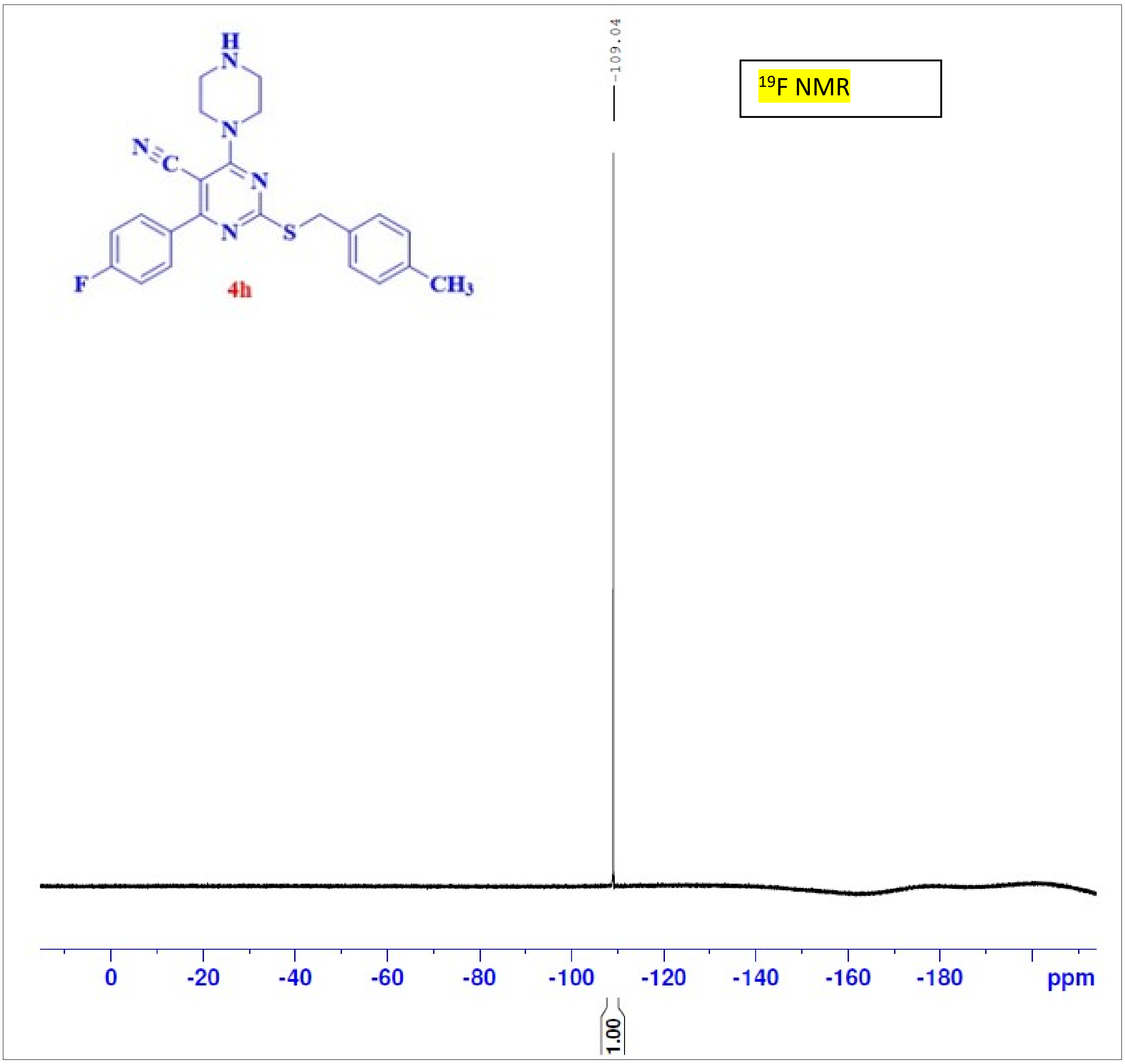

-109.04
19F NMR
4h
1.00
0
-20
-40
-60
-80
-100
-120
-140
-160
-180
ppm

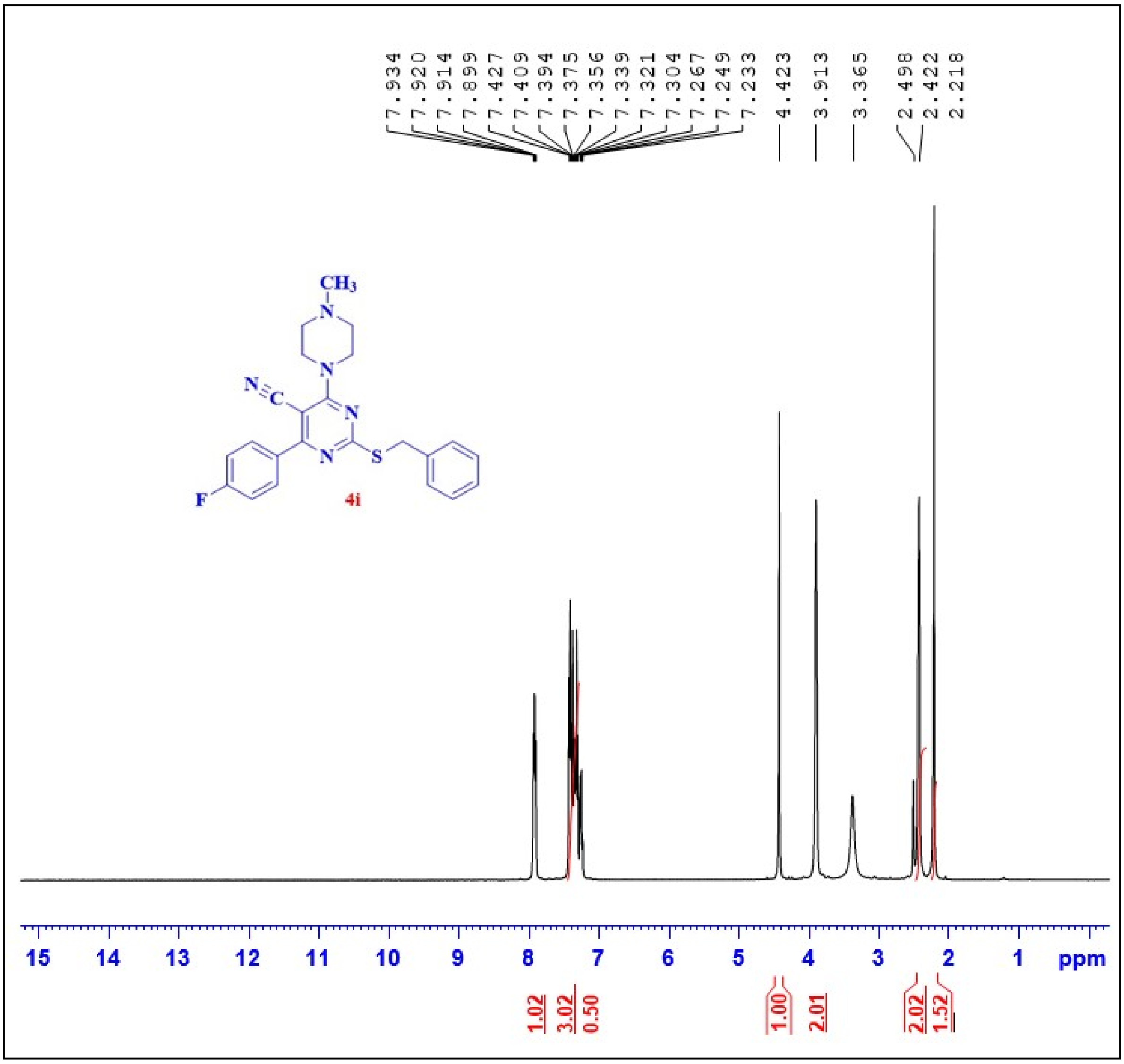
7.934
7.920
7.914
7.899
7.427
7.409
7.394
7.375
7.356
7.339
7.321
7.304
7.267
7.249
7.233
4.423
3.913
3.365
2.498
2.422
2.218
CH3
N
N
N
C
N
N
S
F
4i
15
14
13
12
11
10
9
8
7
6
5
4
3
2
1
ppm
1.02
3.02
0.50
1.00
2.01
2.02
1.52

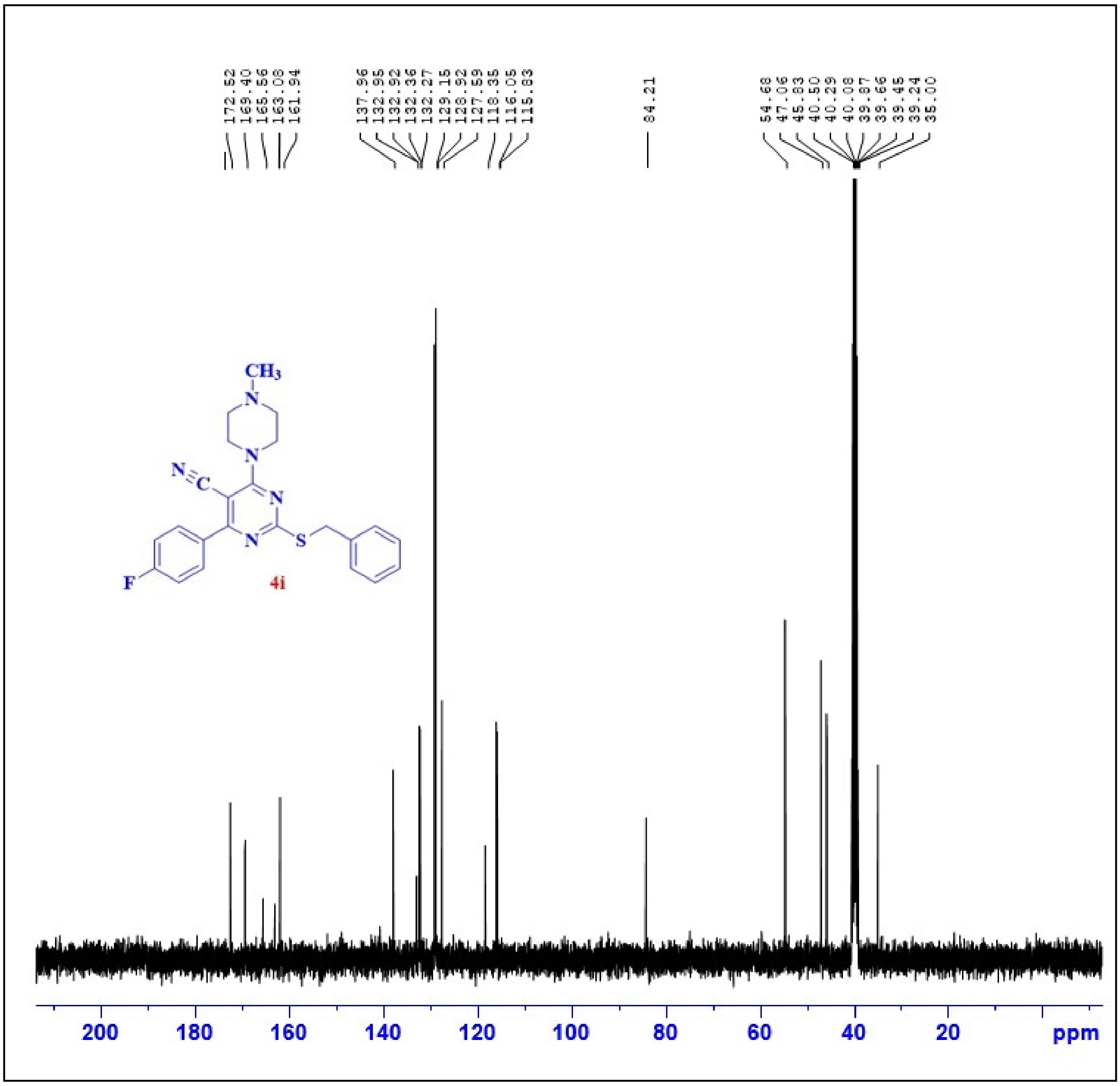
172.52
169.40
165.56
163.08
161.94
137.96
132.95
132.92
132.36
132.27
129.15
128.92
127.59
118.35
116.05
115.83
84.21
54.68
47.06
45.83
40.50
40.29
40.08
39.87
39.66
39.45
39.24
35.00
CH3
N
N
N≡C
N
N
S
F
4i
200
180
160
140
120
100
80
60
40
20
ppm

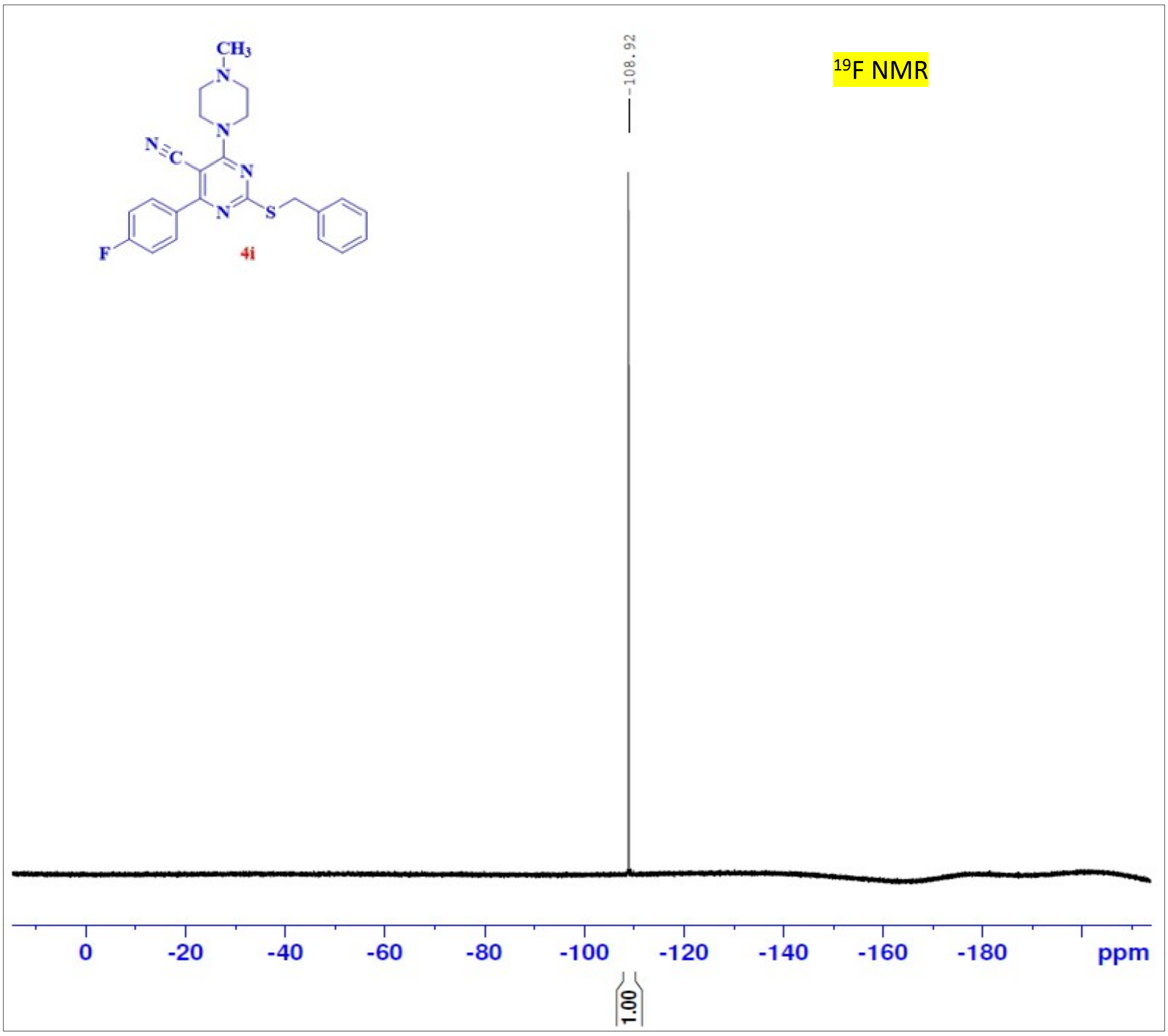

-108.92
19F NMR
CH3
N
N
N
C
N
N
S
F
4i
0
-20
-40
-60
-80
-100
-120
-140
-160
-180
ppm
1.00

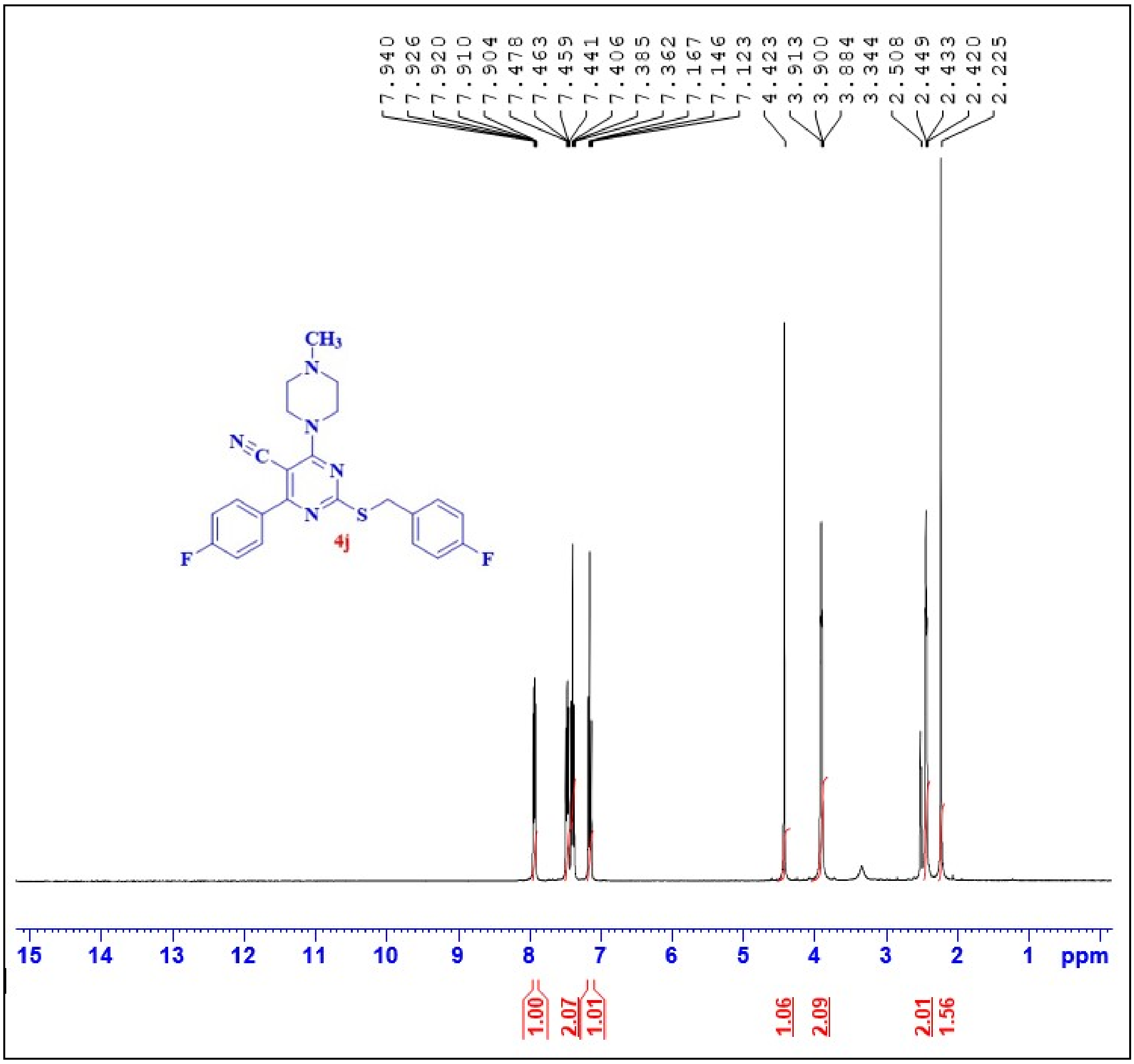

7.940
7.926
7.920
7.910
7.904
7.478
7.463
7.459
7.441
7.406
7.385
7.362
7.167
7.146
7.123
4.423
3.913
3.900
3.884
3.344
2.508
2.449
2.433
2.420
2.225
CH3
N
N
N
C
N
N
S
F
F
4j
15
14
13
12
11
10
9
8
7
6
5
4
3
2
1
ppm
1.00
2.07
1.01
1.06
2.09
2.01
1.56

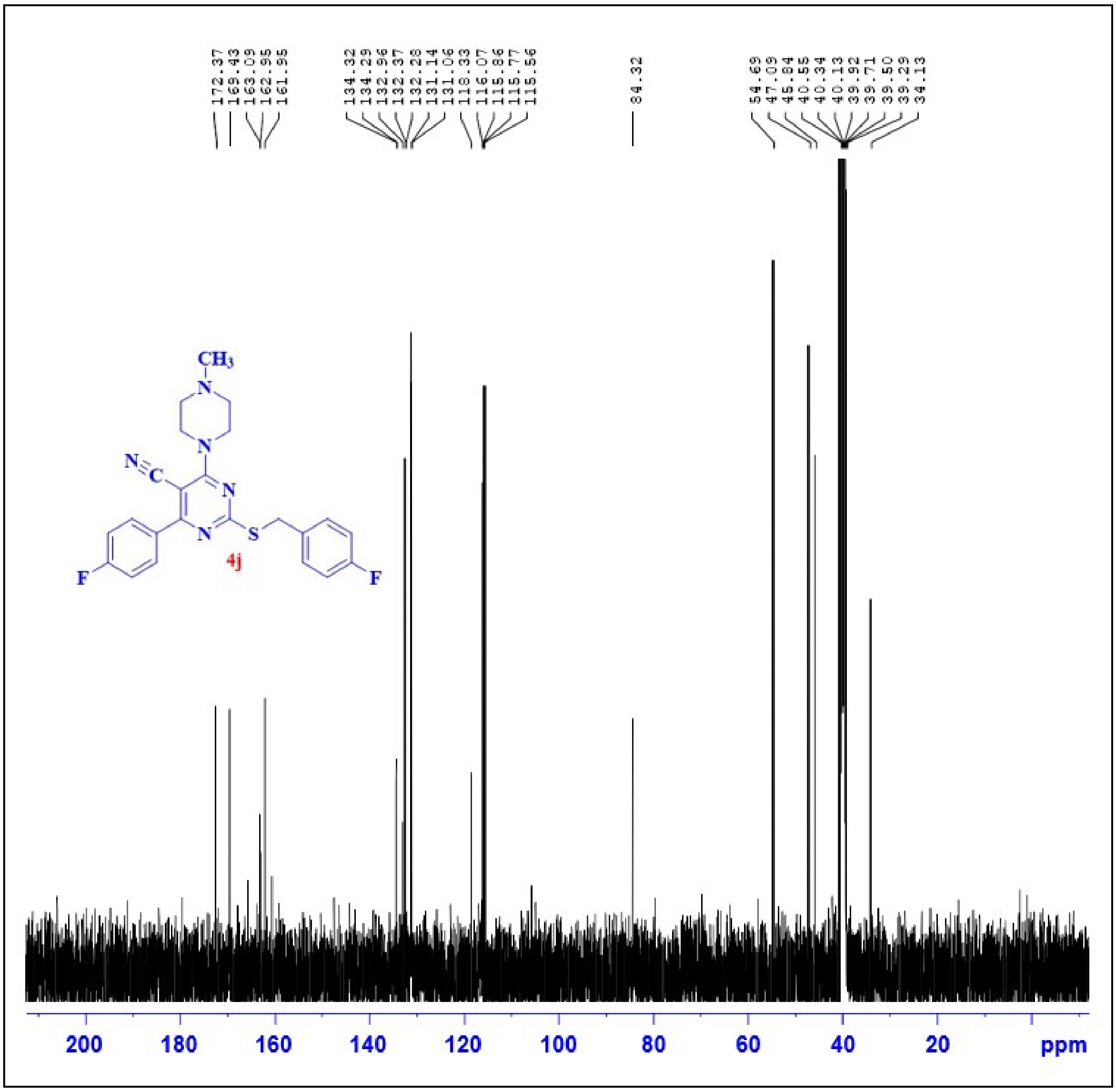

172.37
169.43
163.09
162.95
161.95
134.32
134.29
132.96
132.37
132.28
131.14
131.06
118.33
116.07
115.86
115.77
115.56
84.32
54.69
47.09
45.84
40.55
40.34
40.13
39.92
39.71
39.50
39.29
34.13
CH3
N
N
N≡C
N
N
S
F
F
4j
200
180
160
140
120
100
80
60
40
20
ppm

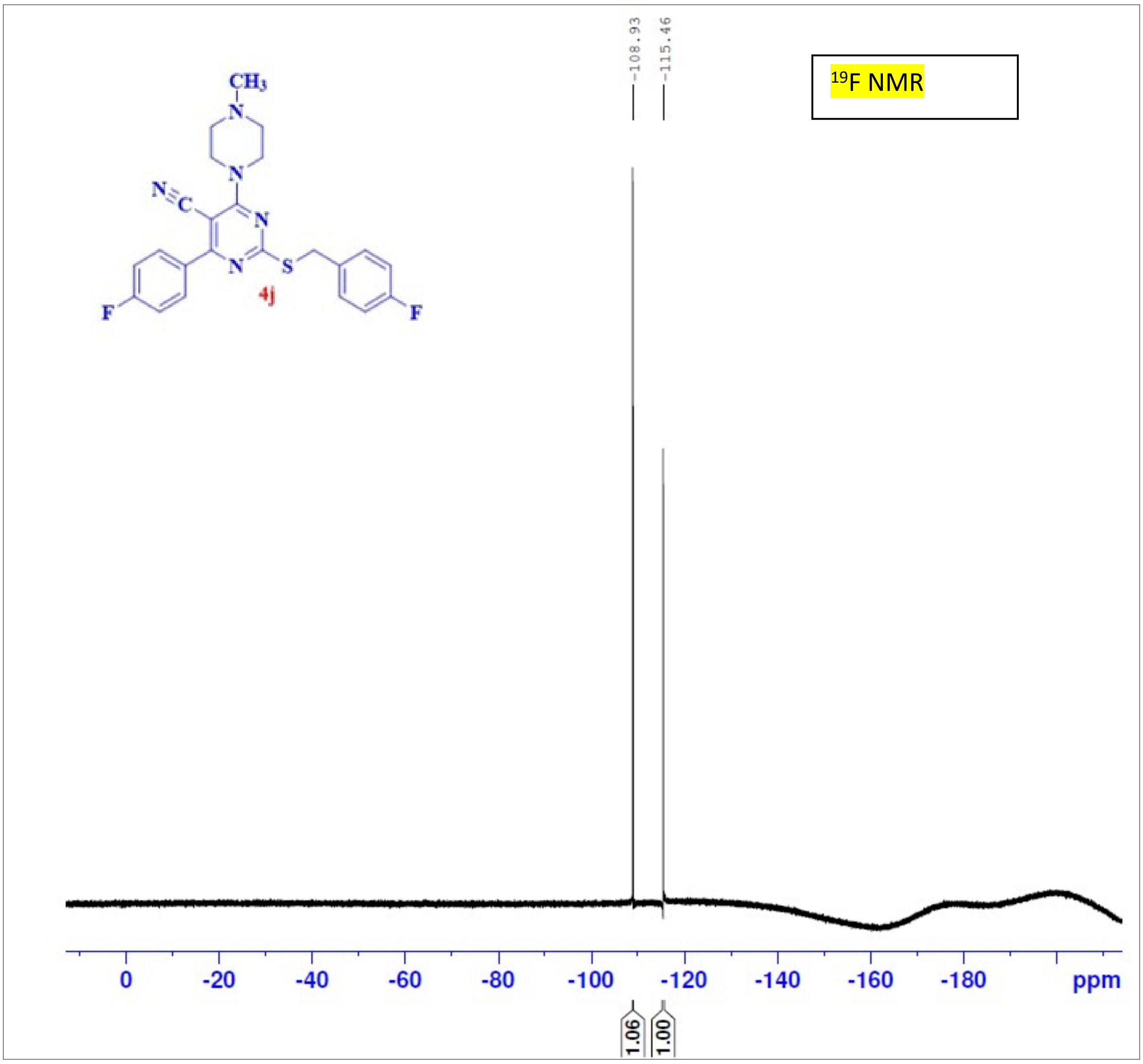

19F NMR
4j
-108.93
-115.46
0
-20
-40
-60
-80
-100
-120
-140
-160
-180
ppm
1.06
1.00

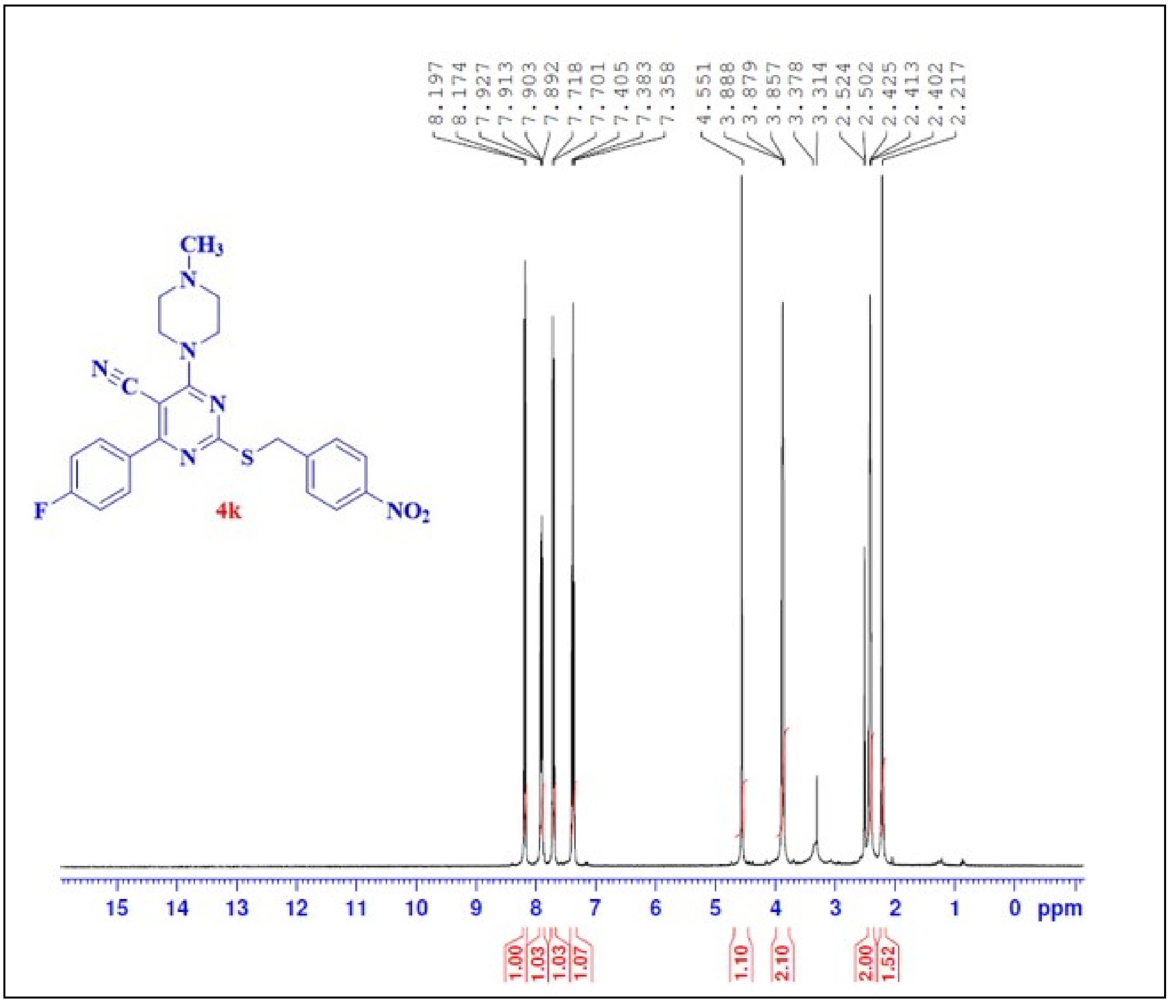

8.197
8.174
7.927
7.913
7.903
7.892
7.718
7.701
7.405
7.383
7.358
4.551
3.888
3.879
3.857
3.378
3.314
2.524
2.502
2.425
2.413
2.402
2.217
CH3
N
N
N
C
N
N
S
F
4k
NO2
15 14 13 12 11 10 9 8 7 6 5 4 3 2 1 0 ppm
1.00
1.03
1.03
1.07
1.10
2.10
2.00
1.52

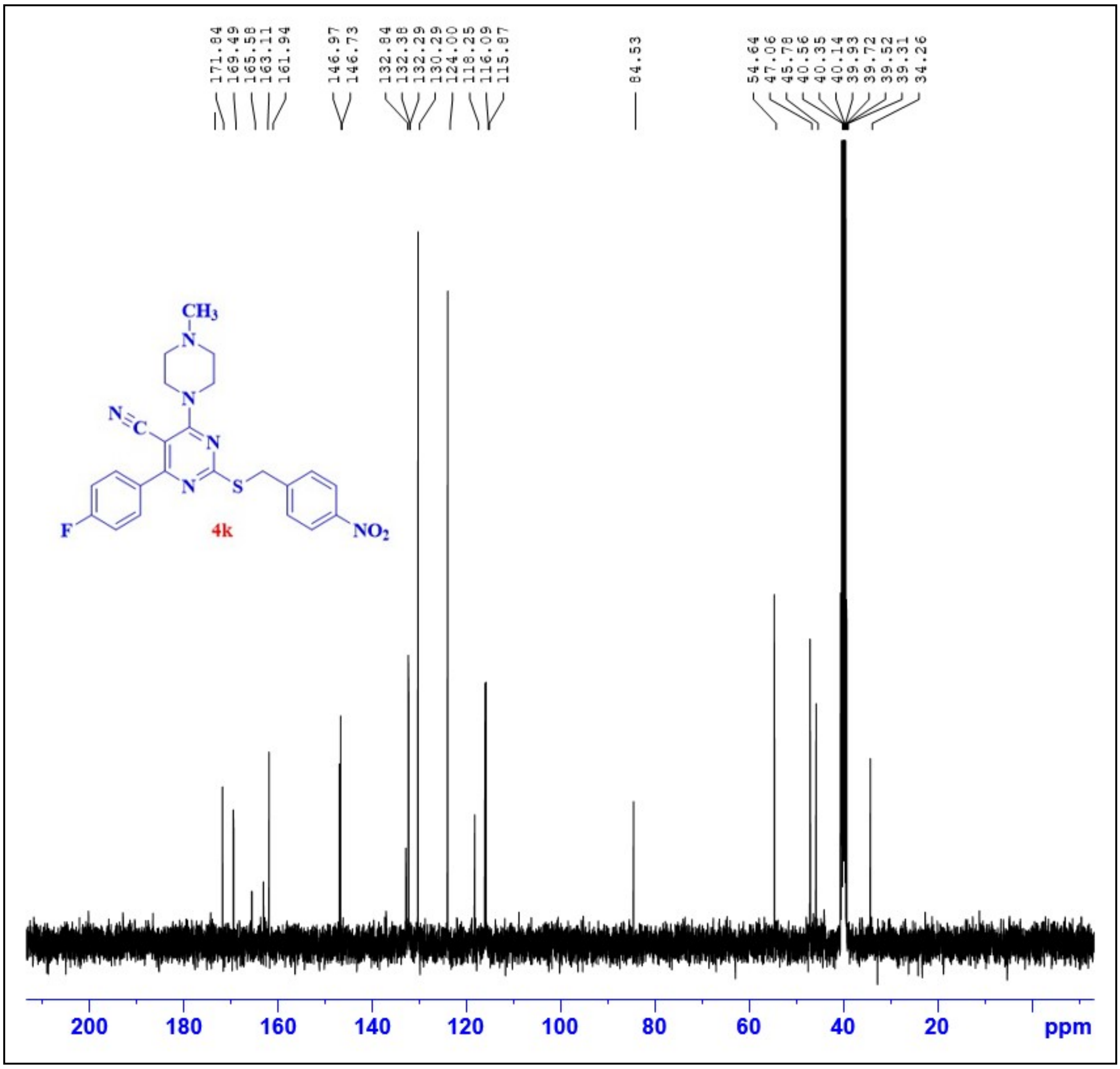
171.84
169.49
165.58
163.11
161.94
146.97
146.73
132.84
132.38
132.29
130.29
124.00
118.25
116.09
115.87
84.53
54.64
47.06
45.78
40.56
40.35
40.14
39.93
39.72
39.52
39.31
34.26
CH3
N
N
N
C
N
N
S
F
NO2
4k
200
180
160
140
120
100
80
60
40
20
ppm

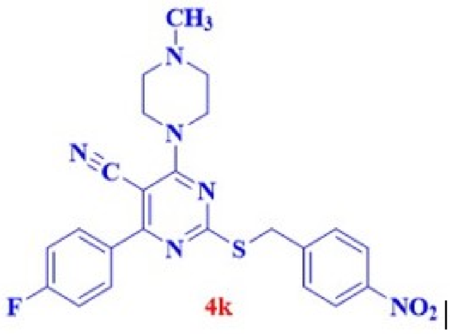
CH3
N
N
N
C
N
N
S
F
4k
NO2

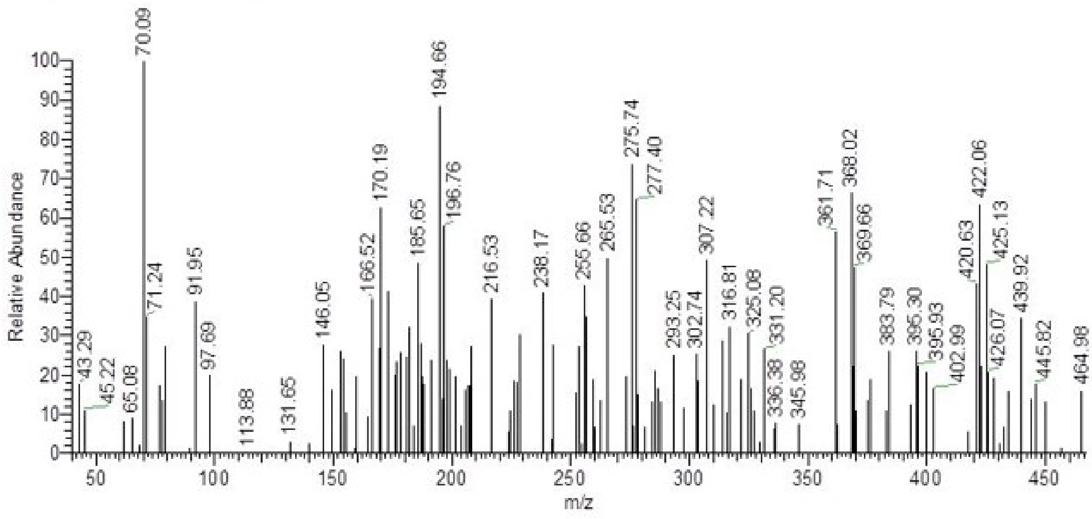
Relative Abundance
m/z
70.09
194.66
170.19
196.76
275.74
277.40
368.02
361.71
369.66
422.06
425.13
420.63
439.92
43.29
45.22
65.08
71.24
91.95
97.69
113.88
131.65
146.05
166.52
185.65
216.53
238.17
255.66
265.53
293.25
302.74
307.22
316.81
325.08
331.20
336.38
345.98
383.79
395.30
395.93
402.99
426.07
445.82
464.98

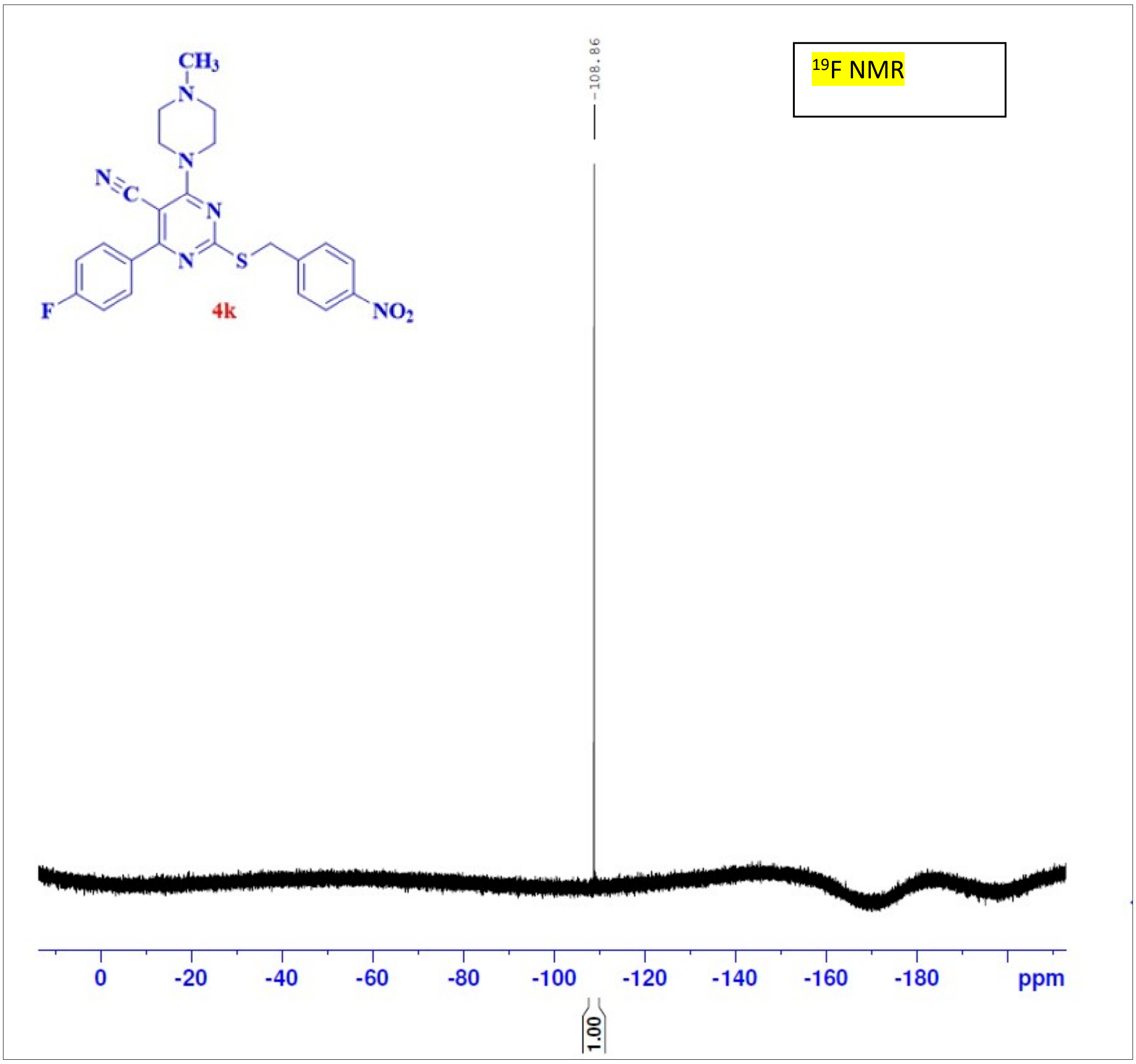

CH3
N
N
N
C
N
N
S
F
4k
NO2
-108.86
19F NMR
0
-20
-40
-60
-80
-100
-120
-140
-160
-180
ppm
1.00

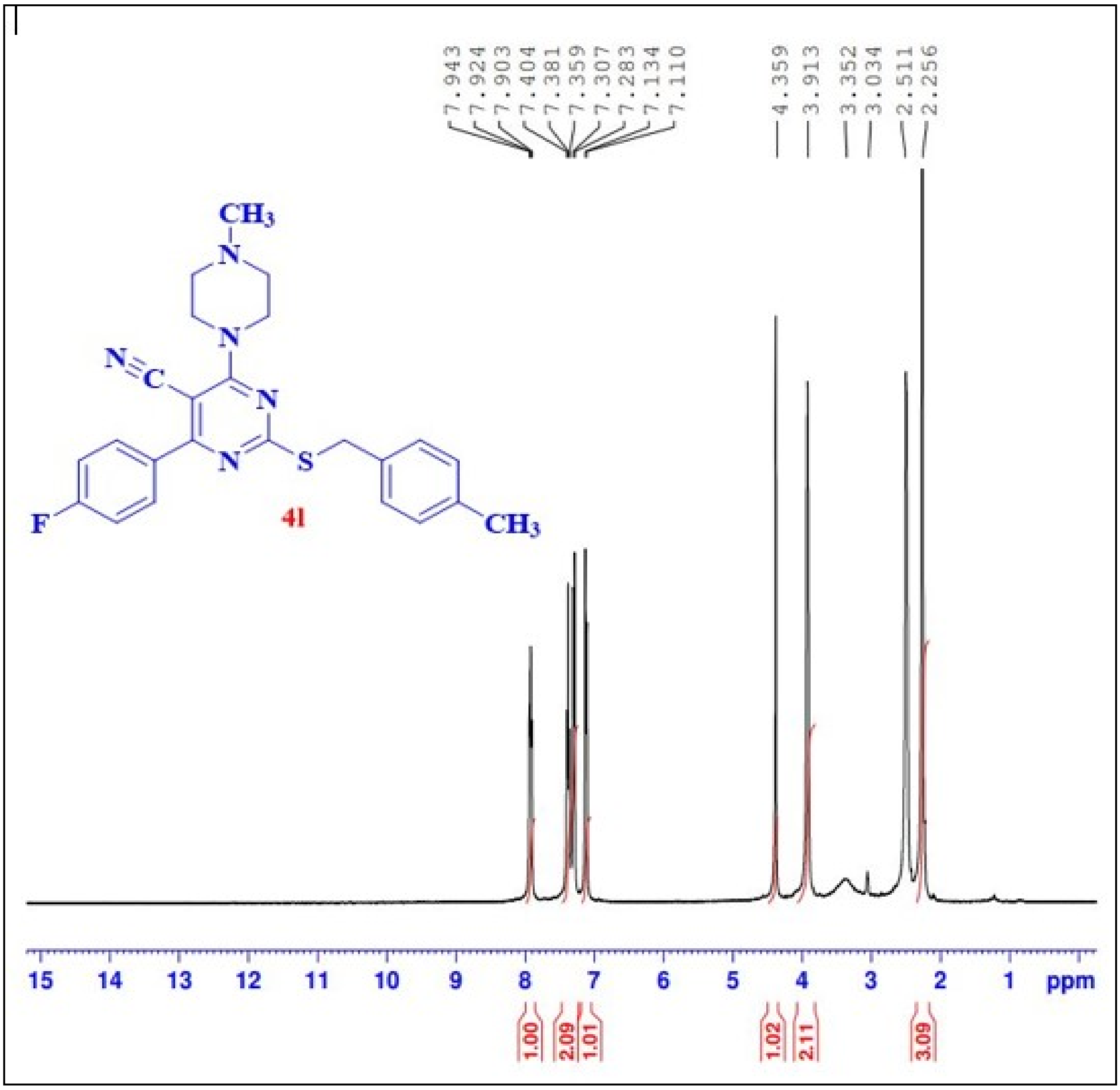

7.943
7.924
7.903
7.404
7.381
7.359
7.307
7.283
7.134
7.110
4.359
3.913
3.352
3.034
2.511
2.256
CH3
N
N
N≡C
N
N
S
F
CH3
4l
15 14 13 12 11 10 9 8 7 6 5 4 3 2 1 ppm
1.00
2.09
1.01
1.02
2.11
3.09

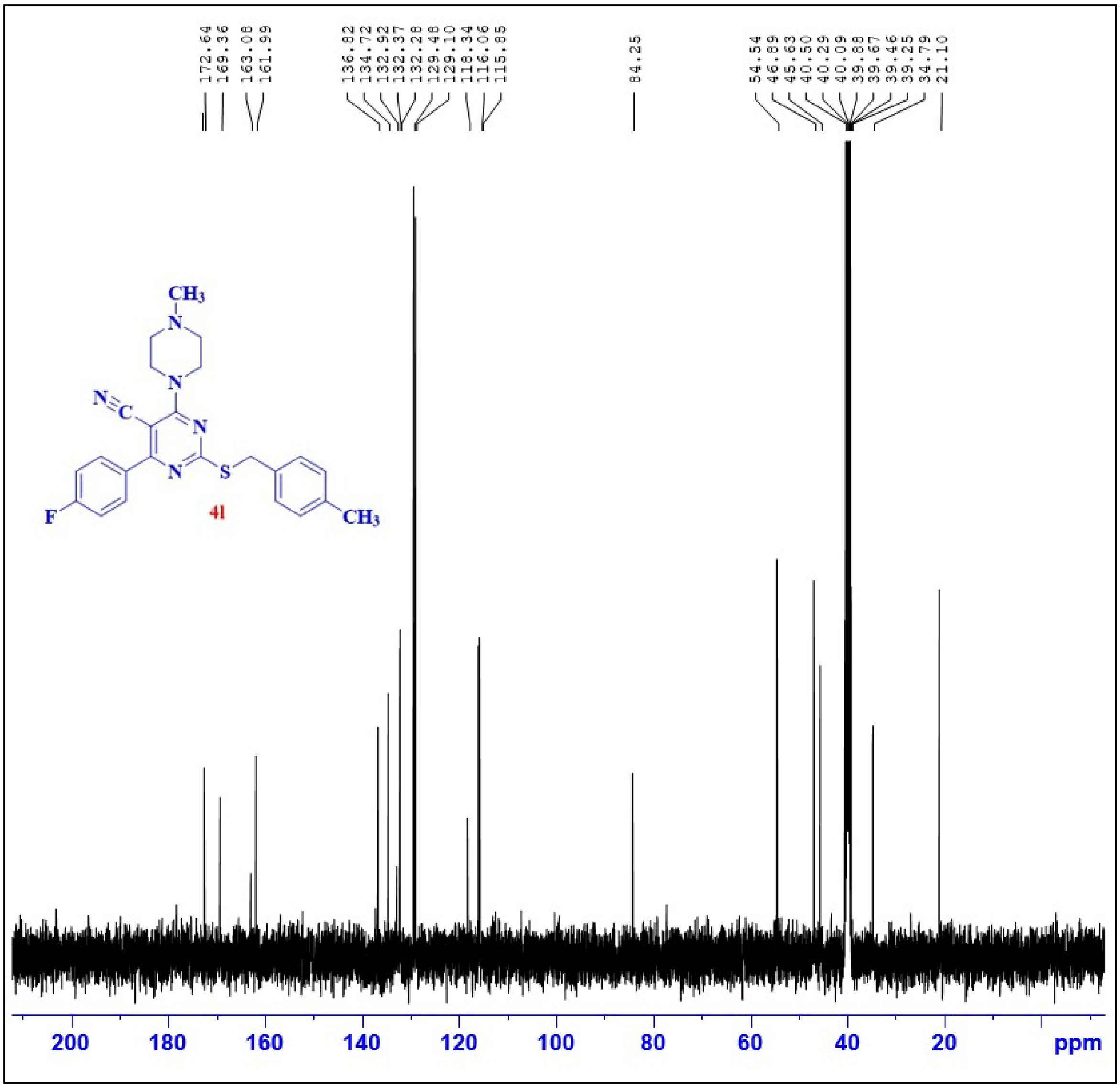
172.64
169.36
163.08
161.99
136.82
134.72
132.92
132.37
132.28
129.40
129.10
118.34
116.06
115.85
84.25
54.54
46.89
45.63
40.50
40.29
40.09
39.88
39.67
39.46
39.25
34.79
21.10
CH3
N
N
N
C
N
N
S
F
CH3
4l
200
180
160
140
120
100
80
60
40
20
ppm

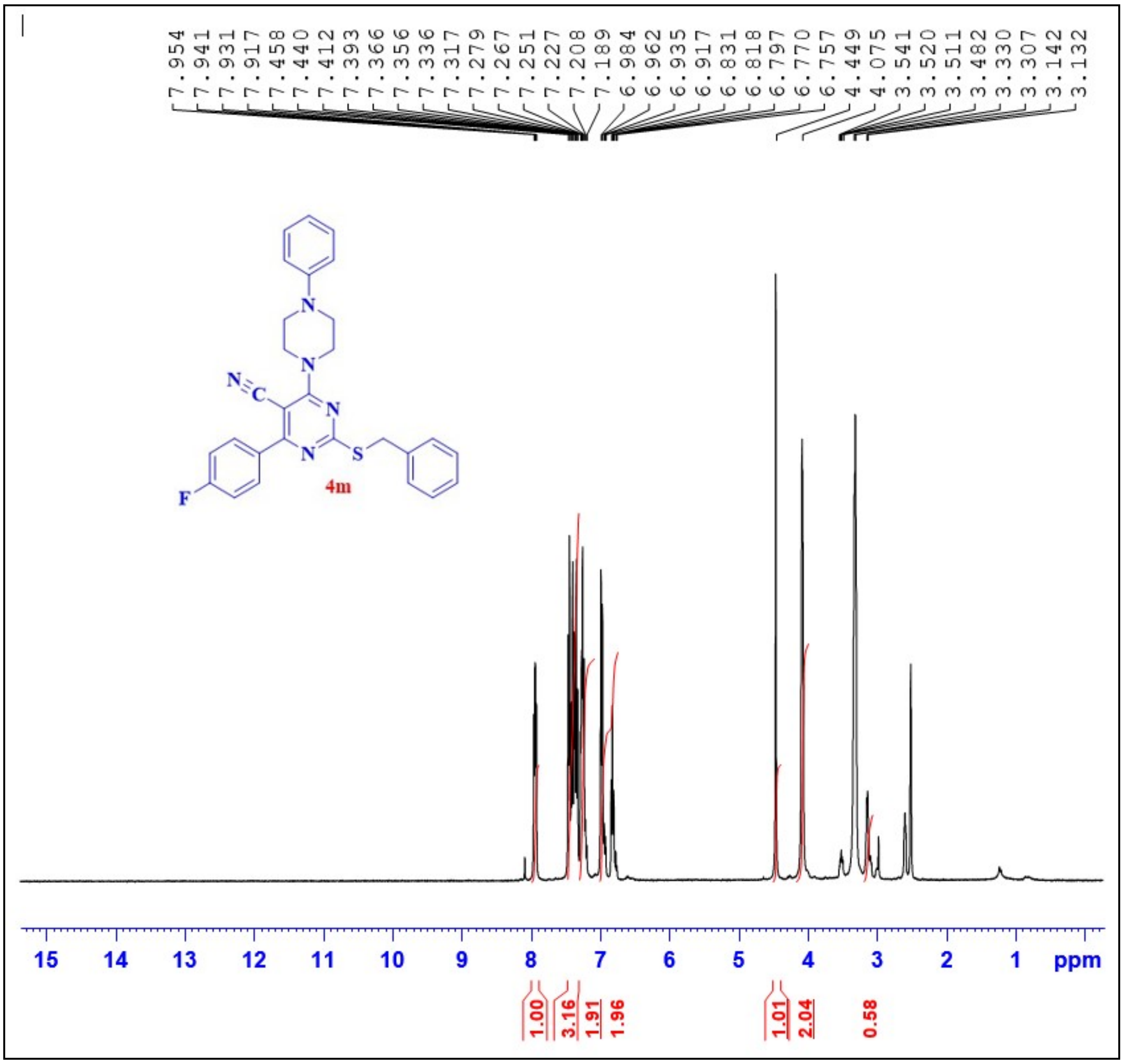
7.954
7.941
7.931
7.917
7.458
7.440
7.412
7.393
7.366
7.356
7.336
7.317
7.279
7.267
7.251
7.227
7.208
7.189
6.984
6.962
6.935
6.917
6.831
6.818
6.797
6.770
6.757
4.449
4.075
3.541
3.520
3.511
3.482
3.330
3.307
3.142
3.132
N
N
N
N
N
C
S
F
4m
15 14 13 12 11 10 9 8 7 6 5 4 3 2 1 ppm
1.00
3.16
1.91
1.96
1.01
2.04
0.58

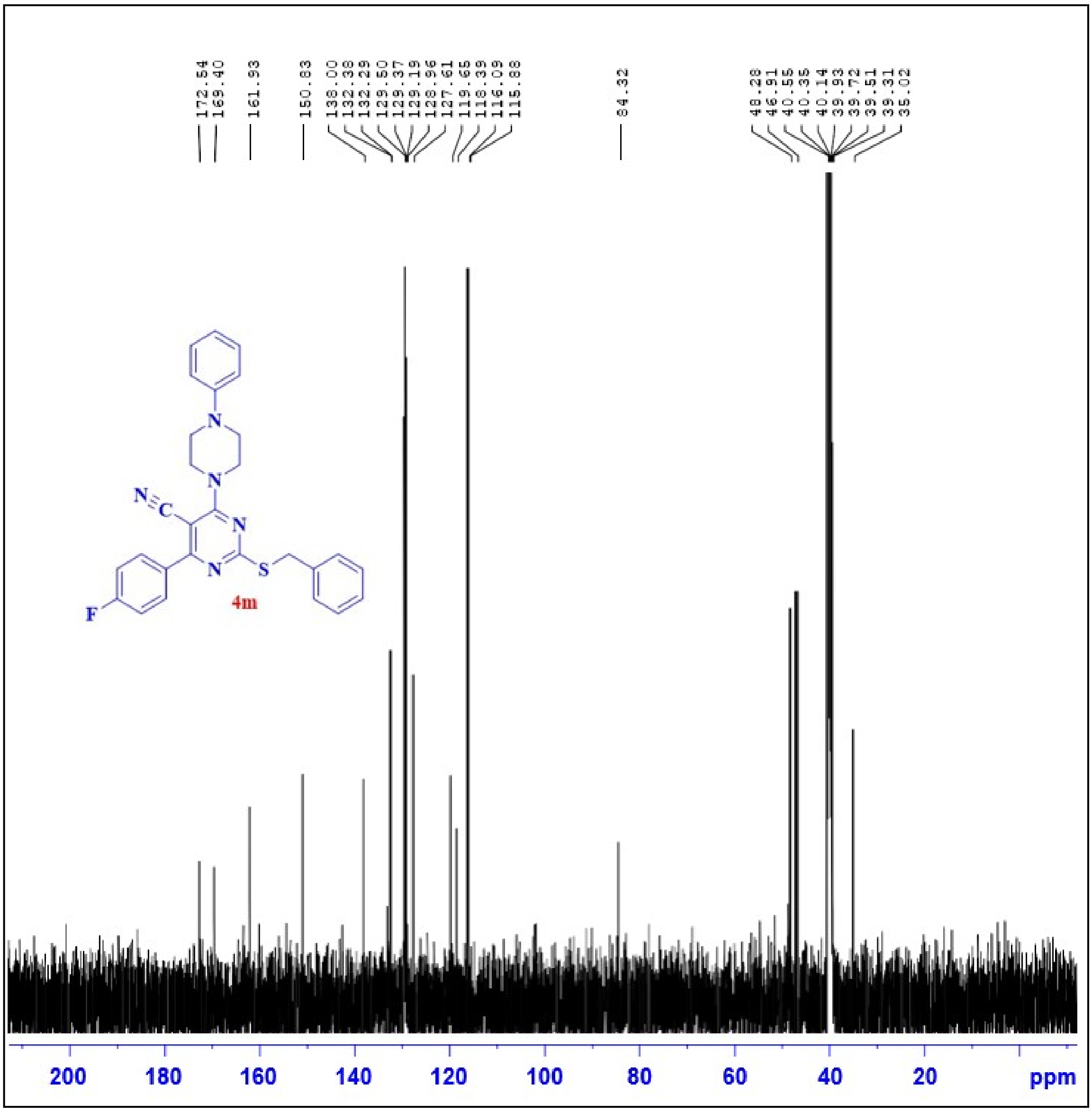

172.54
169.40
161.93
150.83
138.00
132.38
132.29
129.50
129.37
129.19
128.96
127.61
119.65
118.39
116.09
115.88
84.32
48.28
46.91
40.55
40.35
40.14
39.93
39.72
39.51
39.31
35.02
N
N
N
N
C
N
S
F
4m
200
180
160
140
120
100
80
60
40
20
ppm

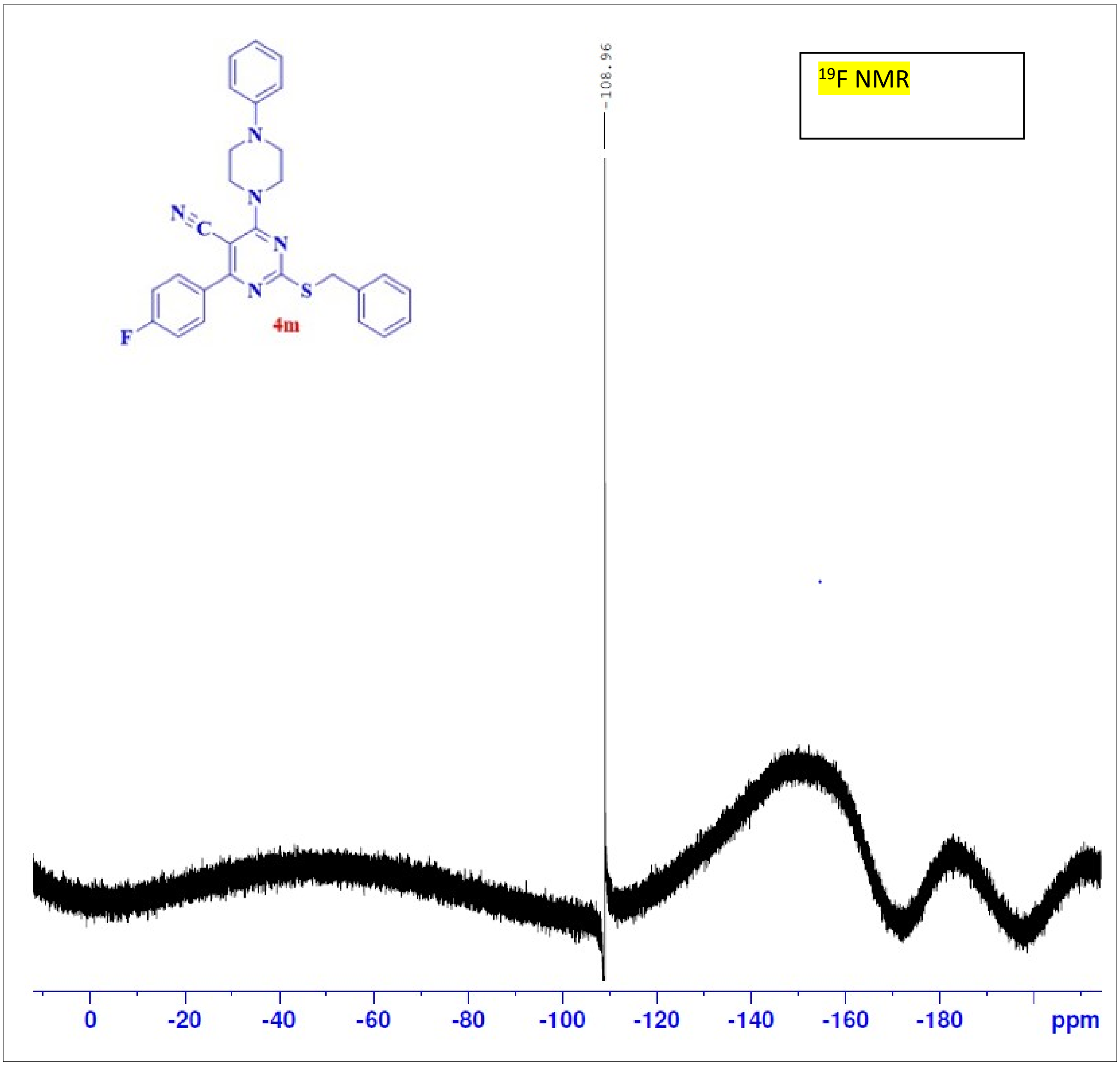

N
C
N
N
N
N
S
F
4m
-108.96
19F NMR
0
-20
-40
-60
-80
-100
-120
-140
-160
-180
ppm

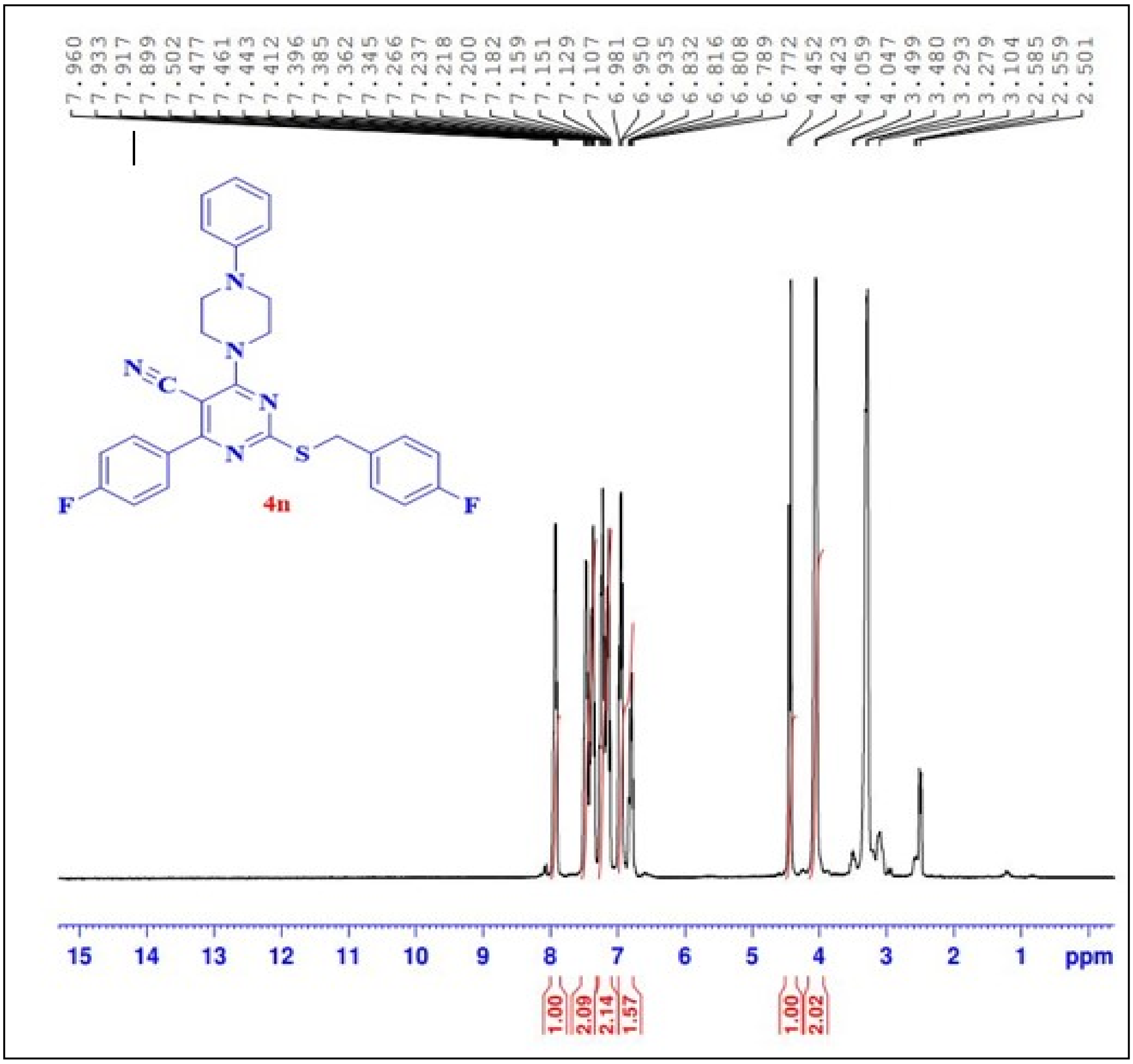
7.960
7.933
7.917
7.899
7.502
7.477
7.461
7.443
7.412
7.396
7.385
7.362
7.345
7.266
7.237
7.218
7.200
7.182
7.159
7.151
7.129
7.107
6.981
6.950
6.935
6.832
6.816
6.808
6.789
6.772
4.452
4.423
4.059
4.047
3.499
3.480
3.293
3.279
3.104
2.585
2.559
2.501
N
N
N≡C
N
N
S
F
F
4n
15 14 13 12 11 10 9 8 7 6 5 4 3 2 1 ppm
1.00
2.09
2.14
1.57
1.00
2.02

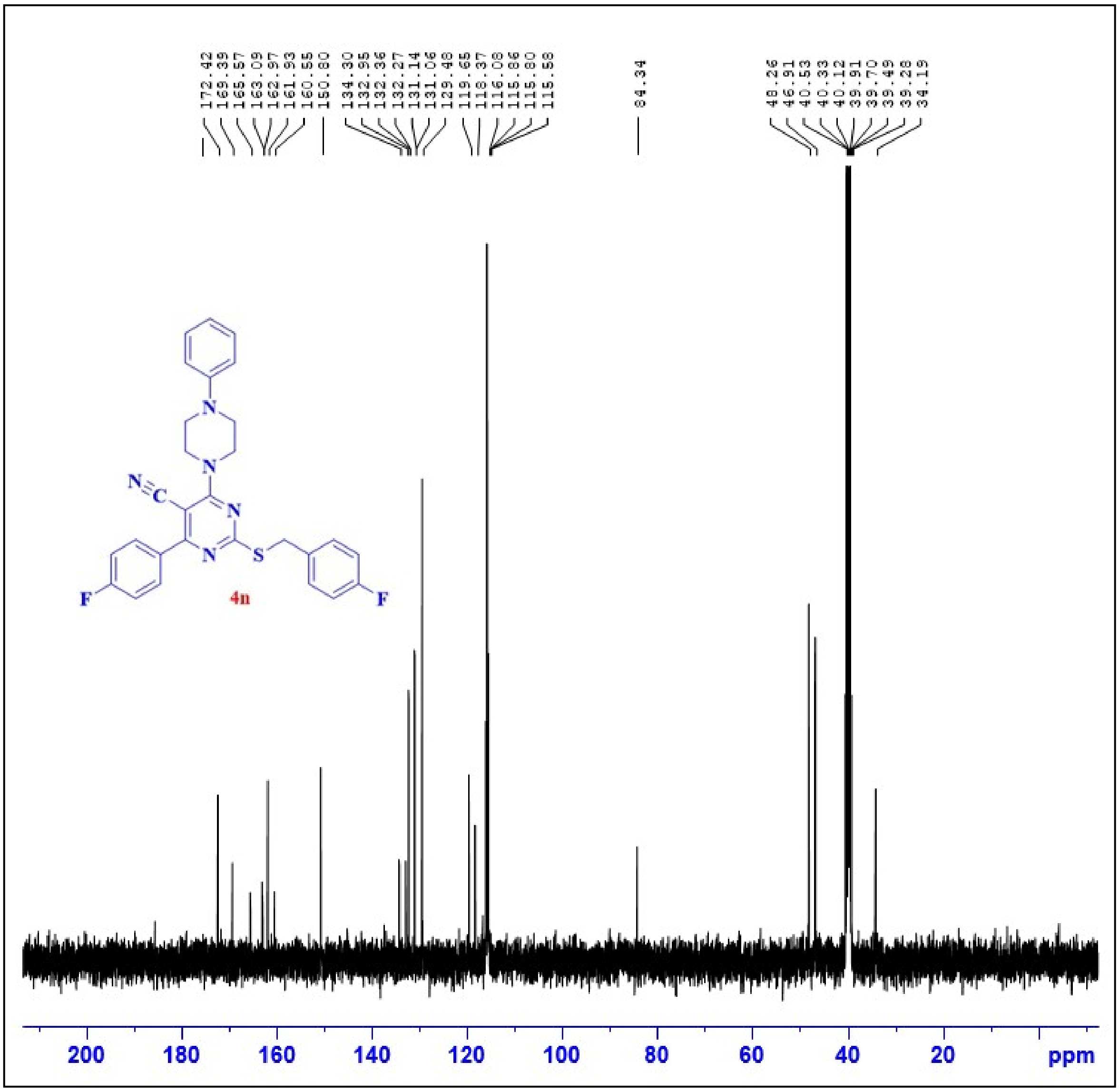
172.42
169.39
165.57
163.09
162.97
161.93
160.55
150.80
134.30
132.95
132.36
132.27
131.14
131.06
129.48
119.65
118.37
116.08
115.86
115.80
115.58
84.34
48.26
46.91
40.53
40.33
40.12
39.91
39.70
39.49
39.28
34.19
N
N
C
N
N
S
F
F
4n
200
180
160
140
120
100
80
60
40
20
ppm

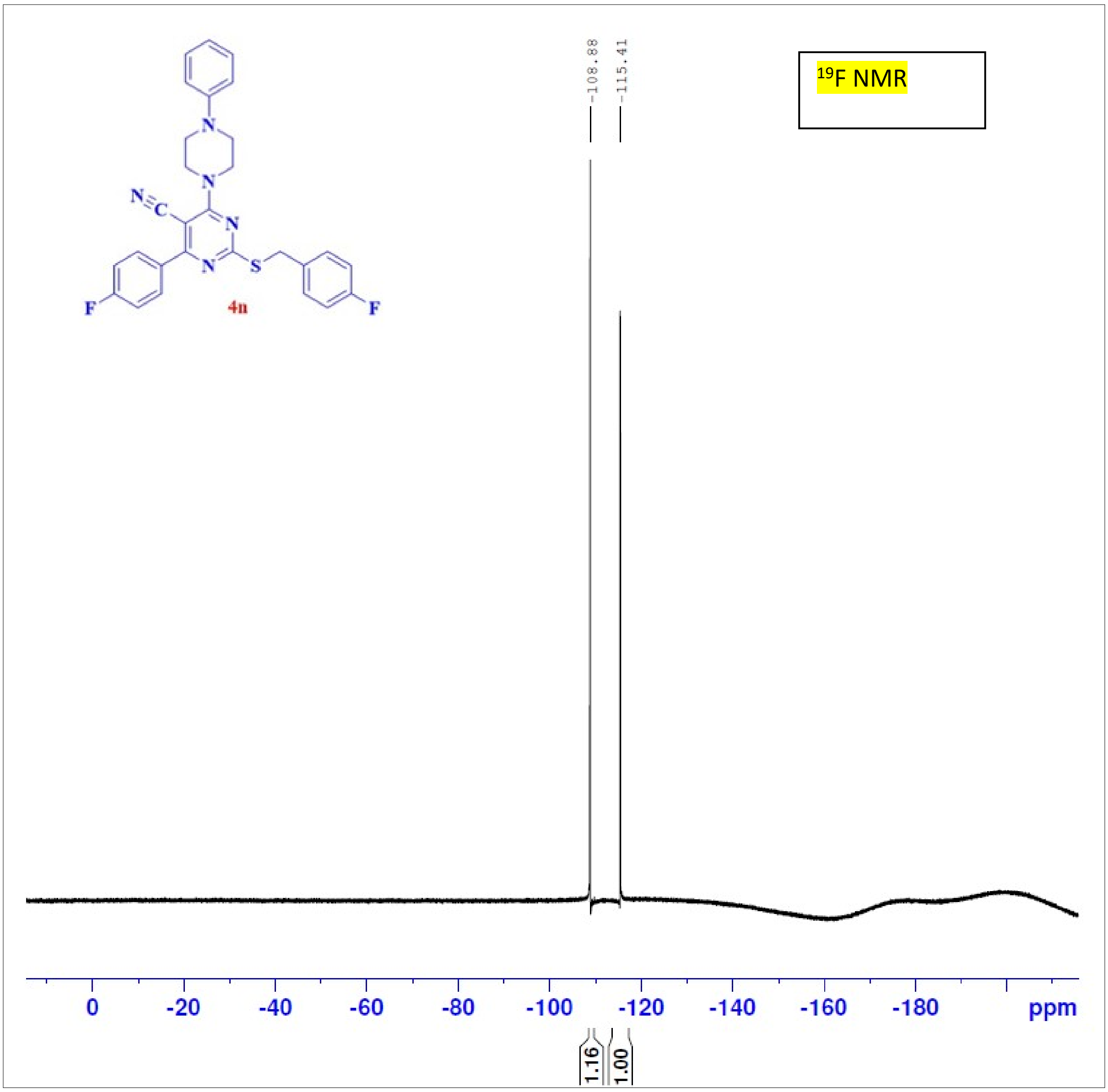

N
C
N
N
N
N
S
F
F
4n
-108.88
-115.41
19F NMR
0
-20
-40
-60
-80
-100
-120
-140
-160
-180
ppm
1.16
1.00

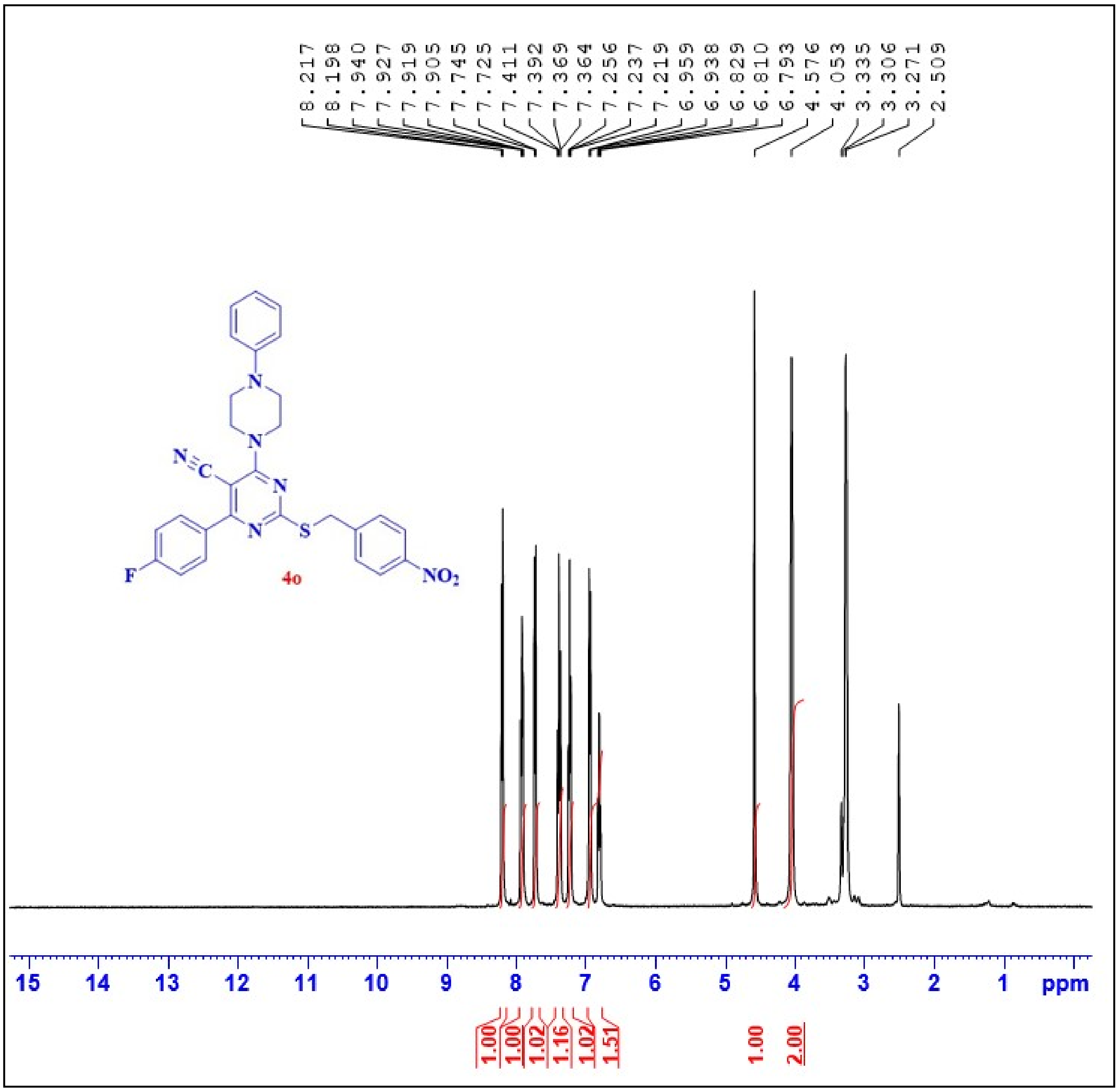
8.217
8.198
7.940
7.927
7.919
7.905
7.745
7.725
7.411
7.392
7.369
7.364
7.256
7.237
7.219
6.959
6.938
6.829
6.810
6.793
4.576
4.053
3.335
3.306
3.271
2.509
N
N
N
C
N
N
S
F
4o
NO_2
15 14 13 12 11 10 9 8 7 6 5 4 3 2 1 ppm
1.00
1.00
1.02
1.16
1.02
1.51
1.00
2.00

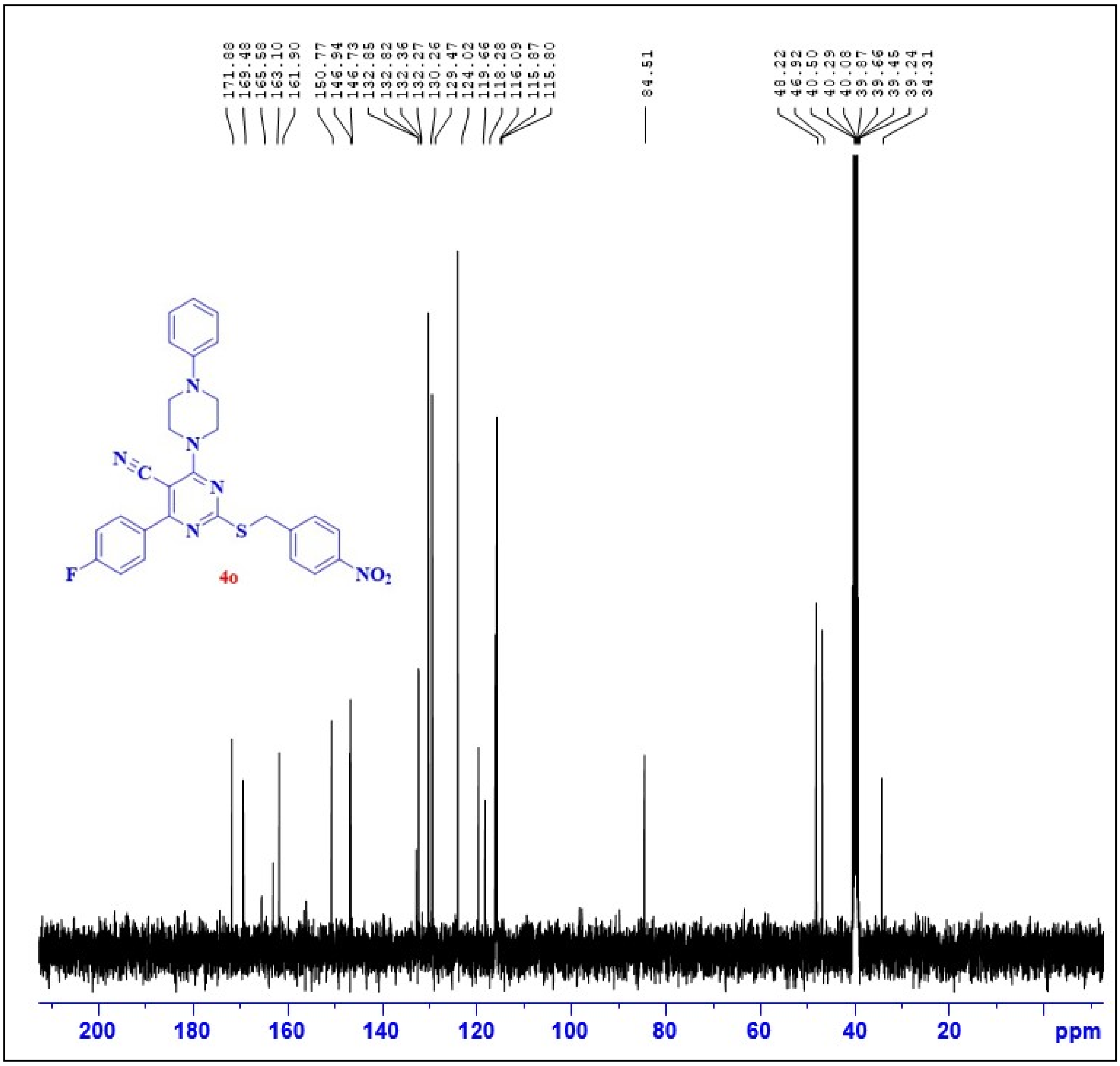
171.88
169.48
165.58
163.10
161.90
150.77
146.94
146.73
132.85
132.82
132.36
132.27
130.26
129.47
124.02
119.66
118.28
116.09
115.87
115.80
84.51
48.22
46.92
40.50
40.29
40.08
39.87
39.66
39.45
39.24
34.31
N
N
N
N
C
S
F
NO_2
4o
200
180
160
140
120
100
80
60
40
20
ppm

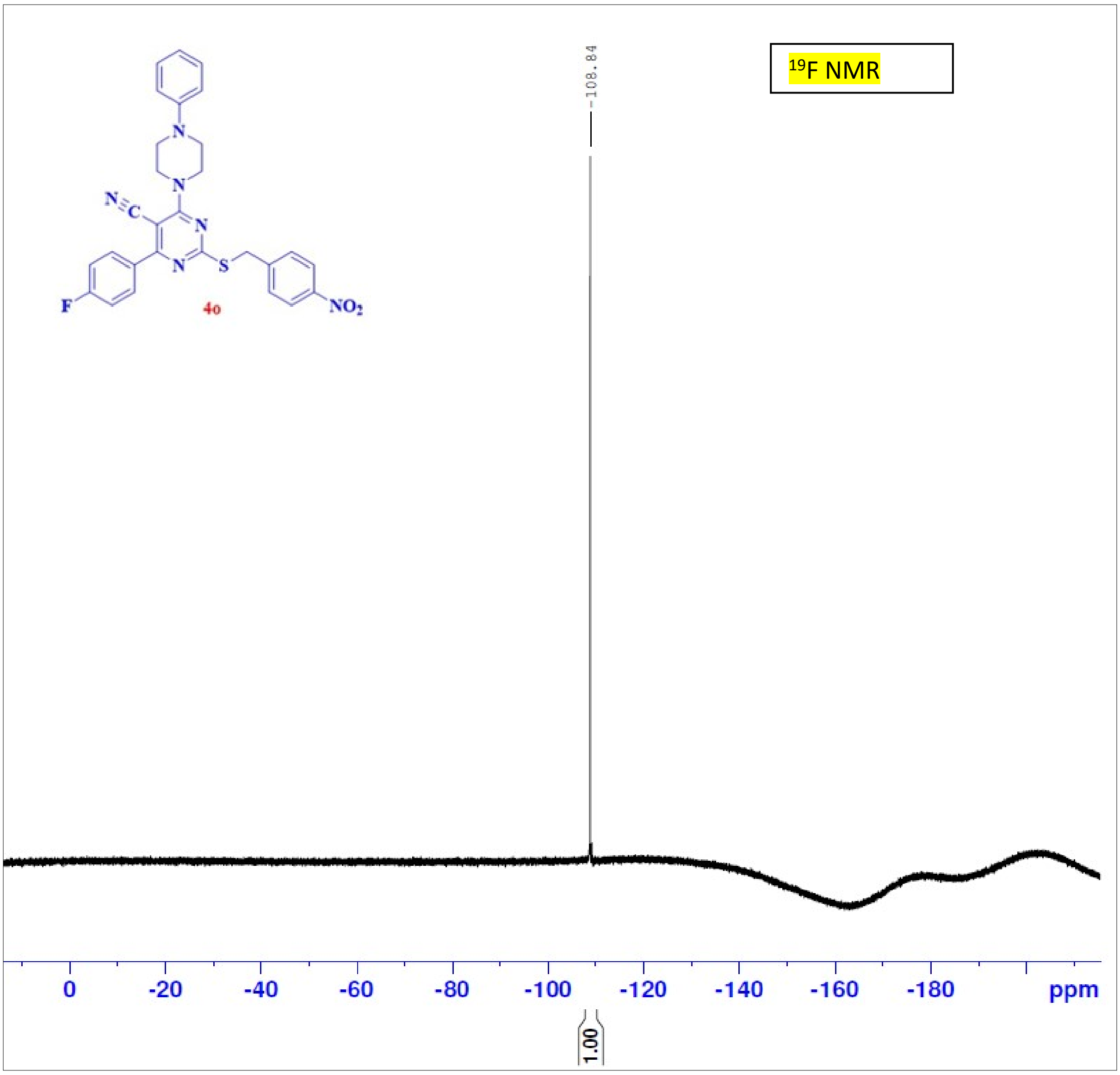

19F NMR
-108.84
4o
F
N
S
NO2
0
-20
-40
-60
-80
-100
-120
-140
-160
-180
ppm
1.00

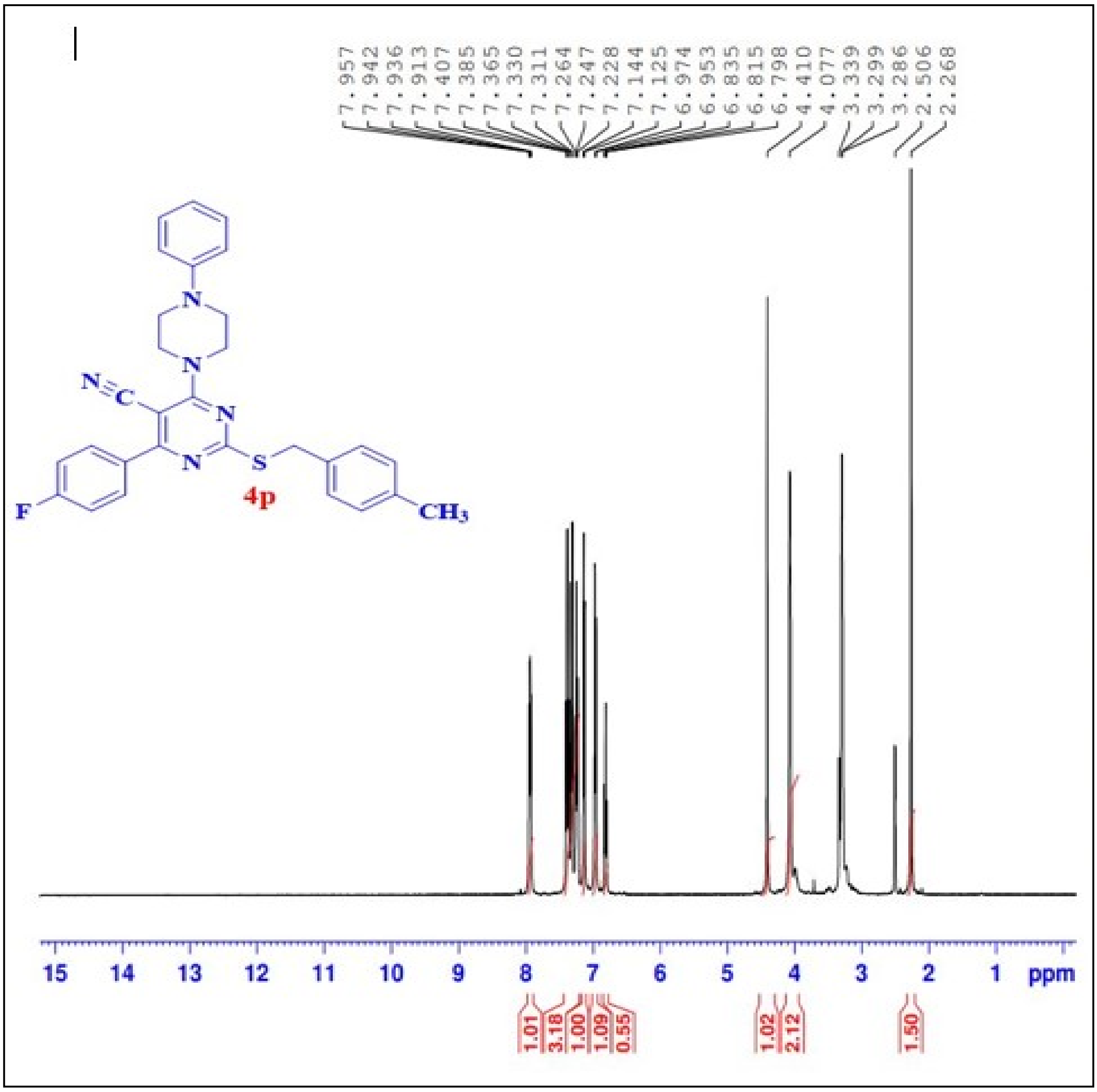
7.957
7.942
7.936
7.913
7.407
7.385
7.365
7.330
7.311
7.264
7.247
7.228
7.144
7.125
6.974
6.953
6.835
6.815
6.798
4.410
4.077
3.339
3.299
3.286
2.506
2.268
4p
15 14 13 12 11 10 9 8 7 6 5 4 3 2 1 ppm
1.01
3.18
1.00
1.09
0.55
1.02
2.12
1.50

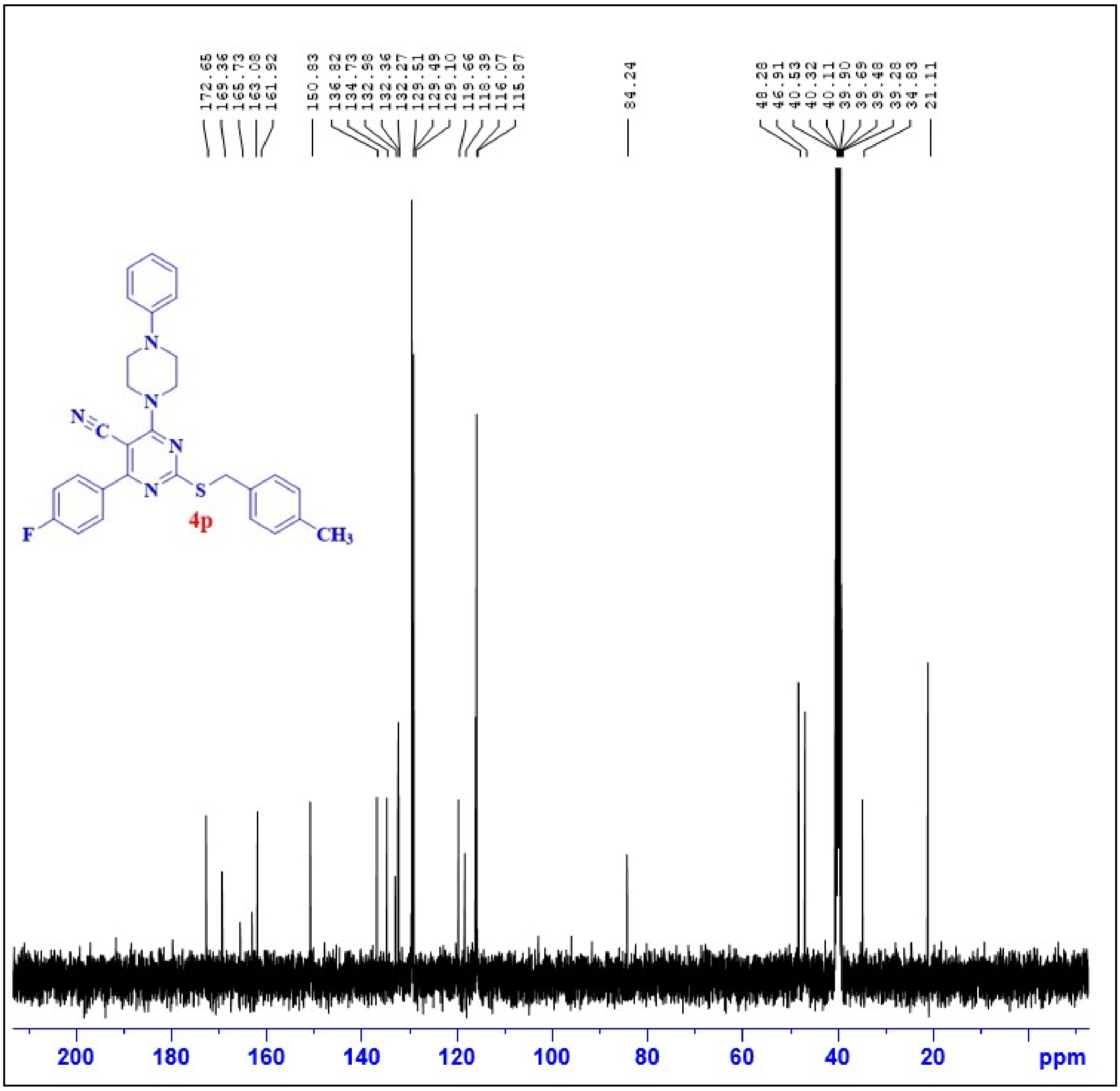
172.65
169.36
165.73
163.08
161.92
150.83
136.82
134.73
132.98
132.36
132.27
129.51
129.49
129.10
119.66
118.39
116.07
115.87
84.24
48.28
46.91
40.53
40.32
40.11
39.90
39.69
39.48
39.28
34.83
21.11
N
N
N
N
N
C
S
F
CH3
4p
200
180
160
140
120
100
80
60
40
20
ppm

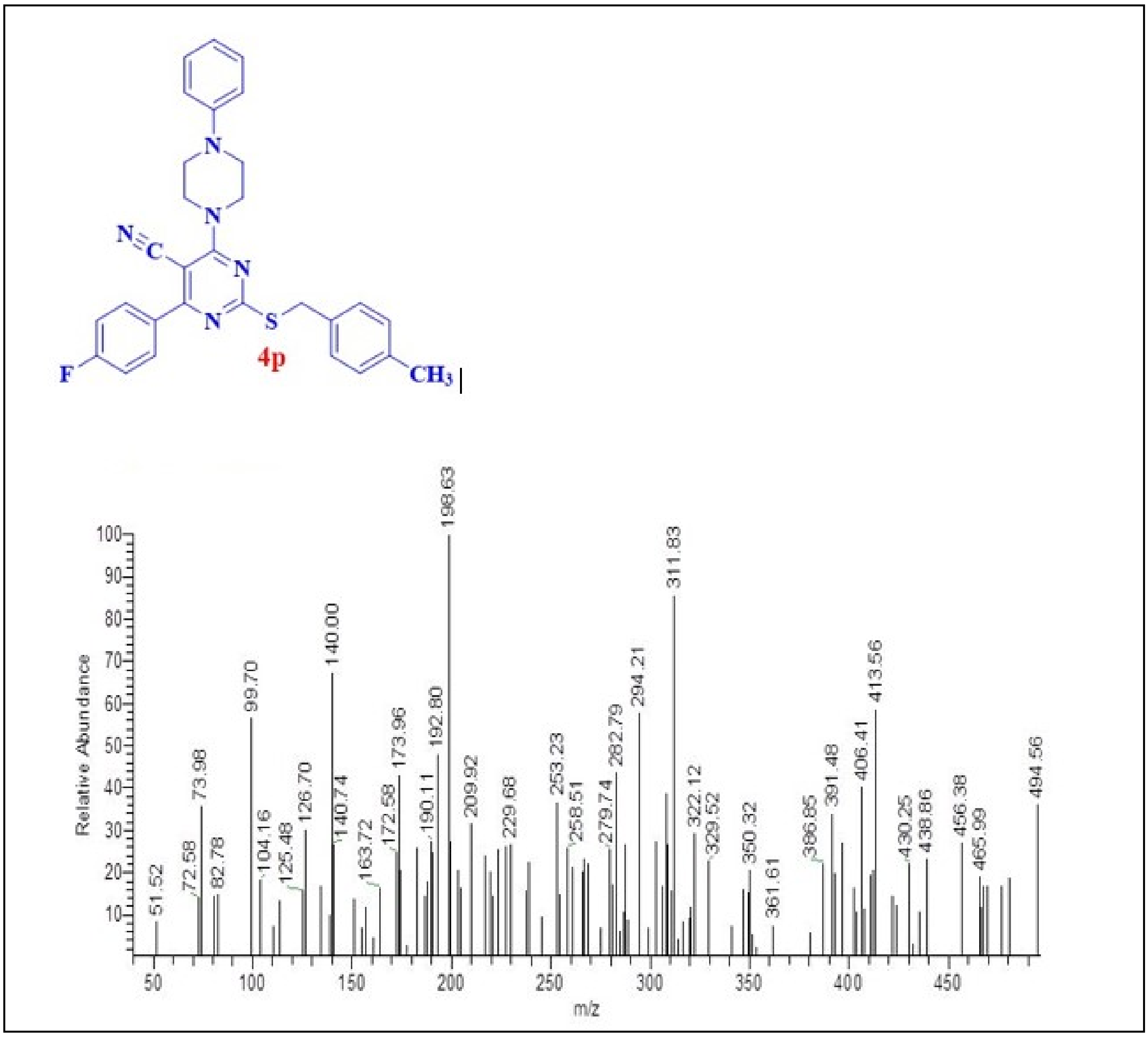

N
N
N≡C
N
N
S
F
4p
CH3
Relative Abundance
m/z
198.63
311.83
140.00
413.56
99.70
294.21
282.79
192.80
173.96
406.41
494.56
253.23
391.48
209.92
126.70
73.98
140.74
322.12
229.68
258.51
190.11
172.58
329.52
350.32
386.85
430.25
438.86
456.38
465.99
279.74
163.72
125.48
104.16
82.78
72.58
51.52
361.61

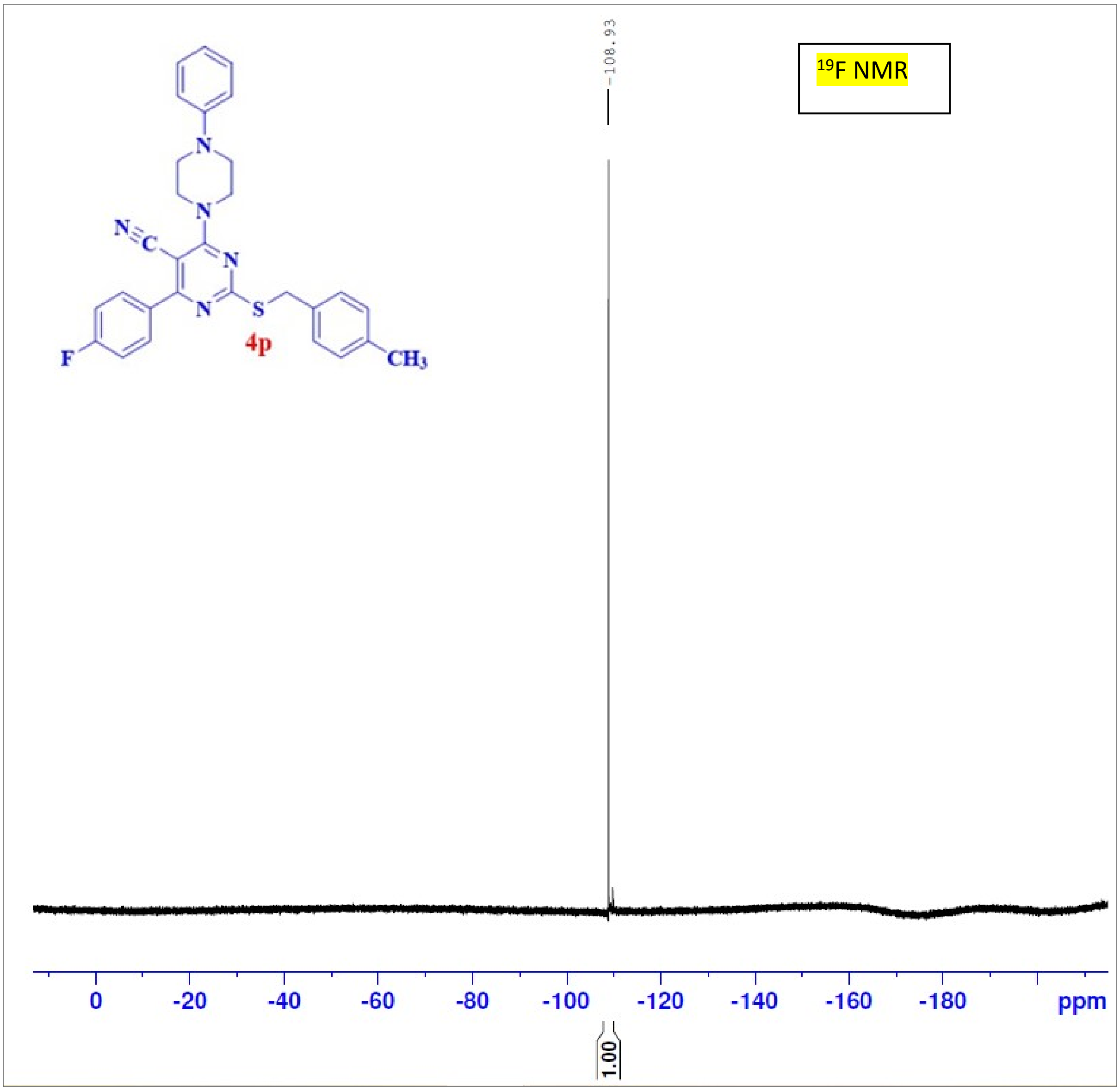

-108.93
19F NMR
N
N
N
N
N
C
S
F
4p
CH3
0
-20
-40
-60
-80
-100
-120
-140
-160
-180
ppm
1.00
