# Supplementary material for: Design, synthesis, and biological evaluation of new pyrimidine-5-carbonitrile derivatives as novel anti-cancer, dual EGFRWT/COX-2 inhibitors with docking studies
Source: RSC Adv. 2023 Nov 2;13(46):32296–320. doi: 10.1039/d3ra06088h (PMC10620772; doi:10.1039/d3ra06088h)
Supplement: RA-013-D3RA06088H-s002 [file RA-013-D3RA06088H-s002.pdf]

Electronic Supplementary Material (ESI) for RSC Advances.
This journal is © The Royal Society of Chemistry 2023

Design, Synthesis, and Biological Evaluation of New Pyrimidine-5-carbonitrile Derivatives as Novel Anti-cancer, Dual EGFR^{WT}/COX-2 Inhibitors with Docking Studies.

Nada Reda[a,*], Ahmed Elshewy[b,c], Hesham I. EL-Askary[e], Khaled O. Mohamed[b], Amira A. Helwa[a]

Supplementary 1

Table 1a: ***In vitro*** **growth inhibition percent (GI%) for the NCI60 cancer cell lines upon treatment with 10 µM of Compounds (2b-d, 3a-d and 4a-d)**

	**2b**	**2c**	**2d**	**3a**	**3b**	**3c**	**3d**	**4a**	**4b**	**4c**	**4d**
**Leukaemia**											
CCRF-CEM	-[a]	15.45	-	-	13.30	32.30	29.71	-	12.89	22.80	-
HL-60(TB)	20.88	49.95	43.38	44.03	49.83	57.98	51.77	31.31	15.01	17.28	-
K-652	-	31.45	-	11.78	30.20	44.30	42.83	16.46	16.20	23.12	10.67
MOLT-4	-	24.34	11.64	-	19.35	38.07	29.08	-	-	26.34	-
RPMI-8226	-	14.16	-	-	-	41.51	25.03	-	-	19.73	-
SR											-
**Non-small cell lung cancer**											
A549/ATCC	15.77	-	10.16	16.44	-	33.50	16.81	-	31.03	36.61	24.90
EKVX	-	12.04	-	-	10.91	20.38	20.92	-	39.92	60.74	13.10
HOP-62	16.84	-	-	12.77	-	-	-	-	**75.65**	65.34	36.77
HOP-92	19.25	33.01	-	14.74	25.21	45.40	34.25	15.08	65.67	63.28	34.62
NCI-H226	24.21	22.53	-	18.61	28.68	46.54	37.93	-	51.64	**76.22**	21.35
NCI-H23	12.73	-	-	-	-	12.20	-	-	28.19	25.17	-
NCI-H322M	7.12	-	-	-	-	13.28	-	-	21.55	17.30	-
NCI-H460	13.27	-	-	-	-	13.48	-	-	26.13	22.56	-
NCI-H522	25.94	30.52	12.91	16.01	25.38	37.48	29.73	16.81	45.02	58.72	44.57
**Colon Cancer**											
COLO 205	-	-	-	-	-	22.66	-	-	-	-	-
HCC-2998	-	-	-	-	-	12.17	-	-	12.64	-	-
HCT-116	33.39	22.23	-	22.79	18.51	40.70	25.73	10.37	33.96	35.64	19.81
HCT-15	-	13.42	15.77	-	13.00	45.64	24.97	-	20.05	27.19	-
HT29	-	37.56	10.72	-	13.44	61.63	51.51	-	-	13.41	-
KM12	-	-	-	-	-	27.77	19.05	-	12.78	15.07	-

SW-620	-	10.78	-	-	-	37.21	11.21	-	14.48	19.46	-
**CNS Cancer**											
SF-268	24.67	-	-	21.56	-	18.20	12.95	-	56.58	50.66	22.32
SF-295	25.73	10.37	-	27.87	10.01	29.81	11.51	-	63.10	58.52	32.25
SF-539	45.33	-	-	24.06	-	28.21	15.58	16.91	50.95	**73.77**	32.27
SNB-19	18.35	-	-	16.70	-	10.67	-	-	56.32	53.96	44.48
SNB-75	23.60	-	-	-	-	-	-	-	**82.51**	**92.38**	**88.04**
U251	33.40	13.78	-	39.15	-	31.17	15.75	-	52.85	44.68	13.13
**Melanoma**											
LOX IMVI	10.55	14.24	-	-	-	14.22	-	-	23.57	15.29	11.10
MALME-3M	-	-	-	-	-	-	-	-	41.38	57.24	26.17
M14	-	-	-	-	-	12.41	-	-	-	12.25	-
MDA-MB-435	-	-	-	-	-	-	-	-	-	16.03	-
SK-MEL-2	-	-	-	-	-	-	-	-	21.29	12.63	-
SK-MEL-28	-	-	-	-	-	-	-	-	14.16	17.90	-
SK-MEL-5	-	11.85	-	-	13.82	22.36	15.98	-	25.68	38.14	10.99
UACC-257	-	-	-	-	-	24.66	-	-	22.89	45.85	17.42
UACC-62	11.33	11.42	-	-	13.95	22.80	17.58	13.24	30.66	37.29	22.83
**Ovarian Cancer**											
IGROV1	-	-	-	-	-	-	-	-	-	19.45	-
OVCAR-3	-	-	-	-	-	13.71	-	-	24.80	30.28	-
OVCAR-4	**73.14**	-	-	49.74	-	18.42	14.97	-	39.53	42.76	20.15
OVCAR-5	-	-	-	-	-	-	-	-	16.89	-	-
OVCAR-8	48.04	-	-	27.14	10.81	16.16	-	21.20	45.16	46.89	23.86
NCI/ADR-RES	37.17	12.03	-	20.64	13.45	27.59	14.93	10.32	36.47	58.37	-
SK-OV-3	26.25	-	-	-	-	11.73	-	-	60.37	55.46	11.69
**Renal Cancer**											
786-0	**84.82**	-	-	38.26	-	21.29	12.30	-	**75.34**	69.78	55.92
A498	-	-	-	-	-	-	-	-	28.08	35.57	-
ACHN	26.83	-	-	45.51	-	14.60	-	-	53.35	48.98	37.30
CAKI-1	24.10	10.99	-	21.66	-	32.89	16.99	-	69.32	64.15	44.00
RXF 393	28.91	-	-	19.19	10.29	29.76	12.49	-	58.43	**90.21**	48.96
SN12C	17.08	14.07	-	-	19.06	29.81	27.59	-	27.12	25.44	-
TK-10	17.90	-	-	-	-	-	-	-	22.42	15.83	-

UO-31	26.71	11.53	-	14.25	12.89	32.10	17.01	-	13.97	49.11	10.06
**Prostate Cancer**											
PC-3	-	18.97	-	-	-	57.37	35.47	-	28.37	40.85	-
DU-145	-	-	-	-	-	24.93	18.41	-	22.16	26.65	-
**Breast Cancer**											
MCF7	-	-	10.82	-	17.40	27.06	12.55	-	36.26	41.49	11.76
MDA-MB-231/ATCC	39.26	-	-	21.72	-	-	-	10.65	49.52	52.93	31.92
HS 578T	24.34	-	-	16.76	-	13.31	-	19.87	**74.34**	**84.42**	48.85
BT-549	-	-	15.75	11.73	-	43.62	18.08	19.15	35.72	41.46	11.94
T-47D	25.99	23.03	13.50	10.38	33.18	68.67	46.84	15.89	26.75	44.55	12.46
MDA-MB-468	14.26	-	-	-	-	19.68	-	-	37.89	49.44	-
Mean	16.01	-	-	-	-	23.91	12.62	-	34.42	39.54	14.01

[a]GI % < 10

Table 1b: *In vitro* growth inhibition percent (GI%) for the NCI60 cancer cell lines upon treatment with 10 µM of Compounds (4a-p)

	4e	4f	4g	4h	4i	4j	4k	4l	4m	4n	4o	4p
Leukaemia												
CCRF-CEM	**L**[a]	**95.51**	-[b]	-	37.86	-	17.51	15.17	11.06	16.65	-	-
HL-60(TB)	**L**	**L**	-	-	36.50	-	-	12.21	-	-	-	-
K-652	**L**	**99.28**	-	-	73.15	18.51	23.28	28.64	10.59	12.49	-	17.97
MOLT-4	**L**	**92.21**	-	-	49.82	-	17.62	10.54	-	12.40	-	-
RPMI-8226	**L**	**L**	-	-	56.41	14.97	17.84	15.46	14.20	22.34	15.35	18.39
SR	**L**	**L**	-	-	**70.98**	-	-	-	-			
Non-small cell lung cancer												
A549/ATCC	**L**	**L**	-	-	38.33	27.72	18.72	13.07	-	-	-	11.63
EKVX	**L**	**98.71**	-	-	20.21	18.41	22.37	12.54	-	18.68	13.93	20.31
HOP-62	**L**	**L**	-	-	10.31	64.39	26.33	12.81	-	-	-	-
HOP-92	**L**	**L**	-	-	22.73	66.16	47.05	39.32	-	-	-	-
NCI-H226	**L**	23.08	-	-	-	37.79	-	-	-	-	-	-
NCI-H23	**L**	**L**	-	-	-	22.20	19.67	-	-	-	-	-
NCI-H322M	**L**	63.95	-	-	16	24.65	26.32	18.01	-	-	-	-
NCI-H460	**L**	**L**	-	-	35.06	-	-	-	-	-	-	-

NCI-H522	**L**	**L**	-	-	31.71	23.62	21.32	33.39	-	10.27	-	13.84
Colon Cancer												
COLO 205	**L**	**L**	-	-	13.56	-	-	-	-	-	-	12.23
HCC-2998	**L**	**L**	-	-	-	-	-	-	-	-	-	-
HCT-116	**L**	**L**	-	-	45.78	25.31	18.38	13.10	-	11.81	-	15.11
HCT-15	**L**	**L**	-	-	46.82	-	15.69	10.32	10.81	-	-	13.95
HT29	**L**	**L**	-	-	67.00	-	-	10.57	-	-	-	-
KM12	**L**	**L**	-	-	19.06	-	-	-	-	-	-	-
SW-620	**L**	**L**	-	-	10.04	-	-	-	-	-	-	-
CNS Cancer												
SF-268	**L**	58.46	-	-	26.82	15.77	-	-	-	14.13	-	15.26
SF-295	**L**	**L**	-	-	12.26	56.76	29.29	17.58	-	-	-	-
SF-539	**L**	**L**	-	-	14.45	54.78	40.98	31.78	-	-	-	-
SNB-19	**L**	50.36	-	-	10.01	40.28	29.58	14.28	-	-	-	-
SNB-75	**L**	**80.66**	15.86	19.47	41.03	**74.91**	25.71	35.62	-	15.56	12.45	14.84
U251	**L**	**L**	-	-	-	26.16	-	-	-	-	-	-
Melanoma												
LOX IMVI	**L**	**L**	-	-	25.45	22.86	20.90	12.85	-	-	-	-
MALME-3M	**L**	**L**	-	-	-	39.52	27.18	11.70	-	-	-	-
M14	**L**	**L**	-	-	18.15	10.37	-	-	-	10.14	-	11.03
MDA-MB-435	**L**	**L**	-	-	12.84	-	-	-	-	-	-	10.29
SK-MEL-2	**L**	**L**	-	-	-	-	-	-	-	10.64	-	15.10
SK-MEL-28	**L**	**L**	-	-	-	16.21	14.43	-	-	-	-	-
SK-MEL-5	**L**	**L**	-	-	33.65	-	16.39	-	-	12.43	-	13.68
UACC-257	**L**	**L**	-	-	27.77	-	-	-	-	12.89	-	14.05
UACC-62	**L**	**L**	-	-	19.60	22.49	19.78	12.96	16.18	25.83	18.11	29.69
Ovarian Cancer												
IGROV1	**L**	**L**	-	-	-	16.88	-	-	-	-	-	-
OVCAR-3	**L**	**L**	-	-	14.43	16.15	-	-	-	-	-	-
OVCAR-4	**L**	**L**	-	-	22.50	24.09	-	-	-	10.40	12.28	10.63
OVCAR-5	**L**	**99.16**	-	-	-	17.45	16.79	-	-	-	-	-
OVCAR-8	**L**	**L**	-	-	14.07	32.05	11.69	11.92	-	-	-	-
NCI/ADR-RES	**L**	**L**	-	-	-	42.93	25.06	12.67	-	-	-	-
SK-OV-3	**L**	43.30	-	-	-	55.50	20.93	-	-	-	-	-
Renal Cancer												
786-0	**L**	**L**	-	-	29.30	67.06	26.44	44.23	-	-	-	-
A498	**L**	-	-	-	12.50	-	-	-	-	-	-	-
ACHN	**L**	**L**	-	-	14.77	39.09	31.49	29.95	-	-	-	-
CAKI-1	**L**	**L**	10.98	11.67	37.93	57.49	34.76	36.94	10.65	16.34	13.69	16.64

RXF 393	**L**	**L**	15.23	-	49.72	**80.61**	68.50	42.40	-	-	-	-
SN12C	**L**	**L**	-	-	12.61	20.47	15.82	11.94	-	-	-	-
TK-10	**L**	**L**	-	-	-	14.97	-	-	-	-	-	-
UO-31	**L**	**91.74**	11.87	12.10	36.00	30.15	40.05	17.62	-			
Prostate Cancer												
PC-3	**L**	**88.74**	-	-	31.85	32.32	25.95	24.21	-	25.21	-	25.11
DU-145	**L**	**L**	-	-	-	-	-	-	-	-	-	-
Breast Cancer												
MCF7	**L**	**L**	-	-	30.78	14.77	17.63	12.79	16.08	24.84	14.77	21.28
MDA-MB-231/ATCC	**L**	**L**	-	-	15.90	45.82	35.12	28.90	-			
HS 578T	**L**	**L**	-	10.10	32.04	60.43	42.57	31.10	-	-	-	-
BT-549	**L**	17.34	-	-	-	32.05	46.49	10.77	-	-	-	-
T-47D	**L**	**L**	-	-	42.49	18.27	28.64	18.05	15.11	24.33	-	12.29
MDA-MB-468	**L**	**L**	-	-	29.17	23.57	12.43	-	-	-	10.88	13.55
Mean	**L**	**L**	-	-	24.31	25.08	17.52	11.17	-	-	-	-

[a] GI % > 100

[b]GI % < 10
